# Microwave-Assisted One-Pot Synthetic Pathways for Pyrido[2,3‑*d*]imidazole Derivatives

**DOI:** 10.1021/acs.joc.5c01864

**Published:** 2026-02-06

**Authors:** Bartosz Orwat, Sylwia Raczak, Kamila Jankowska, Grzegorz Krajewski, Julita Nawrocik, Maciej Kubicki, Rafał Januszewski, Beata Łuszczyńska, Ireneusz Kownacki

**Affiliations:** a Department of Molecular Physics, Faculty of Chemistry, 205634 Lodz University of Technology, Żeromskiego 116Żeromskiego 116, Łódź 90-924, Poland; b Faculty of Chemistry, 467899 Adam Mickiewicz University in Poznań, Uniwersytetu Poznańskiego 8, Poznań 61-614, Poland; c Center for Advanced Technologies, Uniwersytetu Poznańskiego 10, Poznań 61-614, Poland

## Abstract

Herein, we report efficient microwave-assisted one-pot protocols, enabling the efficient synthesis of a broad scope of functionalized pyrido­[2,3-*d*]­imidazoles. The developed methodologies allow obtaining target compounds using cheap reagents and catalysts, in very short reaction times, and perform three-step reactions in the same vessel without the need of intermediate isolation. The obtained pyridoimidazoles equipped with alkyl, alkoxy, hydroxy, halogen, acyl, and amino substituents were fully characterized and constitute useful materials for coordination chemistry or building blocks for further transformations.

## Graphical abstract

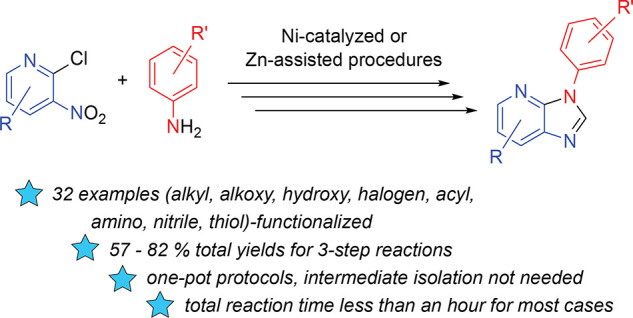

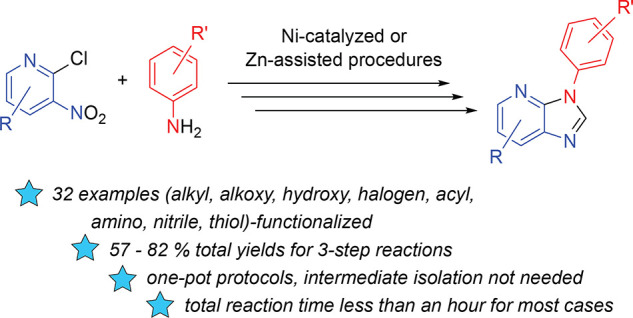

Pyrido­[2,3-*d*]­imidazole derivatives are an interesting group of compounds made of two fused heterocyclic rings, i.e., imidazole and pyridine, with increasing application potential in various areas of chemistry, medicine, and materials engineering. So far, pyridoimidazole derivatives have been reported as antimicrobial agents, proton pump inhibitors (PPIs), and cardiotonic drugs.^1^ Their broad pharmacological profile can be attributed to their similarity to purine.^2^ However, their most interesting application nowadays is the synthesis of iridium­(III) complexes, in which these compounds act as *C*,*C*-cyclometalated *N*-heterocyclic carbene (NHC) ligands. Due to their unique photophysical and emissive properties, they have been intensively studied for their applications as phosphorescent emitters for deep blue organic light-emitting diodes (OLEDs) or sensitizers for hyperphosphorescent OLEDs.^3^ The latter is extremely important as it could revolutionize the OLED market by solving the problem of missing both efficient and stable pure blue emitters.^4^ So, providing efficient methods for the synthesis of various pyridoimidazoles, also bearing substituents with different electronic properties in their structure, would be very appreciated.

Medically applied pyridoimidazoles are often substituted at the *C*-position of the imidazole moiety, whereas this position must bear a C–H bond if their use as a NHC ligand for phosphorescent materials is desired. The latter variant is the only target of this work. According to the literature, such pyridoimidazoles were often obtained starting from 3-chloro-2-nitro-pyridines and anilines via three consecutive reactions, namely, nucleophilic substitution, nitro group reduction, and imidazole ring formation.^2,5^ A few reported syntheses of pyridoimidazoles involved both the reduction and ring closure occurring at the same stage.^3a,3b,6^ This was due to the presence of the reducing agent and formic acid in the reaction system, the latter taking part in both reduction and ring closure. However, these procedures were based on fully stoichiometric reactions, and their disadvantage was the huge excesses of the applied reagent and solvent volume. Moreover, the reaction times were quite long, and all intermediates were isolated, increasing the workload and time spent on the synthesis.

Analysis of the mentioned reports brought us to the conclusion that the synthesis of the pyridoimidazoles can be significantly improved, and there is a need to develop a protocol for the obtainment of a broad scope of functionalized pyridoimidazoles. Our first idea was that nitro group reduction and imidazole ring formation could be catalytically performed, provided that formic acid or its derivatives are involved. A promising system was palladium on activated carbon, accompanied by formate salts.^7^ We found it to be very efficient during initial tests but only for compounds with nonreactive substituents. Once the halogen or other substituent bearing multiple bonds were introduced into the structure, their almost total degradation was observed. Then, we decided to examine a less reactive hydrogenation catalyst analogue to Pd/C, namely, Raney nickel.^8^ The accompanying ammonium formate was found ineffective in our initial tests, contrary to triethylamine formate.^9^ The nickel catalyst showed a less reactive nature; therefore, a higher reaction temperature and higher catalyst loading were necessary, but the process proceeded selectively in the presence of functionalized substrates. Additionally, this catalyst is much cheaper compared to the expensive palladium. Comparison of the reagents used for the Ni-catalyzed stage and the postreaction mixture of nucleophilic substitution showed that both systems should be compatible, and no negative interaction is expected. Thus, we concluded that the pyridoimidazole synthetic path can be carried out according to a one-pot protocol, with all the three-step reactions in the same vessel without isolation of any intermediates. Finally, the whole process could be accelerated by microwave irradiation as a heat source, leading to a shortening of the reaction time, according to our positive experience.^10^ The purpose of all of the proposed innovations was to develop a useful protocol for the quick and efficient synthesis of a broad scope of functionalized pyridoimidazoles. Hereby, we report the details of our studies. An initial version of this work was deposited in ChemRxiv on July 24th, 2025.^17^

## Results and Discussion

After the potential synthetic pathway was designed, aniline and 2-chloro-3-nitropyridine were selected as the model reagent system for optimization (Scheme 1). The first step of the proposed procedure was nucleophilic substitution. The reaction between these two compounds occurred readily when the mixture was heated up, as observed by the mixture turning red, a typical color of 2-anilinosubstituted-3-nitropyridines. However, the mixture was spread on the vessel’s internal surface while stirring and solidified there, hampering the mass transfer and causing local overheating. To counteract this, a small amount (3 equiv) of a moderately volatile alcohol, i.e., ethanol, was selected as an additive to provide reflux conditions.^11^ A slight excess of aniline was proposed, the solvent was selected, and the reaction time was arbitrarily set to 10 min according to our routine, so the remaining parameter to be optimized was the reaction temperature. The yields of the first stage of the model reaction system are shown in Table 1 (entries 1–3). As one can see, the best yield of the isolated nitro-intermediate **1i** was observed at the highest temperature tested, namely, 180 °C. However, the obtained product was much darker compared to other samples, suggesting contamination with degradation byproducts. That high temperature also caused a significant degradation of many functional groups in further reagent scope tests. These issues were not observed in almost all the cases examined when the reaction temperature was lowered by 20 °C, while the yield of the model reaction decreased by only 1%. Therefore, conducting the first stage at 160 °C was selected as a good compromise between selectivity and conversion. Subsequent reagent scope tests revealed that the substitution is strongly affected by the electron-donating/withdrawing character of the aniline substituents. For example, electron-donating methoxy groups enabled complete conversion in just a few minutes, whereas electron-withdrawing nitrile groups caused the heating time to be extended significantly to more than 10 min. So, the proposed reaction time for the general procedure was extended to 30 min. It did not affect the reaction yield negatively but made the procedure more versatile.

**Scheme 1:**
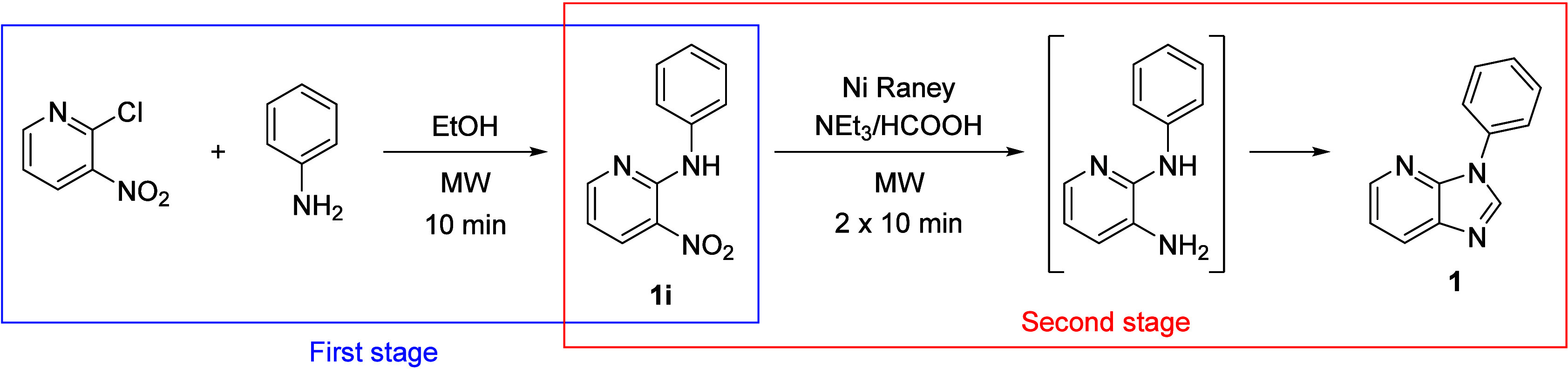
Model System Used for Optimization

**Table 1:** Optimization Results of the Model Reagent

entry	reaction temperature [°C]	catalyst loading [mol %]	yield [%]
first stage			
1	140		77^a^
2	160		96^a^
3	180		97^a^
second stage			
4	100	30	7^b^
5	120	30	22^b^
6	140	30	59^b^
7	160	30	70^b^
8	140	40	69^b^
9	140	50	77^b^
10	140	60	83^b^
11	140	70	80^b^

a Isolated yield.

b GC yield.

Initial tests of Raney nickel in combination with a triethyl amine/formic acid mixture suggested that such a catalytic system was capable of not only nitro group reduction but also imidazole ring closure. So, a systematic optimization of the second stage was implemented (Scheme 1), starting from the evaluation of the temperature effect on the yield of the target product. Therefore, catalytic tests were carried out in the temperature range of 100–160 °C for an arbitrarily selected 10 min reaction time, and the reaction progress was monitored using gas chromatography. It was found that the expected reaction boosted above 120 °C and that the catalytic system was still active after heating for 10 min. The extension of the reaction time did not change the outcome much, but repeating the heating after the addition of an extra 5 equiv of formic acid improved the yield. This action was also beneficial because of significant pressure buildup as a consequence of carbon dioxide formation, the nitro group reduction byproduct. So, the pressure was released, and the reaction could be continued safely. Thus, all catalytic tests were repeated with this additional treatment and the results are presented in Table 1 (entries 4–7). It was observed that the higher the reaction temperature, the better the product yield. At that point, a few anilines bearing functional groups were briefly tested for the reaction selectivity. It was found that some of them (e.g., those bearing chloro and bromo substituents) underwent degradation at 160 °C, while they remained intact at 140 °C. To maintain the versatility of the general procedure, a lower reaction temperature (140 °C) was chosen for further optimization. Because catalyst deactivation was observed and both the remaining substrate and the intermediate were still present in the reaction mixture, the nickel catalyst loading was chosen as the next target for optimization. Since 30 mol % of the Ni catalyst was not enough, higher loadings were also tested (Table 1, entries 8–11). The product yield was correlated to the catalyst loading according to expectations. Complete disappearance of the substrate and amino-intermediate was observed when 60 mol % was applied (entry 10), which provided the highest yield of **1**. A higher catalyst loading resulted in a slightly lower yield (entry 11), presumably due to the coordination of the product. Summarizing this paragraph, a complete set of reaction conditions enabling efficient formation of the model pyridoimidazole **1** was established. Eighty-three percent of the total yield for the three-step reaction procedure (nucleophilic substitution, nitro group reduction, and imidazole ring closure) can be considered a very satisfactory result.

Having the general procedure optimized, a broad scope of commercial anilines bearing various functional groups was subjected to the optimized reaction conditions (Scheme 2). We started the examination with alkyl- and alkoxy-substituted anilines at various positions and up to three substituents per molecule. Since these substituents are electron-donating, the nucleophilic substitution occurred rapidly and efficiently, as confirmed by GC-MS analyses. This was manifested by the appearance of the vivid red color during the preparation of the reaction mixture when the two reagents came into physical contact. Among them, only 3,4,5-trimethoxyaniline required a lowered temperature of the first stage; otherwise, detaching of the methoxy group occurred. No problems were observed in the second stage, and compounds **2**–**8** were obtained in 71–81% yields. Hydroxy-substituted **9** and **10** were prepared according to the general procedure, but the yields were lower, presumably owing to the interactions of acidic −OH with other reagents. Then, we evaluated protocol compatibility with the −OCF_3_ group, successfully obtaining **11** and **12** with around 80% yields. Encouraged by that, we tested other anilines equipped with fluorine substituents, which led to the successful formation of **13**–**18**, materials potentially useful for phosphorescent emitters.^12^ Only **18** required a lower temperature in the first stage. Otherwise, −CF_3_ was converted to the −COOEt groups. Issues were encountered when heavier halogens were present in the reagents’ structure. As mentioned in the optimization section, significant dehalogenation of chloro- and bromo-substituted compounds was observed in the second stage when the reaction temperature was 160 °C. However, the use of a lowered temperature in the second stage allowed mitigating the problem; thus, **17** and **19**–**22** were obtained successfully, constituting very attractive building blocks for further transformations. Finally, acyl-functionalized **23** and **24** were synthesized smoothly according to the general procedure, while amino-functionalized **25** required a decreased temperature during the first stage to increase the substitution selectivity. The chemical structures of all of the compounds reported here were confirmed by NMR and ESI-HRMS techniques (Figures S1–S105).

**Scheme 2:**
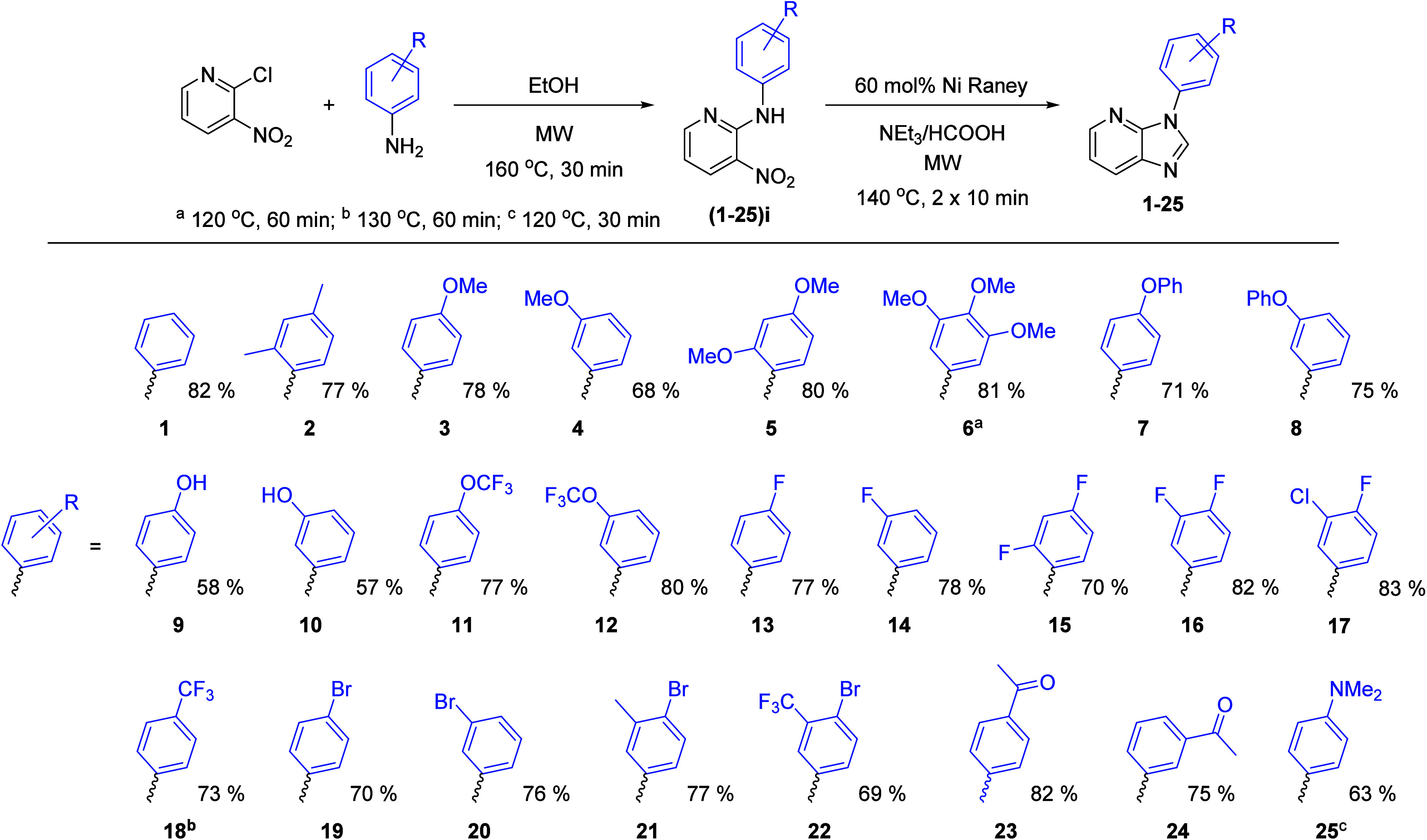
Ni-Catalyzed Pathway and the Scope of the Obtained Pyridoimidazoles

During the exploration of different aniline scopes, it was found that for some of them, the catalytic reduction step was unacceptably nonselective or did not proceed at all. The latter was observed for 4-(methylthio)­aniline, presumably attributed to the fact that thiols are known poisons to metallic catalytic systems.^13^ In search of a solution, alternative pathways for the nitro group reduction were examined.^14^ The most convenient revealed to be the use of zinc powder in the presence of water as a hydrogen source, contrary to many reports on acidic environments.^15^ Neutral pH was essential for this process, as it did not proceed selectively in the presence of ammonium chloride, hydrogen chloride, or formic acid. This system smoothly converted the nitro intermediates into their amine counterparts. The reduction could be easily observed by the disappearance of the intensely red substrate, leading to an almost colorless solution. After successful reduction, the final stage was carried out using triethyl orthoformate, acting as a =CH– source for the ring closure.^16^ Its advantage was the ability to consume excess water, which could hamper the reaction progress. The determination of the reagent excess enabling total conversion finished the development of an alternative protocol for the preparation of the problematic pyridoimidazole **26**, as presented in Scheme 3. The use of the Zn-assisted pathway was also required in the case of 4- and 3-aminobenzonitriles. Their corresponding nitro intermediates underwent very nonselective reduction under Ni-catalyzed protocol conditions, showing the presence of the nitrile reduction products and their subsequent formylation in the postreaction mixtures. Moreover, it was evident that the reduction process was hampered, presumably due to the strong coordinating behavior of the −CN groups. A lowered selectivity for the compounds bearing the nitrile group was observed even earlier at the nucleophilic substitution stage. When the usual 3 equiv of ethanol was applied, a slight amount of side products of the addition of EtOH to the C≡N bond was noticed. The exclusion of ethanol from the reaction mixture was an obvious solution, but it led to very poor conversions. Its replacement with different solvents (DMF, acetonitrile, or NMP) resulted in very poor conversions, too, proving the essential role of the alcohol. Finally, the best outcome was achieved by adopting a compromise approach, which consisted of reducing the ethanol content to 1 equiv and replacing the remaining volume with NMP. This provided maximal conversion and ceased the unwanted ethanol addition process. The remaining step of the synthesis was carried out according to the Zn-assisted pathway, resulting in target CN-functionalized pyridoimidazoles **27** and **28** (Scheme 3). The reduced yields can be attributed to the bonding of the products to the Zn species, discarded during the postreaction mixture processing.

**Scheme 3:**
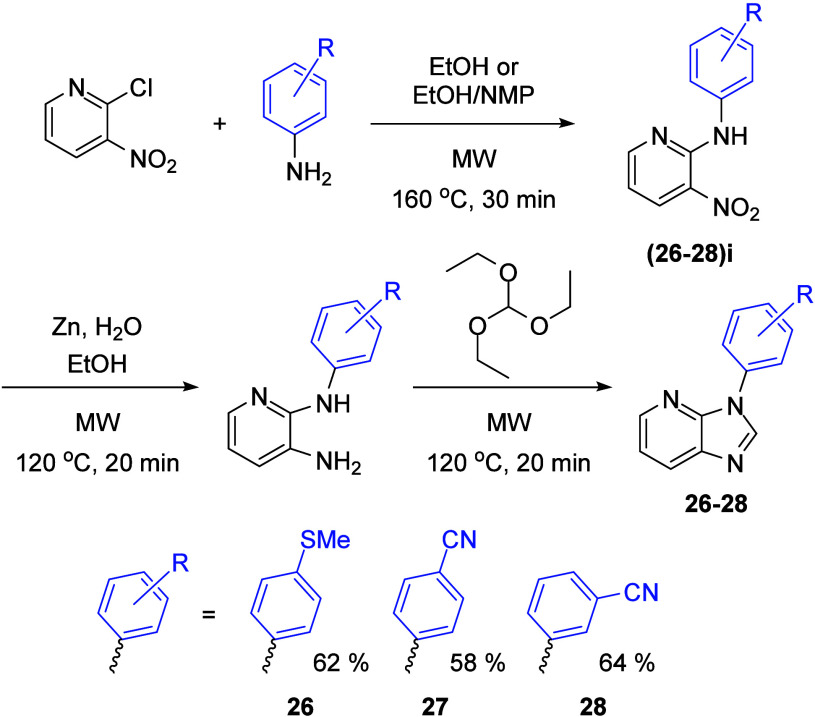
Alternative Zn-Accompanied Pathway for Alkylthio- and Nitrile-Functionalized Pyridoimidazoles

After the applicability of different anilines was evaluated, the change in the chemical structure of the second reagent was addressed. Therefore, a set of commercially available 2-chloro-3-nitropyridine derivatives was subjected to the developed Ni-catalyzed procedure. Fortunately, 2-chloro-4-methyl-3-nitropyridine, 2-chloro-5-methyl-3-nitropyridine, 2-chloro-6-methoxy-3-nitropyridine, and 5-bromo-2-chloro-3-nitropyridine underwent the desired transformation smoothly, leading to the target products (Scheme 4). It should be emphasized that the substrate bearing a bromo substituent was also selectively processed, leading to highly valuable building block **32.**

**Scheme 4:**
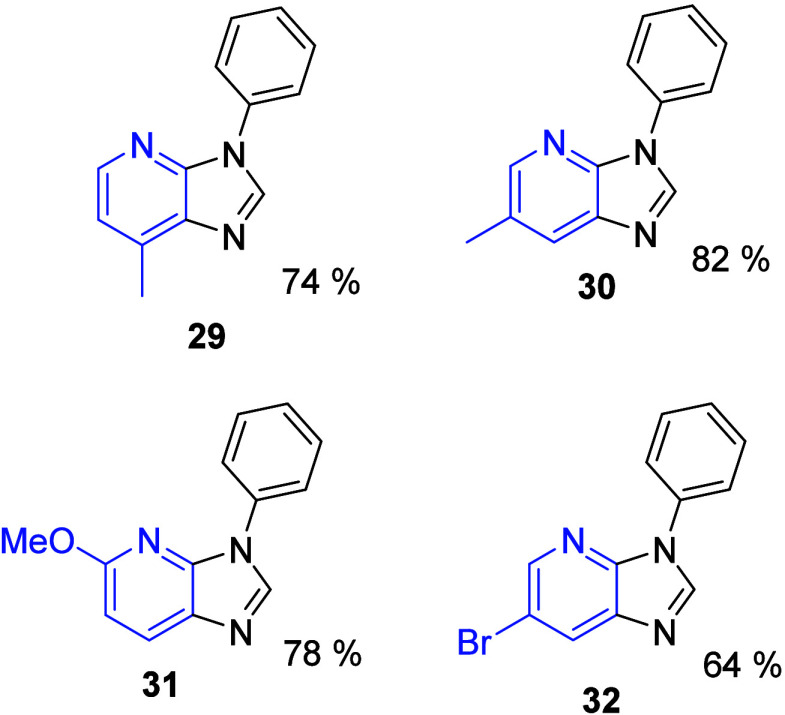
Chemical Structures of the Pyridoimidazoles Derived from Functionalized Pyridine-Based Reagents

Both developed protocols involved the formation of nitro-intermediates **(1**–**25)­i**, the products of the nucleophilic substitution reaction. It was very common that these 2-amino-3-nitropyridines formed crystals suitable for single-crystal XRD analyses right after the vessel was picked up from the microwave reactor. These compounds were not essential from our point of view, so they were not isolated. Moreover, we consider it an advantage that the whole synthetic route can be completed continuously in the same vessel, without isolation of any intermediate. However, 14 crystal structures of the mentioned intermediates were determined by taking advantage of the crystals formed. The exemplary crystal structure of **11i** is shown in Figure 1a, while the rest of them and the crystallographic details are provided in the Supporting Information. A common feature of these compounds was the presence of a hydrogen bond between an oxygen atom of the nitro group and a hydrogen atom of the secondary amine, which stiffens the structure. Almost all of the structures showed coplanarity of the aromatic rings, with minor variation in their torsion. Following the established synthetic protocols, several crystalline samples of the target pyridoimidazoles were formed during their purification. The resolved crystal structures for six compounds serve as evidence that the desired species were obtained successfully. It can be concluded that the coplanarity of the aromatic moieties was much more distorted compared to that of the nitro-intermediates. It was surprising to see that the change of =CH– to =N– should result in lowered steric hindrance compared to benzimidazoles.^3g^ An exemplary crystal structure of **12** is presented in Figure 1b, while the remaining ones are shown in the Supporting Information.

**Figure 1:**
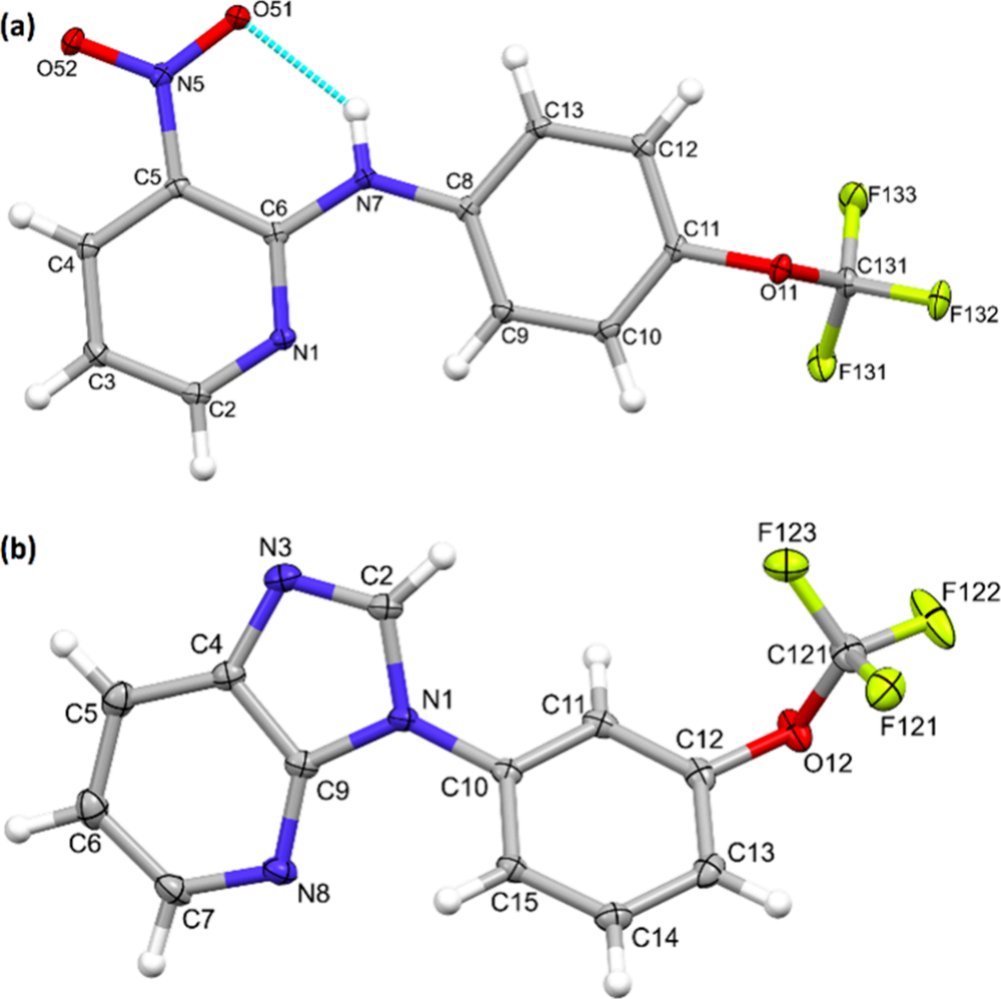
Crystal structures of **11i** (a) and **12** (b).

Another aspect investigated in this study was the reaction mechanism. Among the obtained pyridoimidazoles, only **31** required an extended reaction time in the second stage due to inhibited ring closure. After a standard applied heating time (2 × 10 min), the GC-MS analysis indicated only the target product and a significant amount of an unidentified additional product, the *m*/*z* value of which suggested that it might be a formylated amine intermediate. That was the only case where such a stable intermediate was observed in GC-MS analyses, proving that the electron-donating substituents of the pyridine moiety can modulate the ring closure. Taking the above into account, it seems that formylation is the first step in imidazole ring formation, as this intermediate was consumed over the extended reaction time and the target product content increased. Observation of that intermediate prompted us to thoroughly explore the reaction mechanism. We believe that there is no need to explain nucleophilic substitution, as this process is well known. Instead, we focused on the processes occurring in the second stage of the synthetic procedure in the presence of the Ni/NEt_3_/HCOOH system. The interesting intermediate was isolated from the postreaction mixture after the synthesis of **31** using column chromatography. Material suitable for single-crystal XRD analysis was obtained by evaporation of a dichloromethane solution. The solved crystal structure (Figure 2b) clearly showed that the intermediate was a formamide derivative **31i-CHO**, indicating that the preceding process must be the reduction of the nitro group to the corresponding amine (Figure 1a). Formylation occurred at the primary amine group, as it is less hindered than that at the secondary −NH– group. The NMR analysis of **31i-CHO** crystals revealed its tautomerism, showing two sets of signals corresponding to the aldehyde and enolic forms (Figure 2a,c). Tautomerism was also confirmed by the equilibrium shifting observed in NMR spectra recorded in solvents of different polarities (compare Figures S74 and S75). Further heteronuclear single quantum coherence (HSQC) studies enabled assignment of the signals to each form. The most downfield-shifted carbon signal (δ 164.14 ppm), corresponding to the carbonyl group of the aldehyde form, was coupled with the doublet hydrogen signal at δ 9.28 ppm. Thus, the neighboring singlet hydrogen signal at δ 9.47 ppm must originate from the enolic form, and indeed, it was coupled with the carbon signal at δ 160.8 (Figure S77). The latter carbon signal is expected to be slightly upfield-shifted compared to the formyl group at δ 164.14 ppm due to the increased electron density on this carbon atom caused by the extended conjugation with the pyridine moiety. A similar case was reported in the literature.^18^ As the final step in the mechanism, **31i-CHO** must undergo cyclization and water elimination to yield the target pyridoimidazole **31**. The last process was monitored using NMR spectroscopy by **31i-CHO** NMR sample heating. No target compound formation was observed at 100 °C. However, after increasing the temperature to 140 °C and extending the heating time, a conversion of **31i-CHO** of target **31** was observed (Figure S78). However, a complete conversion was not achieved even after 12 h of heating at 140 °C. To gain a better perspective, the corresponding formylated intermediate, **1i-CHO**, lacking the methoxy group, was synthesized and subjected to the same NMR studies and thermal treatment. This time, a much faster and complete cyclization to **1** was observed (Figure S81), proving that the presence of an electron-donating group in the *para* position to the formamide moiety is responsible for the increased stability of **31i-CHO**. Notably, the formation of the imidazole ring occurs much faster in the Ni/NEt_3_/HCOOH system, compared to the DMSO environment (minute vs hour time scale) for both **1i-CHO** and **31i-CHO**. Finally, a complete reaction mechanism occurring in the presence of the Ni/NEt_3_/HCOOH system is shown in Figure 2a. We believe that the findings revealed contribute to the development of organic chemistry.

**Figure 2:**
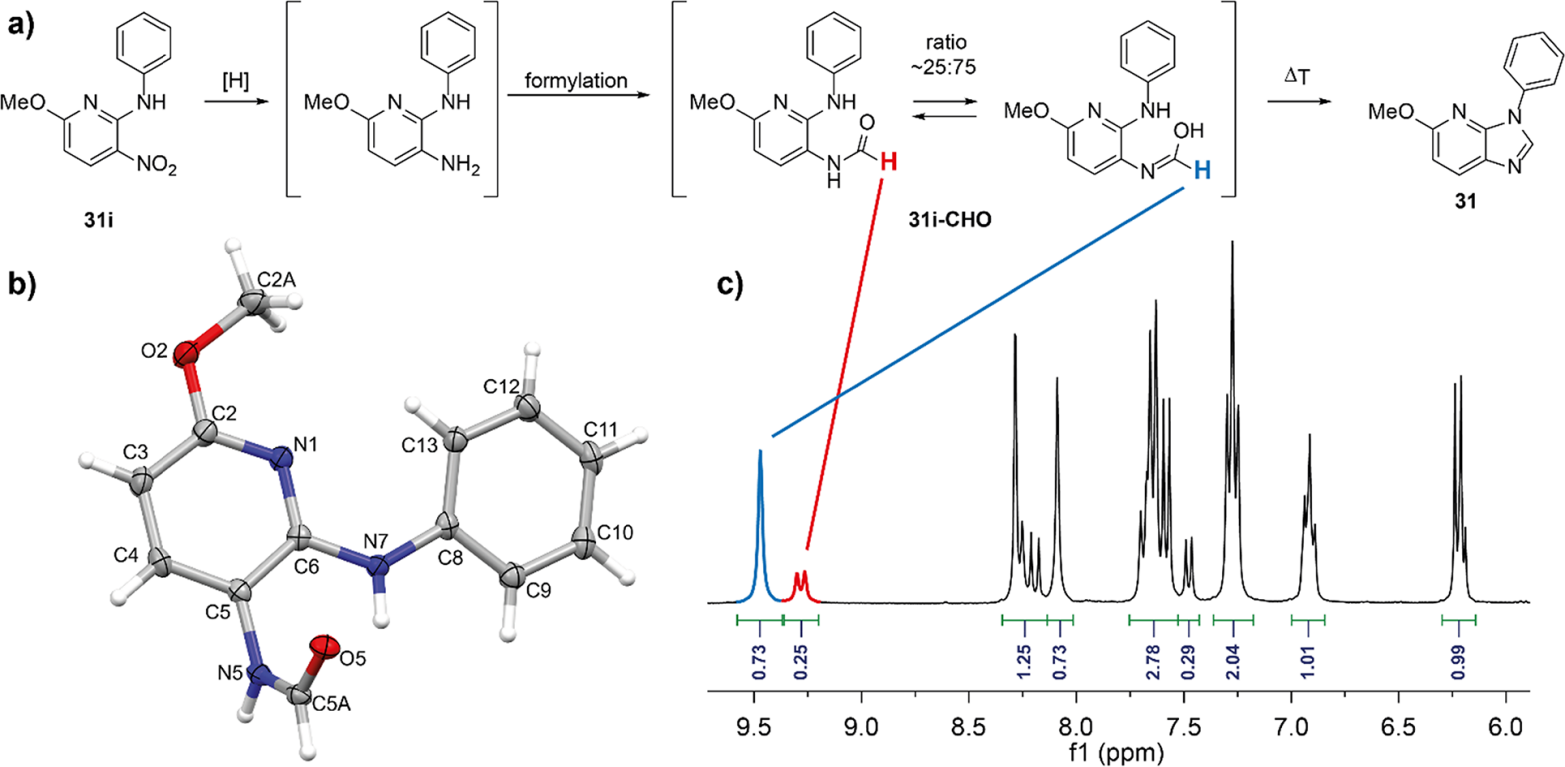
Reaction mechanism of the second stage in the presence of the Ni/NEt_3_/HCOOH system (a) and its key intermediate **31i-CHO** crystal structure (b). ^1^H NMR spectrum showing the tautomeric equilibrium (c).

Encouraged by the positive results of 11 mmol-scale synthesis, the feasibility of reaction scale-up was explored. Considering the limitation of the reaction vessel size, the target product amount was set to 3 times higher than before. However, since the same microwave vial was used, a trivial multiplication of all the reaction components’ amounts was not a good choice because of the vessel’s empty space acting as a pressure buffer. For example, heating a tripled volume of ethanol (∼6 mL) in the first stage resulted in a barely acceptable pressure level at the target reaction temperature. Meanwhile, the role of ethanol is to maintain reflux, facilitating mass circulation and preventing local overheating, so its amount does not need to be increased. The same applies to triethylamine, which reduces the volatility of formic acid and maintains an appropriate pH level. Finally, the formic acid and catalyst loading directly determine the volume of CO_2_ released in the course of the reaction, which would be excessive for a single run if the reactant content was tripled in the same vessel. Therefore, the starting amount of triethylamine and formic acid used was the same as in the small-scale reaction, while the amount of 2-chloro-3-nitropyridine and 4-trifluoromethoxyaniline was tripled. A schematic illustration of a scaled-up synthesis of **11** is presented in Scheme 5. The nucleophilic substitution proceeded smoothly with a reduced ethanol content. The second stage was performed in the same manner as in the small scale, i.e., the catalyst load and NEt_3_/HCOOH or HCOOH volumes added remained unchanged, but their equivalent values were 3 times lower due to the 3 times higher substrate content. The catalyst and HCOOH were required to be added continuously until the reaction was complete and split into several portions to prevent excessive CO_2_ pressure build-up. After each addition and heating step, the substrate conversion was determined. Surprisingly, the scaled-up process required only a 40 mol % Ni catalyst and 8.33 equiv of HCOOH to achieve the same yield, showing better efficiency compared to the small scale. To sum up, the amount of product obtained was 3 times higher, while it required only double the catalyst and HCOOH loads compared to the small-scale protocol.

**Scheme 5:**
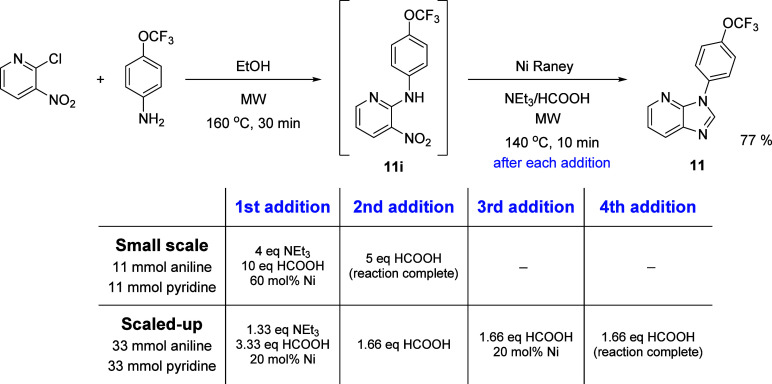
Comparison of Small-Scale and Scaled-Up Syntheses of **11**

To summarize, efficient microwave-assisted one-pot protocols for the synthesis of many functionalized pyridoimidazoles were developed. The adopted and optimized Ni-catalyzed procedure showed broad applicability, being tolerant to many functional groups attached to the aniline moiety, e.g., alkyl, alkoxy, hydroxy, halogen, acyl, and amino substituents. Another great advantage of this one-pot approach is that nucleophilic substitution, nitro group reduction, and imidazole ring formation can be done in a very short reaction time in the same vessel and without isolation of any intermediates, thus saving materials, work, and time. The desired products were obtained mostly in very good yields, keeping in mind that these were the total yields of three-step reactions. The encountered problematic thio- and nitrile-derived substrates were transformed via an alternatively developed Zn-accompanied stoichiometric three-stage pathway. Finally, the usefulness of the Ni-catalyzed procedure was confirmed in the conversion of substituted 2-chloro-3-nitropyridines, leading to the desired pyridoimidazoles functionalized at the pyridine moiety. The developed procedures allowed the acquisition of 32 pyridoimidazoles, all of which were confirmed by NMR and HRMS techniques. The synthesis of an exemplary compound was scaled up 3-fold, and the process was found to be more efficient, as lower NEt_3_, HCOOH, and catalyst equivalents were required. A detailed mechanistic study showed that the formation of the imidazole ring proceeds via a formamide derivative, which shows tautomerism, and the process can be modulated by the electron density-modulating substituent on the pyridine moiety. Twenty XRD structures were solved for nitro-derived intermediates and the target products, proving the chemical structures of the obtained compounds and providing insight into their crystal structure. The synthesized compounds can find many applications, in particular as precursors of NHC carbene ligands for efficient iridium-based phosphorescent deep blue emitters suitable for use in OLED technology. Among them, the bromine-functionalized ones seem to be promising building blocks for further transformations, successfully synthesized thanks to the selectivity of the Ni-based catalytic system.

## Supplementary Material



## Data Availability

The data underlying this study are available in the published article and its Supporting Information.

## Notes

Experimental details, synthetic procedures, NMR spectra, HRMS spectra, and crystallographic data (PDF)
