# Supplementary material for: Microwave-Assisted One-Pot Synthetic Pathways for Pyrido[2,3‑d]imidazole Derivatives
Source: J Org Chem. 2026 Feb 6;91(7):2851–8. doi: 10.1021/acs.joc.5c01864 (PMC13298884; doi:10.1021/acs.joc.5c01864)
Supplement: Supplementary file 1 [file jo5c01864_si_001.pdf]

Microwave-assisted one-pot synthetic pathways for pyrido[2,3-*d*]imidazole derivatives

Bartosz Orwat*[a,b,c], Sylwia Raczak[b,c], Kamila Jankowska[b,c], Grzegorz Krajewski[b,c], Julita Nawrocik[b,c], Maciej Kubicki[a], Rafał Januszewski[b,c], Beata Łuszczyńska[a], Ireneusz Kownacki*[b,c]

[a] Department of Molecular Physics, Faculty of Chemistry, Lodz University of Technology, Zeromskiego 116, 90-924 Lodz, Poland

[b] Faculty of Chemistry, Adam Mickiewicz University in Poznan, Uniwersytetu Poznanskiego 8, 61-614 Poznan, Poland

[c] Center for Advanced Technologies, Uniwersytetu Poznanskiego 10, 61-614 Poznan, Poland

Corresponding authors' emails: bartosz.orwat@p.lodz.pl, ireneusz.kownacki@amu.edu.pl

Supporting information

Table of contents

Experimental details *S2*

Synthetic procedures and analytical data *S3*

^{1}H and ^{13}C NMR spectra *S17*

ESI-HRMS spectra *S63*

X-ray crystallography *S79*

References *S89*

Experimental details

Ethanol (puriss p.a.) and ammonia aqueous solution were obtained from Chempur. Acetonitrile, aniline, 2,4-dimethylaniline, 4-methoxyaniline, 2,4-dimethoxyaniline, 3,4,5-trimethoxyaniline, 3-phenoxyaniline, 4-phenoxyaniline, 3-hydroxyaniline, 2,4-difluoroaniline, 3-bromoaniline, 4-bromoaniline, 3-chloro-4-fluoroaniline, 3-acetylaniline, 4-acetylaniline, triethylamine, formic acid (80 %), sodium hydroxide, aluminum/nickel (50/50) alloy were purchased from Sigma-Aldrich (Merck). 3-aminobenzonitrile and 4-aminobenzonitrile were purchased from TCI Chemicals. 2-chloro-3-nitropyridine, 3-methoxyaniline, 4-hydroxyaniline, 3-(trifluoromethoxy)aniline, 4-(trifluoromethoxy)aniline, 3-fluoroaniline, 4-fluoroaniline, 3,4-difluoroaniline, 4-(trifluoromethyl)aniline, 4-bromo-3-methylaniline, 4-bromo-3-(trifluoromethyl)aniline, 4-(dimethylamino)aniline, 4-(methylthio)aniline, 2-chloro-4-methyl-3-nitropyridine, 2-chloro-5-methyl-3-nitropyridine, 5-bromo-2-chloro-3-nitropyridine, 2-chloro-6-methoxy-3-nitropyridine were purchased from Fluorochem and distilled under reduced pressure prior to use. Deuterated solvents were purchased from Deutero GmbH and Sigma-Aldrich.

A mixture of triethylamine and 80 % aqueous formic acid was prepared by slow addition of the acid (6.19 mL) to vigorously stirred triethylamine (5.19 mL), in a 10 : 4 molar ratio. The mixture was cooled in a water bath during the addition (neutralization is exothermic). The mixture could be prepared in larger amounts and stored at room temperature.

Reactions accelerated with microwave irradiation were conducted in 10 or 80 mL capped quartz vials using CEM Discover and CEM SP-D 80, pressurized microwave reactors. The vials with reaction mixtures containing nickel catalyst were flushed with argon (99.999 % purity) prior to closing to remove most of the oxygen. The reaction progress was monitored using a gas chromatograph (Bruker 436-GC) with a thermal conductivity detector and another one (Bruker 450-GC) coupled to a mass detector (Bruker 320-MS). In some cases, thin-layer chromatography (TLC) was applied. The chemical structures of the obtained compounds were confirmed by nuclear magnetic resonance (NMR) spectroscopy using Bruker Ultrashield 300 MHz and Bruker Ascend 400 MHz Nanobay spectrometers. $CDCl_3$, CD_2Cl_2 and DMSO-d_6 were used for NMR analysis with the residual solvent peak as an internal standard (chloroform ^1H δ = 7.26 ppm, ^{13}C δ = 77.16 ppm; dichloromethane ^1H δ = 5.32 ppm, ^{13}C δ = 54.00 ppm; DMSO ^1H δ = 2.50 ppm, ^{13}C δ = 39.52 ppm). High-resolution mass spectrometry (HRMS) analysis was conducted using a time-of-flight (TOF) mass detector (Bruker Impact III HD), in the electrospray ionization (ESI) mode. ESI-HRMS spectra were recorded in positive ionization mode and the analytes were dissolved in HPLC grade ACN. Celite 545 0.02-0.1 mm (Merck) was used for filtration.

Diffraction data were collected by the ω-scan technique, using two different Rigaku four-circle diffractometers: at room temperature or 100(1) K on XCalibur with EOS CCD detector and graphite-monochromated MoK_α radiation (λ=0.71073 Å), at room temperature or 130(1) K on SuperNova with Atlas CCD detector and Nova microfocus CuK_α radiation source (λ = 1.54178 Å); or Bruker D8 QUEST KAPPA diffractometer with a microfocus sealed tube using a multilayer mirror as monochromator (CuKα radiation, λ=1.54178 Å) and a Bruker PHOTON III CPAD detector. The data were corrected for Lorentz-polarization as well as for absorption effects [1]. Precise unit-cell parameters were determined by a least-squares fit of the reflections of the highest intensity, chosen from the whole experiment. The structures were solved with SHELXT [2] and refined with the full-matrix least-squares procedure on F^2 by SHELXL [3]. All non-hydrogen atoms were refined anisotropically. In all structures with the exception of **2i** NH hydrogen atoms were freely refined, while all other hydrogen atoms were placed in idealized positions and refined as a 'riding model' with isotropic displacement parameters set at 1.2 (1.5 for CH_3) times U_{eq} of appropriate carrier atoms. The crystals of **2i** appeared to be twinned, and this was taken into account during both data reduction and structure refinement. The BASF parameter [3], describing the content of the two components, refined at 9.8(5)%. The crystals of **18** appeared to be twinned, and this was taken into account during both data reduction and structure refinement. The BASF parameter [3], describing the content of the two components, refined at 57.12(12)%. In **19**, the substantial disorder of the ethylene bridges has been found; both alternative positions were however refined without restraints; s.o.f.'s converged at 0.78(1)/0.22(1).

Crystallographic data for the structural analysis have been deposited with the Cambridge Crystallographic Data Centre. Copies of this information may be obtained free of charge from: The Director, CCDC, 12 Union Road, Cambridge, CB2 1EZ, UK; e-mail: deposit@ccdc.cam.ac.uk, or www: www.ccdc.cam.ac.uk.

Synthetic procedures and analytical data

Procedure for the Ni catalyst preparation

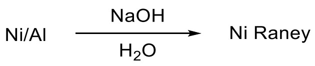

A suitable glass vial equipped with a stirring bar covering the whole bottom was loaded with a 20 % w/w NaOH aqueous solution. The vial was placed in a water bath at room temperature, on a magnetic stirrer. Ni/Al alloy powder (200 mg per 1 mL of NaOH solution) was added in a few portions to the slowly stirred solution. The vial was kept loosely capped whenever possible, to reduce the air contact with the solution. Each portion was added in 10 min intervals, to prevent the spill of the foam being formed. After the addition complete, the water bath was set to 50 ºC and the suspension was stirred till the significant gas evolution cease was noticed (usually around 1 hour). After that, the water bath was removed and the same amount of water was added. After settling down, the liquid phase was removed by decantation. The precipitate was suspended in the same amount of water, stirred for a while, allowed to settle down, and decanted. The catalyst was washed in this way 3 times with water and 3 times with ethanol in total. The catalyst was stored under the last portion of ethanol. Whenever necessary, the ethanol was decanted and the catalyst was taken up using the triethylamine/formic acid mixture for the target reaction.

Procedure for the reaction conditions optimization

Nucleophilic substitution stage

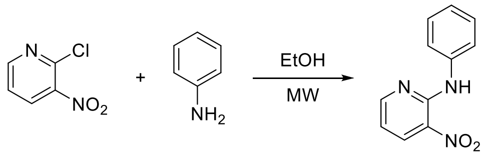

A 10 mL microwave vial was loaded with 2-chloro-3-nitropyridine (178 mg, 1.1 mmol, 1.0 eq), aniline (105 mg, 1.12 mmol, 1.02 eq), and a small amount of ethanol (192 µL, 3.30 mmol, 3.0 eq). The vial was capped and subjected to microwave irradiation (various temperatures, 30 min, P_{max} = 50 W). After cooling down, 2 mL of chloroform and 0.5 mL of ammonia aqueous solution were added. The organic phase was extracted 3 times with chloroform and evaporated to dryness. The obtained material was weighted and the product content was determined using the NMR spectroscopy.

Catalytic reduction and ring closure stage

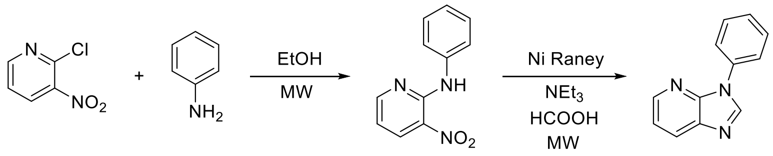

A 10 mL microwave vial was loaded with 2-chloro-3-nitropyridine (178 mg, 1.1 mmol, 1.0 eq), aniline (105 mg, 1.12 mmol, 1.02 eq), and a small amount of ethanol (192 µL, 3.30 mmol, 3.0 eq). The vial was capped and subjected to microwave irradiation (160 ºC, 30 min, P_{max} = 50 W). After cooling down, the target amount of the Ni catalyst was transferred to the reaction vial using 1.14 mL of the previously prepared triethylamine (619 µL, 4.40 mmol, 4.0 eq) and formic acid (519 µL, 11.0 mmol, 10.0 eq) mixture. The vial was purged with argon, closed, and placed again into the reactor. The reaction was subjected to microwave irradiation (various temperatures, 10 min, P_{max} = 150 W). After cooling down, an additional portion of formic acid was added (260 µL, 5.5 mmol, 5.0 eq), the vial was purged with argon, closed, and heated once again in the same conditions. After cooling down, a precise amount of internal standard was added (squalane), followed by 4 mL of chloroform and 1.7 mL of triethylamine. The mixture was intensively stirred for a few minutes and the organic phase was subjected to GC analysis. The content of the desired product was determined by employing a previously prepared calibration curve.

General procedure for the Ni-catalyzed synthesis of pyridoimidazoles

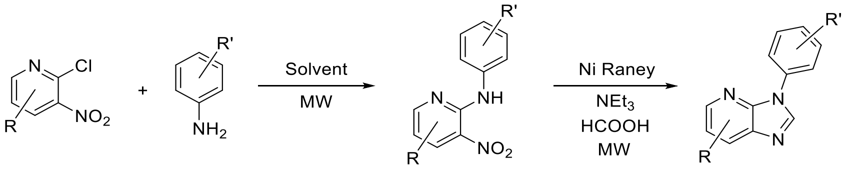

An 80 mL microwave vial was loaded with 2-chloro-3-nitropyridine derivative (11.0 mmol, 1.0 eq), aniline derivative (11.00 mmol, 1.0 eq), and a small amount of ethanol (1.92 mL, 33.0 mmol, 3.0 eq). The vial was capped and subjected to microwave irradiation (various temperatures, 30 min, P_{max} = 150 W). After cooling down, the target amount of the Ni catalyst (60 mol%) was transferred to the reaction vial using 11.4 mL of the previously prepared triethylamine (6.19 mL, 44.0 mmol, 4.0 eq) and formic acid (5.19 mL, 110.0 mmol, 10.0 eq) mixture. The vial was purged with argon, closed, and placed into the reactor. The reaction was subjected to microwave irradiation (various temperatures, 10 min, P_{max} = 100 W). After cooling down, extra formic acid was added (2.6 mL, 55.0 mmol, 5.0 eq), the vial was purged with argon, closed, and heated once again in the same conditions. After cooling down, the post-reaction mixture was transferred to a flask and 20 mL of water and 20 mL of chloroform (or ethyl

acetate in some cases) were added. The mixture was basified with ammonia aqueous solution under vigorous stirring to reach basic pH of the aqueous phase. The mixture was filtered through a celite plug. The organic phase was separated and washed 3 times with water. The extract was dried with anhydrous sodium sulfate, filtered, and concentrated. The crude product was purified via a *trap-to-trap* distillation (discarding more volatile fractions) or hydrochloride precipitation method (a crude product was dissolved in up to 10 mL of THF. Under vigorous stirring, muriatic acid was added dropwise until all the product precipitated in the hydrochloride form. The suspension was filtered, and the precipitate was washed 3 times with THF, 3 times with hexanes, and dried. The obtained hydrochloride was added to a water/dichloromethane mixture, and followed by sodium hydroxide aqueous solution until the basic pH of the aqueous phase was reached. The organic phase was separated and washed 3 times with water. The extract was dried with anhydrous sodium sulfate, filtered, and concentrated to dryness). When necessary, a recrystallization from hot 2-propanol was performed. If the product was not recovered fully, a slight amount of hexanes was added to facilitate crystallization.

General procedure for the alternative Zn-involved synthesis of pyridoimidazoles

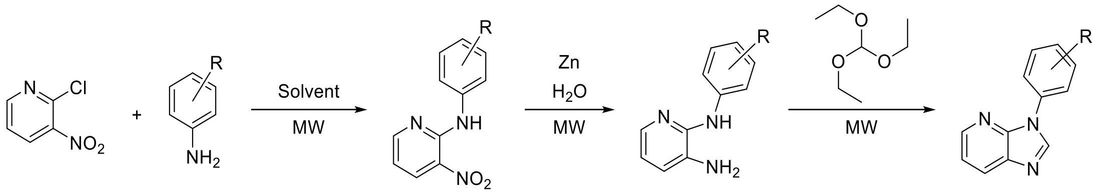

An 80 mL microwave vial was loaded with 2-chloro-3-nitropyridine (1.78 g, 11.0 mmol, 1.0 eq), an aniline derivative (11.55 mmol, 1.05 eq), and a suitable solvent. The vial was capped and subjected to microwave irradiation for the indicated time (160 °C, P_{max} = 150 W). After cooling down, zinc powder (3.6 g, 55.0 mmol, 5.0 eq), water (1.98 mL, 110 mmol, 10.0 eq), and 20 mL of ethanol were added. The mixture was stirred well, the vessel was briefly purged with argon and capped. The vial was placed in the reactor and heated (120 °C, 20 min, P_{max} = 100 W). After cooling down, triethyl orthoformate (12.8 mL, 77.0 mmol, 7.0 eq) was added and the mixture was heated in the reactor for the last time (120 °C, 20 min, P_{max} = 100 W). After cooling down, the post-reaction mixture was transferred to a flask, and all the volatiles were evaporated using a rotavapor. Then, 20 mL of water and 20 mL of chloroform were added, followed by ammonia aqueous solution with vigorous stirring to reach basic pH. The mixture was filtered through a celite plug. The organic phase was separated and washed 3 times with water basified with a slight ammonia additive. The extract was dried with anhydrous sodium sulfate, filtered, and concentrated. The crude product was purified via a *trap-to-trap* distillation or the hydrochloride precipitation method described above.

3-phenyl-3H-imidazo[4,5-b]pyridine (1)

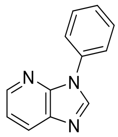

1

The compound was obtained via the Ni-catalyzed process. The first stage of the synthesis was carried out at 160 °C, and the second one at 140 °C. The compound was purified by means of *trap-to-trap* distillation under reduced pressure.

White crystalline solid (1.76 g, 9.02 mmol). Yield: 82 %.

^{1}H NMR (300 MHz, $CDCl_3$) δ 8.46 (dd, J = 4.8, 1.5 Hz, 1H), 8.34 (s, 1H), 8.15 (dd, J = 8.1, 1.5 Hz, 1H), 7.79 – 7.72 (m, 2H), 7.62 – 7.53 (m, 2H), 7.48 – 7.41 (m, 1H), 7.31 (dd, J = 8.1, 4.8 Hz, 1H).

$^{13}C\{^{1}H\}$ NMR (75 MHz, $CDCl_3$) δ 146.9, 145.1, 143.2, 136.0, 135.2, 123.0, 128.5, 128.1, 123.8, 119.1.

MS (ESI-HRMS): calculated for $[C_{12}H_{10}N_3]^+$ 196.0869, measured 196.0871 (error 1.0 ppm).

3-(2,4-dimethylphenyl)-3H-imidazo[4,5-b]pyridine (2)

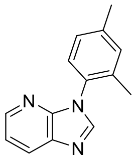

2

The compound was obtained via the Ni-catalyzed process. The first stage of the synthesis was carried out at 160 °C, and the second one at 140 °C. The compound was purified by means of *trap-to-trap* distillation under reduced pressure.

Brownish crystalline solid (1.90 g, 8.51 mmol). Yield: 77 %.

^{1}H NMR (300 MHz, $CDCl_3$) δ 8.40 (dd, J = 4.8, 1.5 Hz, 1H), 8.15 (dd, J = 8.1, 1.5 Hz, 1H), 8.10 (s, 1H), 7.28 (dd, J = 8.0, 4.8 Hz, 1H), 7.24 – 7.19 (m, 2H), 7.19 – 7.14 (m, 1H), 2.40 (s, 3H), 2.09 (s, 3H).

^{13}C{^{1}H} NMR (101 MHz, $CDCl_3$) δ 147.9, 145.2, 144.4, 139.8, 135.3, 135.2, 132.3, 131.1, 128.3, 127.9, 127.7, 118.6, 21.3, 18.0.

MS (ESI-HRMS): calculated for $[C_{14}H_{14}N_3]^+$ 224.1182, measured 224.1181 (error 0.4 ppm).

3-(4-methoxyphenyl)-3H-imidazo[4,5-b]pyridine (3)

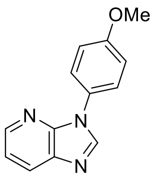

3

The compound was obtained via the Ni-catalyzed process. The first stage of the synthesis was carried out at 160 ºC, and the second one at 140 ºC. The compound was purified employing the hydrochloride precipitation method.

Brownish crystalline solid (1.93 g, 8.58 mmol). Yield: 78 %.

^{1}H NMR (400 MHz, $CDCl_3$) δ 8.44 (dd, J = 4.8, 1.5 Hz, 1H), 8.26 (s, 1H), 8.14 (dd, J = 8.1, 1.5 Hz, 1H), 7.63 – 7.58 (m, 2H), 7.29 (dd, J = 8.1, 4.8 Hz, 1H), 7.10 – 7.04 (m, 2H), 3.86 (s, 3H).

^{13}C{^{1}H} NMR (101 MHz, $CDCl_3$) δ 159.4, 147.2, 145.1, 143.5, 135.8, 128.4, 128.1, 125.5, 118.9, 115.1, 55.8.

MS (ESI-HRMS): calculated for $[C_{13}H_{12}N_3O]^+$ 226.0975, measured 226.0977 (error 0.9 ppm).

3-(3-methoxyphenyl)-3H-imidazo[4,5-b]pyridine (4)

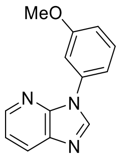

4

The compound was obtained via the Ni-catalyzed process. The first stage of the synthesis was carried out at 160 ºC, and the second one at 140 ºC. The compound was purified by means of *trap-to-trap* distillation under reduced pressure followed by recrystallization from 2-propanol/hexanes.

White powder (1.68 g, 7.48 mmol). Yield: 68 %.

^{1}H NMR (300 MHz, $CDCl_3$) δ 8.46 (dd, J = 4.8, 1.5 Hz, 1H), 8.34 (s, 1H), 8.14 (dd, J = 8.1, 1.6 Hz, 1H), 7.51 – 7.41 (m, 1H), 7.36 – 7.27 (m, 3H), 7.02 – 6.92 (m, 1H), 3.88 (s, 3H).

^{13}C{^{1}H} NMR (101 MHz, $CDCl_3$) δ 160.7, 146.9, 145.2, 143.2, 136.3, 135.9, 130.7, 128.4, 119.1, 115.8, 113.6, 109.9, 55.7.

MS (ESI-HRMS): calculated for $[C_{13}H_{12}N_3O]^+$ 226.0975, measured 226.0980 (error 2.2 ppm).

3-(2,4-dimethoxyphenyl)-3H-imidazo[4,5-b]pyridine (5)

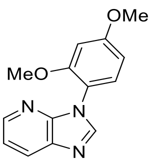

5

The compound was obtained via the Ni-catalyzed process. The first stage of the synthesis was carried out at 160 ºC, and the second one at 140 ºC. The compound was purified by means of *trap-to-trap* distillation under reduced pressure followed by recrystallization from 2-propanol/hexanes.

Grey powder (2.24 g, 8.76 mmol). Yield: 80 %.

^{1}H NMR (300 MHz, CD_2Cl_2) δ 8.34 (dd, *J* = 4.8, 1.5 Hz, 1H), 8.17 (s, 1H), 8.08 (dd, *J* = 8.0, 1.5 Hz, 1H), 7.45 (dd, *J* = 8.4, 0.6 Hz, 1H), 7.25 (dd, *J* = 8.0, 4.8 Hz, 1H), 6.70 – 6.63 (m, 2H), 3.88 (s, 3H), 3.77 (s, 3H).

^{13}C{^{1}H} NMR (75 MHz, $CDCl_3$) δ 161.2, 155.2, 147.8, 145.7, 144.8, 135.1, 128.8, 128.0, 118.5, 116.5, 104.8, 100.0, 55.9, 55.9, 55.8, 55.8.

MS (ESI-HRMS): calculated for $[C_{14}H_{14}N_3O_2]^+$ 256.1083, measured 256.1081 (error 0.8 ppm).

3-(3,4,5-trimethoxyphenyl)-3H-imidazo[4,5-b]pyridine (6)

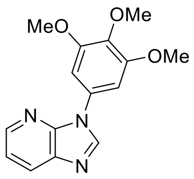

6

The compound was obtained via the Ni-catalyzed process. The first stage of the synthesis was carried out at 120 °C for 60 minutes, and the second one at 140 °C. The compound was purified by means of *trap-to-trap* distillation under reduced pressure followed by recrystallization from 2-propanol/hexanes.

White powder (2.54 g, 8.91 mmol). Yield: 81 %.

^{1}H NMR (300 MHz, $CDCl_3$) δ 8.45 (dd, *J* = 4.8, 1.5 Hz, 1H), 8.29 (s, 1H), 8.14 (dd, *J* = 8.1, 1.4 Hz, 1H), 7.30 (dd, *J* = 8.1, 4.8 Hz, 1H), 6.91 (s, 2H), 3.91 (s, 6H), 3.88 (s, 3H).

^{13}C{^{1}H} NMR (75 MHz, $CDCl_3$) δ 154.1, 147.1, 145.2, 143.4, 138.0, 135.8, 130.8, 128.5, 119.1, 102.0, 61.1, 56.5.

MS (ESI-HRMS): calculated for $[C_{15}H_{15}N_3NaO_3]^+$ 308.1006, measured 308.1007 (error 0.3 ppm).

3-(4-phenoxyphenyl)-3H-imidazo[4,5-b]pyridine (7)

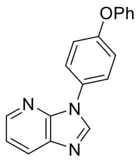

7

The compound was obtained via the Ni-catalyzed process. The first stage of the synthesis was carried out at 160 °C, and the second one at 140 °C. The compound was purified employing the hydrochloride precipitation method.

Purplish crystalline solid (2.24 g, 7.81 mmol). Yield: 71 %.

^{1}H NMR (400 MHz, $CDCl_3$) δ 8.46 (dd, *J* = 4.8, 1.6 Hz, 1H), 8.29 (s, 1H), 8.16 (dd, *J* = 8.1, 1.5 Hz, 1H), 7.70 – 7.65 (m, 2H), 7.41 – 7.35 (m, 2H), 7.31 (dd, *J* = 8.1, 4.8 Hz, 1H), 7.21 – 7.13 (m, 3H), 7.11 – 7.06 (m, 2H).

^{13}C{^{1}H} NMR (101 MHz, $CDCl_3$) δ 157.3, 156.7, 147.1, 145.1, 143.3, 136.0, 130.2, 130.1, 128.6, 125.5, 124.1, 119.7, 119.5, 119.0.

MS (ESI-HRMS): calculated for $[C_{18}H_{14}N_3O]^+$ 288.1131, measured 288.1139 (error 2.8 ppm).

3-(3-phenoxyphenyl)-3H-imidazo[4,5-b]pyridine (8)

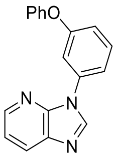

8

The compound was obtained via the Ni-catalyzed process. The first stage of the synthesis was carried out at 160 °C, and the second one at 140 °C. The compound was purified employing the hydrochloride precipitation method.

Brown viscous liquid (2.37 g, 8.25 mmol). Yield: 75 %.

^{1}H NMR (300 MHz, $CDCl_3$) δ 8.45 (dd, J = 4.8, 1.5 Hz, 1H), 8.31 (s, 1H), 8.13 (dd, J = 8.1, 1.5 Hz, 1H), 7.55 – 7.42 (m, 3H), 7.41 – 7.33 (m, 2H), 7.30 (dd, J = 8.1, 4.8 Hz, 1H), 7.18 – 7.07 (m, 3H), 7.03 (dt, J = 6.8, 2.3 Hz, 1H).

^{13}C{^{1}H} NMR (101 MHz, $CDCl_3$) δ 158.8, 156.3, 146.8, 145.1, 143.0, 136.5, 136.1, 130.9, 130.1, 128.5, 124.2, 119.6, 119.1, 117.9, 117.5, 113.8.

MS (ESI-HRMS): calculated for $[C_{18}H_{14}N_3O]^+$ 288.1131, measured 288,1136 (error 1.7 ppm).

4-(3H-imidazo[4,5-b]pyridin-3-yl)phenol (9)

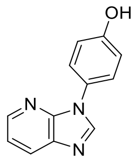

9

The compound was obtained via the Ni-catalyzed process. The first stage of the synthesis was carried out at 120 ºC for 60 minutes, and the second one at 140 ºC. The compound was purified by means of *trap-to-trap* distillation under reduced pressure followed by recrystallization from 2-propanol/hexanes.

Red powder (1.34 g, 6.38 mmol). Yield: 58 %.

^{1}H NMR (300 MHz, DMSO-d_6) δ 9.81 (s, 1H), 8.73 (s, 1H), 8.39 (dd, J = 4.7, 1.6 Hz, 1H), 8.17 (dd, J = 8.1, 1.5 Hz, 1H), 7.68 – 7.60 (m, 2H), 7.35 (dd, J = 8.1, 4.7 Hz, 1H), 7.00 – 6.92 (m, 2H).

^{13}C{^{1}H} NMR (101 MHz, DMSO-d_6) δ 156.9, 146.6, 144.4, 144.2, 135.2, 127.7, 126.6, 125.1, 118.5, 115.8.

MS (ESI-HRMS): calculated for $[C_{12}H_{10}N_3O]^+$ 212.0818, measured 212.0818 (error 0.0 ppm).

3-(3H-imidazo[4,5-b]pyridin-3-yl)phenol (10)

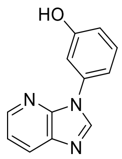

10

The compound was obtained via the Ni-catalyzed process. The first stage of the synthesis was carried out at 160 ºC, and the second one at 140 ºC. The compound was purified by means of *trap-to-trap* distillation under reduced pressure followed by recrystallization from 2-propanol/hexanes.

White fluffy solid (1.32 g, 6.27 mmol). Yield: 57 %.

^{1}H NMR (300 MHz, DMSO-d_6) δ 9.93 (s, 1H), 8.86 (s, 1H), 8.43 (dd, J = 4.8, 1.5 Hz, 1H), 8.20 (dd, J = 8.1, 1.5 Hz, 1H), 7.46 – 7.27 (m, 4H), 6.91 – 6.81 (m, 1H).

^{13}C{^{1}H} NMR (101 MHz, DMSO-d_6) δ 158.2, 146.4, 144.4, 144.2, 136.2, 135.6, 130.3, 128.0, 118.9, 114.4, 113.5, 110.2.

MS (ESI-HRMS): calculated for $[C_{12}H_{10}N_3O]^+$ 212.0818, measured 212.0819 (error 0.5 ppm).

3-(4-(trifluoromethoxy)phenyl)-3H-imidazo[4,5-b]pyridine (11)

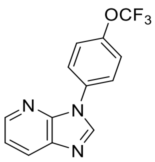

11

The compound was obtained via the Ni-catalyzed process. The first stage of the synthesis was carried out at 160 ºC, and the second one at 140 ºC. The compound was purified by means of *trap-to-trap* distillation under reduced pressure followed by recrystallization from 2-propanol/hexanes.

Grey powder (2.36 g, 8.47 mmol). Yield: 77 %.

^{1}H NMR (401 MHz, cdcl$_3$) δ 8.46 (dd, *J* = 4.8, 1.5 Hz, 1H), 8.34 (s, 1H), 8.17 (dd, *J* = 8.1, 1.5 Hz, 1H), 7.85 – 7.80 (m, 2H), 7.46 – 7.41 (m, 2H), 7.34 (dd, *J* = 8.1, 4.8 Hz, 1H).

^{13}C{^{1}H} NMR (101 MHz, CDCl$_3$) δ 148.5, 148.5, 146.8, 145.3, 142.8, 136.0, 133.8, 128.7, 125.0, 122.6, 121.8, 119.4, 119.3.

^{19}F NMR (376 MHz, CDCl$_3$) δ -57.94.

MS (ESI-HRMS): calculated for $[C_{13}H_9F_3N_3O]^+$ 280.0692, measured 280.0694 (error 0.7 ppm).

3-(3-(trifluoromethoxy)phenyl)-3H-imidazo[4,5-b]pyridine (12)

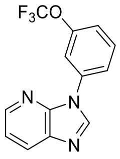

12

The compound was obtained via the Ni-catalyzed process. The first stage of the synthesis was carried out at 160 ºC, and the second one at 140 ºC. The compound was purified by means of *trap-to-trap* distillation under reduced pressure followed by recrystallization from 2-propanol/hexanes.

Brownish powder (2.45 g, 8.80 mmol). Yield: 80 %.

^{1}H NMR (300 MHz, CDCl$_3$) δ 8.48 (dd, *J* = 4.8, 1.5 Hz, 1H), 8.37 (s, 1H), 8.16 (dd, *J* = 8.1, 1.6 Hz, 1H), 7.80 – 7.73 (m, 2H), 7.65 – 7.56 (m, 1H), 7.38 – 7.27 (m, 2H).

^{13}C{^{1}H} NMR (101 MHz, CDCl$_3$) δ 150.1, 150.1, 150.1, 150.1, 146.7, 145.4, 142.6, 136.6, 136.1, 131.1, 128.7, 124.4, 121.8, 121.4, 120.0, 119.5, 119.3, 116.7, 116.3.

^{19}F NMR (376 MHz, CDCl$_3$) δ -57.78.

MS (ESI-HRMS): calculated for $[C_{13}H_9F_3N_3O]^+$ 280.0692, measured 280.0697 (error 1.8 ppm).

3-(4-fluorophenyl)-3H-imidazo[4,5-b]pyridine (13)

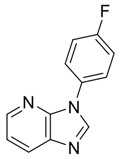

13

The compound was obtained via the Ni-catalyzed process. The first stage of the synthesis was carried out at 160 ºC, and the second one at 140 ºC. The compound was purified employing the hydrochloride precipitation method.

Grey fluffy solid (1.80 g, 8.47 mmol). Yield: 77 %.

^{1}H NMR (300 MHz, CD_2Cl_2) δ 8.42 (dd, *J* = 4.8, 1.5 Hz, 1H), 8.30 (s, 1H), 8.13 (dd, *J* = 8.0, 1.5 Hz, 1H), 7.81 – 7.73 (m, 2H), 7.35 – 7.24 (m, 3H).

^{13}C{^{1}H} NMR (101 MHz, CDCl$_3$) δ 163.3, 160.8, 147.0, 145.2, 143.1, 136.0, 131.4, 131.3, 128.6, 125.7, 125.7, 119.2, 117.0, 116.8.

^{19}F NMR (376 MHz, CDCl$_3$) δ -113.14 – -113.23 (m).

MS (ESI-HRMS): calculated for $[C_{12}H_9FN_3]^+$ 214.0779, measured 214.0777 (error 0.9 ppm).

3-(3-fluorophenyl)-3H-imidazo[4,5-b]pyridine (14)

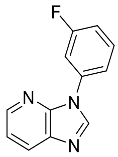

14

The compound was obtained via the Ni-catalyzed process. The first stage of the synthesis was carried out at 160 °C, and the second one at 140 °C. The compound was purified employing the hydrochloride precipitation method.

Grey crystalline solid (1.83 g, 8.58 mmol). Yield: 78 %.

^{1}H NMR (400 MHz, $CDCl_3$) δ 8.47 (dd, J = 4.8, 1.6 Hz, 1H), 8.35 (s, 1H), 8.15 (dd, J = 8.1, 1.5 Hz, 1H), 7.64 (dt, J = 9.5, 2.3 Hz, 1H), 7.60 – 7.50 (m, 2H), 7.33 (dd, J = 8.1, 4.8 Hz, 1H), 7.14 (tdd, J = 8.2, 2.4, 1.3 Hz, 1H).

^{13}C{^{1}H} NMR (101 MHz, $CDCl_3$) δ 164.5, 162.0, 146.7, 145.2, 142.7, 136.7, 136.6, 136.2, 131.3, 131.2, 128.7, 119.4, 118.8, 118.8, 115.0, 114.8, 111.2, 111.0.

^{19}F NMR (376 MHz, $CDCl_3$) δ -109.74 – -109.82 (m).

MS (ESI-HRMS): calculated for $[C_{12}H_9FN_3]^+$ 214.0779, measured 214.0775 (error 1.9 ppm).

3-(2,4-difluorophenyl)-3H-imidazo[4,5-b]pyridine (15)

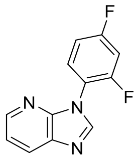

15

The compound was obtained via the Ni-catalyzed process. The first stage of the synthesis was carried out at 160 °C, and the second one at 140 °C. The compound was purified employing the hydrochloride precipitation method.

Grey fluffy solid (1.78 g, 7.70 mmol). Yield: 70 %.

^{1}H NMR (400 MHz, $CDCl_3$) δ 8.43 (dd, J = 4.8, 1.5 Hz, 1H), 8.24 (d, J = 2.3 Hz, 1H), 8.17 (dd, J = 8.1, 1.6 Hz, 1H), 7.80 – 7.72 (m, 1H), 7.33 (dd, J = 8.1, 4.8 Hz, 1H), 7.15 – 7.07 (m, 2H).

^{13}C{^{1}H} NMR (101 MHz, $CDCl_3$) δ 163.8, 163.6, 161.2, 161.1, 158.1, 158.0, 155.5, 155.4, 147.2, 145.3, 144.0, 144.0, 135.3, 129.0, 129.0, 128.7, 119.3, 119.3, 119.3, 119.2, 119.1, 112.6, 112.6, 112.4, 112.4, 106.0, 105.7, 105.7, 105.5.

^{19}F NMR (376 MHz, $CDCl_3$) δ -107.78 – -107.87 (m), -117.83 – -117.93 (m).

MS (ESI-HRMS): calculated for $[C_{12}H_8F_2N_3]^+$ 232.0681, measured 232.0685 (error 1.7 ppm).

3-(3,4-difluorophenyl)-3H-imidazo[4,5-b]pyridine (16)

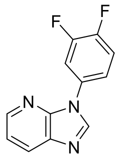

16

The compound was obtained via the Ni-catalyzed process. The first stage of the synthesis was carried out at 160 °C, and the second one at 140 °C. The compound was purified employing the hydrochloride precipitation method.

Grey fluffy solid (2.08 g, 9.02 mmol). Yield: 82 %.

^{1}H NMR (400 MHz, $CDCl_3$) δ 8.46 (dd, J = 4.8, 1.5 Hz, 1H), 8.30 (s, 1H), 8.16 (dd, J = 8.1, 1.5 Hz, 1H), 7.75 (ddd, J = 10.8, 6.9, 2.6 Hz, 1H), 7.54 – 7.48 (m, 1H), 7.41 – 7.31 (m, 2H).

^{13}C{^{1}H} NMR (101 MHz, $CDCl_3$) δ 152.0, 151.9, 151.1, 151.0, 149.5, 149.4, 148.7, 148.5, 146.7, 145.3, 142.6, 136.0, 131.7, 131.6, 128.8, 119.5, 119.5, 119.5, 119.4, 118.5, 118.5, 118.4, 118.3, 113.6, 113.4.

^{19}F NMR (376 MHz, $CDCl_3$) δ -133.51 – -133.66 (m), -137.53 – -137.67 (m).

MS (ESI-HRMS): calculated for $[C_{12}H_8F_2N_3]^+$ 232.0681, measured 232.0685 (error 1.7 ppm).

Synthetic procedures and analytical data

3-(3-chloro-4-fluorophenyl)-3H-imidazo[4,5-b]pyridine (17)

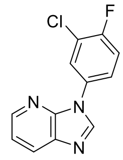

17

The compound was obtained via the Ni-catalyzed process. The first stage of the synthesis was carried out at 160 ºC, and the second one at 140 ºC. The compound was purified employing the hydrochloride precipitation method followed by *trap-to-trap* distillation under reduced pressure.

White fluffy solid (2.25 g, 9.13 mmol). Yield: 83 %.

^{1}H NMR (300 MHz, $CDCl_3$) δ 8.46 (dd, *J* = 4.7, 1.5 Hz, 1H), 8.29 (s, 1H), 8.15 (dd, *J* = 8.1, 1.6 Hz, 1H), 7.89 (dd, *J* = 6.3, 2.6 Hz, 1H), 7.70 – 7.63 (m, 1H), 7.38 – 7.29 (m, 2H).

^{13}C{^{1}H} NMR (75 MHz, $CDCl_3$) δ 159.2, 155.9, 146.7, 145.4, 142.6, 136.0, 131.9, 131.8, 128.8, 126.1, 126.0, 123.5, 123.4, 123.4, 123.3, 123.3, 122.6, 122.4, 119.4, 117.9, 117.6.

^{19}F NMR (376 MHz, $CDCl_3$) δ -115.46 – -115.53 (m).

MS (ESI-HRMS): calculated for $[C_{12}H_8ClFN_3]^+$ 248.0385, measured 248.0389 (error 1.6 ppm).

3-(4-(trifluoromethyl)phenyl)-3H-imidazo[4,5-b]pyridine (18)

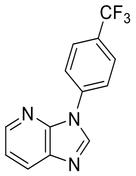

18

The compound was obtained via the Ni-catalyzed process. The first stage of the synthesis was carried out at 130 ºC for 60 minutes, and the second one at 140 ºC. The compound was purified by means of *trap-to-trap* distillation under reduced pressure.

Brownish crystalline solid (2.11 g, 8.03 mmol). Yield: 73 %.

^{1}H NMR (300 MHz, $CDCl_3$) δ 8.51 – 8.46 (m, 2H), 8.19 (dd, *J* = 8.1, 1.5 Hz, 1H), 8.03 – 7.96 (m, 2H), 7.88 – 7.81 (m, 2H), 7.36 (dd, *J* = 8.1, 4.8 Hz, 1H).

^{13}C{^{1}H} NMR (75 MHz, $CDCl_3$) δ 146.5, 145.5, 142.4, 138.2, 135.8, 130.6, 130.2, 129.7, 129.3, 129.2, 128.7, 125.6, 123.4, 122.0, 119.7, 118.4.

^{19}F NMR (283 MHz, $CDCl_3$) δ -63.08.

MS (ESI-HRMS): calculated for $[C_{13}H_9F_3N_3]^+$ 264.0743, measured 264.0738 (error 1.9 ppm).

3-(4-bromophenyl)-3H-imidazo[4,5-b]pyridine (19)

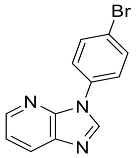

19

The compound was obtained via the Ni-catalyzed process. The first stage of the synthesis was carried out at 160 ºC, and the second one at 140 ºC. The compound was purified by means of *trap-to-trap* distillation under reduced pressure.

White powder (2.11 g, 7.70 mmol). Yield: 70 %.

^{1}H NMR (300 MHz, $CDCl_3$) δ 8.45 (d, *J* = 5.9 Hz, 1H), 8.32 (s, 1H), 8.15 (d, *J* = 8.0 Hz, 1H), 7.68 (s, 4H), 7.32 (dd, *J* = 8.1, 4.8 Hz, 1H).

$^{13}C\{^1H\}$ NMR (75 MHz, $CDCl_3$) δ 146.7, 145.2, 142.7, 142.6, 136.1, 134.3, 133.1, 128.7, 125.1, 121.6, 119.3.

MS (ESI-HRMS): calculated for $[C_{12}H_9BrN_3]^+$ 273.9974, measured 273.9977 (error 1.1 ppm).

3-(3-bromophenyl)-3H-imidazo[4,5-b]pyridine (20)

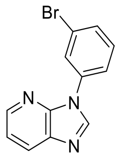

20

The compound was obtained via the Ni-catalyzed process. The first stage of the synthesis was carried out at 160 °C, and the second one at 140 °C. The compound was purified by means of *trap-to-trap* distillation under reduced pressure.

Yellow needles (2.29 g, 8.36 mmol). Yield: 76 %.

^1H NMR (300 MHz, $CDCl_3$) δ 8.47 (dd, J = 4.8, 1.5 Hz, 1H), 8.34 (s, 1H), 8.15 (dd, J = 8.1, 1.5 Hz, 1H), 7.99 (t, J = 2.0 Hz, 1H), 7.78 – 7.71 (m, 1H), 7.60 – 7.54 (m, 1H), 7.48 – 7.40 (m, 1H), 7.33 (dd, J = 8.1, 4.8 Hz, 1H).

$^{13}C\{^1H\}$ NMR (101 MHz, $CDCl_3$) δ 146.7, 145.3, 142.7, 136.5, 136.0, 131.2, 131.0, 128.7, 126.6, 123.4, 122.0, 119.4.

MS (ESI-HRMS): calculated for $[C_{12}H_9BrN_3]^+$ 273.9974, measured 273.9975 (error 0.4 ppm).

3-(4-bromo-3-methylphenyl)-3H-imidazo[4,5-b]pyridine (21)

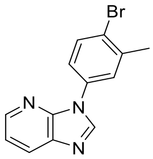

21

The compound was obtained via the Ni-catalyzed process. The first stage of the synthesis was carried out at 160 °C, and the second one at 140 °C. The compound was purified employing the hydrochloride precipitation method followed by *trap-to-trap* distillation under reduced pressure.

White powder (2.44 g, 8.47 mmol). Yield: 77 %.

^1H NMR (300 MHz, $CDCl_3$) δ 8.44 (dd, J = 4.8, 1.5 Hz, 1H), 8.29 (s, 1H), 8.14 (dd, J = 8.1, 1.5 Hz, 1H), 7.70 (d, J = 8.5 Hz, 1H), 7.65 (dd, J = 2.6, 0.8 Hz, 1H), 7.48 – 7.42 (m, 1H), 7.30 (dd, J = 8.1, 4.8 Hz, 1H), 2.49 (d, J = 0.7 Hz, 3H).

$^{13}C\{^1H\}$ NMR (75 MHz, $CDCl_3$) δ 146.8, 145.2, 142.8, 139.9, 136.0, 134.4, 133.7, 128.6, 125.8, 124.2, 122.5, 119.2, 23.3.

MS (ESI-HRMS): calculated for $[C_{13}H_{10}BrN_3Na]^+$ 309.9950, measured 309.9952 (error 0.6 ppm).

3-(4-bromo-3-(trifluoromethyl)phenyl)-3H-imidazo[4,5-b]pyridine (22)

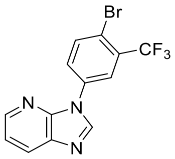

22

The compound was obtained via the Ni-catalyzed process. The first stage of the synthesis was carried out at 160 °C, and the second one at 140 °C. The compound was purified by means of *trap-to-trap* distillation under reduced pressure followed by recrystallization from 2-propanol/hexanes.

White fluffy solid (2.59 g, 7.59 mmol). Yield: 69 %.

^1H NMR (401 MHz, $CDCl_3$) δ 8.48 (dd, J = 4.8, 1.5 Hz, 1H), 8.41 (s, 1H), 8.21 – 8.16 (m, 2H), 7.92 (d, J = 1.5 Hz, 2H), 7.36 (dd, J = 8.1, 4.8 Hz, 1H).

$^{13}C\{^1H\}$ NMR (101 MHz, $CDCl_3$) δ 146.5, 145.6, 142.1, 136.6, 135.9, 134.7, 132.4, 132.1, 131.8, 131.5, 128.9, 127.4, 126.6, 123.9, 122.8, 122.7, 122.7, 122.6, 121.1, 119.8, 119.0, 118.9, 118.4.

^{19}F NMR (283 MHz, $CDCl_3$) δ -63.47.

MS (ESI-HRMS): calculated for $[C_{13}H_8BrF_3N_3]^+$ 341.9848, measured 341.9851 (error 0.9 ppm).

1-(4-(3H-imidazo[4,5-b]pyridin-3-yl)phenyl)ethan-1-one (23)

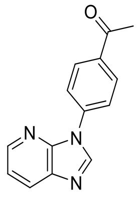

23

The compound was obtained via the Ni-catalyzed process. The first stage of the synthesis was carried out at 160 °C, and the second one at 140 °C. The compound was purified by means of *trap-to-trap* distillation under reduced pressure followed by recrystallization from 2-propanol/hexanes.

White fluffy solid (2.59 g, 9.02 mmol). Yield: 82 %.

^{1}H NMR (401 MHz, $CDCl_3$) δ 8.50 – 8.46 (m, 2H), 8.19 – 8.14 (m, 3H), 8.00 – 7.96 (m, 2H), 7.35 (dd, J = 8.1, 4.8 Hz, 1H), 2.65 (s, 3H).

^{13}C{^{1}H} NMR (101 MHz, $CDCl_3$) δ 196.8, 146.6, 145.4, 142.4, 139.1, 136.1, 136.0, 130.2, 128.7, 122.8, 119.6, 26.8.

MS (ESI-HRMS): calculated for $[C_{14}H_{12}N_3O]^+$ 238.0975, measured 238.0982 (error 3.0 ppm).

1-(3-(3H-imidazo[4,5-b]pyridin-3-yl)phenyl)ethan-1-one (24)

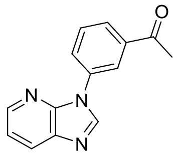

24

The compound was obtained via the Ni-catalyzed process. The first stage of the synthesis was carried out at 160 °C, and the second one at 140 °C. The compound was purified by means of *trap-to-trap* distillation under reduced pressure followed by recrystallization from 2-propanol/hexanes.

White fluffy solid (1.96 g, 8.25 mmol). Yield: 75 %.

^{1}H NMR (400 MHz, $CDCl_3$) δ 8.46 (dd, J = 4.8, 1.5 Hz, 1H), 8.39 (s, 1H), 8.35 (t, J = 2.0 Hz, 1H), 8.16 (dd, J = 8.1, 1.5 Hz, 1H), 8.05 (ddd, J = 7.9, 2.3, 1.1 Hz, 1H), 8.01 (dt, J = 7.8, 1.2 Hz, 1H), 7.69 (t, J = 7.9 Hz, 1H), 7.33 (dd, J = 8.1, 4.8 Hz, 1H), 2.67 (s, 3H).

^{13}C{^{1}H} NMR (101 MHz, $CDCl_3$) δ 197.0, 146.8, 145.3, 142.8, 138.7, 136.1, 135.9, 130.3, 128.7, 127.9, 127.7, 123.0, 119.3, 26.9.

MS (ESI-HRMS): calculated for $[C_{14}H_{12}N_3O]^+$ 238.0975, measured 238.0985 (error 4.2 ppm).

4-(3H-imidazo[4,5-b]pyridin-3-yl)-N,N-dimethylaniline (25)

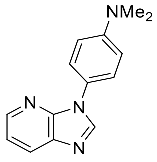

25

The compound was obtained via the Ni-catalyzed process. The first stage of the synthesis was carried out at 120 °C, and the second one at 140 °C, the second heating run (after the addition of the formic acid) was extended to 20 minutes. The compound was purified by means of *trap-to-trap* distillation under reduced pressure followed by recrystallization from 2-propanol/hexanes.

Beige needles (1.66 g, 6.97 mmol). Yield: 63 %.

^{1}H NMR (300 MHz, CD_2Cl_2) δ 8.39 (dd, J = 4.7, 1.6 Hz, 1H), 8.23 (s, 1H), 8.09 (dd, J = 8.0, 1.6 Hz, 1H), 7.55 – 7.47 (m, 2H), 7.27 (dd, J = 8.1, 4.8 Hz, 1H), 6.89 – 6.81 (m, 2H), 3.02 (s, 6H).

^{13}C{^{1}H} NMR (75 MHz, CD_2Cl_2) δ 150.9, 148.1, 145.0, 144.2, 136.3, 128.3, 125.7, 124.7, 118.9, 113.1, 40.9.

MS (ESI-HRMS): calculated for $[C_{14}H_{15}N_4]^+$ 239.1291, measured 239.1292 (error 0.4 ppm).

3-(4-(methylthio)phenyl)-3H-imidazo[4,5-b]pyridine (26)

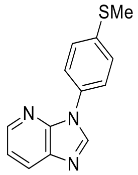

26

The compound was obtained via the Zn-involved process. The first stage of the synthesis was carried out using ethanol (1.92 mL, 33.0 mmol, 3.0 eq). The compound was purified employing the hydrochloride precipitation method.

Purplish crystalline solid (1.65 g, 6.82 mmol). Yield: 62 %.

^{1}H NMR (400 MHz, $CDCl_3$) δ 8.45 (dd, J = 4.8, 1.6 Hz, 1H), 8.30 (s, 1H), 8.14 (dd, J = 8.0, 1.5 Hz, 1H), 7.69 – 7.64 (m, 2H), 7.46 – 7.41 (m, 2H), 7.30 (dd, J = 8.1, 4.8 Hz, 1H), 2.53 (s, 3H).

^{13}C{^{1}H} NMR (101 MHz, $CDCl_3$) δ 147.0, 145.1, 143.0, 138.9, 136.0, 132.3, 128.5, 127.8, 124.2, 119.1, 16.1.

MS (ESI-HRMS): calculated for $[C_{13}H_{12}N_3S]^+$ 242.0746, measured 242.0745 (error 0.4 ppm).

4-(3H-imidazo[4,5-b]pyridin-3-yl)benzonitrile (27)

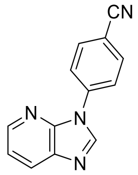

27

The compound was obtained via the Zn-involved process. The first stage of the synthesis was carried out using ethanol (321 µL, 5.5 mmol, 0.5 eq) and NMP (3 mL). The compound was purified by means of *trap-to-trap* distillation under reduced pressure followed by recrystallization from 2-propanol/hexanes.

White fluffy solid (1.41 g, 6.40 mmol). Yield: 58 %.

^{1}H NMR (300 MHz, $CDCl_3$) δ 8.48 (dd, J = 4.8, 1.5 Hz, 1H), 8.42 (s, 1H), 8.18 (dd, J = 8.2, 1.5 Hz, 1H), 8.10 – 8.03 (m, 2H), 7.91 – 7.84 (m, 2H), 7.37 (dd, J = 8.1, 4.8 Hz, 1H).

^{13}C{^{1}H} NMR (75 MHz, $CDCl_3$) δ 146.5, 145.4, 142.0, 139.1, 136.4, 134.0, 129.0, 123.1, 119.8, 118.2, 111.3.

MS (ESI-HRMS): calculated for $[C_{13}H_9N_4]^+$ 221.0822, measured 221.0818 (error 1.8 ppm).

3-(3H-imidazo[4,5-b]pyridin-3-yl)benzonitrile (28)

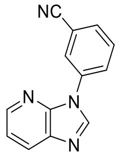

28

The compound was obtained via the Zn-involved process. The first stage of the synthesis was carried out using ethanol (321 µL, 5.5 mmol, 0.5 eq) and NMP (3 mL). The compound was purified by means of *trap-to-trap* distillation under reduced pressure followed by recrystallization from 2-propanol/hexanes.

White fluffy solid (1.55 g, 7.04 mmol). Yield: 64 %.

^{1}H NMR (300 MHz, $CDCl_3$) δ 8.47 (dd, *J* = 4.8, 1.5 Hz, 1H), 8.38 (s, 1H), 8.26 – 8.22 (m, 1H), 8.17 (dd, *J* = 8.1, 1.5 Hz, 1H), 8.13 – 8.04 (m, 1H), 7.75 – 7.66 (m, 2H), 7.35 (dd, *J* = 8.1, 4.8 Hz, 1H).

$^{13}C\{^{1}H\}$ NMR (75 MHz, $CDCl_3$) δ 146.5, 145.4, 142.1, 136.3, 136.2, 131.1, 130.9, 128.9, 127.2, 126.5, 119.7, 117.9, 114.2.

MS (ESI-HRMS): calculated for $[C_{13}H_9N_4]^+$ 221.0822, measured 221.0824 (error 0.9 ppm).

7-methyl-3-phenyl-3H-imidazo[4,5-b]pyridine (29)

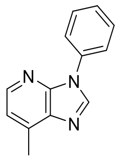

29

The compound was obtained via the Ni-catalyzed process. The first stage of the synthesis was carried out at 160 ºC, and the second one at 140 ºC. The compound was purified via *trap-to-trap* distillation under reduced pressure followed by the hydrochloride precipitation method.

Pink needles (1.70 g, 8.14 mmol). Yield: 74 %.

^{1}H NMR (300 MHz, $CDCl_3$) δ 8.36 – 8.30 (m, 2H), 7.78 – 7.72 (m, 2H), 7.62 – 7.53 (m, 2H), 7.48 – 7.41 (m, 1H), 7.14 (dd, *J* = 4.9, 0.8 Hz, 1H), 2.75 (d, *J* = 0.9 Hz, 3H).

$^{13}C\{^{1}H\}$ NMR (75 MHz, $CDCl_3$) δ 146.4, 145.0, 142.0, 140.1, 135.7, 135.4, 129.9, 127.9, 123.7, 120.0, 16.4.

MS (ESI-HRMS): calculated for $[C_{13}H_{12}N_3]^+$ 210.1026, measured 210.1027 (error 0.5 ppm).

6-methyl-3-phenyl-3H-imidazo[4,5-b]pyridine (30)

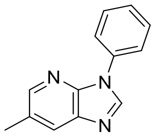

30

The compound was obtained via the Ni-catalyzed process. The first stage of the synthesis was carried out at 160 ºC, and the second one at 140 ºC. The compound was purified via *trap-to-trap* distillation under reduced pressure followed by recrystallization from 2-propanol/hexanes.

Brownish crystalline solid (1.88 g, 9.0 mmol). Yield: 82 %.

^{1}H NMR (300 MHz, $CDCl_3$) δ 8.34 (s, 1H), 8.31 (d, *J* = 1.7 Hz, 1H), 7.97 – 7.94 (m, 1H), 7.78 – 7.72 (m, 2H), 7.61 – 7.54 (m, 2H), 7.47 – 7.41 (m, 1H), 2.51 (s, 3H).

$^{13}C\{^{1}H\}$ NMR (75 MHz, $CDCl_3$) δ 145.9, 145.2, 143.0, 136.0, 135.4, 129.9, 128.7, 128.4, 127.9, 123.5, 18.7.

MS (ESI-HRMS): calculated for $[C_{13}H_{12}N_3]^+$ 210.1026, measured 210.1030 (error 1.9 ppm).

5-methoxy-3-phenyl-3H-imidazo[4,5-b]pyridine (31)

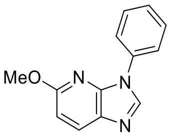

31

The compound was obtained via the Ni-catalyzed process. The first stage of the synthesis was carried out at 140 ºC for 60 minutes, and the second one at 140 ºC, the second heating run (after the addition of the formic acid) extended to 60 minutes. The compound was purified via *trap-to-trap* distillation under reduced pressure followed by recrystallization from 2-propanol/hexanes.

Pinkish crystalline solid (1.93 g, 8.58 mmol). Yield: 78 %.

^{1}H NMR (300 MHz, $CDCl_3$) δ 8.17 (s, 1H), 8.00 (d, *J* = 8.7 Hz, 1H), 7.83 – 7.77 (m, 2H), 7.58 – 7.50 (m, 2H), 7.43 – 7.36 (m, 1H), 6.75 (d, *J* = 8.7 Hz, 1H), 3.96 (s, 3H).

^{13}C{^{1}H} NMR (101 MHz, $CDCl_3$) δ 161.9, 143.9, 140.0, 135.7, 131.2, 130.8, 129.7, 127.4, 122.9, 107.1, 53.9.

MS (ESI-HRMS): calculated for $[C_{13}H_{12}N_3O]^+$ 226.0975, measured 226.0976 (error 0.4 ppm).

6-bromo-3-phenyl-3H-imidazo[4,5-b]pyridine (32)

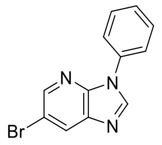

32

The compound was obtained via the Ni-catalyzed process. The first stage of the synthesis was carried out at 160 °C, and the second one at 140 °C, the second heating run (after the addition of the formic acid) was extended to 180 minutes. The compound was purified via *trap-to-trap* distillation under reduced pressure followed by recrystallization from 2-propanol/hexanes.

White crystalline solid (1.93 g, 7.04 mmol). Yield: 64 %.

^{1}H NMR (300 MHz, $CDCl_3$) δ 8.50 (d, *J* = 2.1 Hz, 1H), 8.33 (s, 1H), 8.29 (d, *J* = 2.1 Hz, 1H), 7.76 – 7.69 (m, 2H), 7.63 – 7.54 (m, 2H), 7.51 – 7.42 (m, 1H).

^{13}C{^{1}H} NMR (101 MHz, $CDCl_3$) δ 145.9, 145.6, 144.4, 137.3, 134.9, 130.9, 130.1, 128.4, 123.7, 114.7.

MS (ESI-HRMS): calculated for $[C_{12}H_9BrN_3]^+$ 273.9974, measured 273.9964 (error 3.6 ppm).

Procedure for the up-scaled Ni-catalyzed synthesis of 11

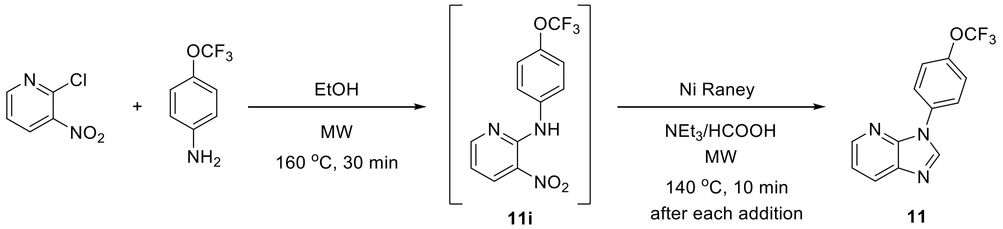

An 80 mL microwave vial was loaded with 2-chloro-3-nitropyridine (33.0 mmol, 1.0 eq), 4-trifluoromethoxyaniline (33.00 mmol, 1.0 eq), and a small amount of ethanol (1.92 mL, 33.0 mmol, 1.0 eq). The vial was capped and subjected to microwave irradiation (160 °C, 30 min, P_{max} = 150 W). After cooling down, the first portion of the Ni catalyst (20 mol%) was transferred to the reaction vial using 11.4 mL of the previously prepared triethylamine (6.19 mL, 44.0 mmol, 1.33 eq) and formic acid (5.19 mL, 110.0 mmol, 3.33 eq) mixture. The vial was purged with argon, closed, and placed into the reactor. The reaction was subjected to microwave irradiation (140 °C, 10 min, P_{max} = 100 W). After cooling down, extra formic acid was added (2.6 mL, 55.0 mmol, 1.66 eq), the vial was purged with argon, closed, and heated once again in the same conditions. After cooling down, the second portion of the Ni catalyst (20 mol%) and formic acid (2.6 mL, 55.0 mmol, 1.66 eq),) were added, he vial was purged with argon, closed, and heated once again in the same conditions. Finally, the vial was cooled down, extra formic acid was added (2.6 mL, 55.0 mmol, 1.66 eq), the vial was purged with argon, closed, and heated last time in the same conditions. After cooling down, the product was isolated in the same way as reported for the small scale synthesis. The product was obtained with 77 % yield (7.09 g, 25.4 mmol).

N-(2-(phenylamino)pyridin-3-yl)formamide (1i-CHO)

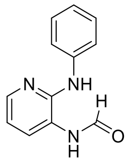

1i-CHO

The compound was obtained according to the reported procedure [4]. The NMR spectra revealed the presence of 2 tautomeric forms.

White crystalline solid.

^{1}H NMR (300 MHz, DMSO-d_6) δ 9.83 – 9.55 (m, 1H), 8.45 – 8.31 (m, 1H), 8.22 (s, 0.28H), 8.10 (s, 0.7H), 8.06 – 7.97 (m, 1H), 7.84 (dd, J = 7.8, 1.8 Hz, 0.73H), 7.72 – 7.50 (m, 2.3H), 7.26 (t, J = 8.0 Hz, 2H), 6.99 – 6.88 (m, 1H), 6.88 – 6.76 (m, 1H).

^{13}C{^{1}H} NMR (75 MHz, DMSO-d_6) δ 163.9, 160.8, 148.8, 148.6, 143.9, 143.7, 141.3, 141.1, 132.2, 130.0, 128.4, 121.1, 120.9, 120.3, 119.7, 119.1, 119.0, 114.9, 114.9.

MS (GC-MS): calculated for $[C_{12}H_{11}N_3O]^+$ 213.1, found 195.0, 194.0. This compound undergoes imidazole ring formation under the elevated temperature of the injector and chromatography column, so only signals corresponding to **1** are observable.

N-(6-methoxy-2-(phenylamino)pyridin-3-yl)formamide (31i-CHO)

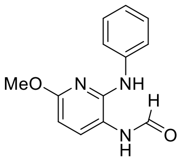

31i-CHO

The compound was isolated as a byproduct of the synthesis of **31**, which was carried out without excessive heating at the last stage (the last heating cycle was shortened from 60 to 10 minutes). Instead of *trap-to-trap* distillation, the compound was purified by column chromatography. Elution with 3 % v/v MeOH in DCM yielded **31**, while **31i-CHO** was obtained by eluting with 5 % v/v MeOH in DCM. The material was crystallized by a slow evaporation of its dichloromethane solution. The NMR spectra revealed the presence of 2 tautomeric forms.

White crystalline solid.

^{1}H NMR (300 MHz, DMSO-d_6) δ 9.47 (s, 0.73H), 9.28 (d, J = 10.9 Hz, 0.25H), 8.34 – 8.23 (m, 1H), 8.19 (d, J = 10.8 Hz, 0.26H), 8.09 (s, 0.73H), 7.75 – 7.53 (m, 2.78H), 7.48 (d, J = 8.2 Hz, 0.29H), 7.36 – 7.18 (m, 2H), 7.00 – 6.84 (m, 1H), 6.30 – 6.14 (m, 1H), 3.80 (s, 3H).

^{13}C{^{1}H} NMR (75 MHz, DMSO-d_6) δ 164.1, 160.8, 160.3, 159.8, 147.9, 147.5, 141.0, 140.8, 137.2, 136.0, 128.4, 121.1, 121.0, 119.1, 118.9, 112.2, 111.4, 98.8, 98.8, 53.4.

MS (GC-MS): calculated for $[C_{13}H_{13}N_3O_2]^+$ 243.1, found 243.3, 226.2, 215.1, 200.1.

^{1}H and ^{13}C NMR spectra

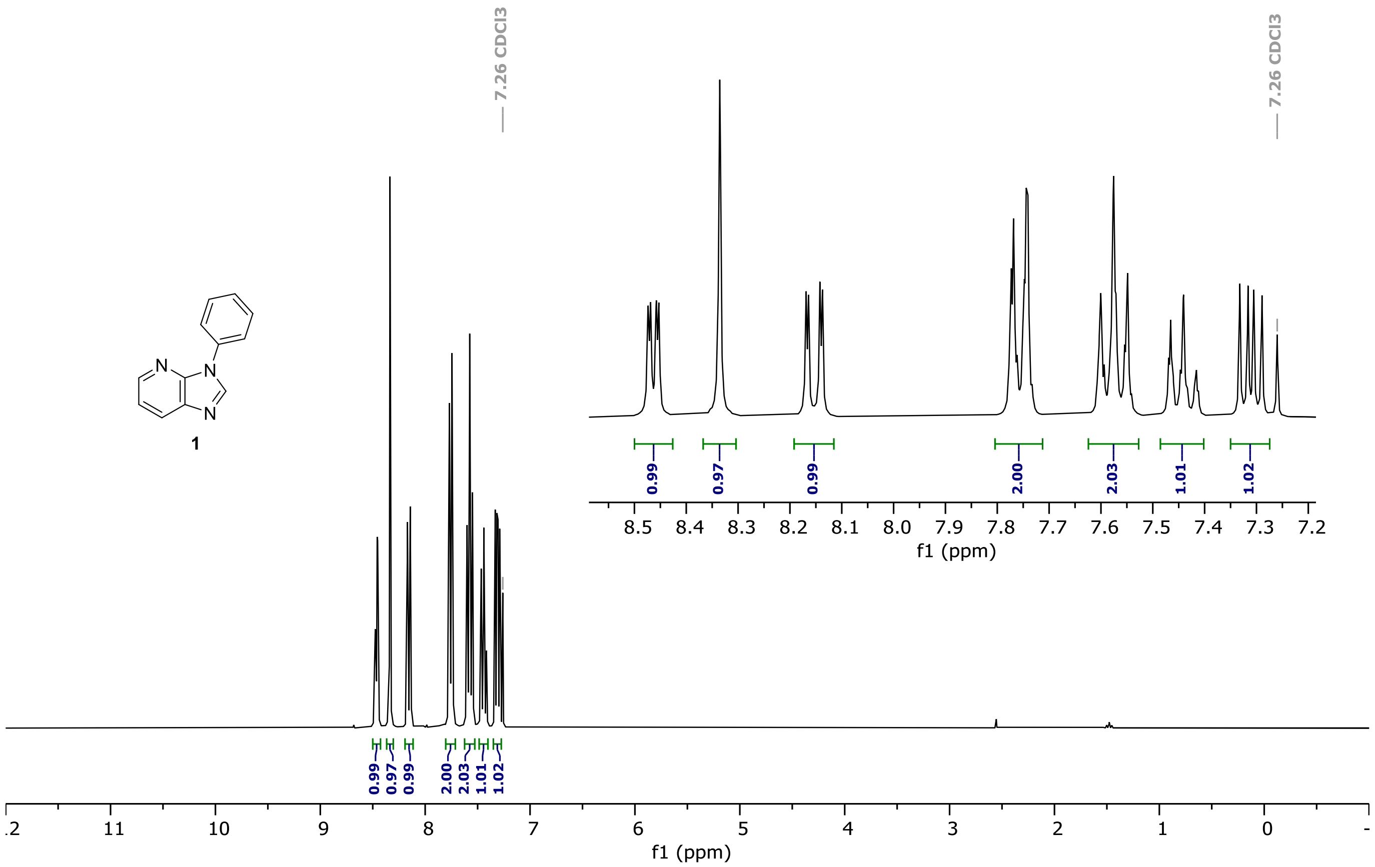

Figure S1. *^{1}H NMR (300 MHz, $CDCl_3$, 298 K) spectrum of **1***

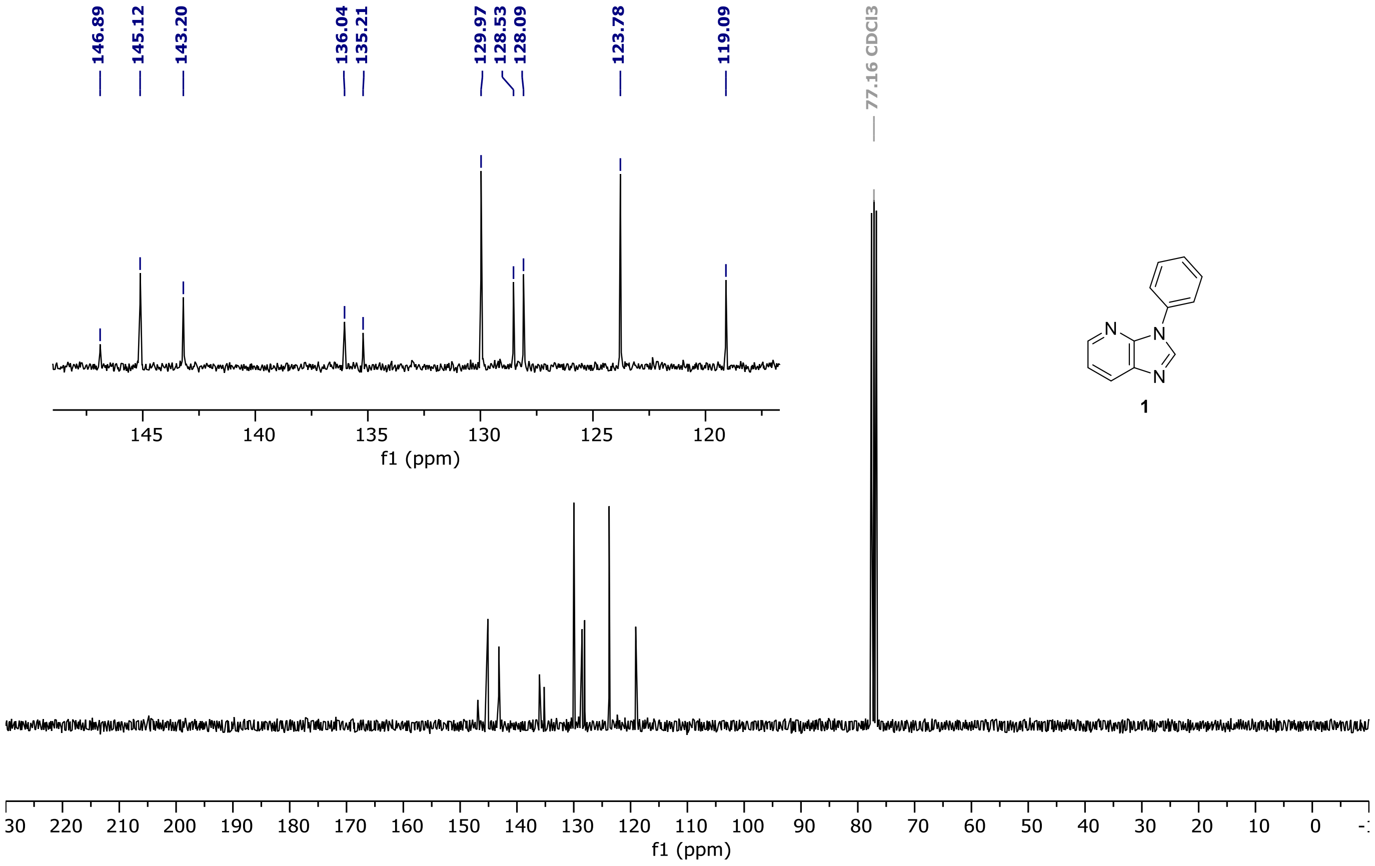

Figure S2. *$^{13}C\{^{1}H\}$ NMR (75 MHz, $CDCl_3$, 298 K) spectrum of **1***

^1H and ^{13}C NMR spectra

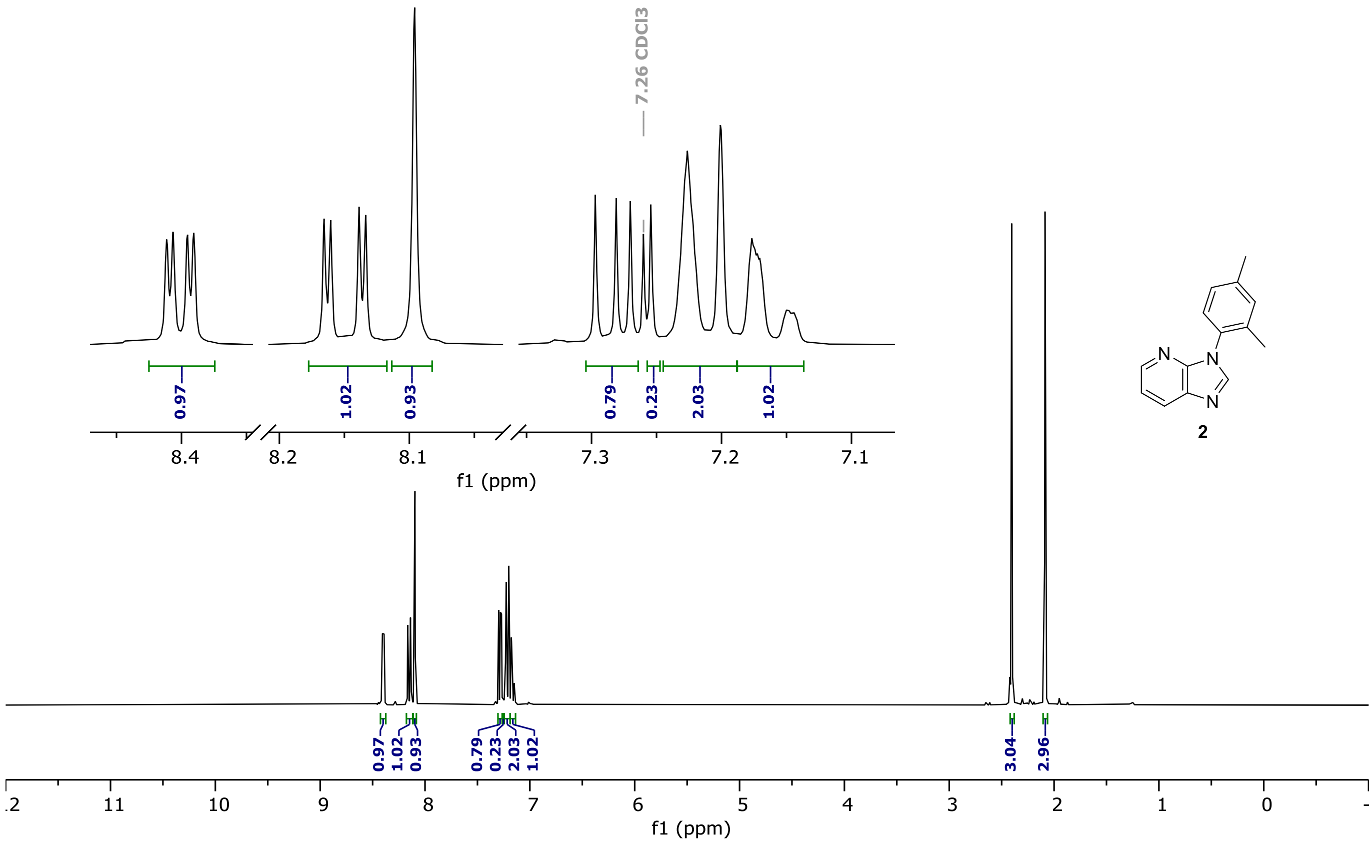

Figure S3. *^1H NMR (300 MHz, $CDCl_3$, 298 K) spectrum of* ***2***

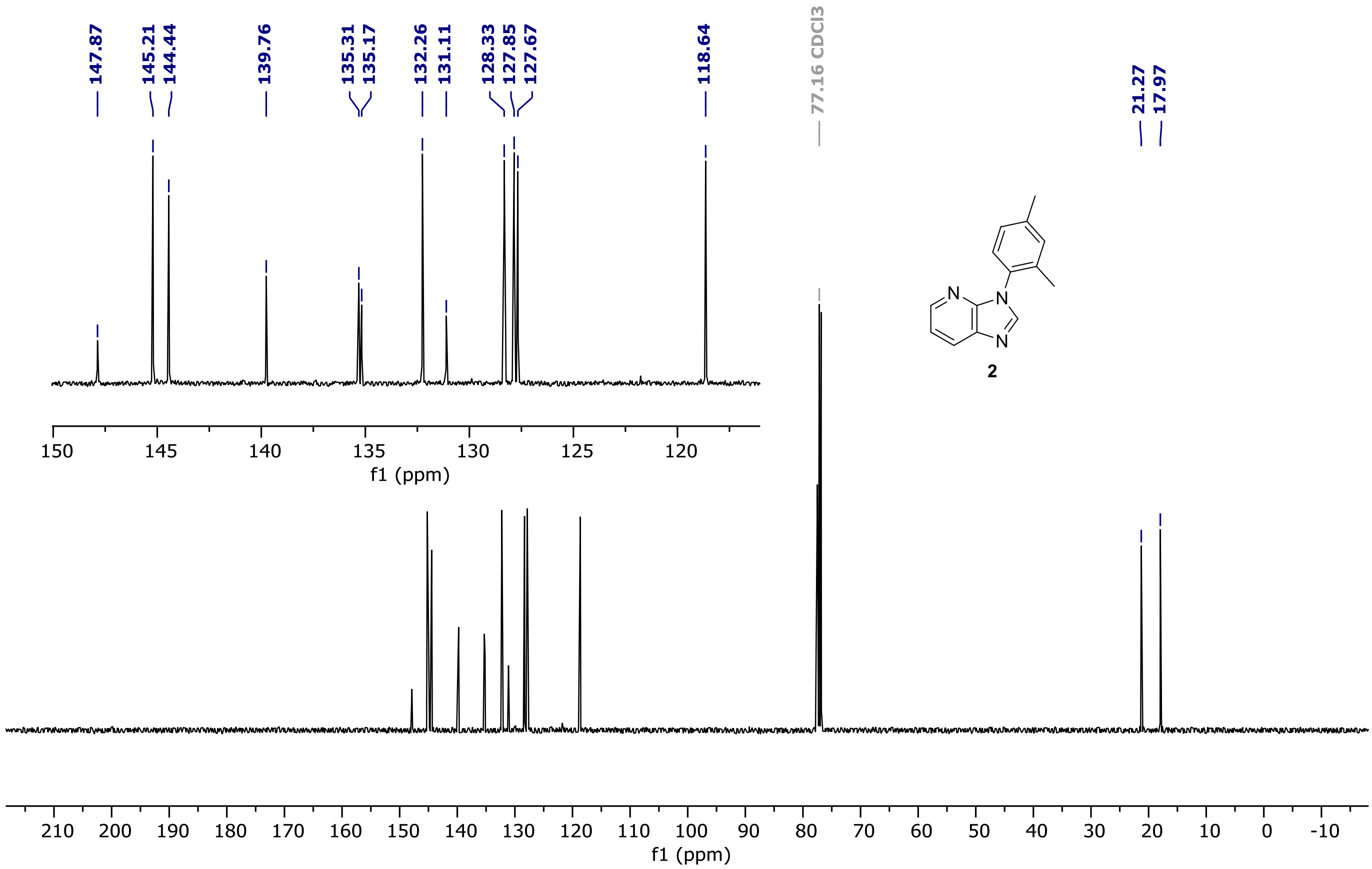

Figure S4. *$^{13}C\{^1H\}$ NMR (75 MHz, $CDCl_3$, 298 K) spectrum of* ***2***

^{1}H and ^{13}C NMR spectra

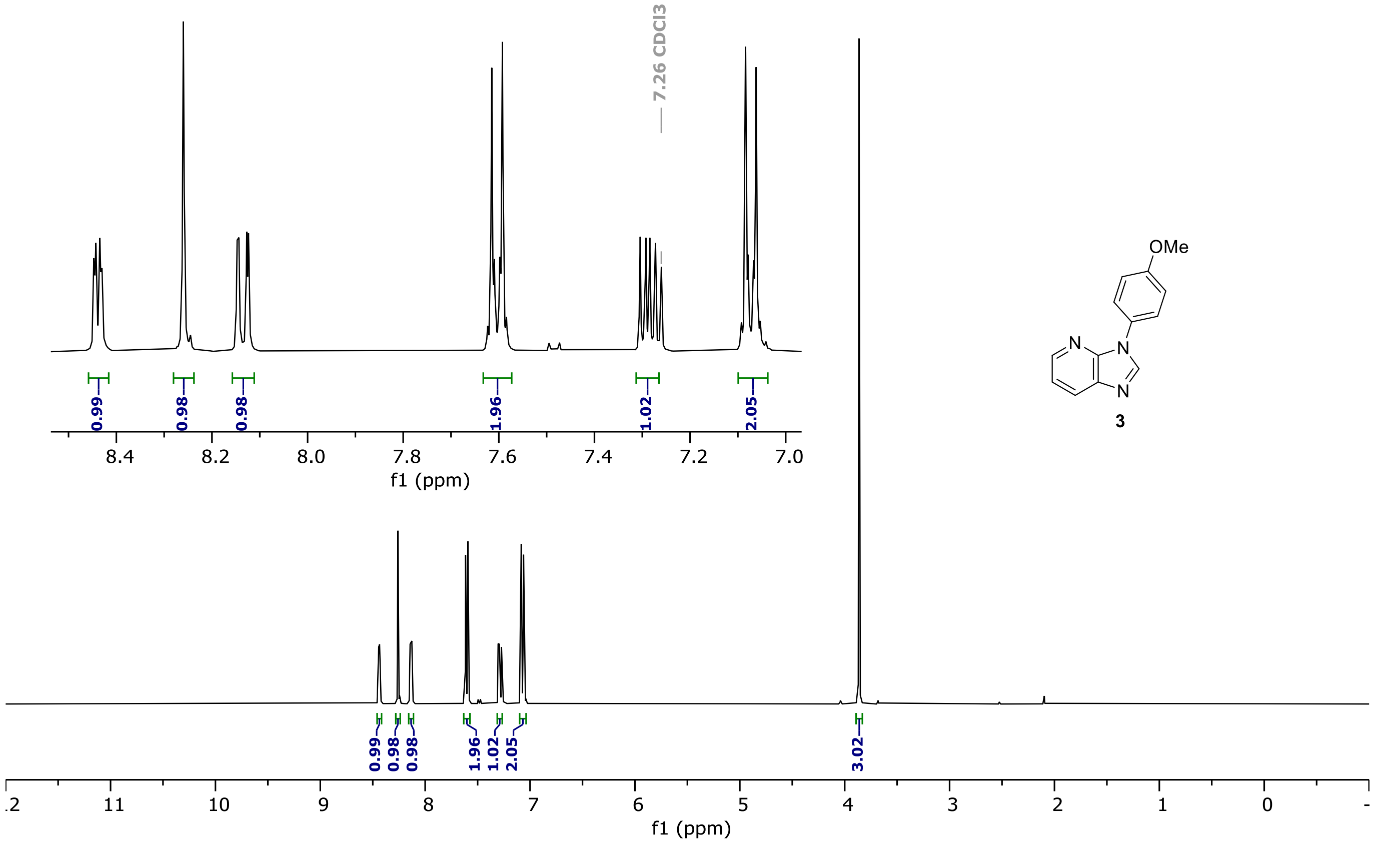

Figure S5. *^{1}H NMR (300 MHz, $CDCl_3$, 298 K) spectrum of* ***3***

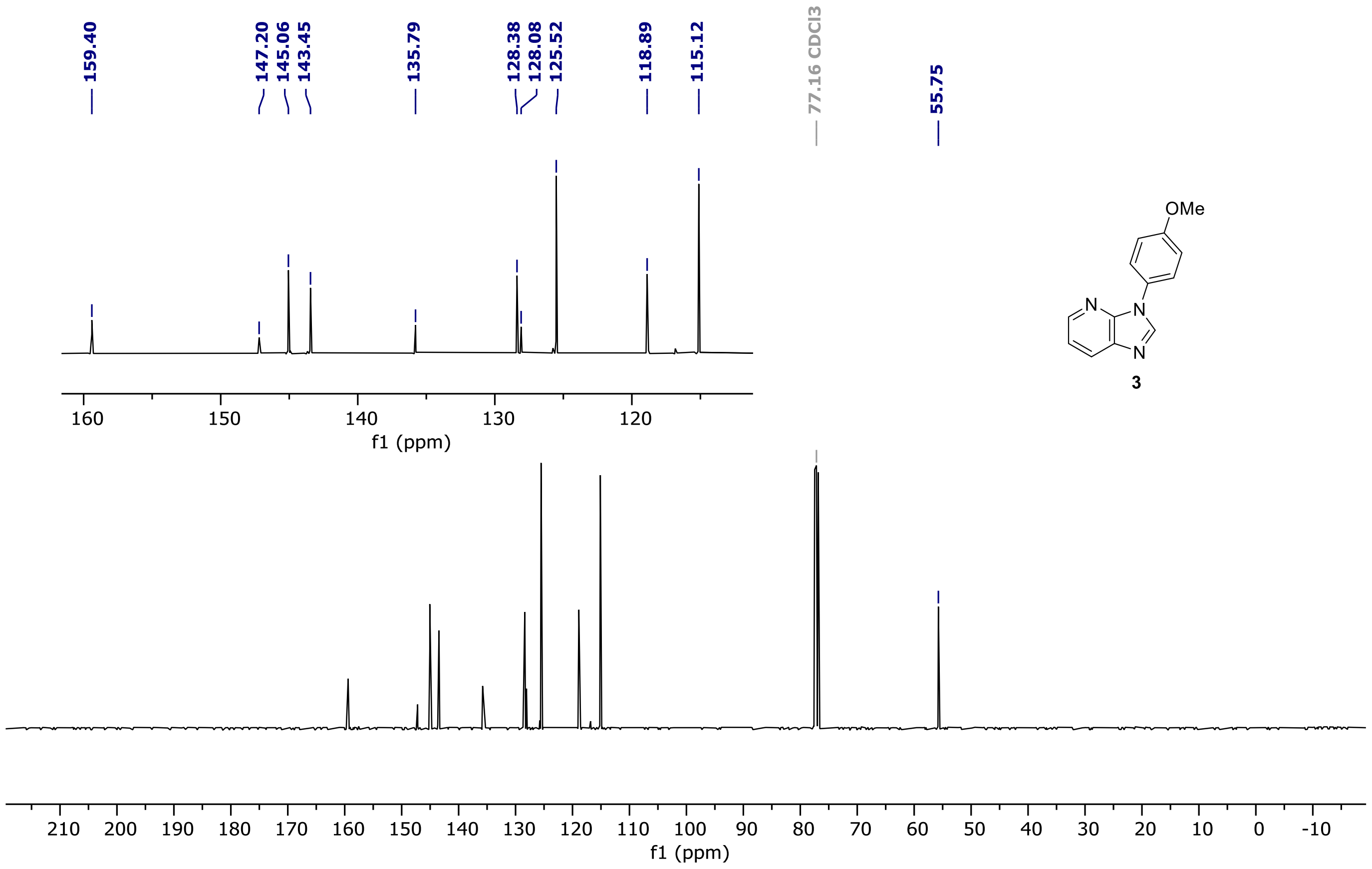

Figure S6. *$^{13}C\{^{1}H\}$ NMR (75 MHz, $CDCl_3$, 298 K) spectrum of* ***3***

^{1}H and ^{13}C NMR spectra

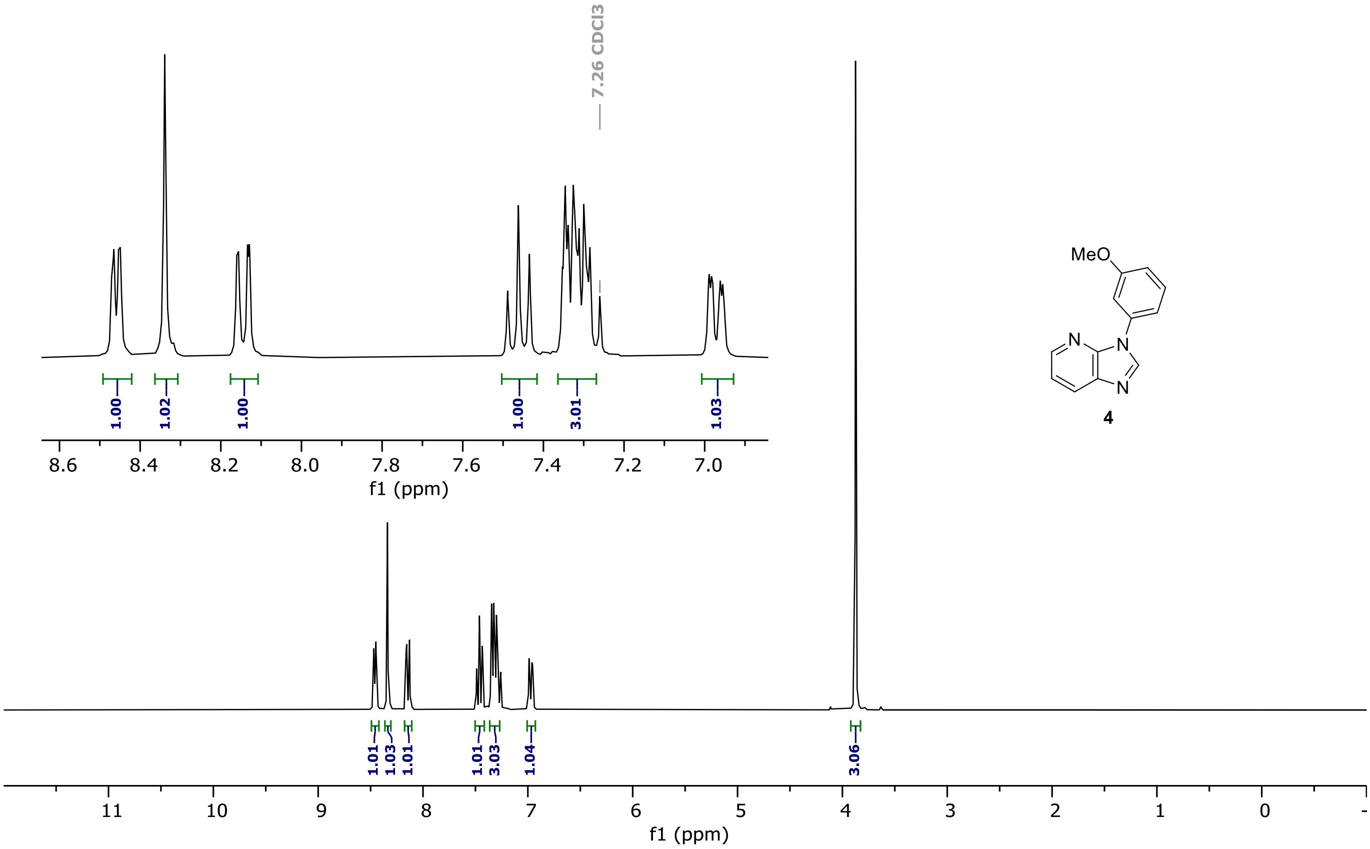

Figure S7. *^{1}H NMR (300 MHz, $CDCl_3$, 298 K) spectrum of* ***4***

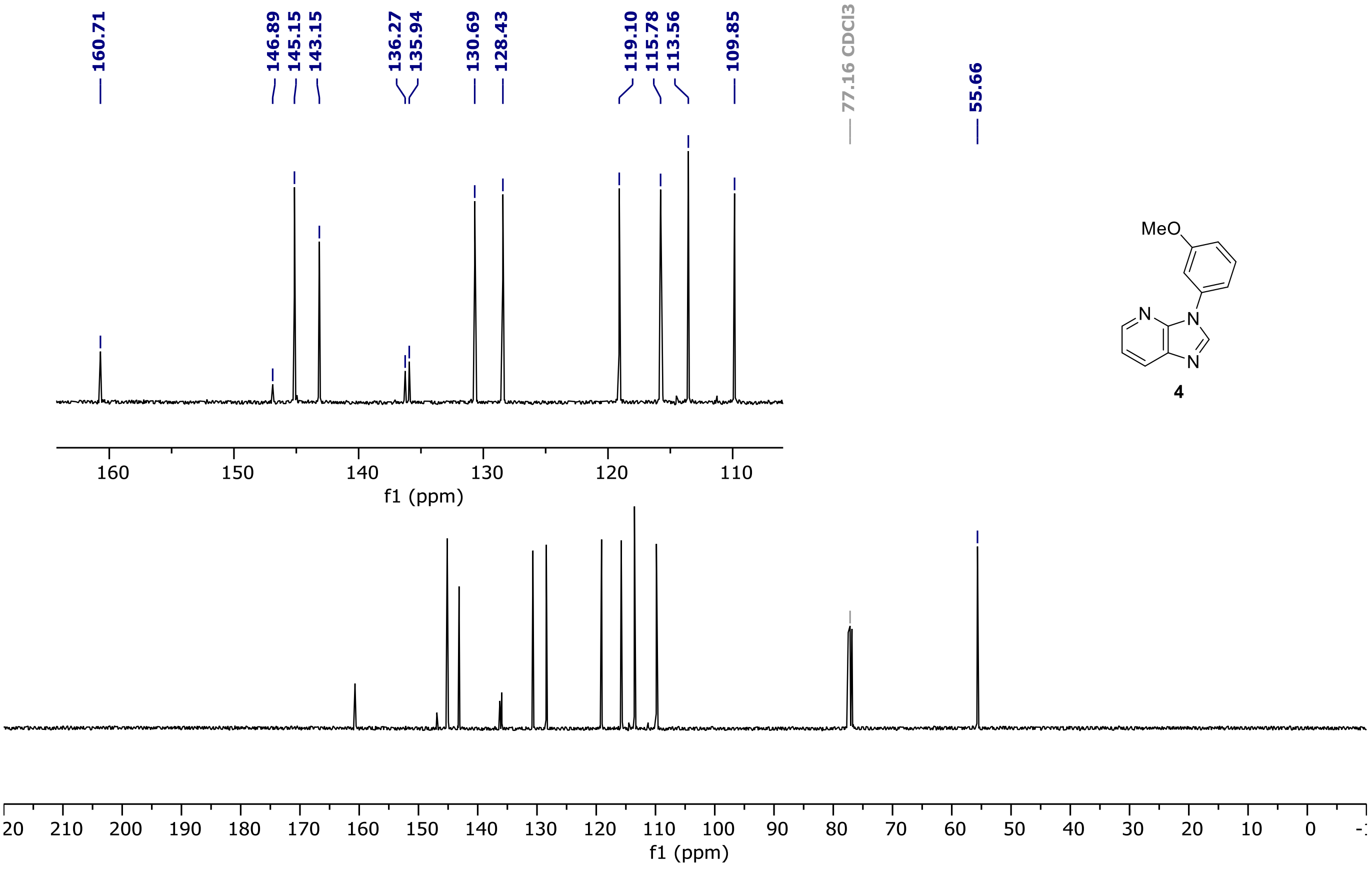

Figure S8. *^{13}C{^{1}H} NMR (75 MHz, $CDCl_3$, 298 K) spectrum of* ***4***

^{1}H and ^{13}C NMR spectra

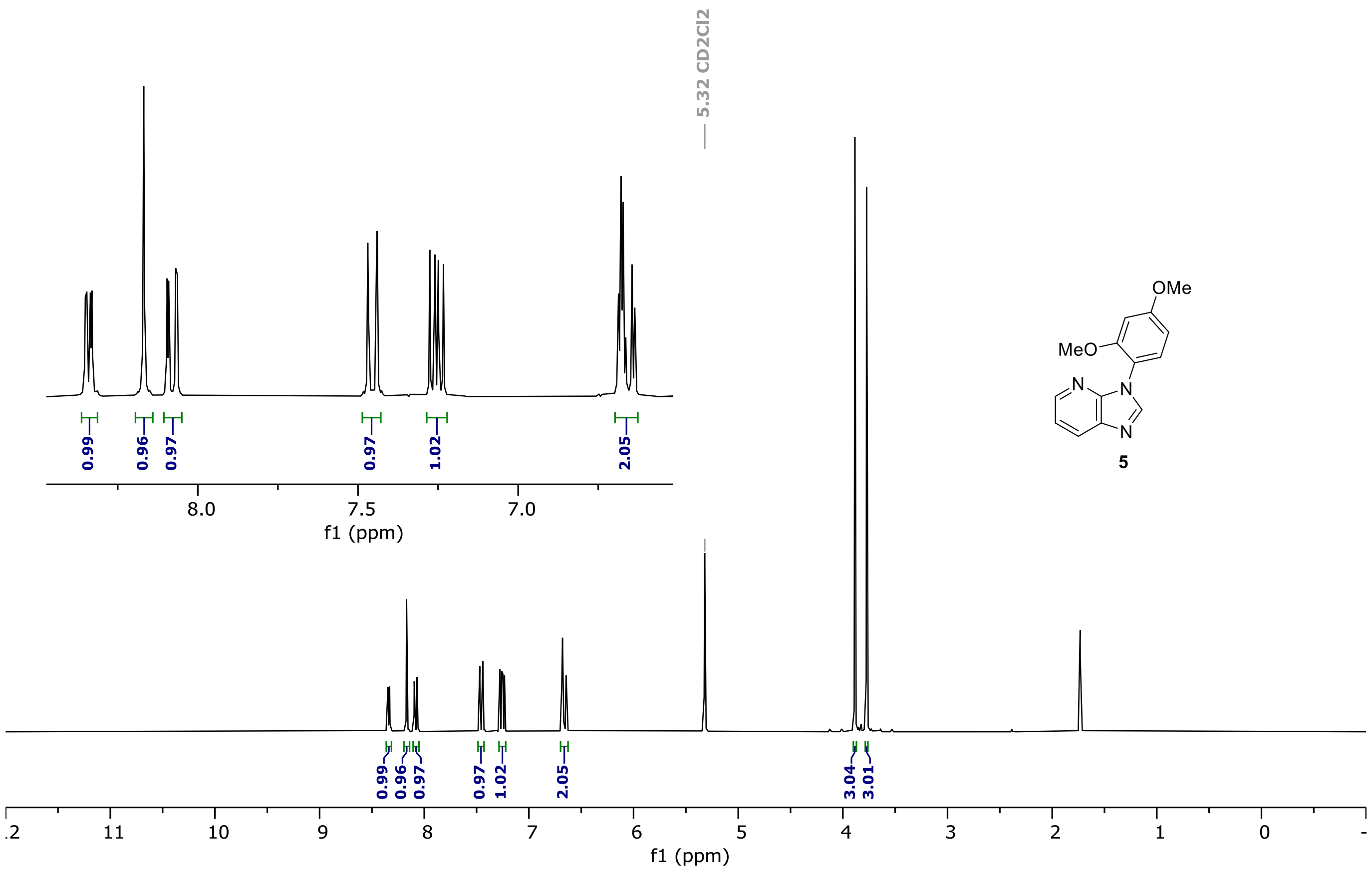

Figure S9. *^{1}H NMR (300 MHz, CD_2Cl_2, 298 K) spectrum of **5***

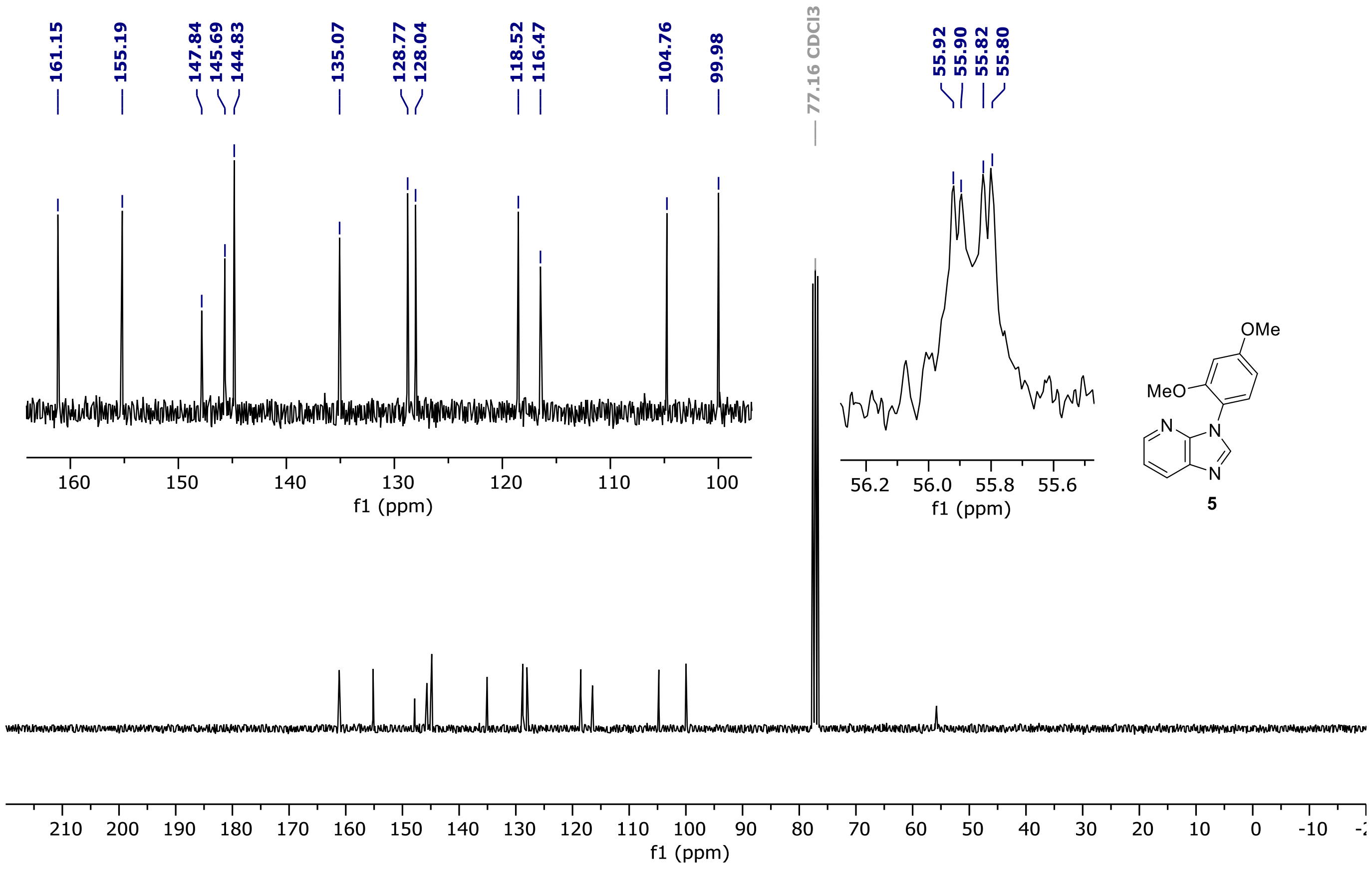

Figure S10. *$^{13}C\{^1H\}$ NMR (75 MHz, $CDCl_3$, 298 K) spectrum of **5***

^{1}H and ^{13}C NMR spectra

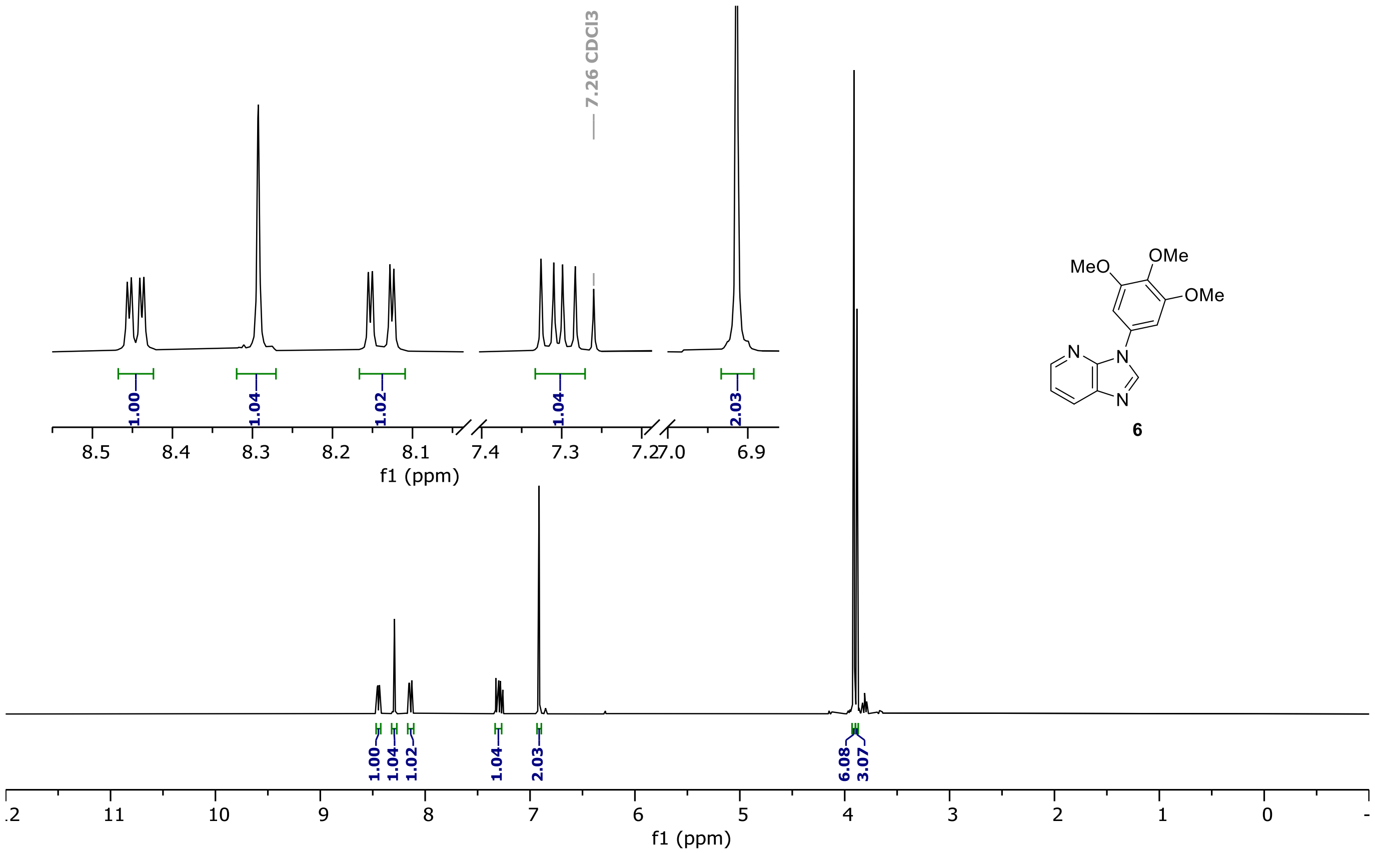

Figure S11. *^{1}H NMR (300 MHz, $CDCl_3$, 298 K) spectrum of **6***

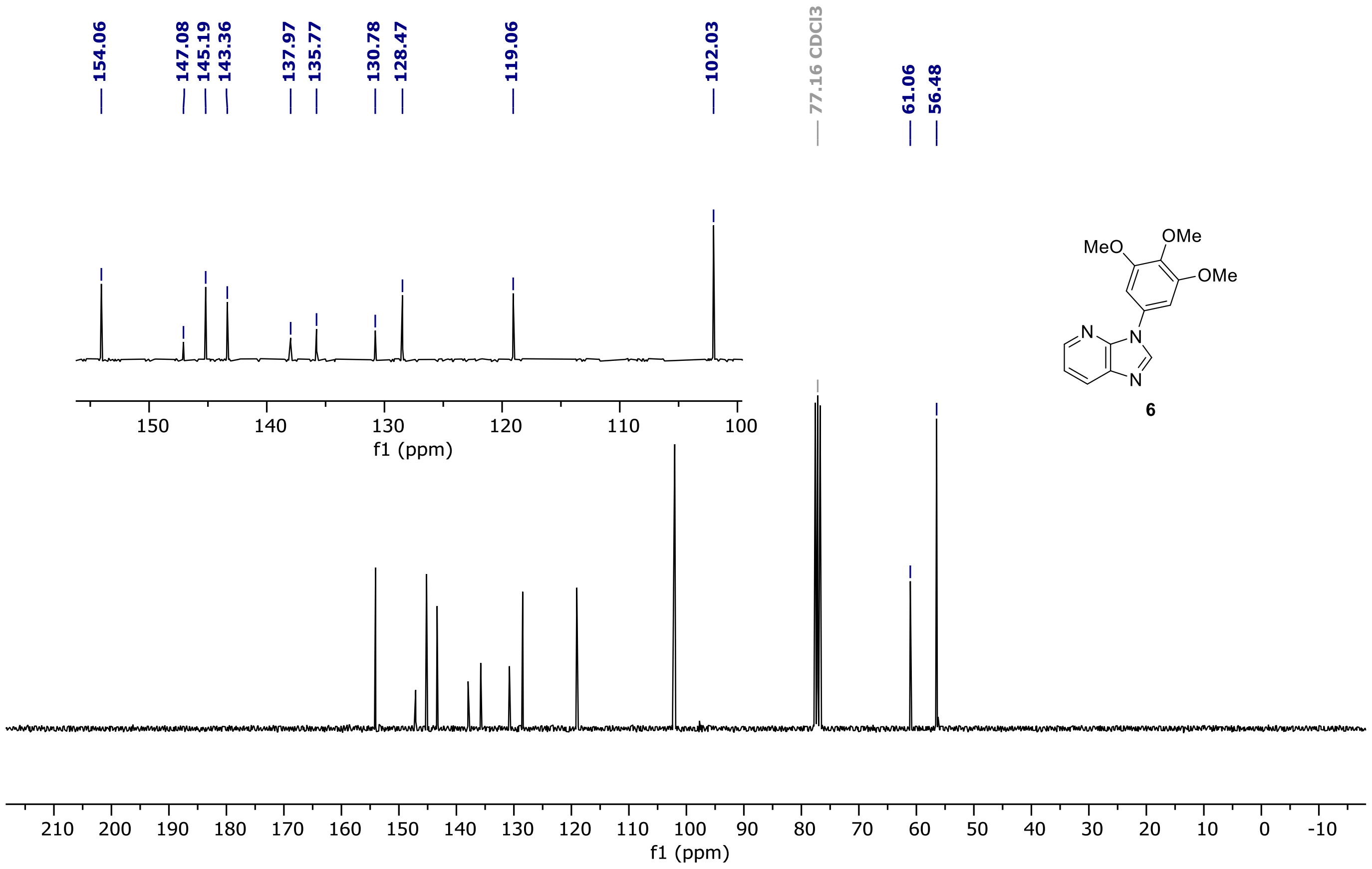

Figure S12. *$^{13}C\{^1H\}$ NMR (75 MHz, $CDCl_3$, 298 K) spectrum of **6***

^{1}H and ^{13}C NMR spectra

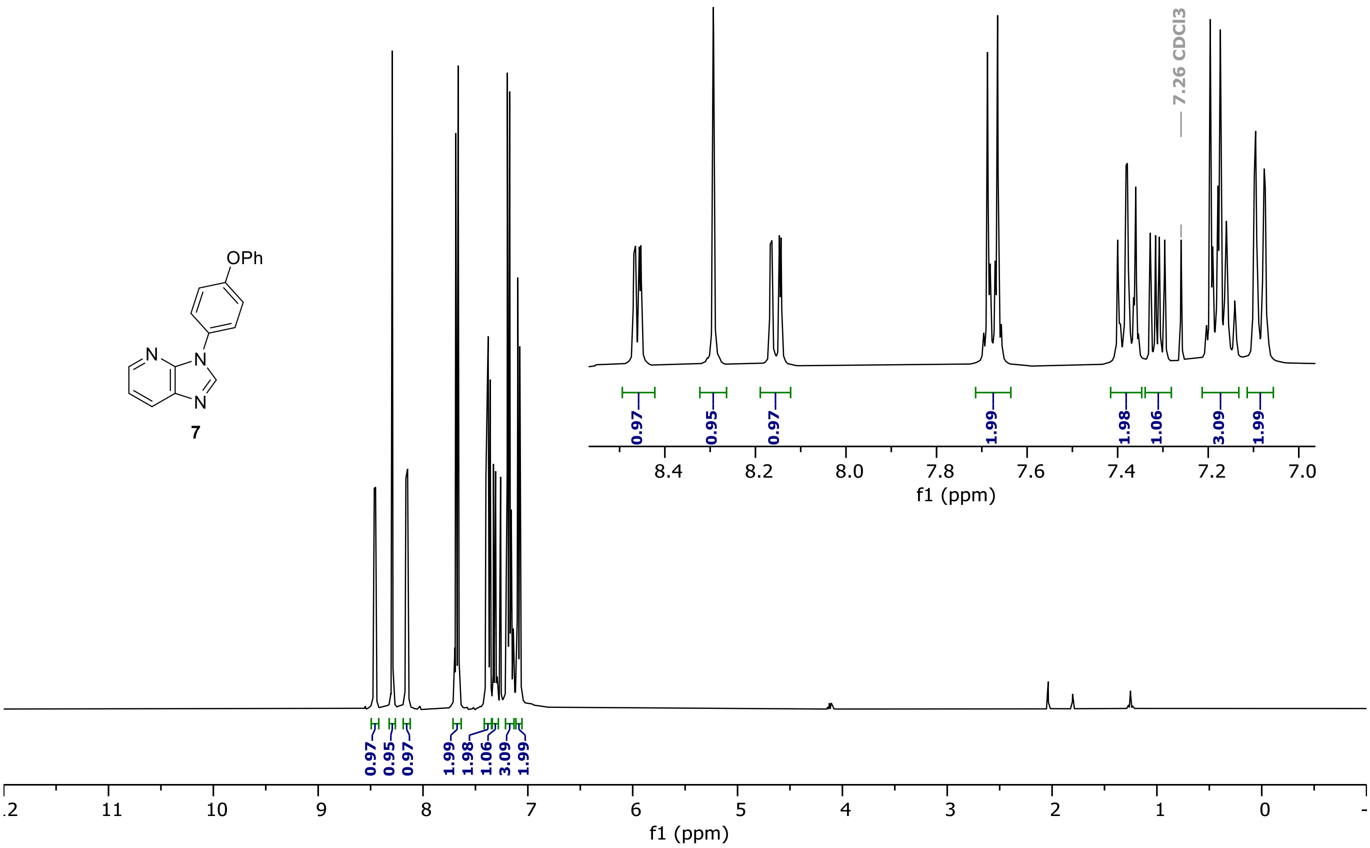

Figure S13. ^{1}H NMR (300 MHz, $CDCl_3$, 298 K) spectrum of **7**

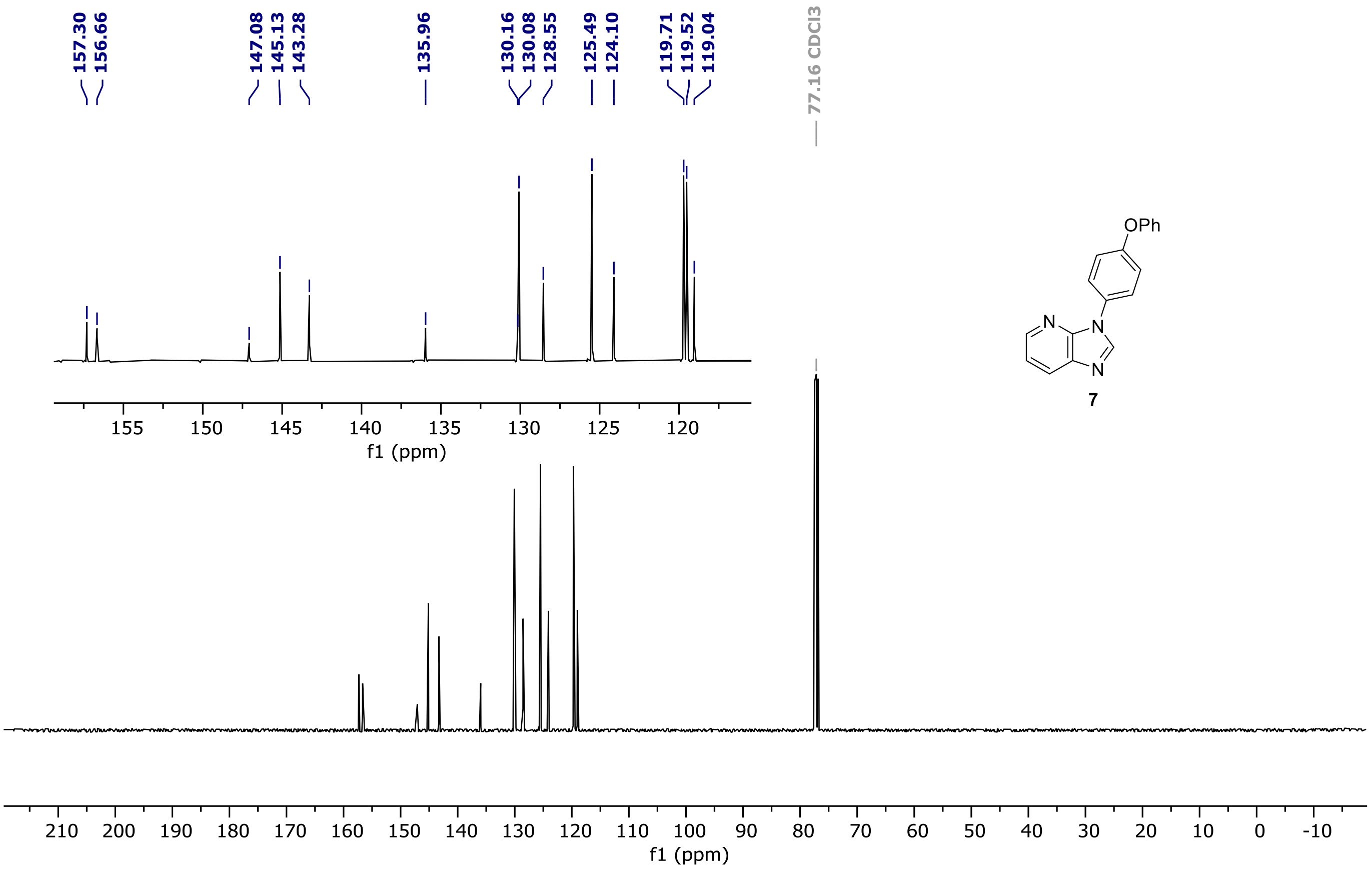

Figure S14. $^{13}C\{^{1}H\}$ NMR (75 MHz, $CDCl_3$, 298 K) spectrum of **7**

^{1}H and ^{13}C NMR spectra

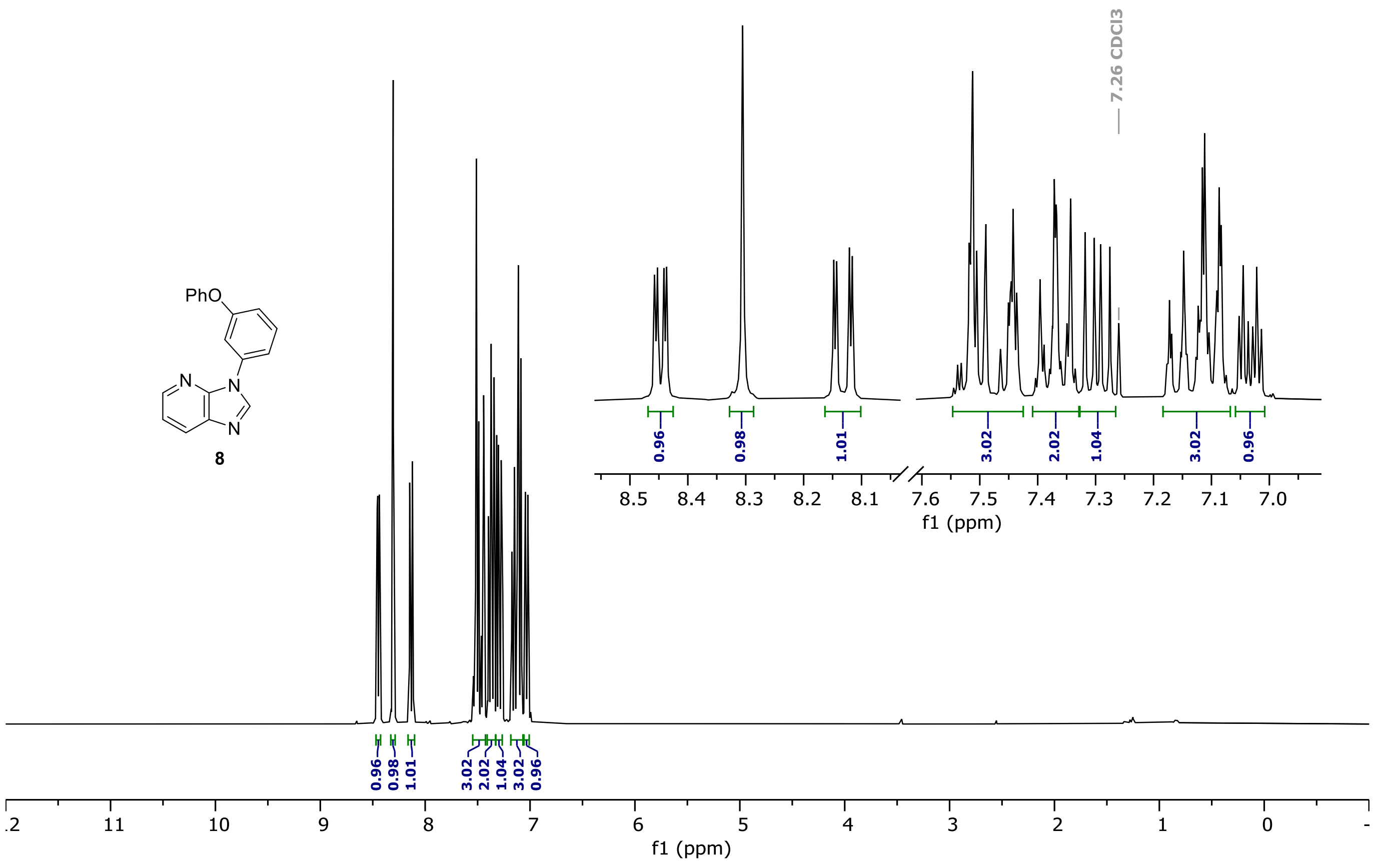

Figure S15. *^{1}H NMR (300 MHz, $CDCl_3$, 298 K) spectrum of* ***8***

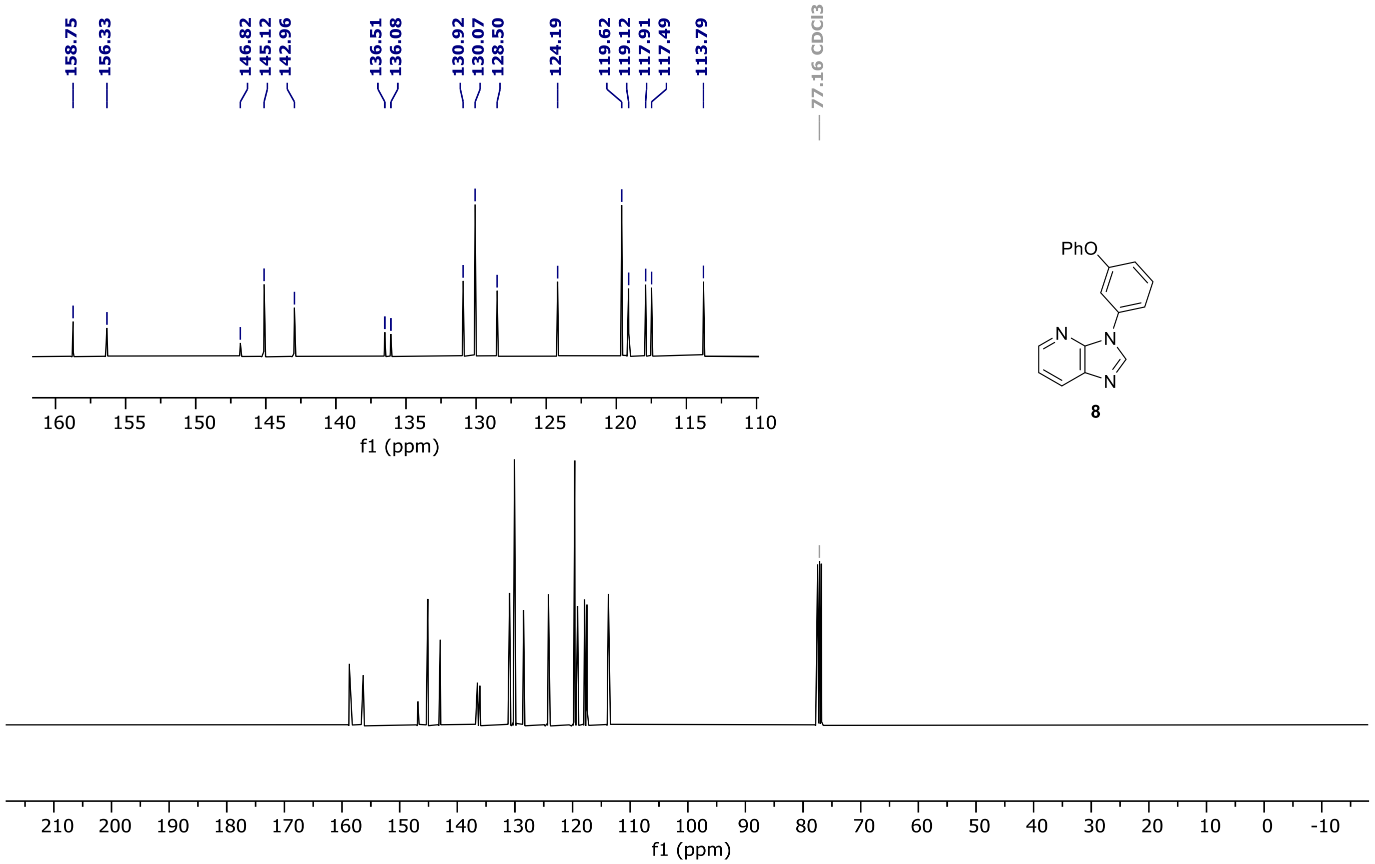

Figure S16. *$^{13}C\{^{1}H\}$ NMR (300 MHz, $CDCl_3$, 298 K) spectrum of* ***8***

^{1}H and ^{13}C NMR spectra

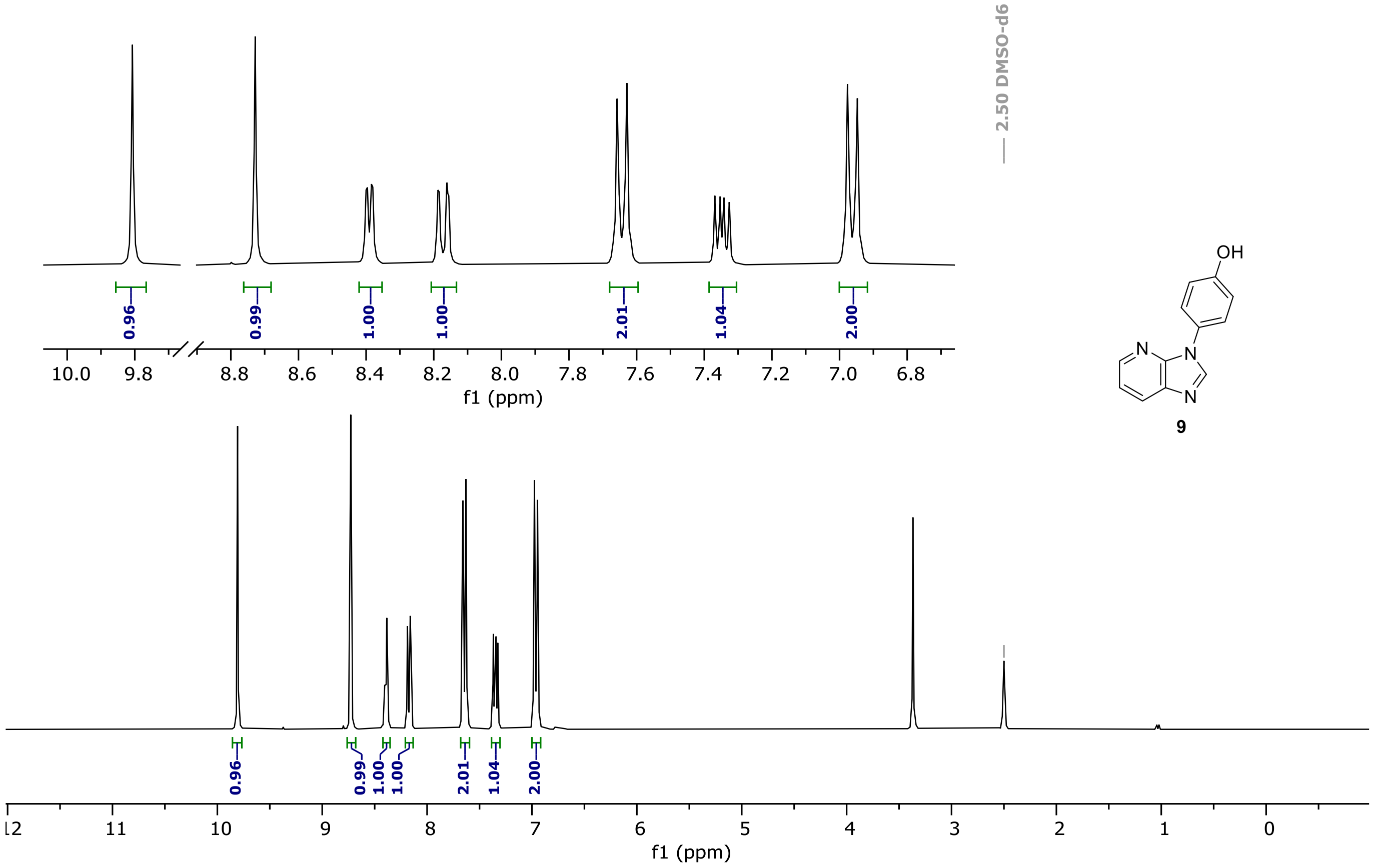

Figure S17. *^{1}H NMR (300 MHz, DMSO-d_6, 298 K) spectrum of* ***9***

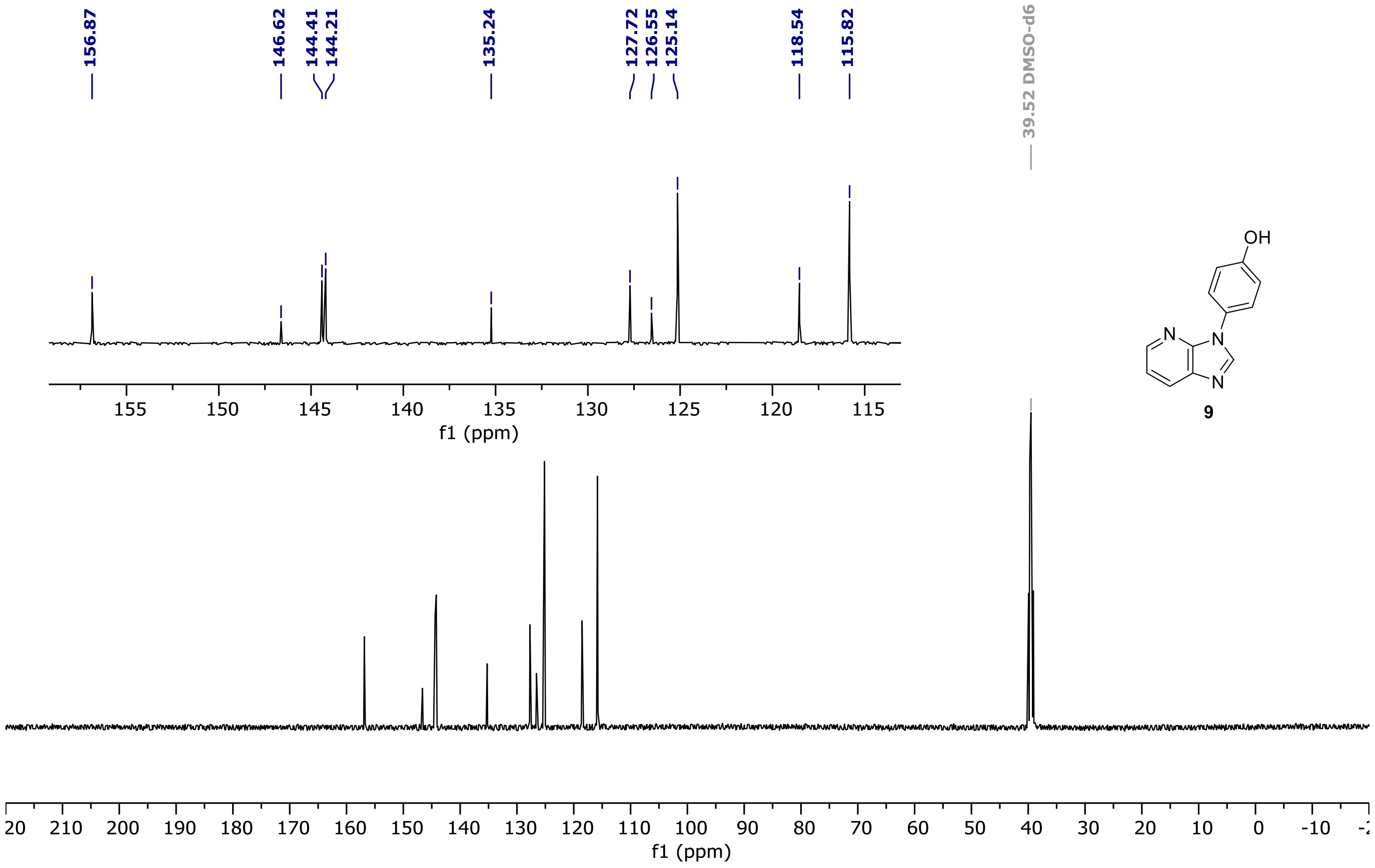

Figure S18. *$^{13}C\{^{1}H\}$ NMR (75 MHz, DMSO-d_6, 298 K) spectrum of* ***9***

^{1}H and ^{13}C NMR spectra

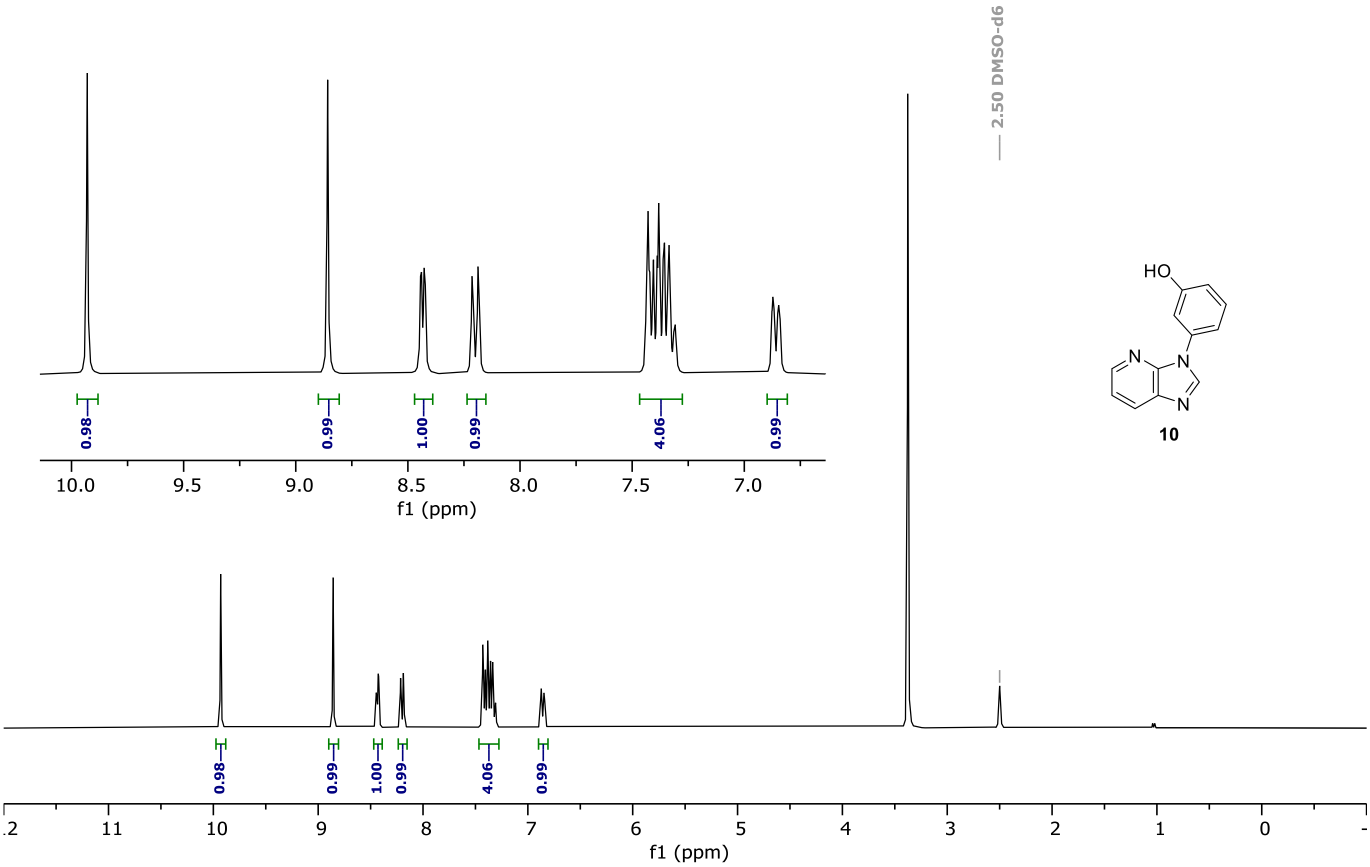

Figure S19. *^{1}H NMR (300 MHz, DMSO-d_6, 298 K) spectrum of* ***10***

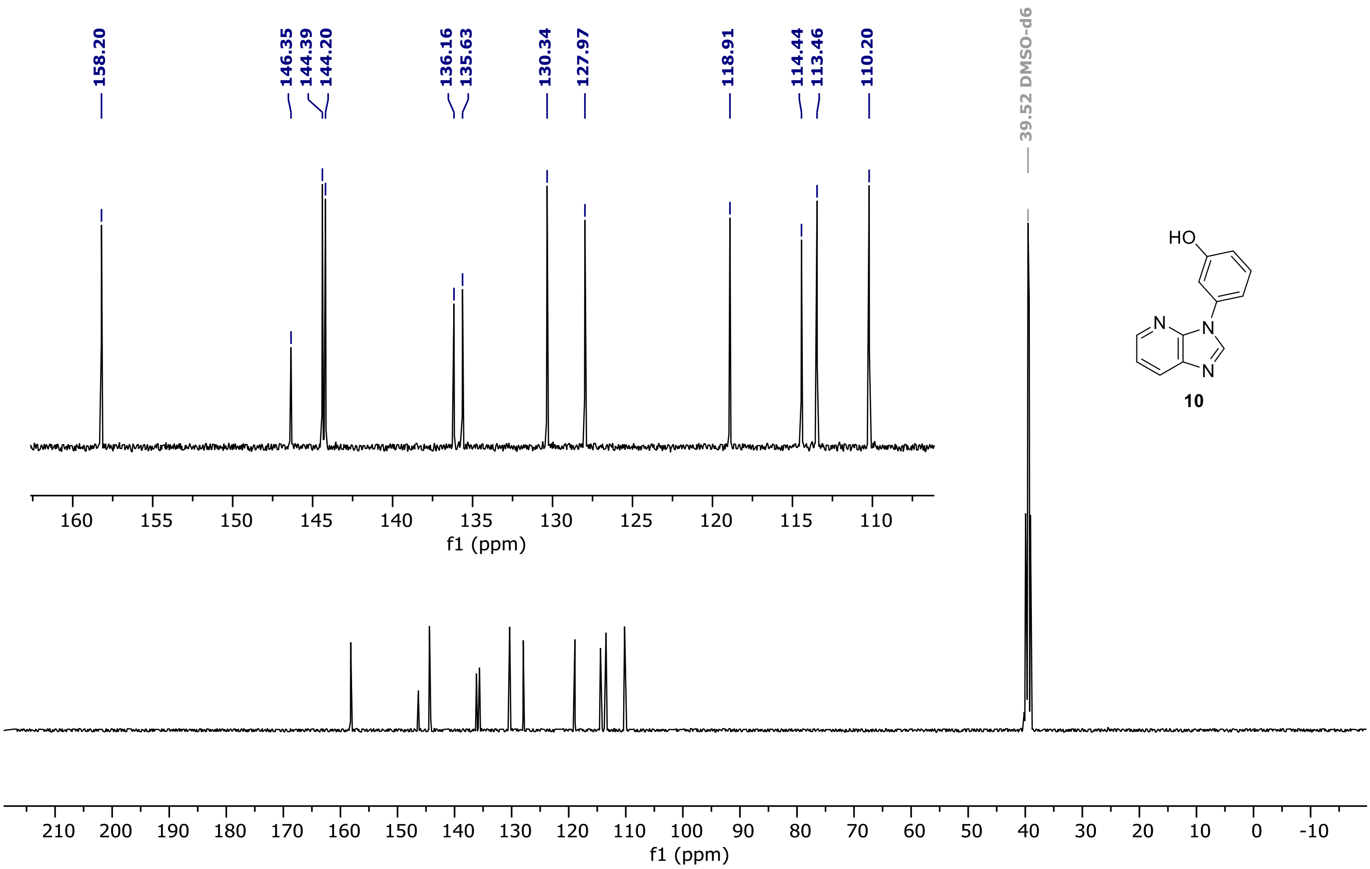

Figure S20. *$^{13}C\{^{1}H\}$ NMR (75 MHz, DMSO-d_6, 298 K) spectrum of* ***10***

^{1}H and ^{13}C NMR spectra

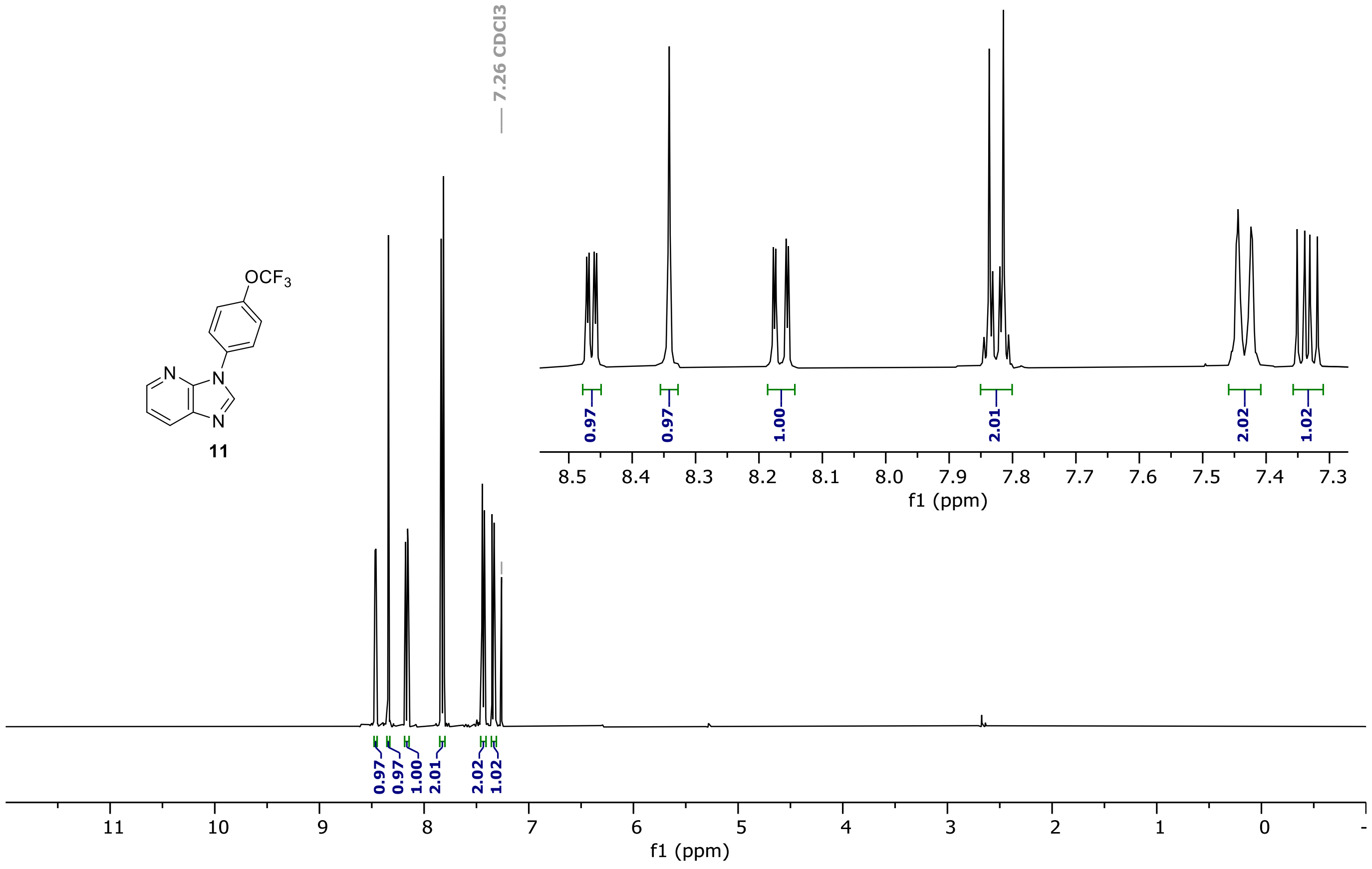

Figure S21. *^{1}H NMR (401 MHz, $CDCl_3$, 298 K) spectrum of* ***11***

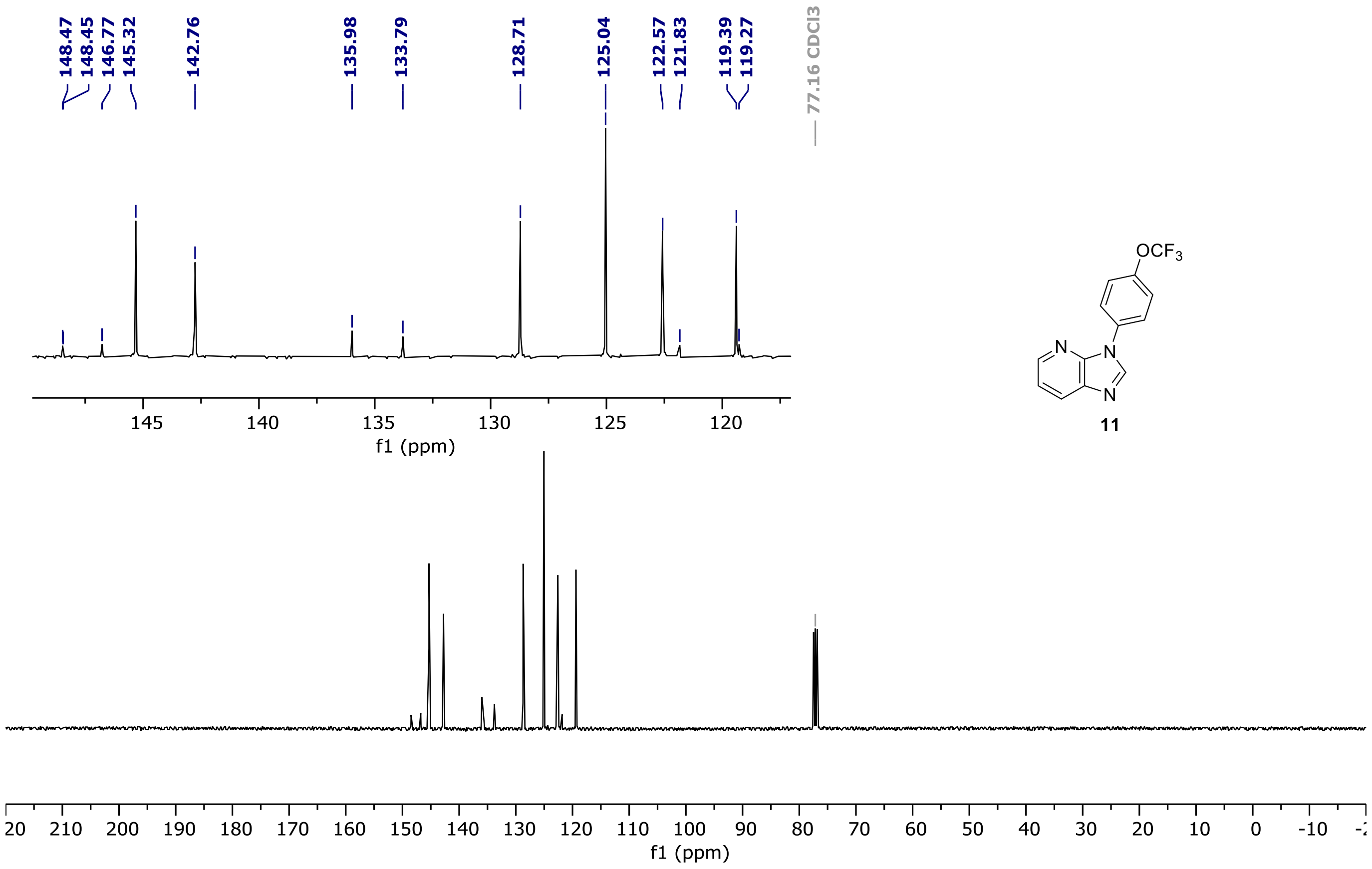

Figure S22. *$^{13}C\{^{1}H\}$ NMR (101 MHz, $CDCl_3$, 298 K) spectrum of* ***11***

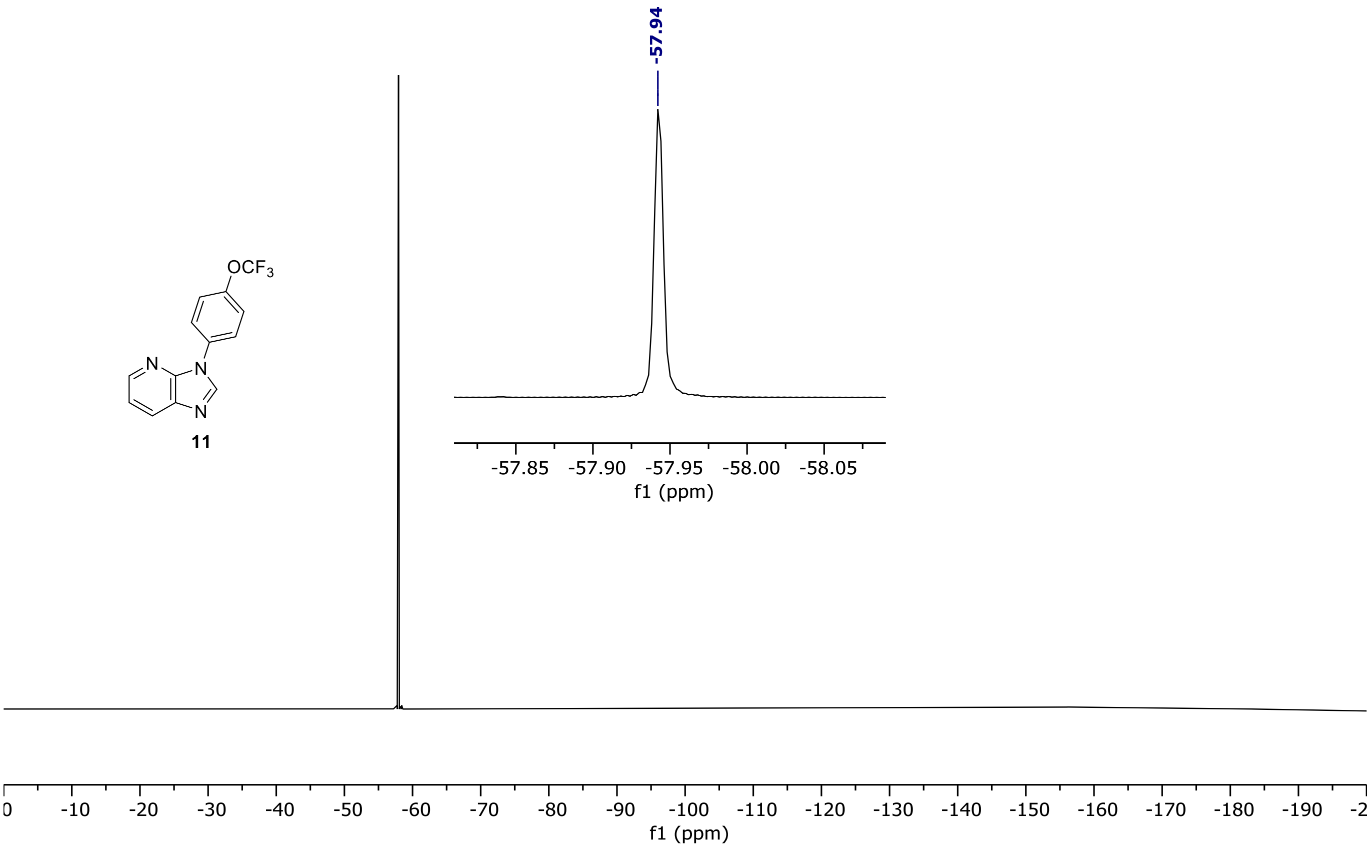

Figure S23. *^{19}F NMR (376 MHz, $CDCl_3$, 298 K) spectrum of* ***11***

^1H and ^{13}C NMR spectra

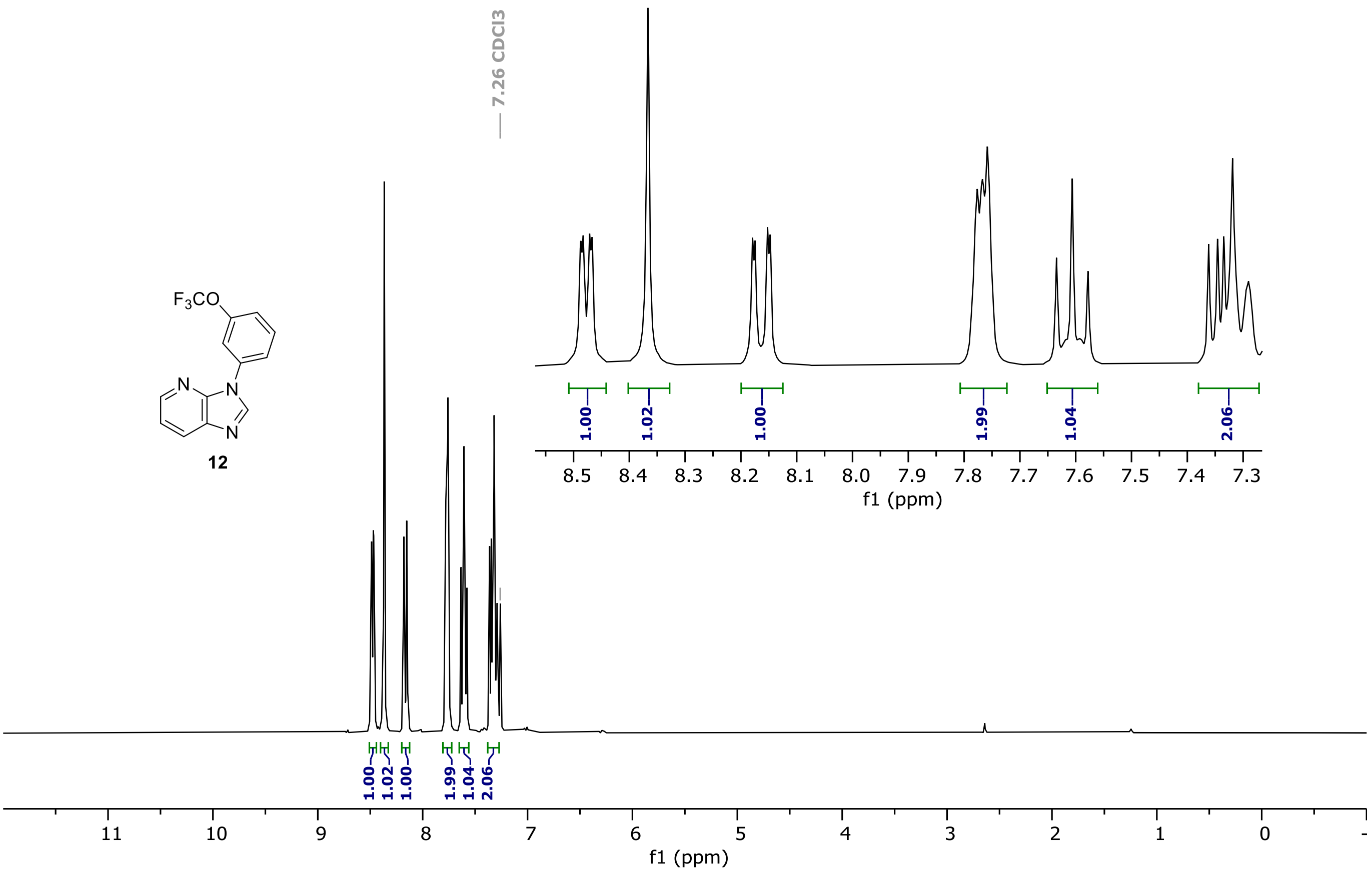

Figure S24. *^1H NMR (300 MHz, $CDCl_3$, 298 K) spectrum of* ***12***

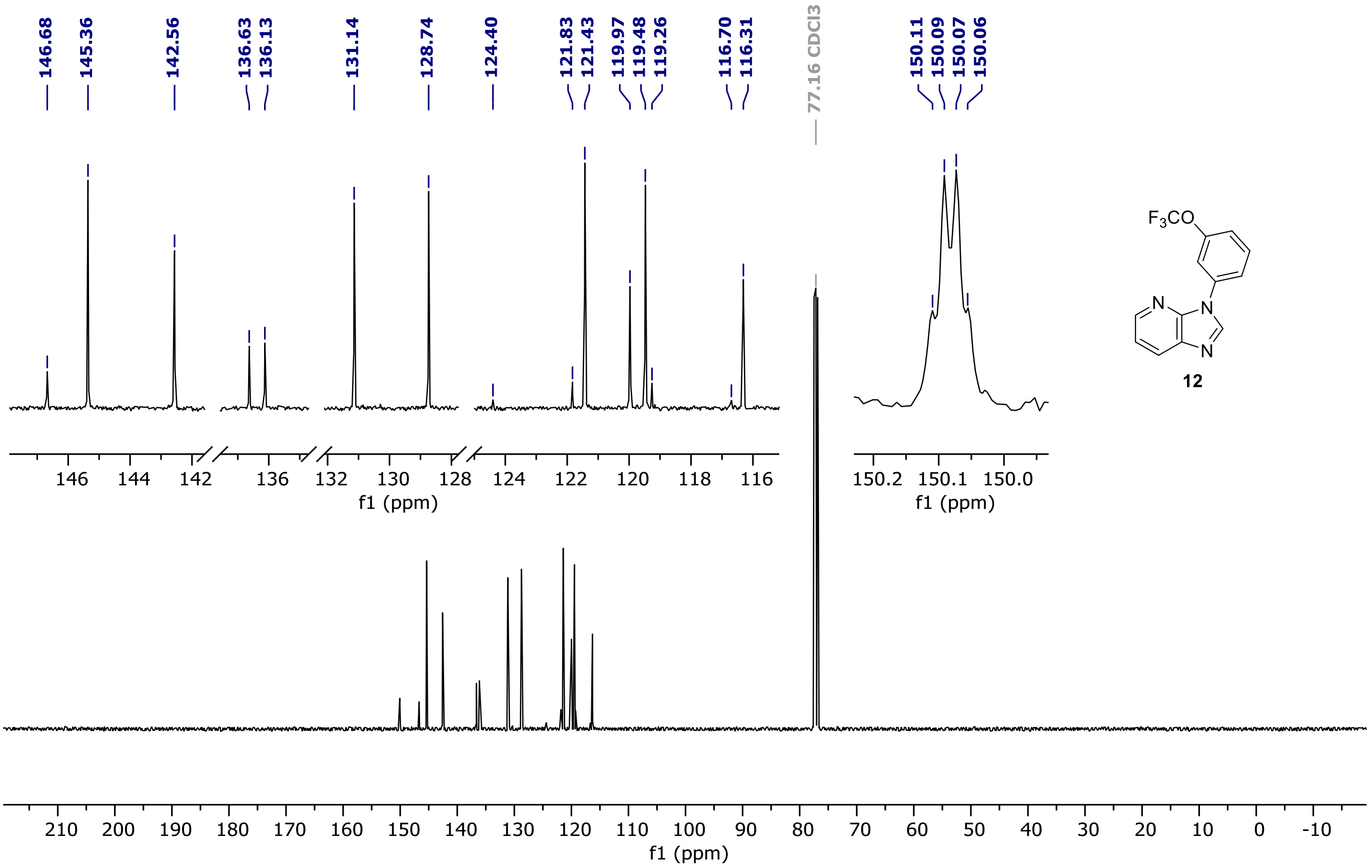

Figure S25. *$^{13}C\{^1H\}$ NMR (101 MHz, $CDCl_3$, 298 K) spectrum of* ***12***

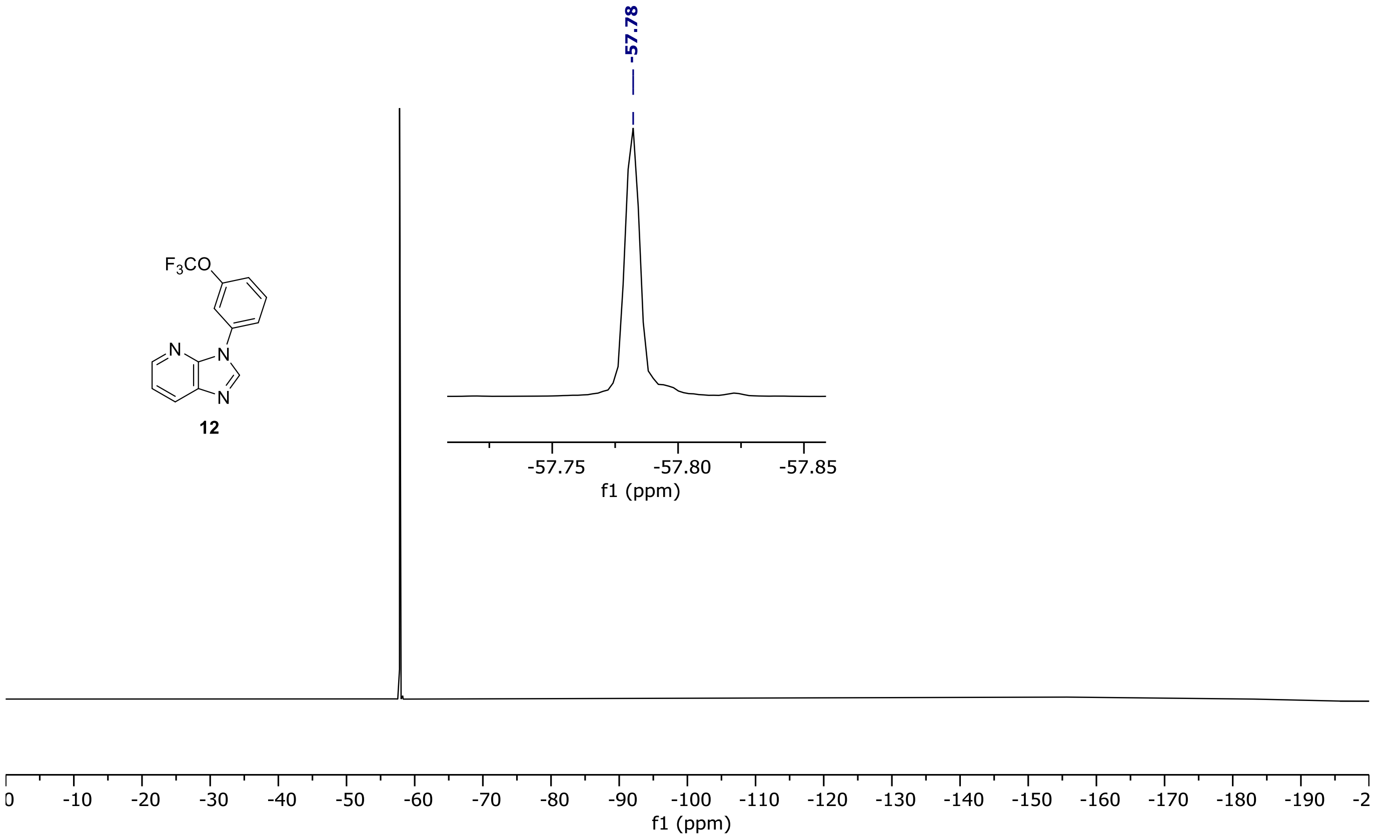

Figure S26. *^{19}F NMR (376 MHz, $CDCl_3$, 298 K) spectrum of **12***

^{1}H and ^{13}C NMR spectra

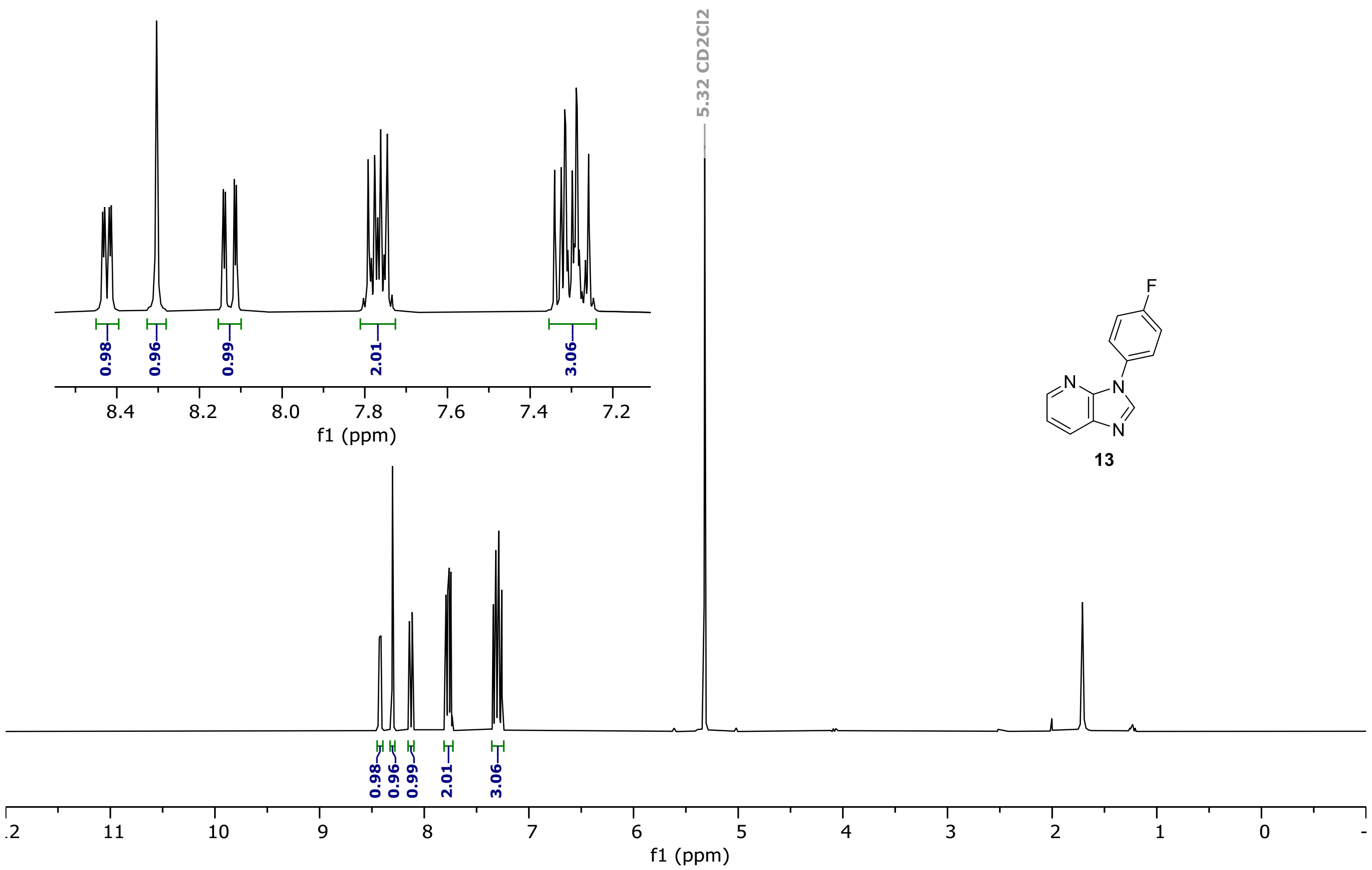

Figure S27. *^{1}H NMR (300 MHz, CD_2Cl_2, 298 K) spectrum of* ***13***

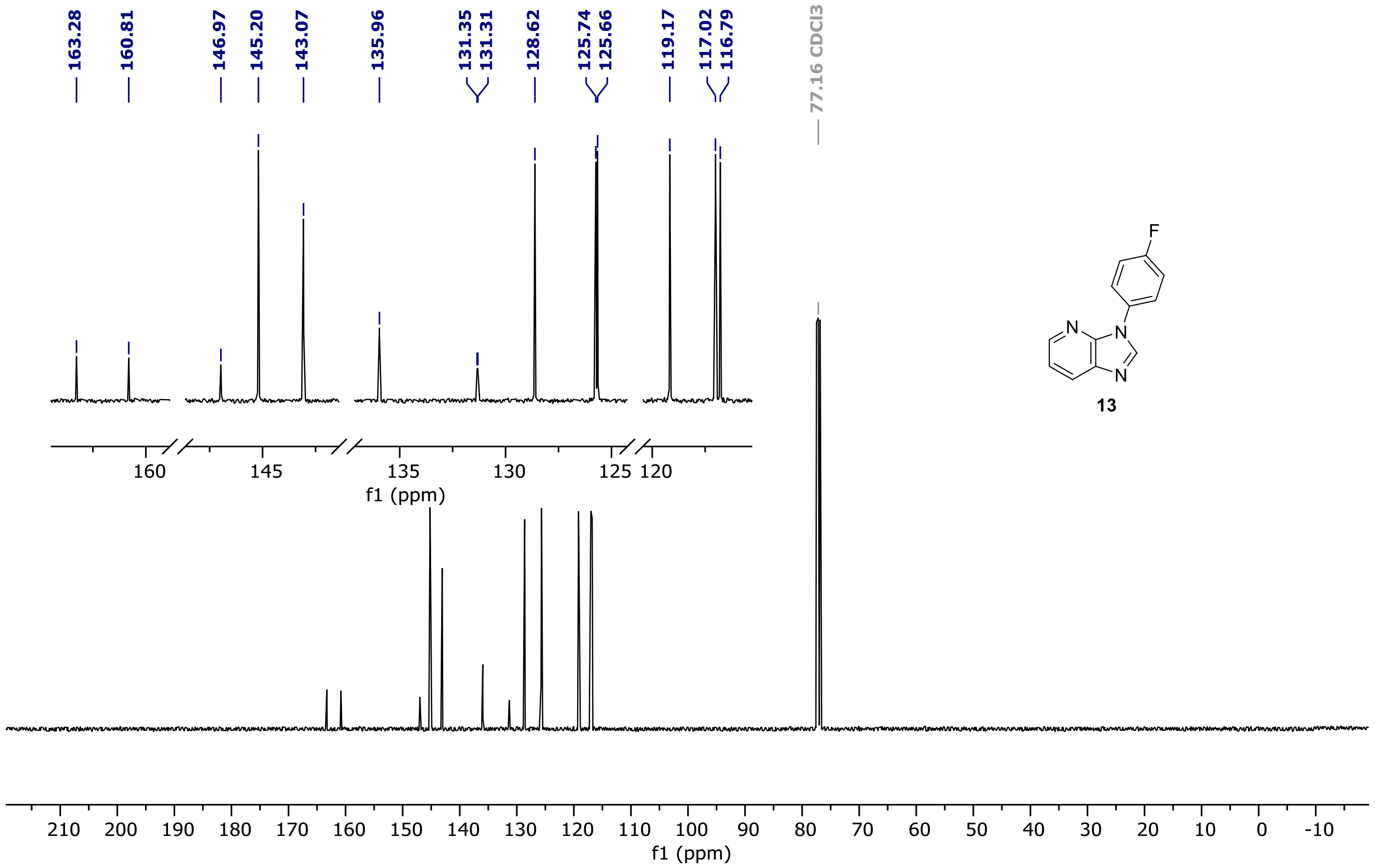

Figure S28. *$^{13}C\{^{1}H\}$ NMR (101 MHz, $CDCl_3$, 298 K) spectrum of* ***13***

^{1}H and ^{13}C NMR spectra

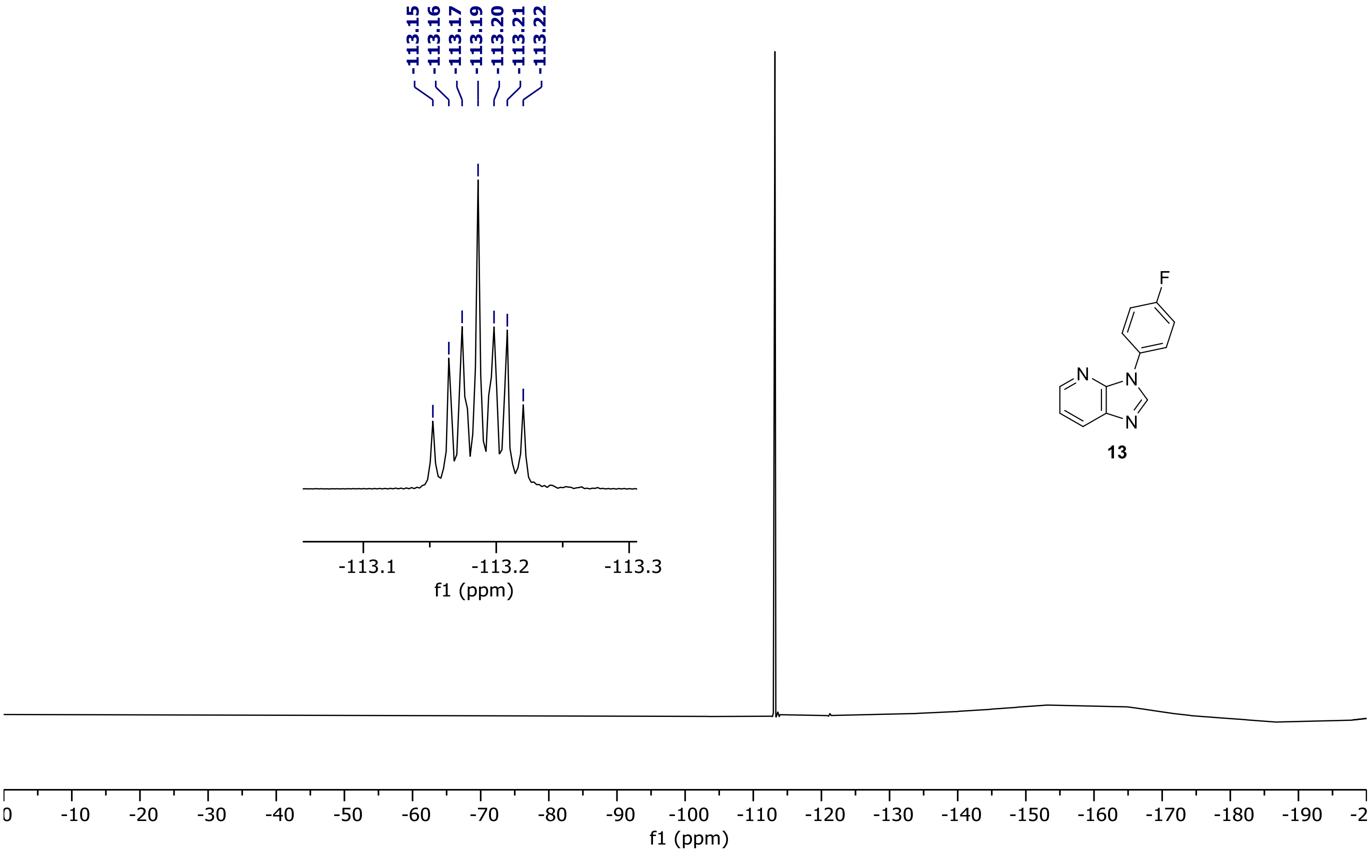

13

Figure S29. *^{19}F NMR (376 MHz, CD_2Cl_2, 298 K) spectrum of* ***13***

^1H and ^{13}C NMR spectra

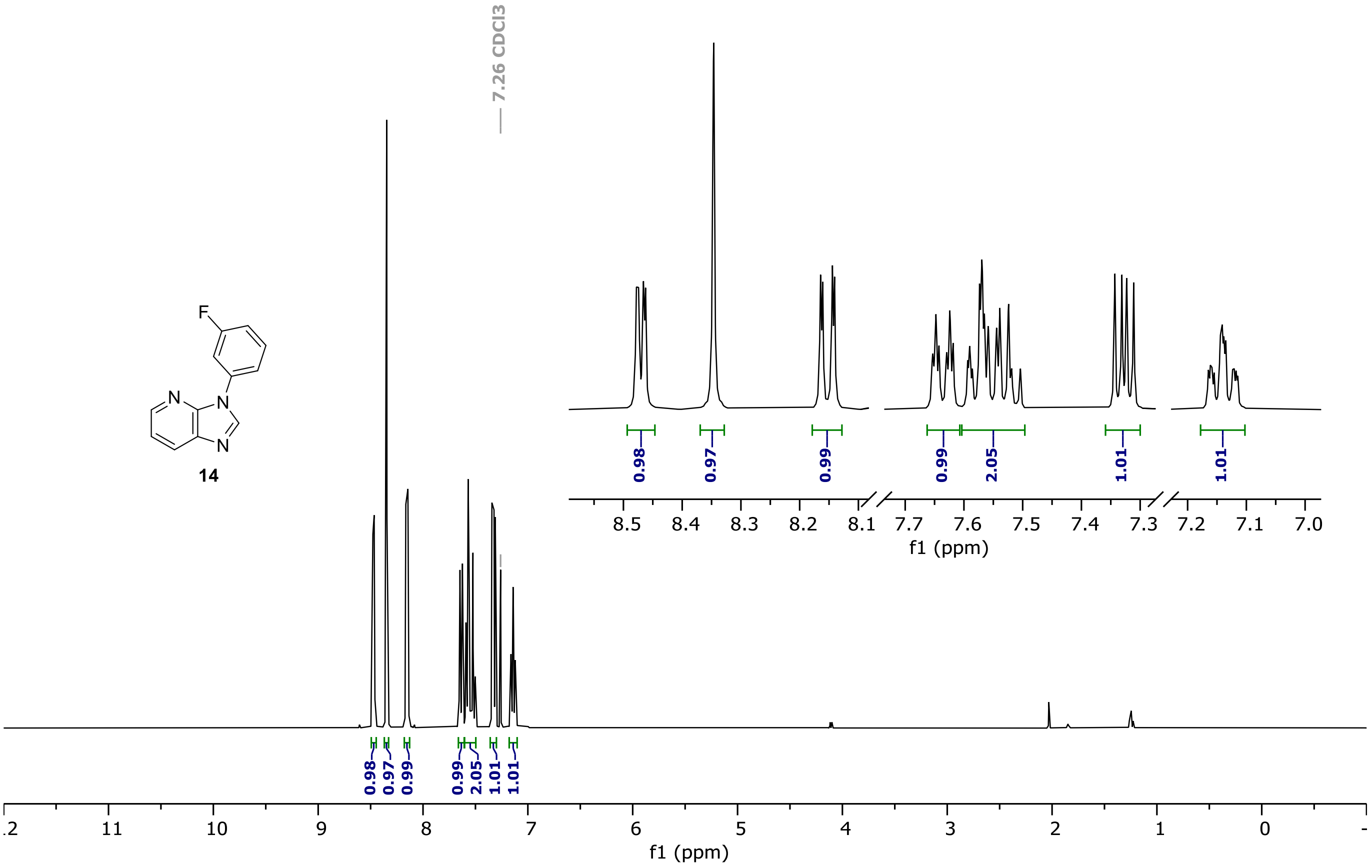

Figure S30. ^1H NMR (300 MHz, $CDCl_3$, 298 K) spectrum of **14**

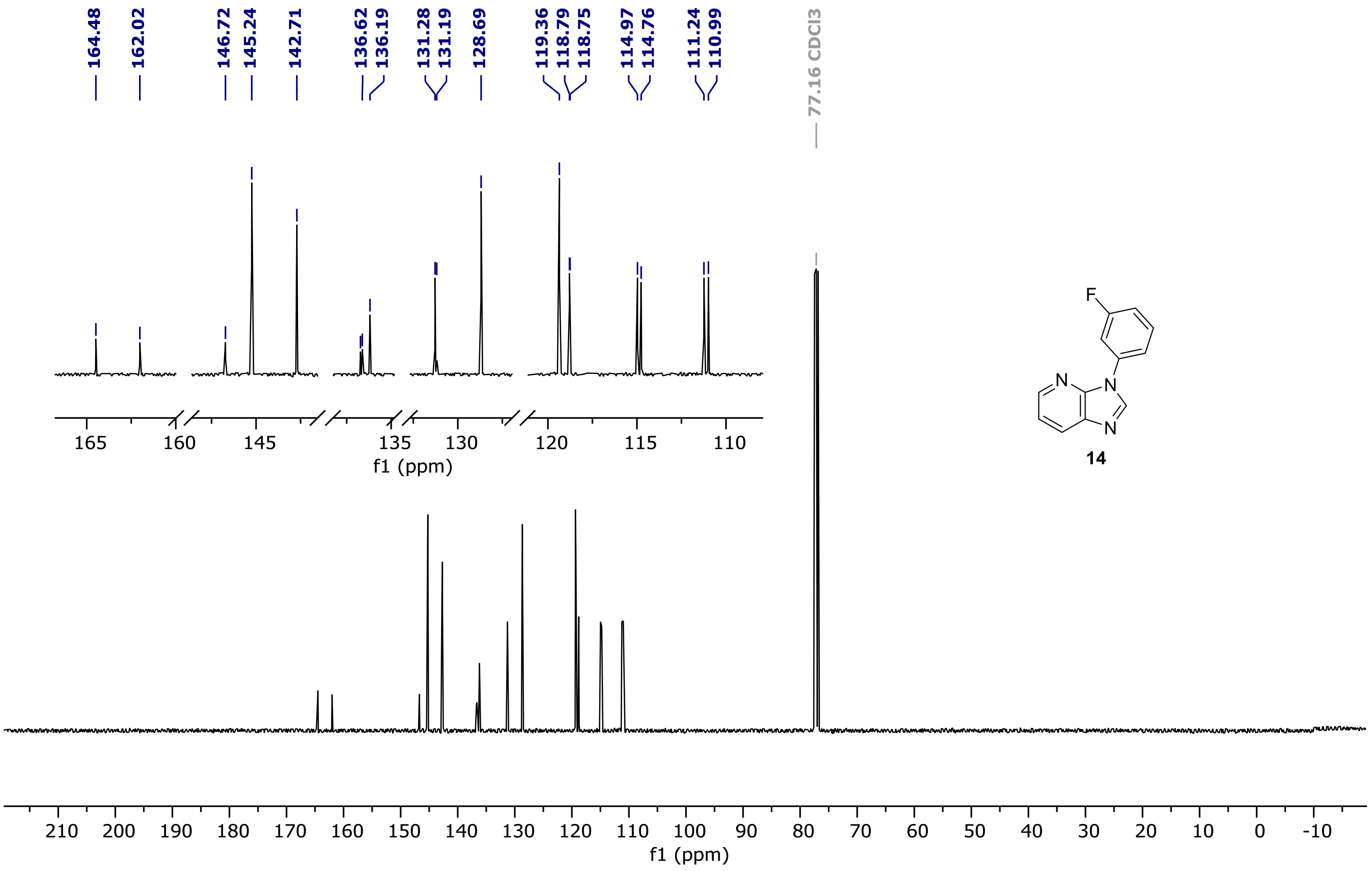

Figure S31. $^{13}C\{^1H\}$ NMR (101 MHz, $CDCl_3$, 298 K) spectrum of **14**

^{1}H and ^{13}C NMR spectra

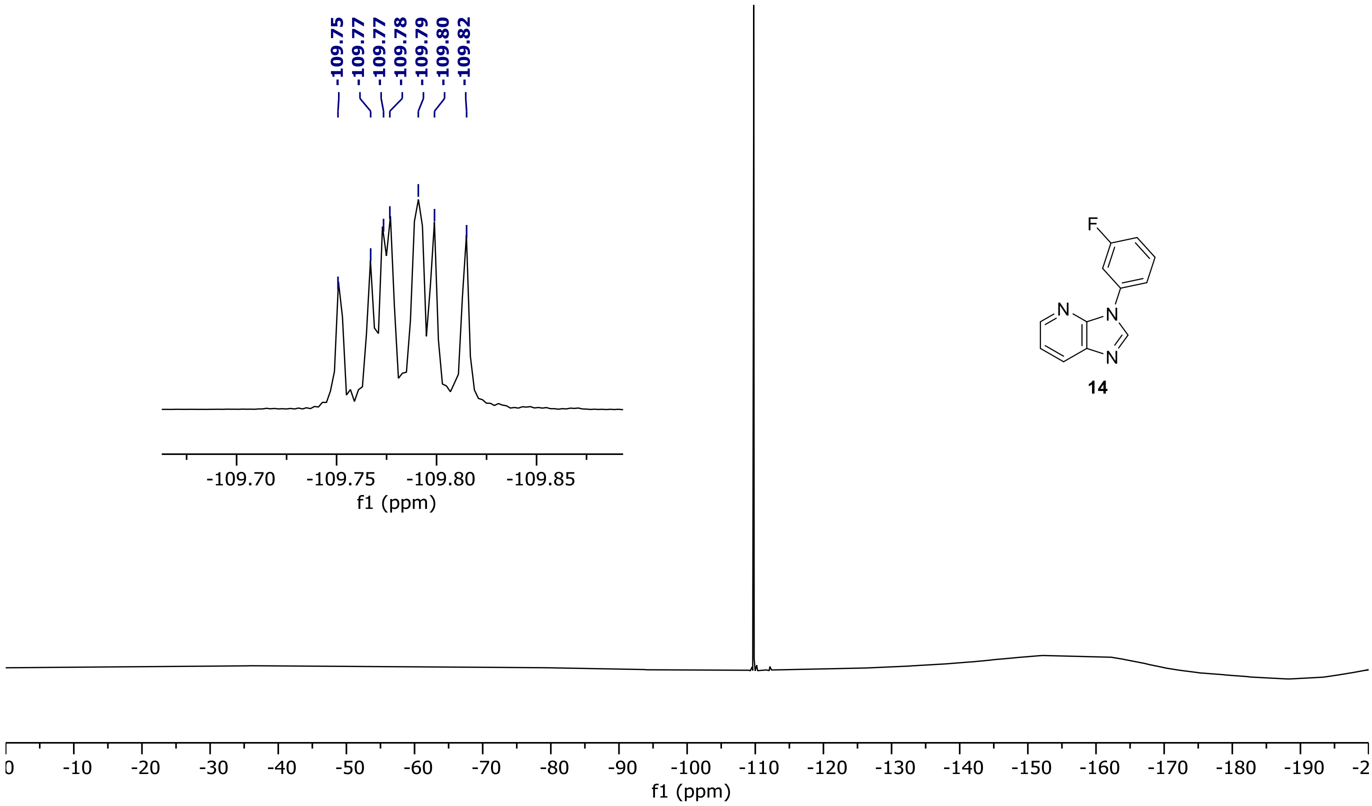

Figure S32. *^{19}F NMR (376 MHz, CD_2Cl_2, 298 K) spectrum of* ***14***

^{1}H and ^{13}C NMR spectra

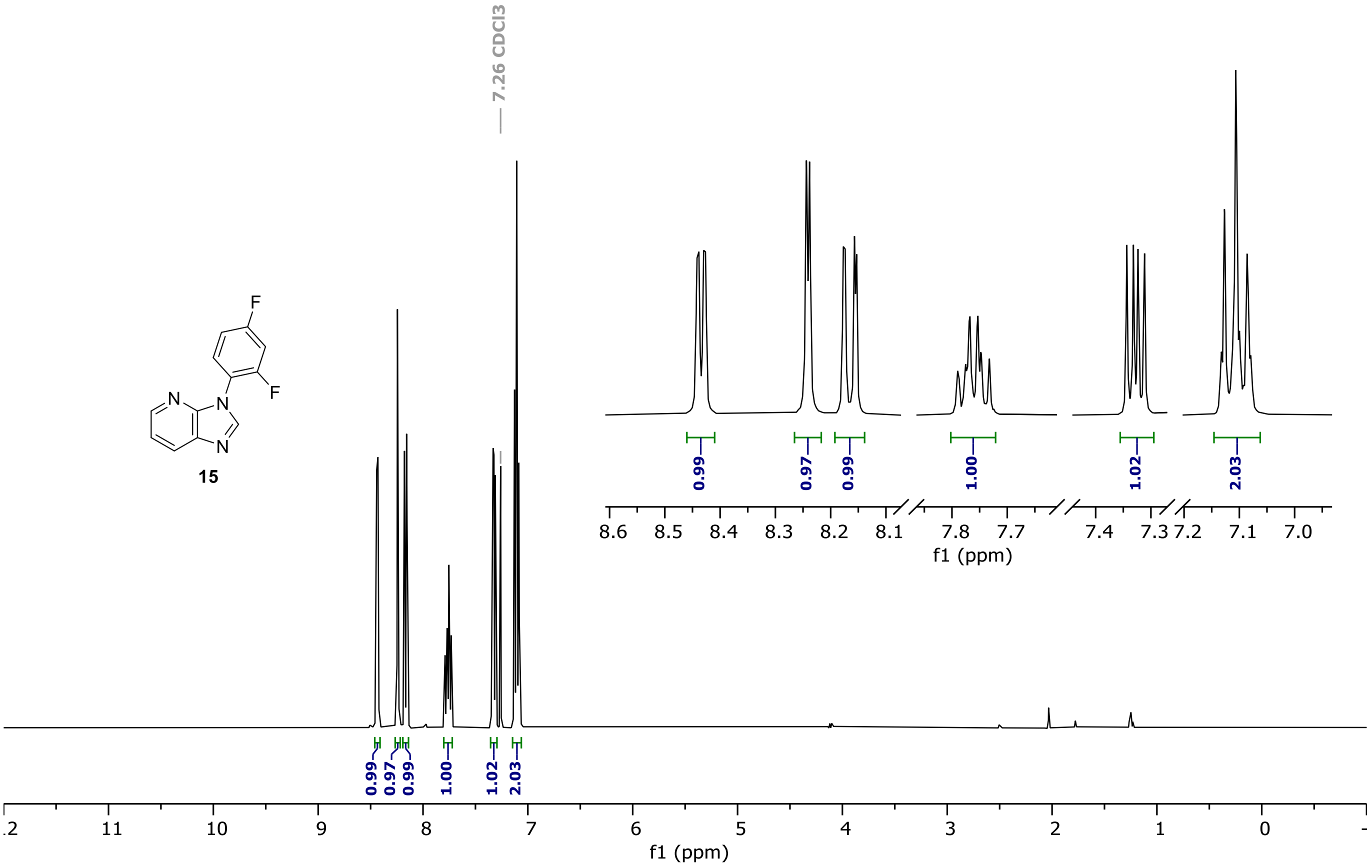

Figure S33. *^{1}H NMR (400 MHz, $CDCl_3$, 298 K) spectrum of **15***

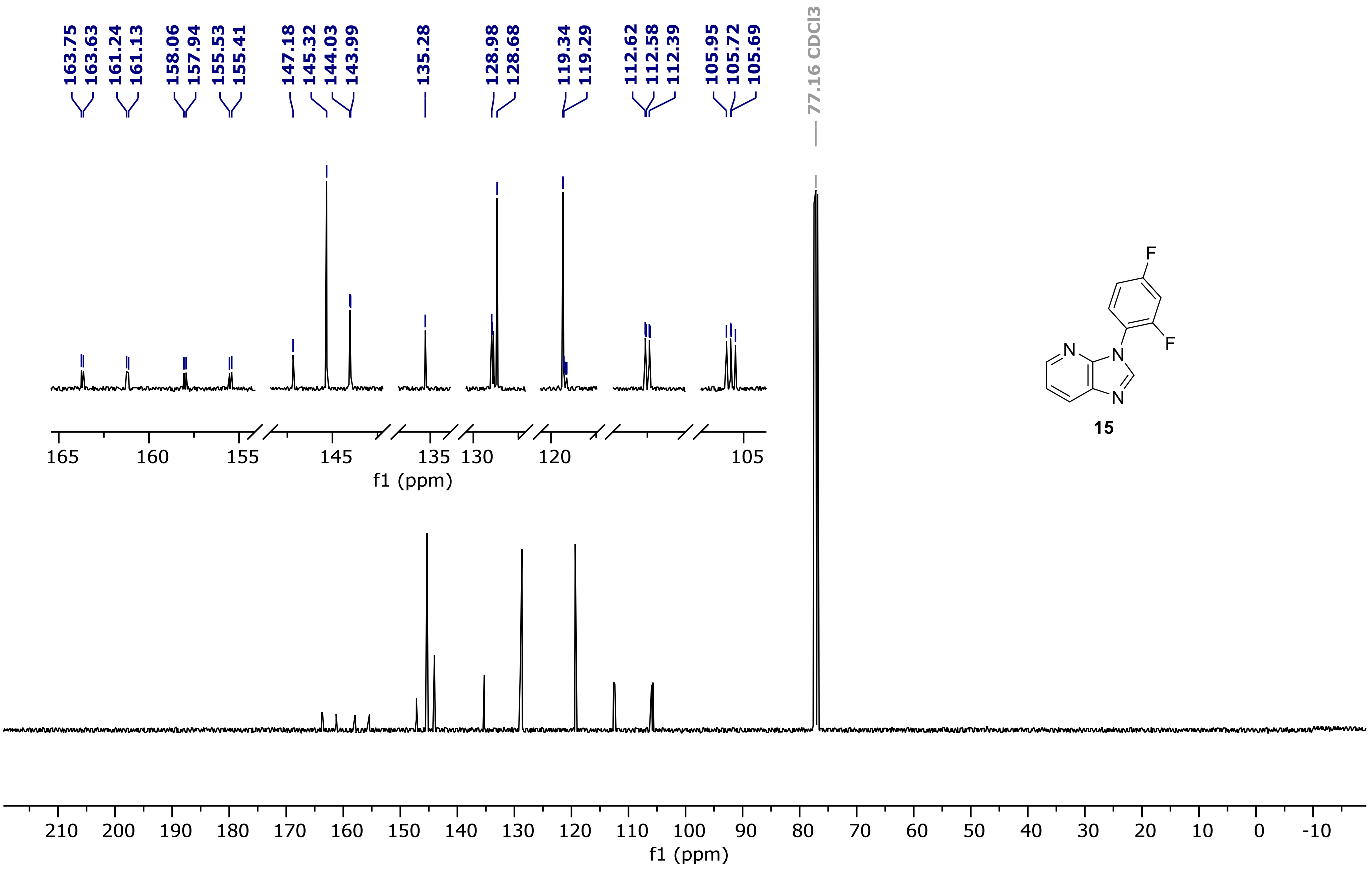

Figure S34. *$^{13}C\{^{1}H\}$ NMR (101 MHz, $CDCl_3$, 298 K) spectrum of **15***

^{1}H and ^{13}C NMR spectra

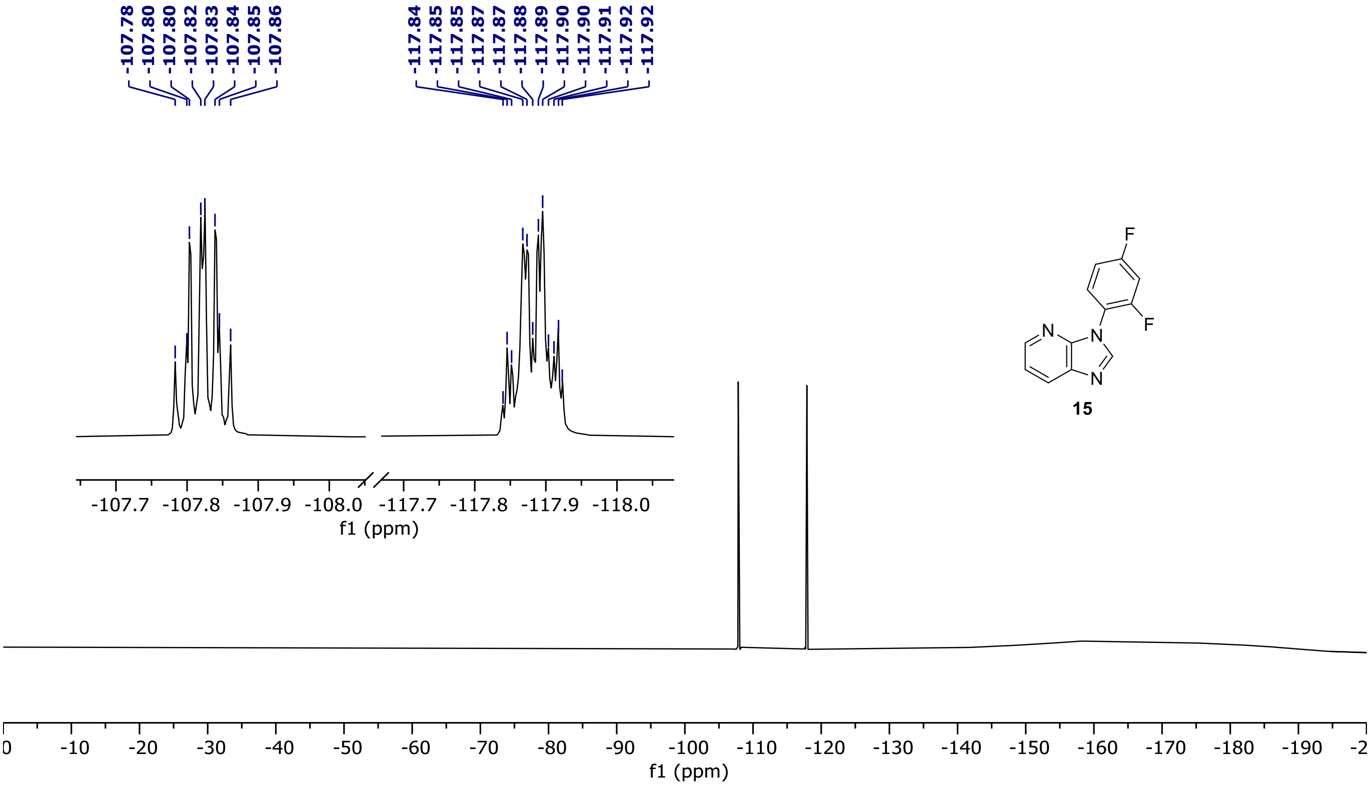

Figure S35. *^{19}F NMR (376 MHz, $CDCl_3$, 298 K) spectrum of* ***15***

^1H and ^{13}C NMR spectra

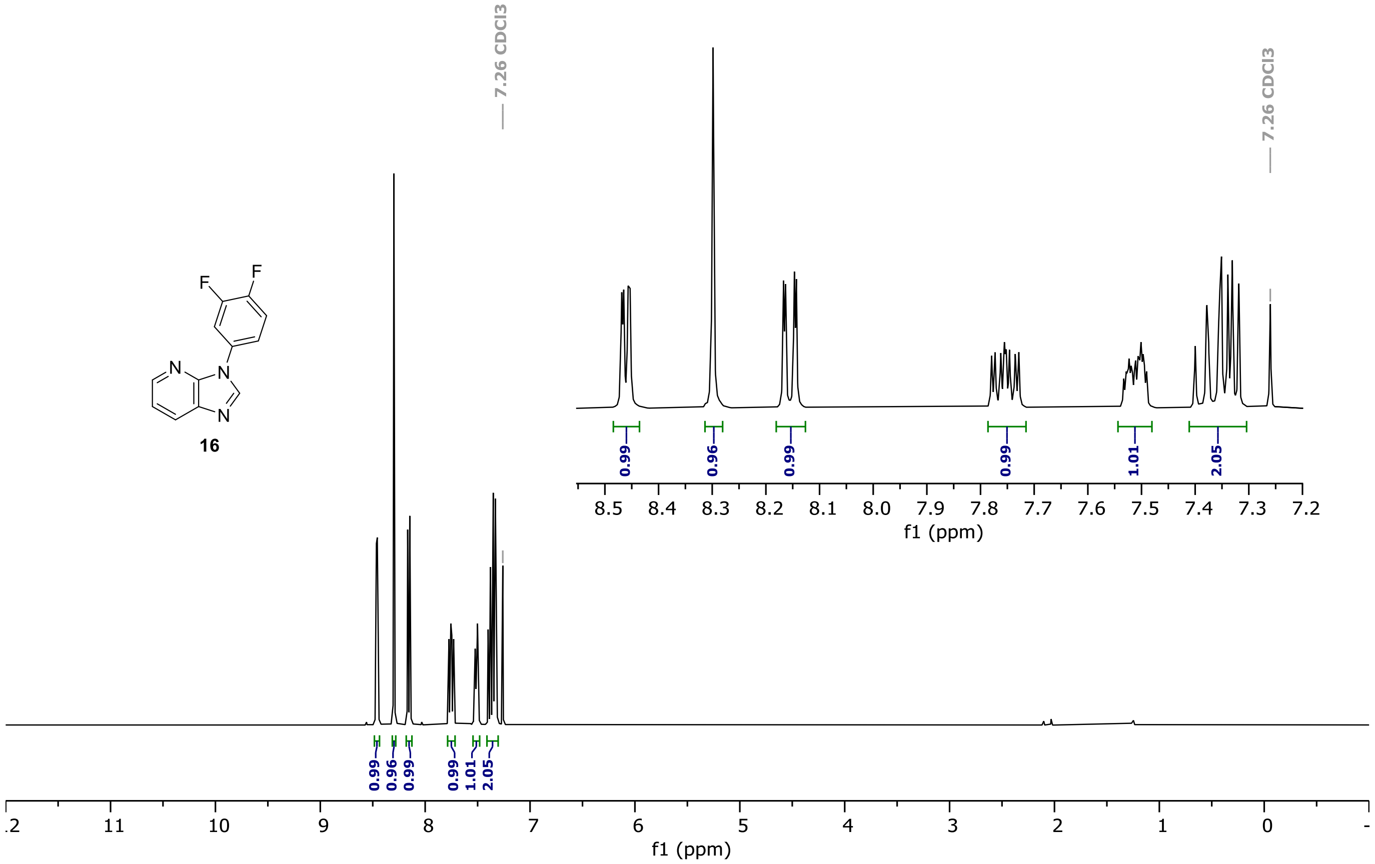

Figure SS36. *^1H NMR (300 MHz, $CDCl_3$, 298 K) spectrum of* ***16***

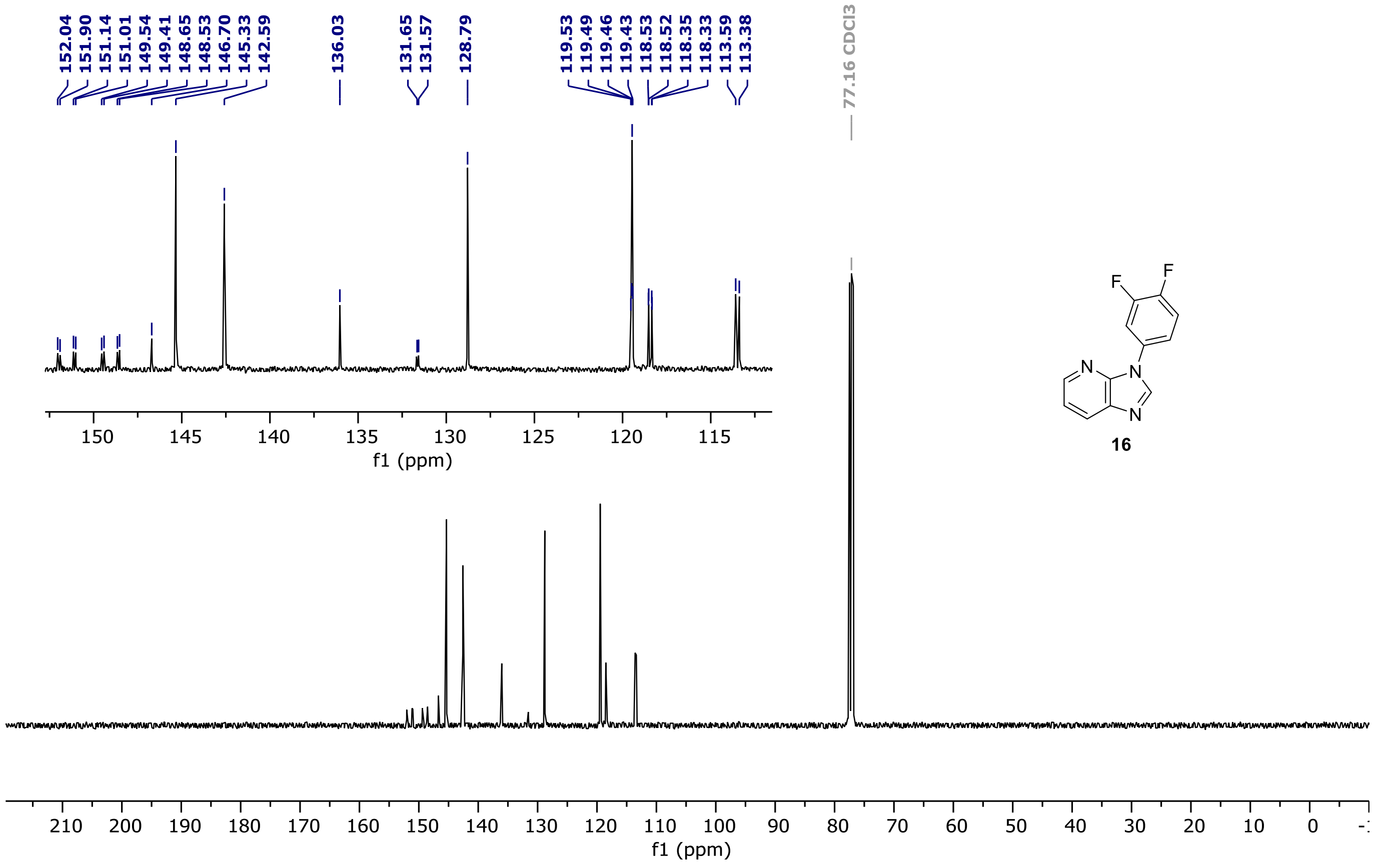

Figure SS37. *$^{13}C\{^1H\}$ NMR (75 MHz, $CDCl_3$, 298 K) spectrum of* ***16***

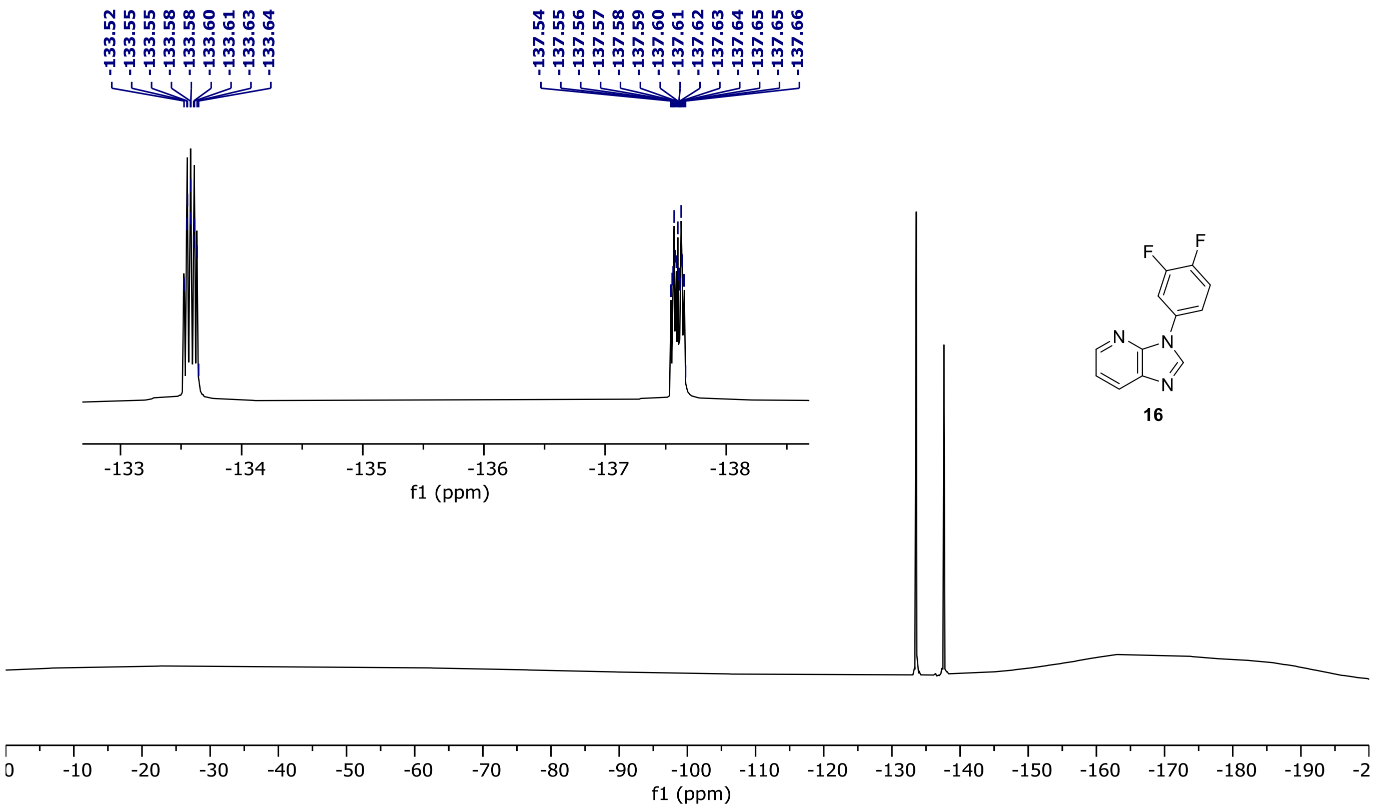

Figure S38. *^{19}F NMR (376 MHz, $CDCl_3$, 298 K) spectrum of **16***

^{1}H and ^{13}C NMR spectra

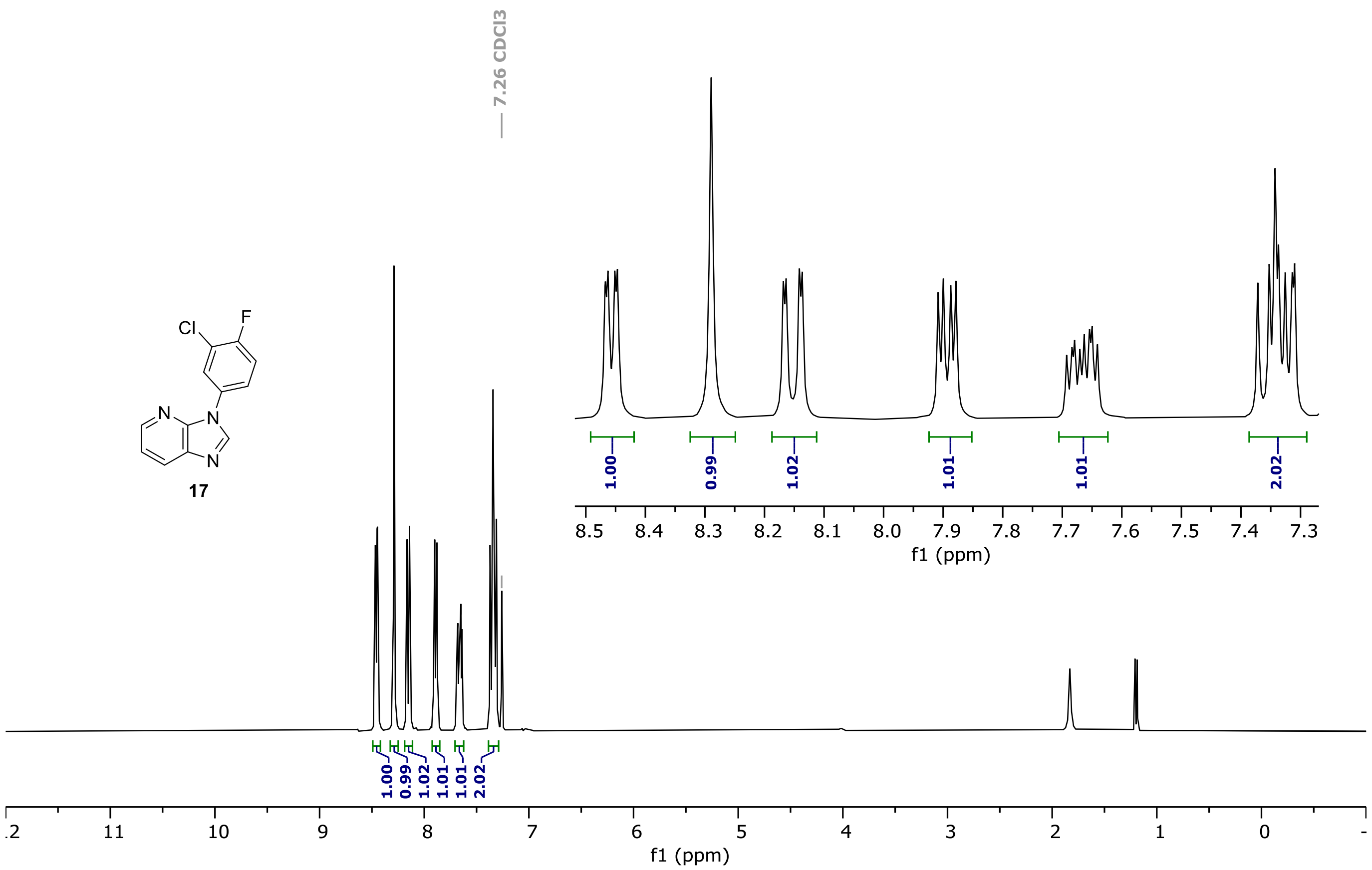

Figure S39. *^{1}H NMR (300 MHz, $CDCl_3$, 298 K) spectrum of **17***

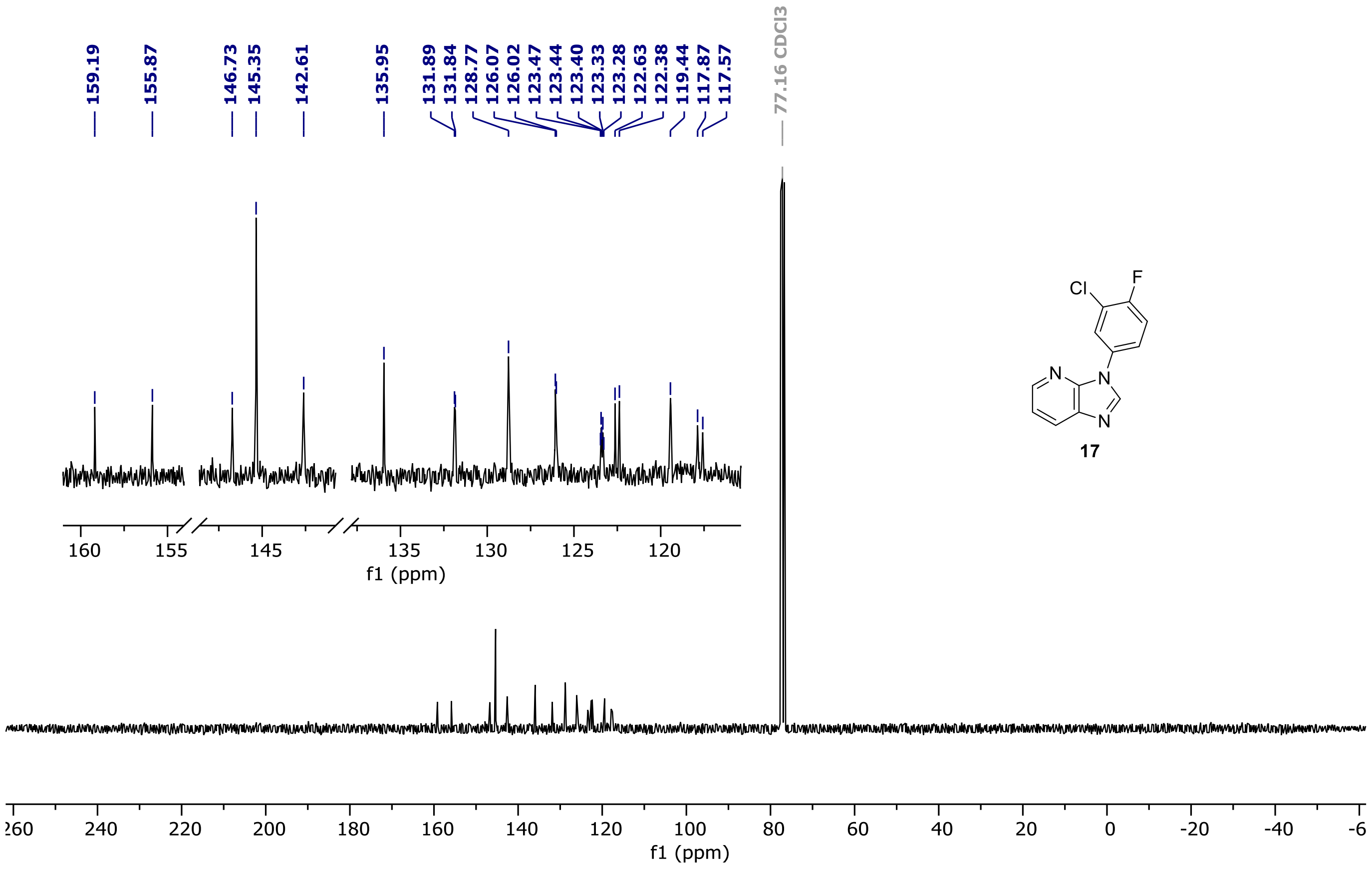

Figure S40. *$^{13}C\{^{1}H\}$ NMR (75 MHz, $CDCl_3$, 298 K) spectrum of **17***

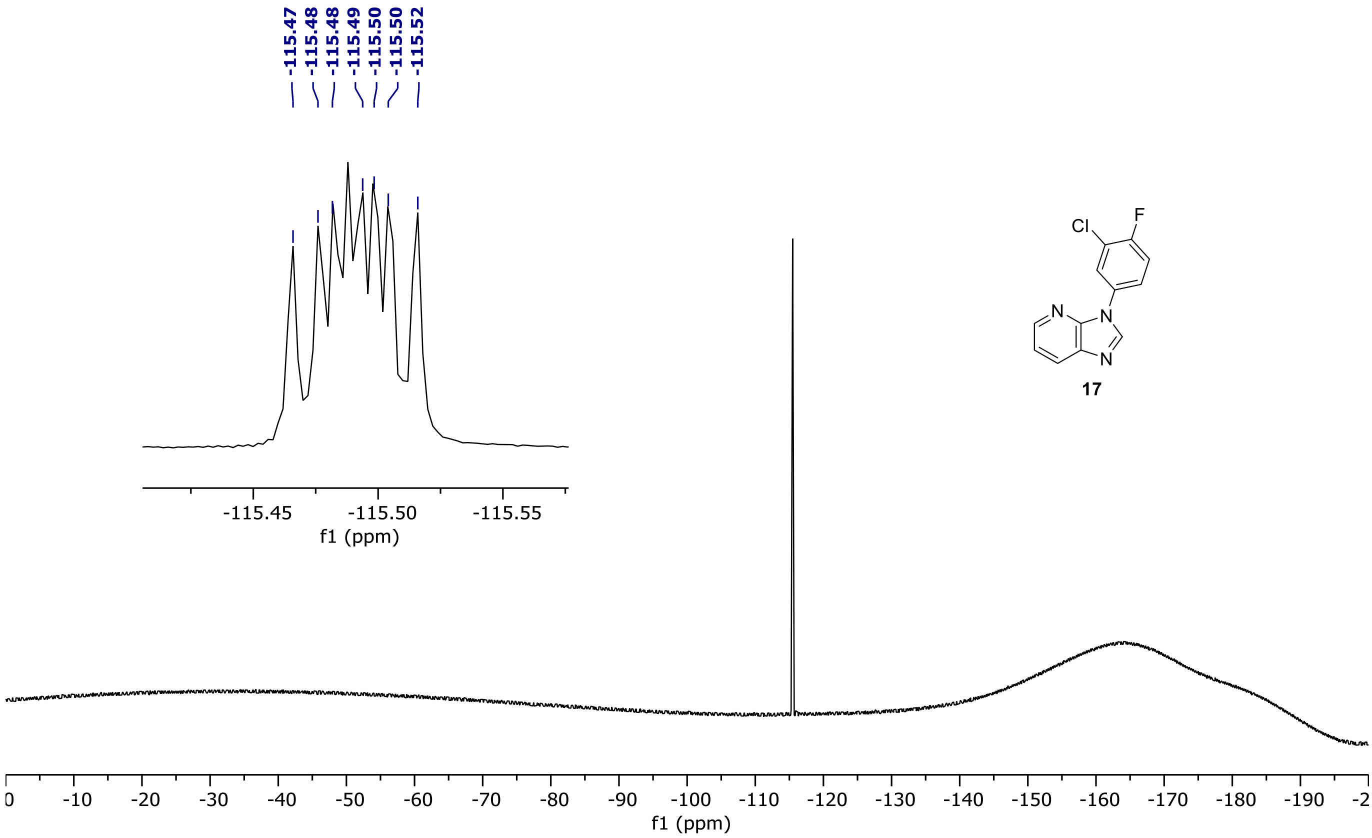

Figure S41. *^{19}F NMR (376 MHz, $CDCl_3$, 298 K) spectrum of* ***17***

^{1}H and ^{13}C NMR spectra

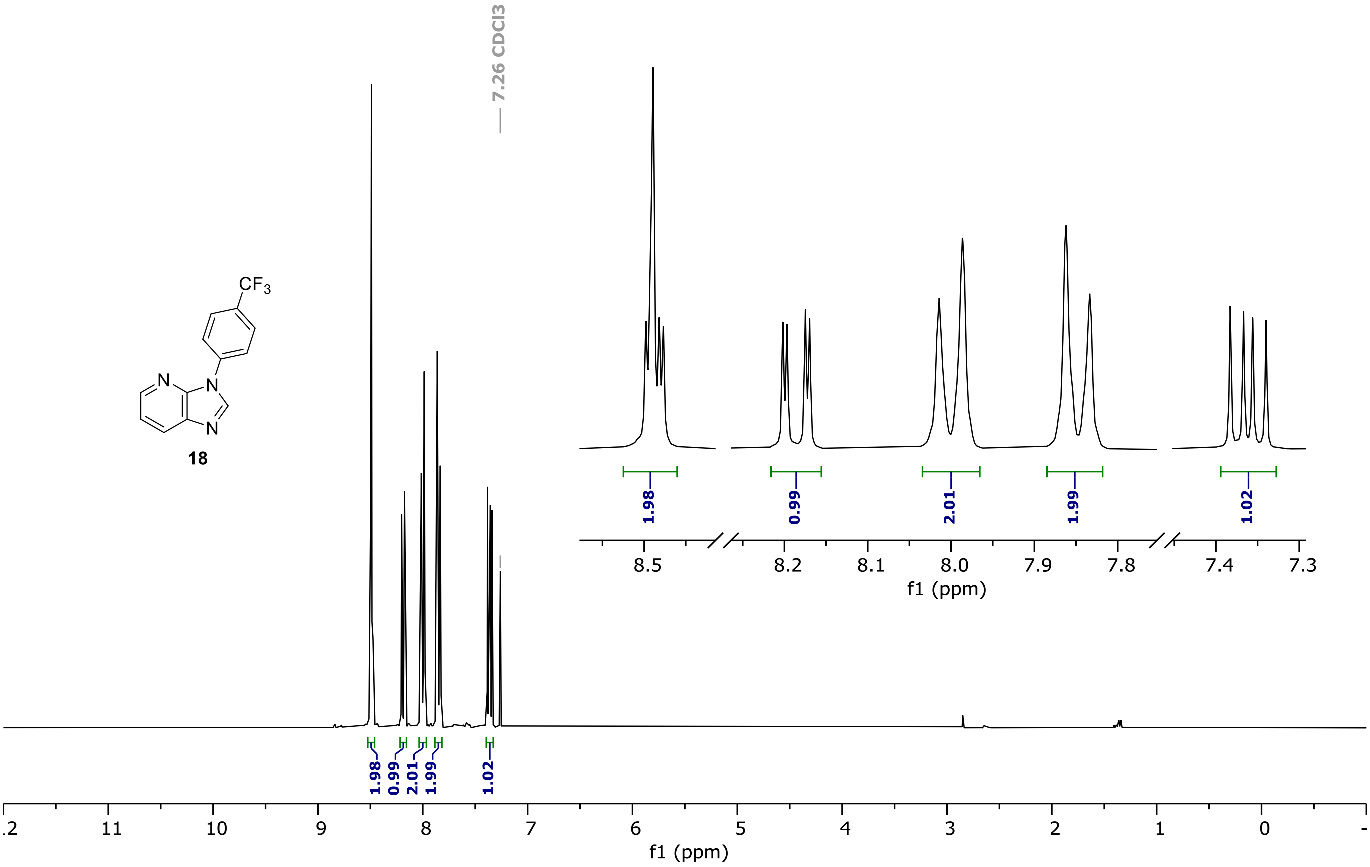

Figure S42. ^{1}H NMR (300 MHz, $CDCl_3$, 298 K) spectrum of **18**

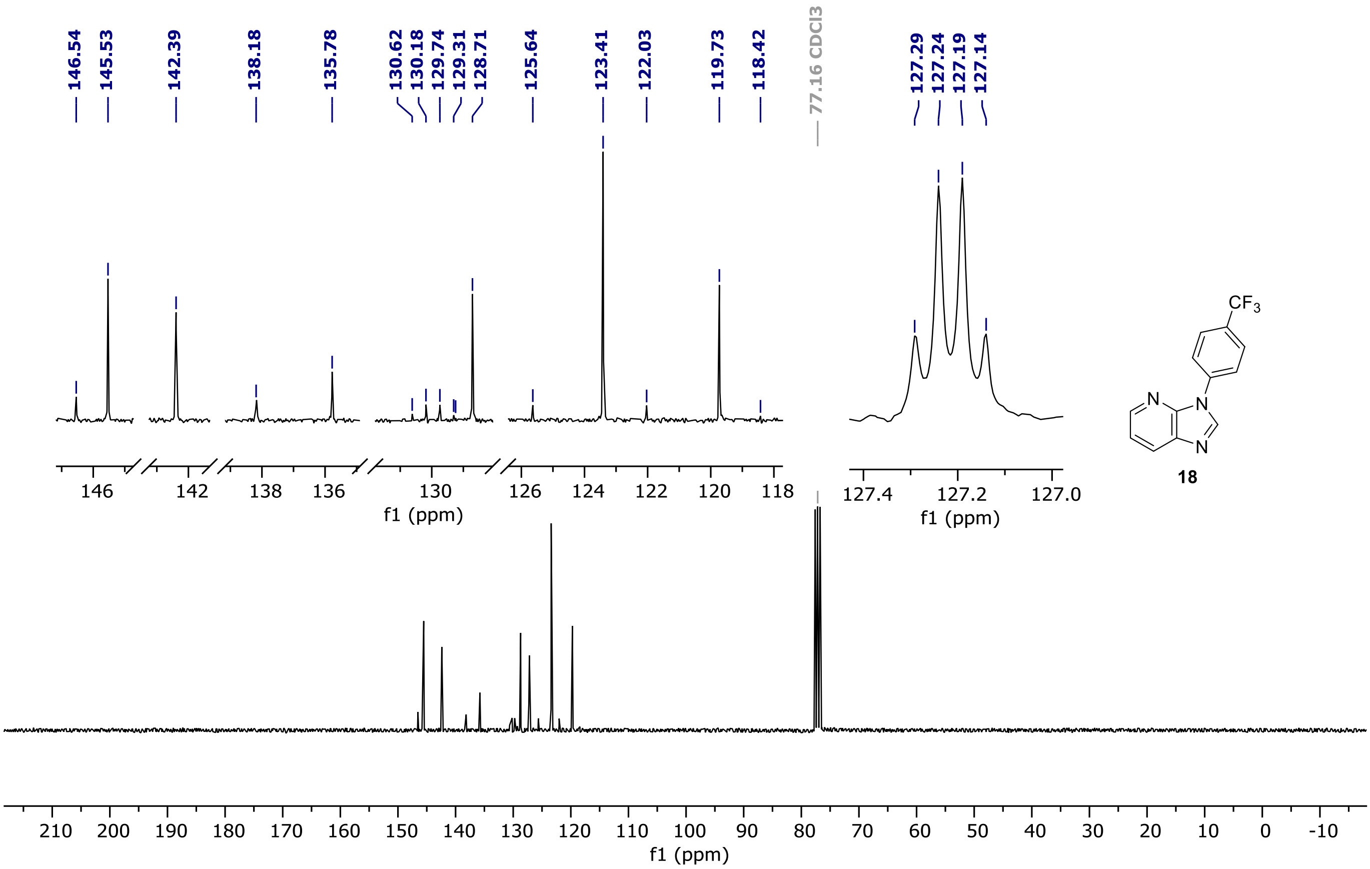

Figure S43. $^{13}C\{^{1}H\}$ NMR (75 MHz, $CDCl_3$, 298 K) spectrum of **18**

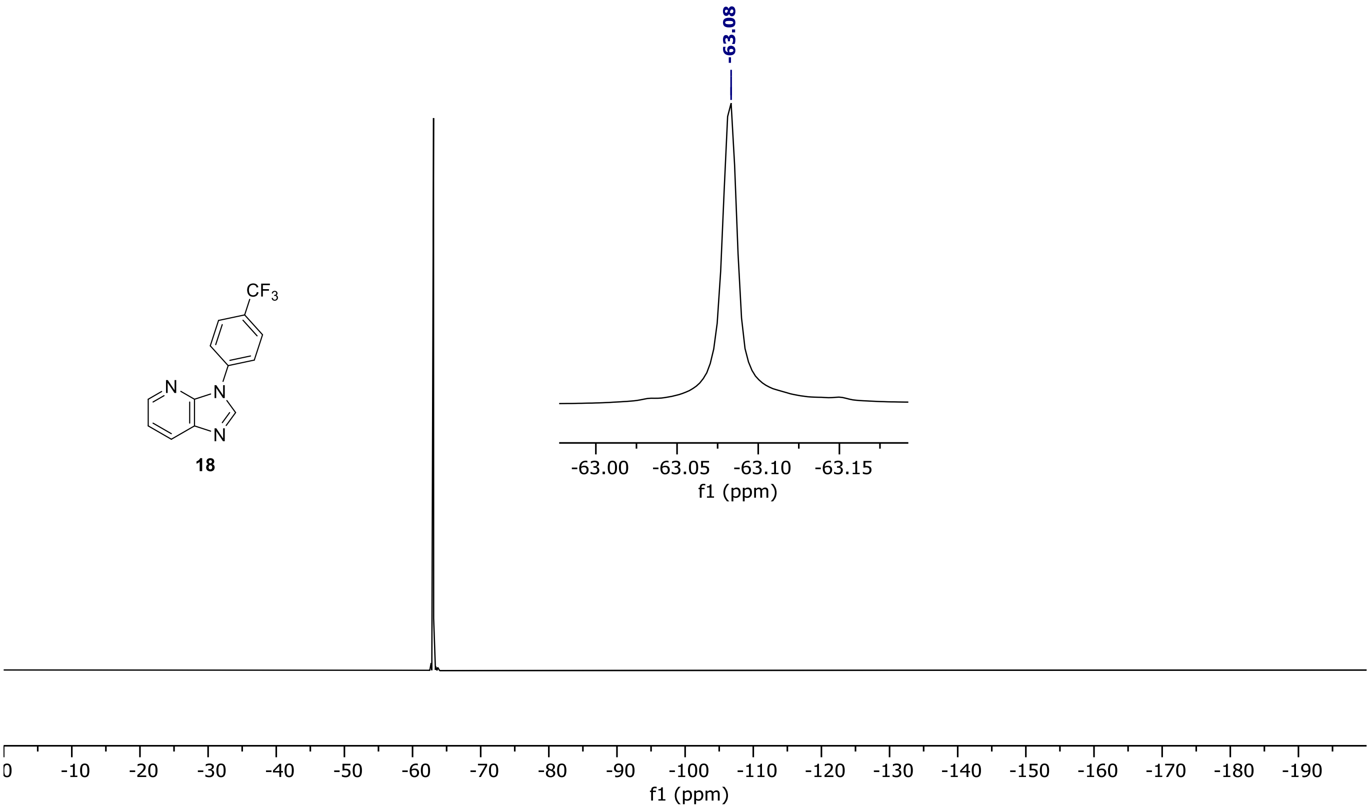

Figure S44. *^{19}F NMR (376 MHz, $CDCl_3$, 298 K) spectrum of* ***18***

^{1}H and ^{13}C NMR spectra

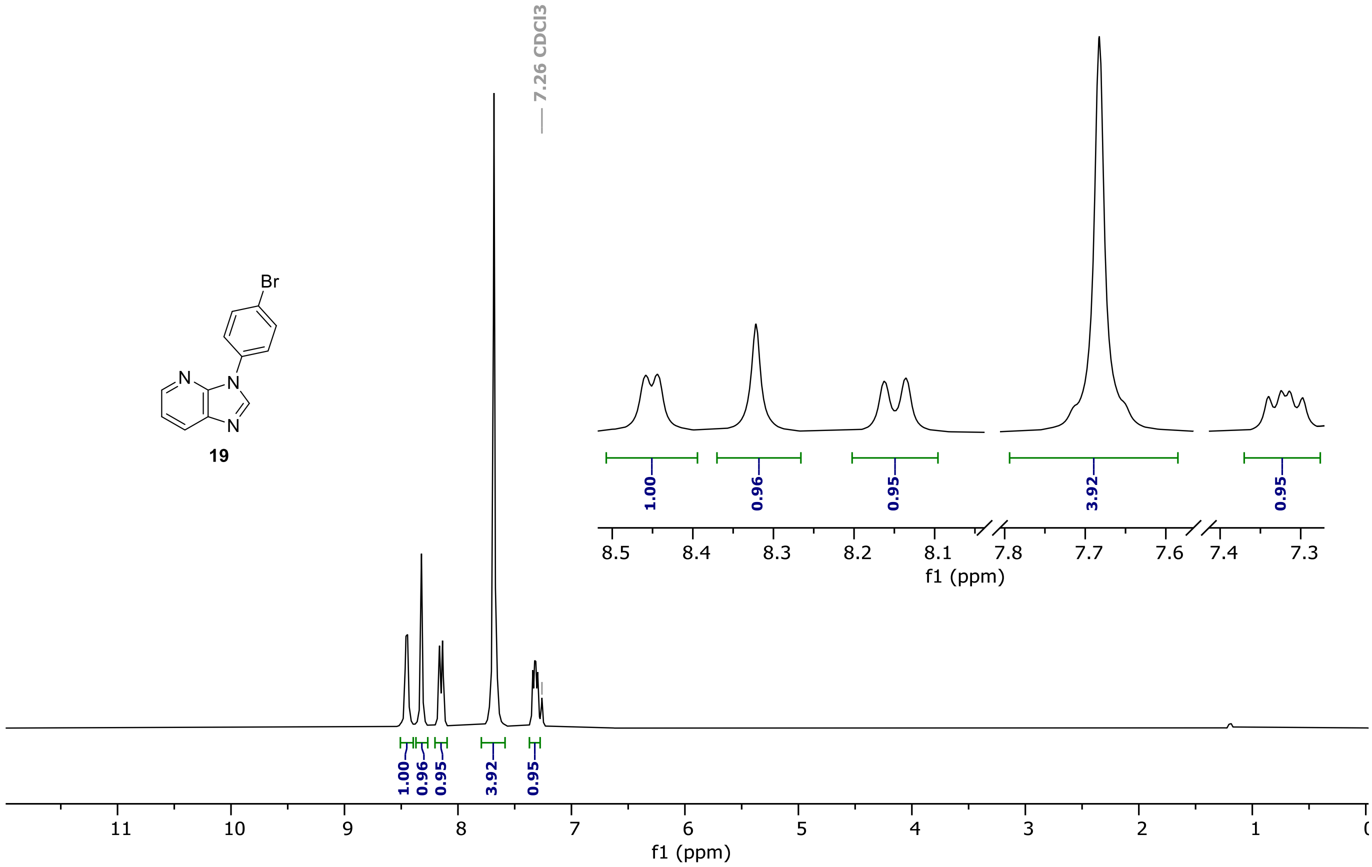

Figure S45. *^{1}H NMR (300 MHz, $CDCl_3$, 298 K) spectrum of **19***

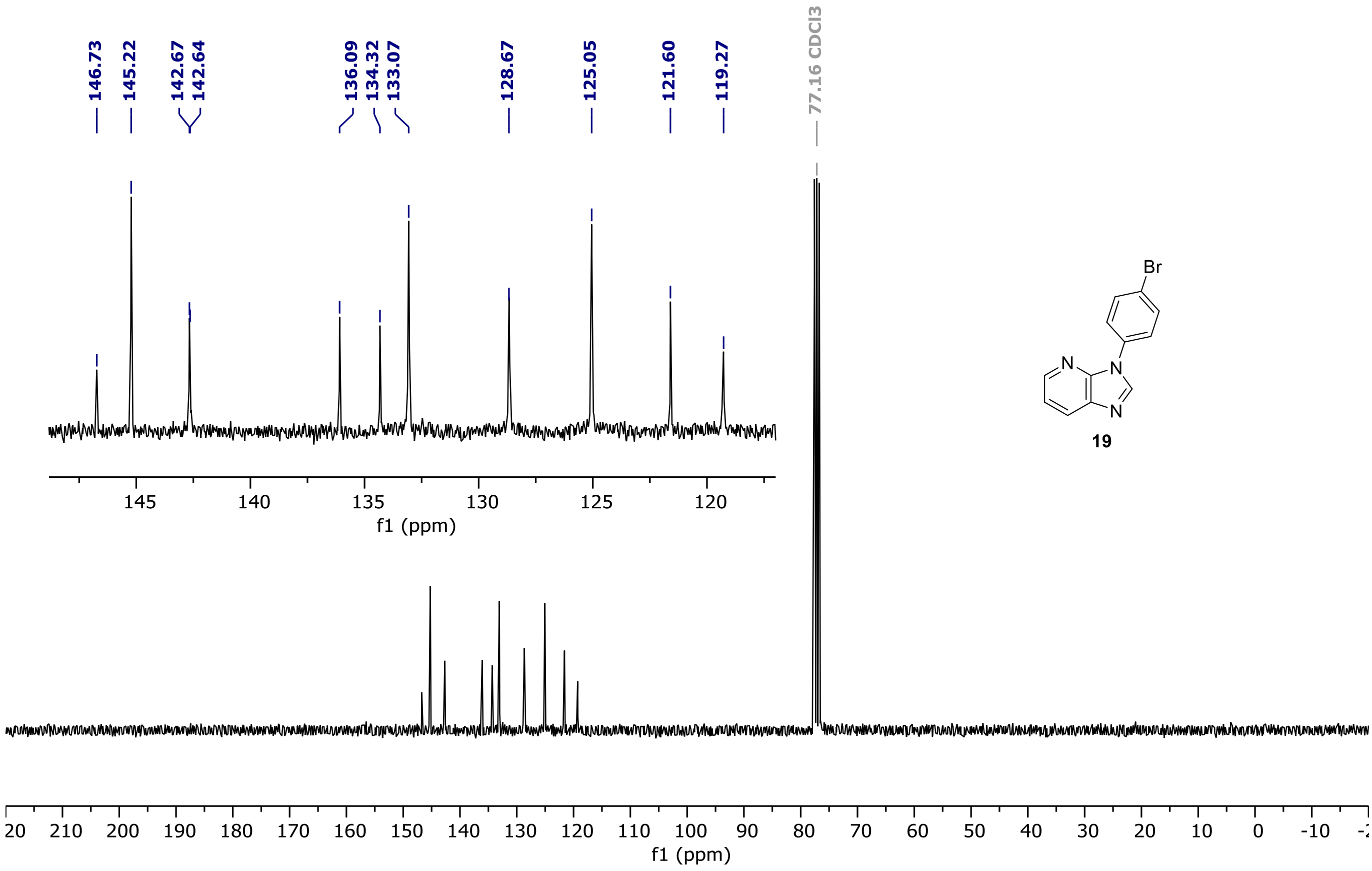

Figure S46. *$^{13}C\{^{1}H\}$ NMR (75 MHz, $CDCl_3$, 298 K) spectrum of **19***

^{1}H and ^{13}C NMR spectra

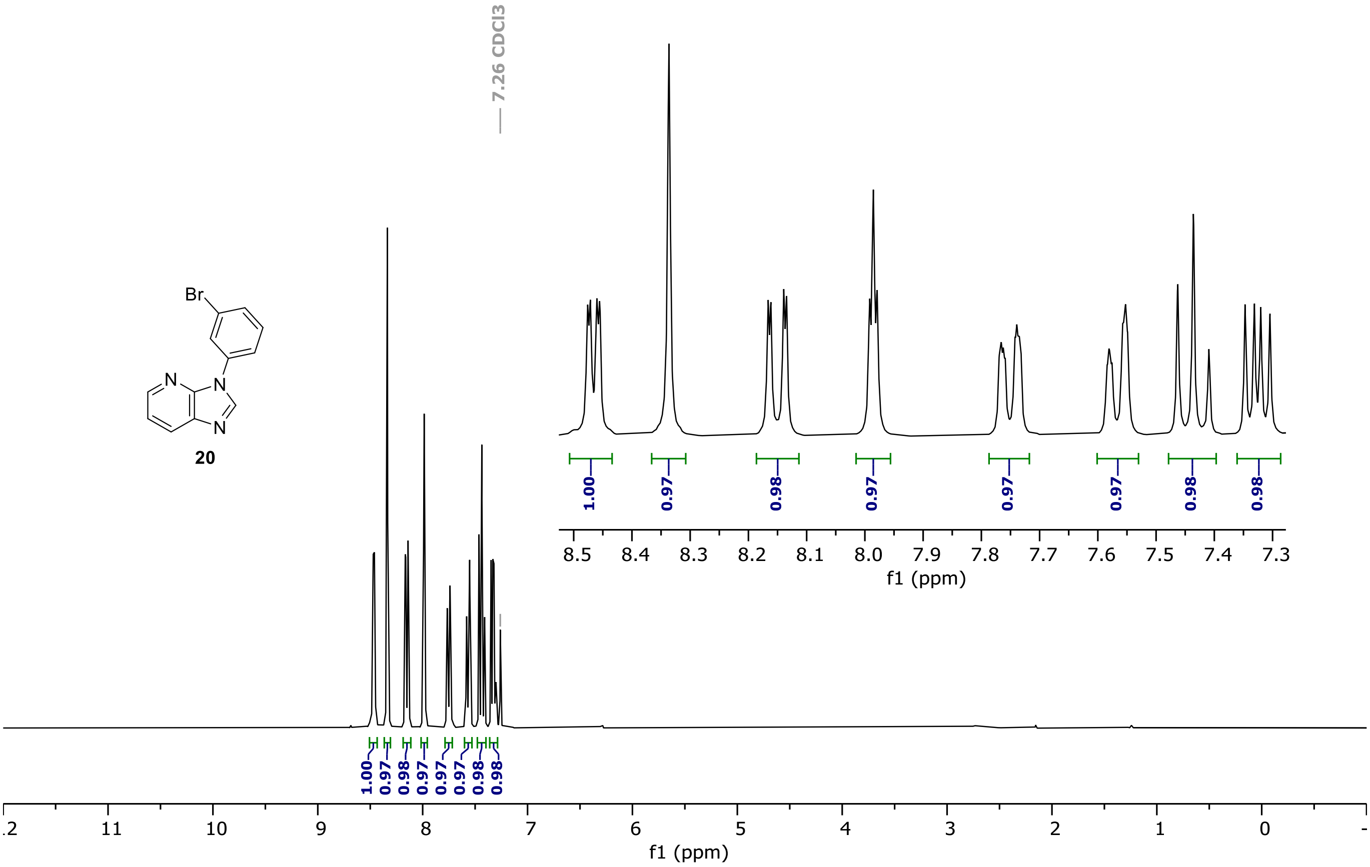

Figure S47. *^{1}H NMR (300 MHz, $CDCl_3$, 298 K) spectrum of* ***20***

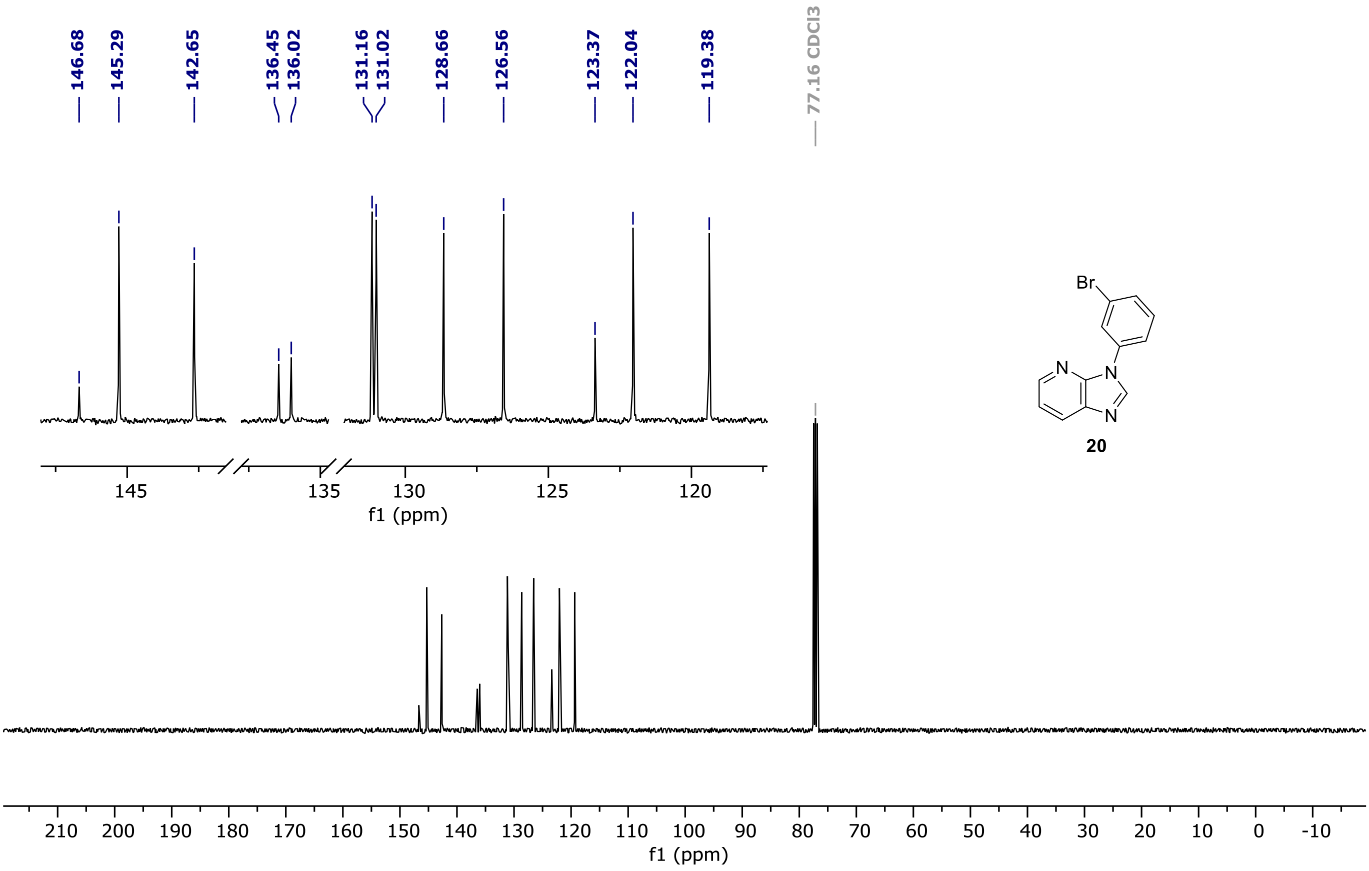

Figure S48. *$^{13}C\{^{1}H\}$ NMR (101 MHz, $CDCl_3$, 298 K) spectrum of* ***20***

¹H and ¹³C NMR spectra

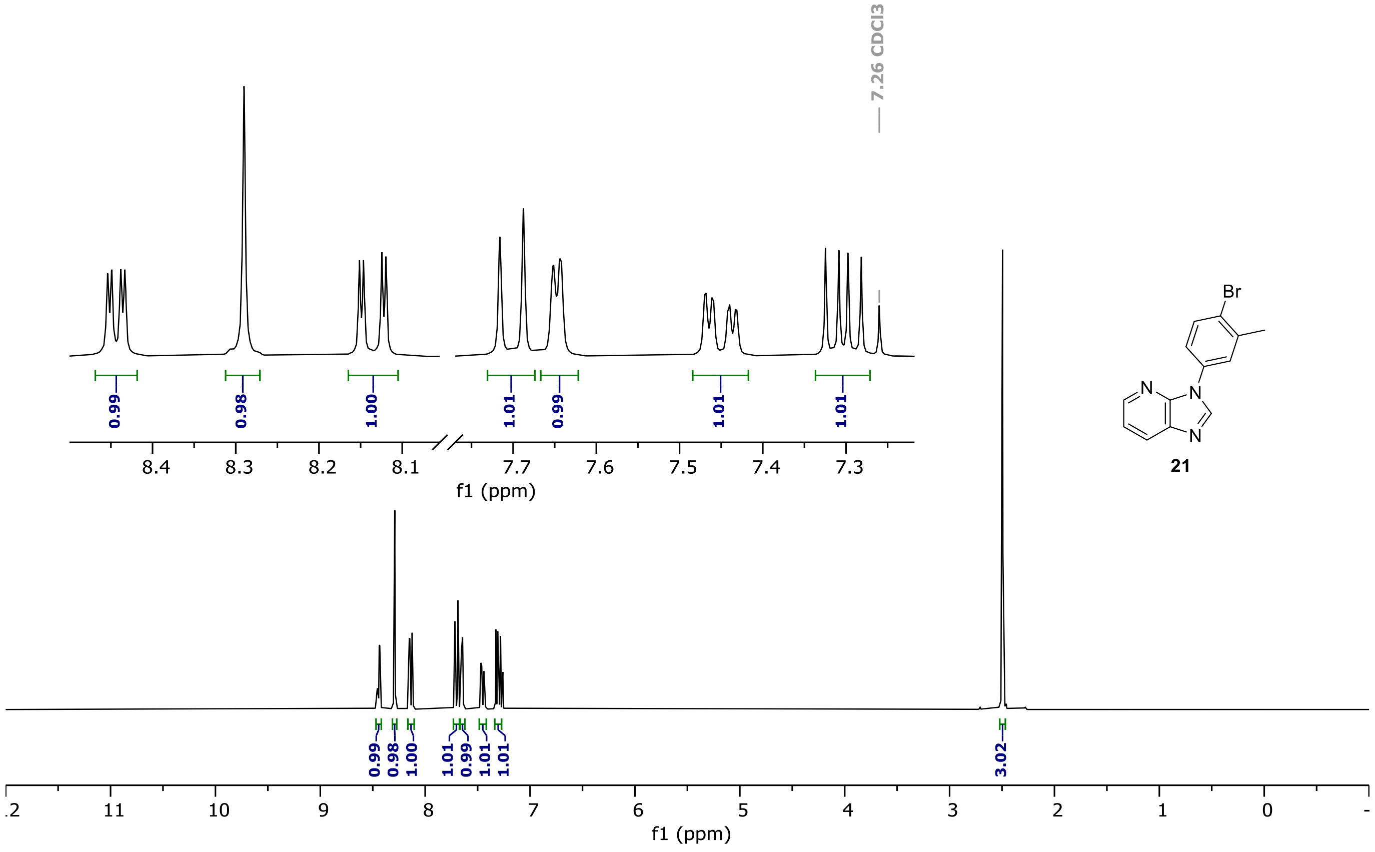

Figure S49. *¹H NMR (300 MHz, CDCl₃, 298 K) spectrum of* ***21***

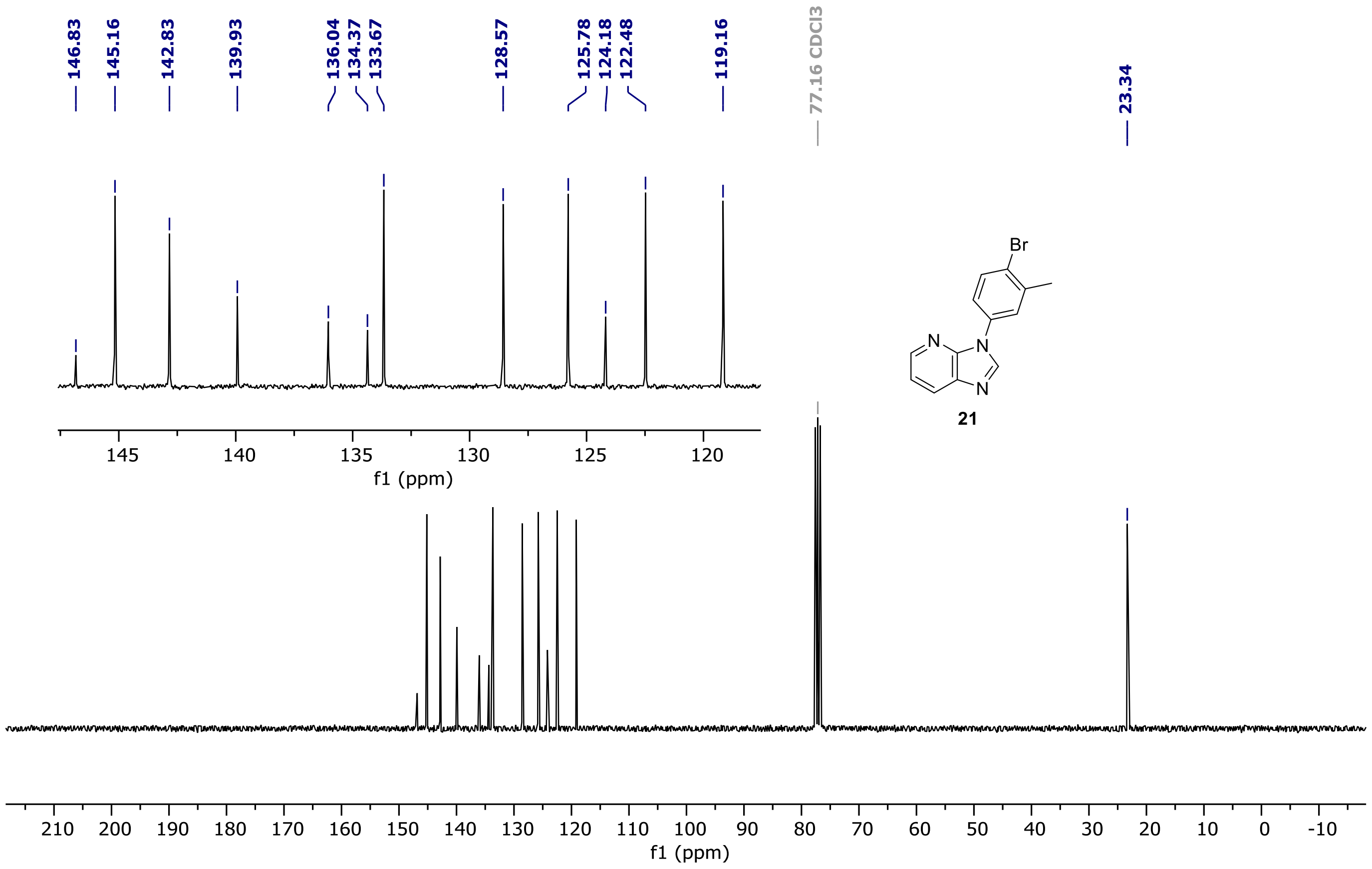

Figure S50. *¹³C{¹H} NMR (75 MHz, CDCl₃, 298 K) spectrum of* ***21***

^{1}H and ^{13}C NMR spectra

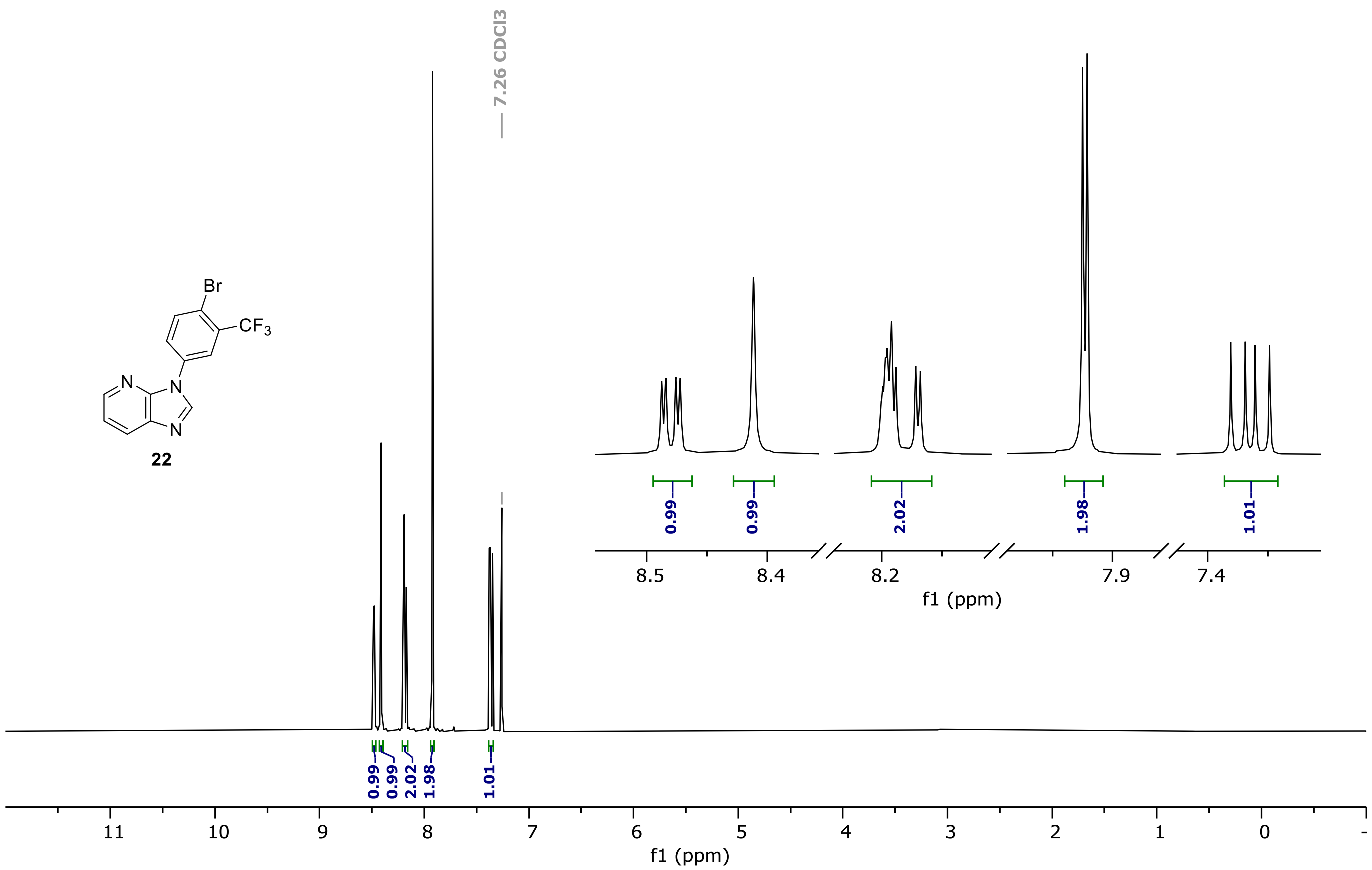

Figure S51. *^{1}H NMR (401 MHz, $CDCl_3$, 298 K) spectrum of* ***22***

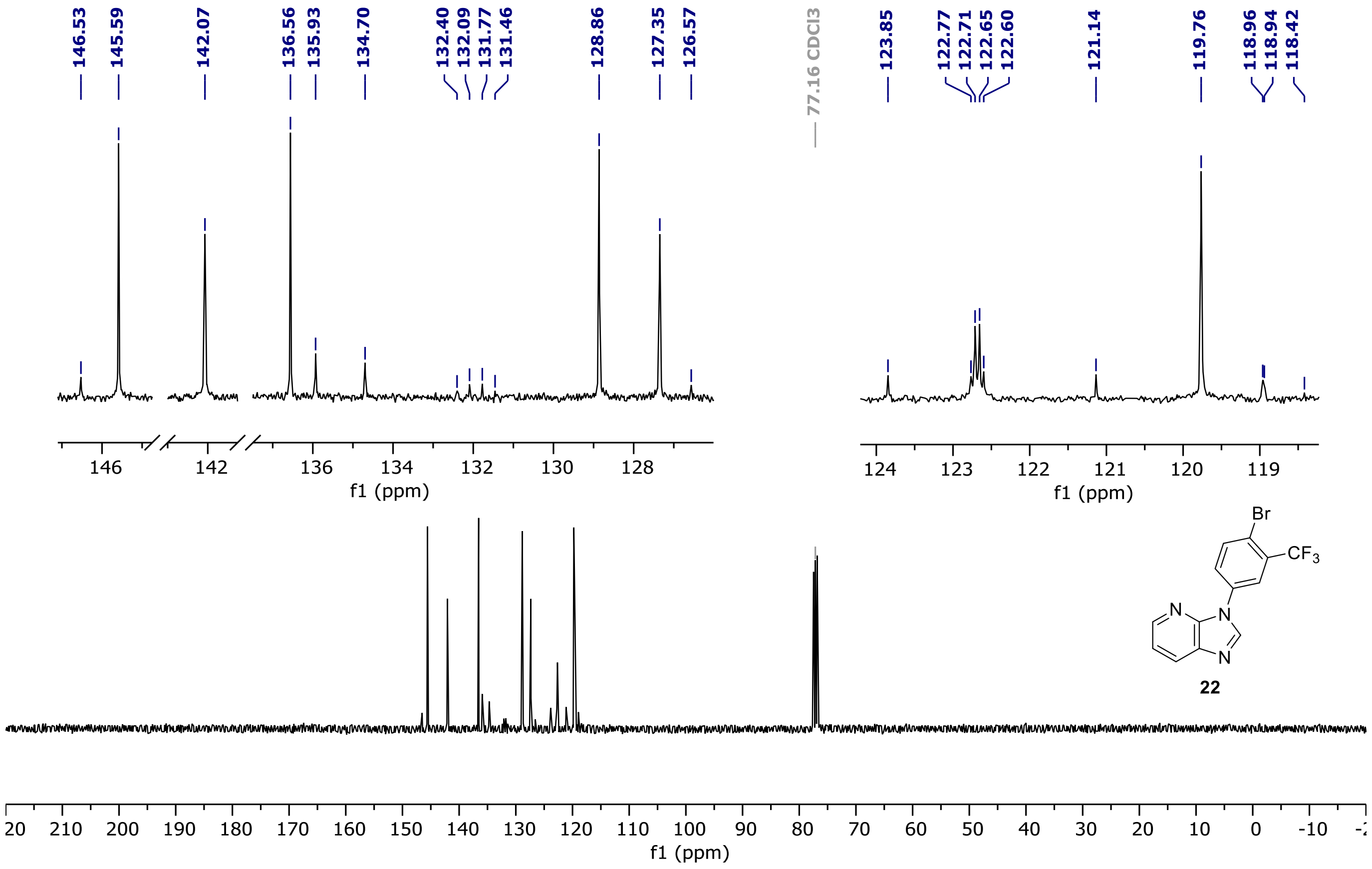

Figure S52. *$^{13}C\{^{1}H\}$ NMR (101 MHz, $CDCl_3$, 298 K) spectrum of* ***22***

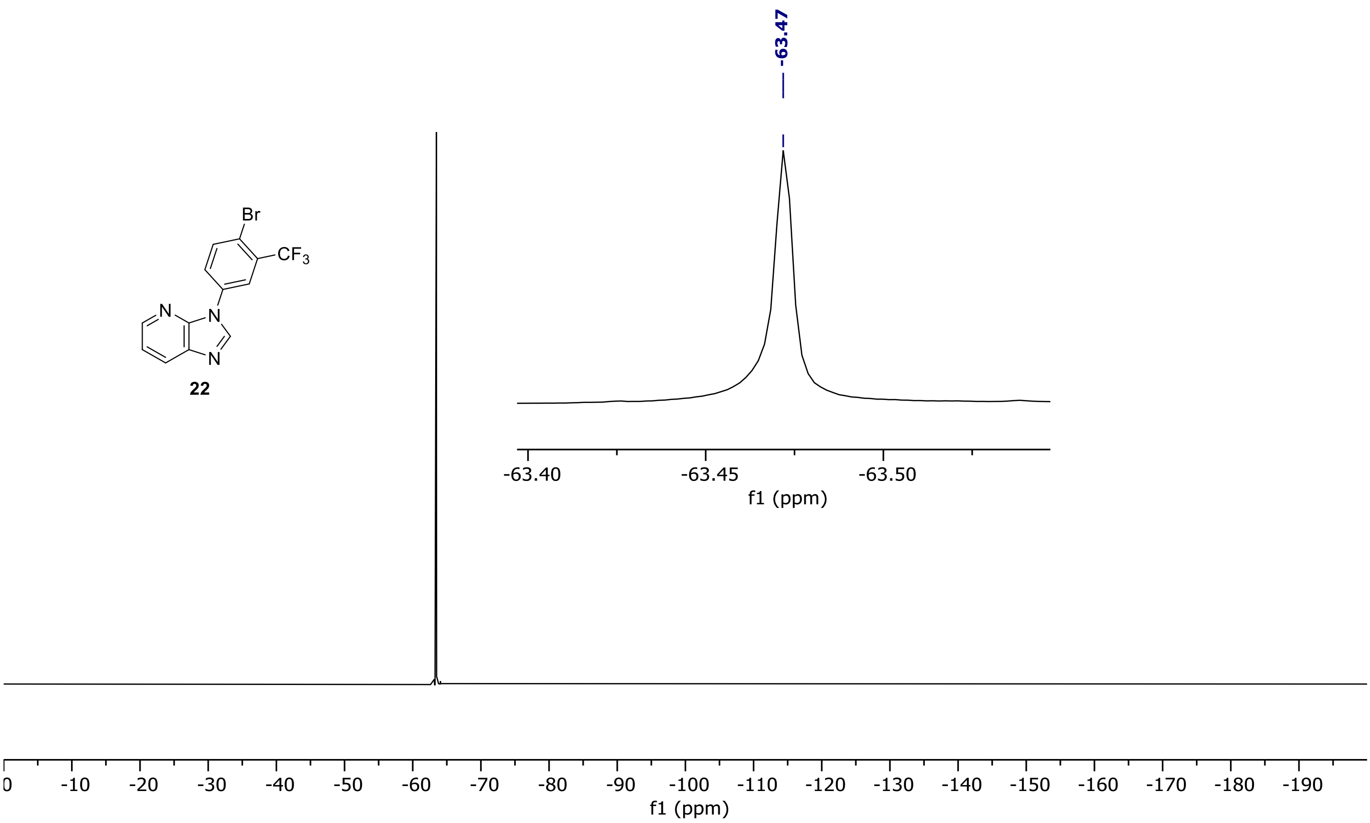

Figure S53. *^{19}F NMR (376 MHz, $CDCl_3$, 298 K) spectrum of* ***22***

^1H and ^{13}C NMR spectra

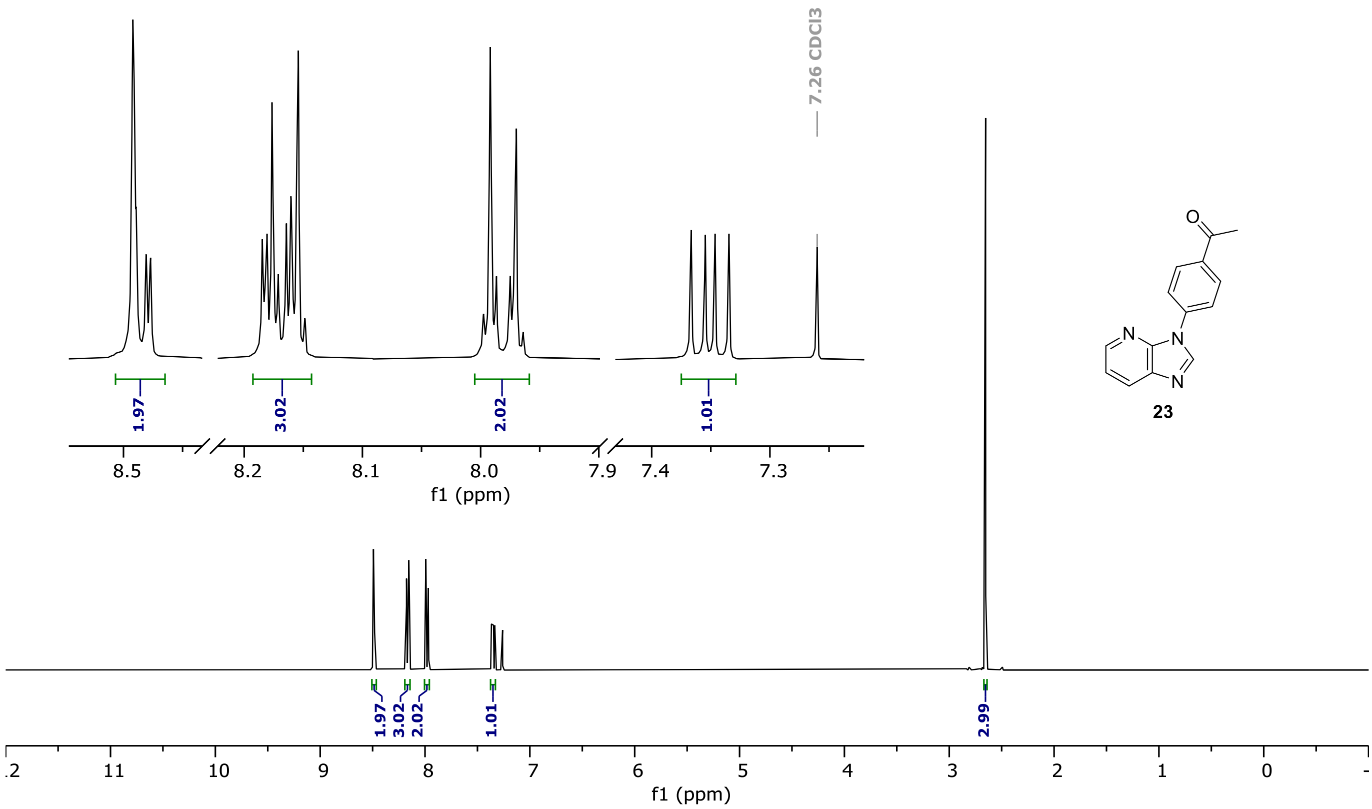

Figure S54. *^1H NMR (401 MHz, $CDCl_3$, 298 K) spectrum of* ***23***

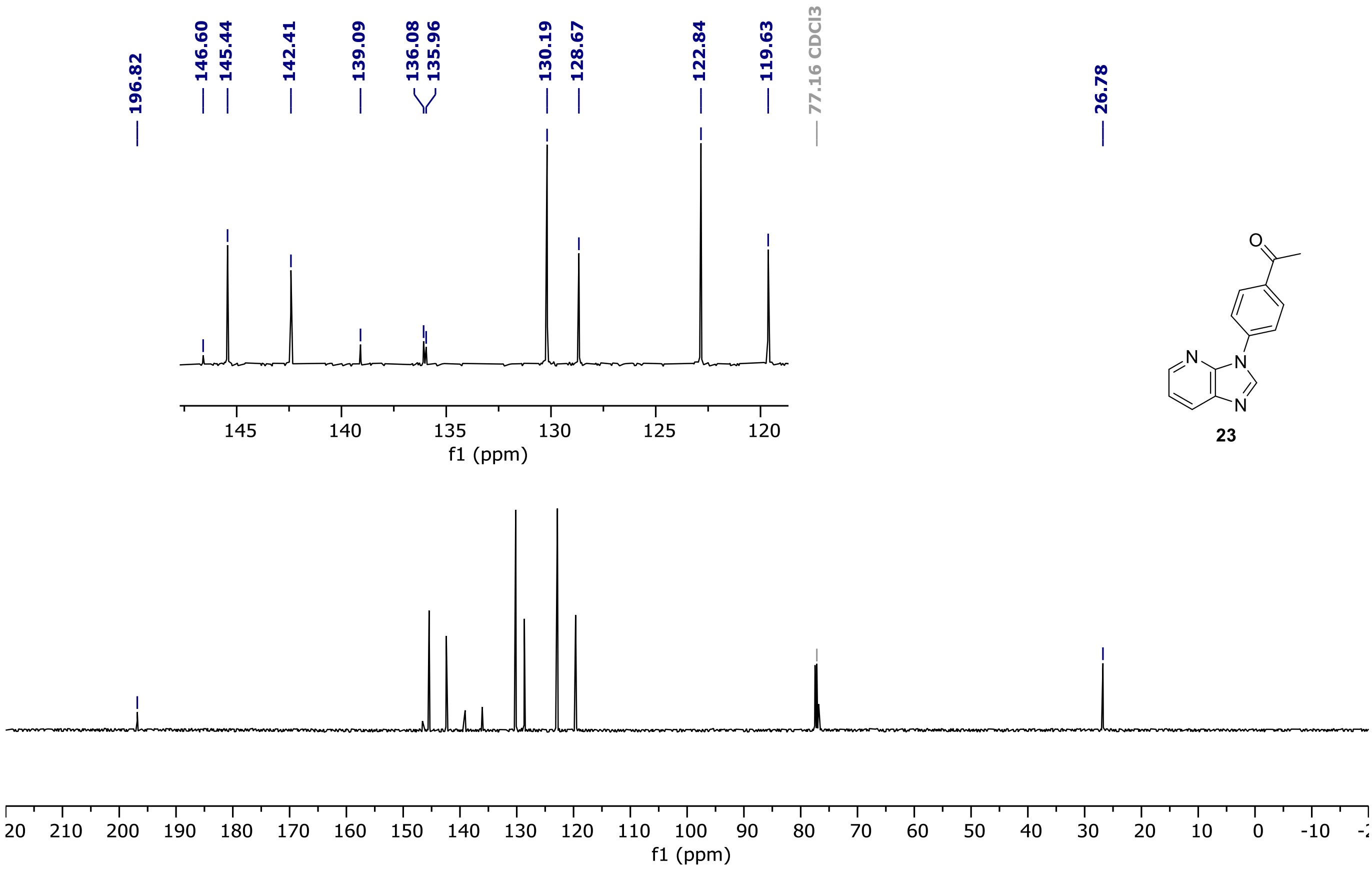

Figure S55. *$^{13}C\{^1H\}$ NMR (101 MHz, $CDCl_3$, 298 K) spectrum of* ***23***

^{1}H and ^{13}C NMR spectra

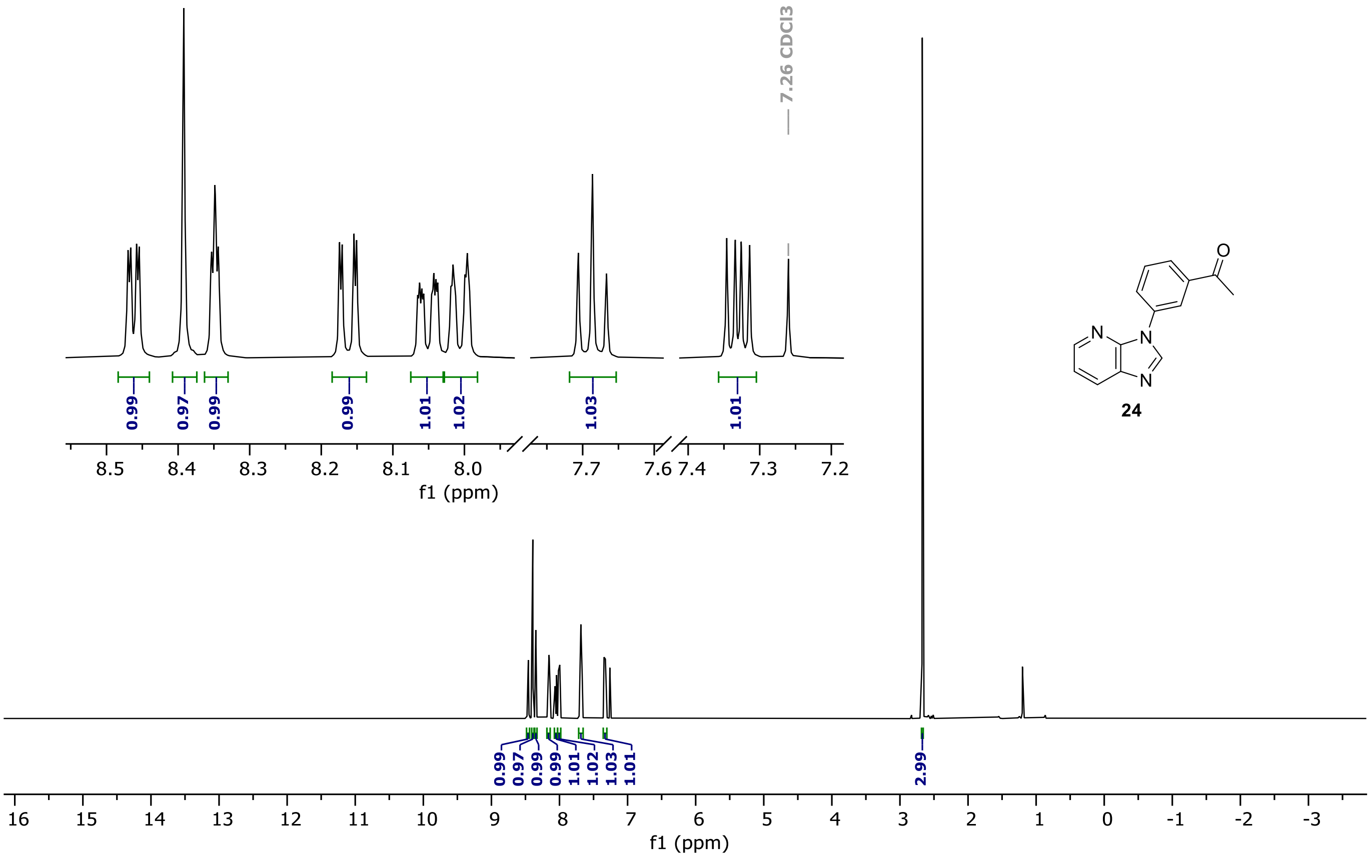

Figure S56. *^{1}H NMR (401 MHz, $CDCl_3$, 298 K) spectrum of* ***24***

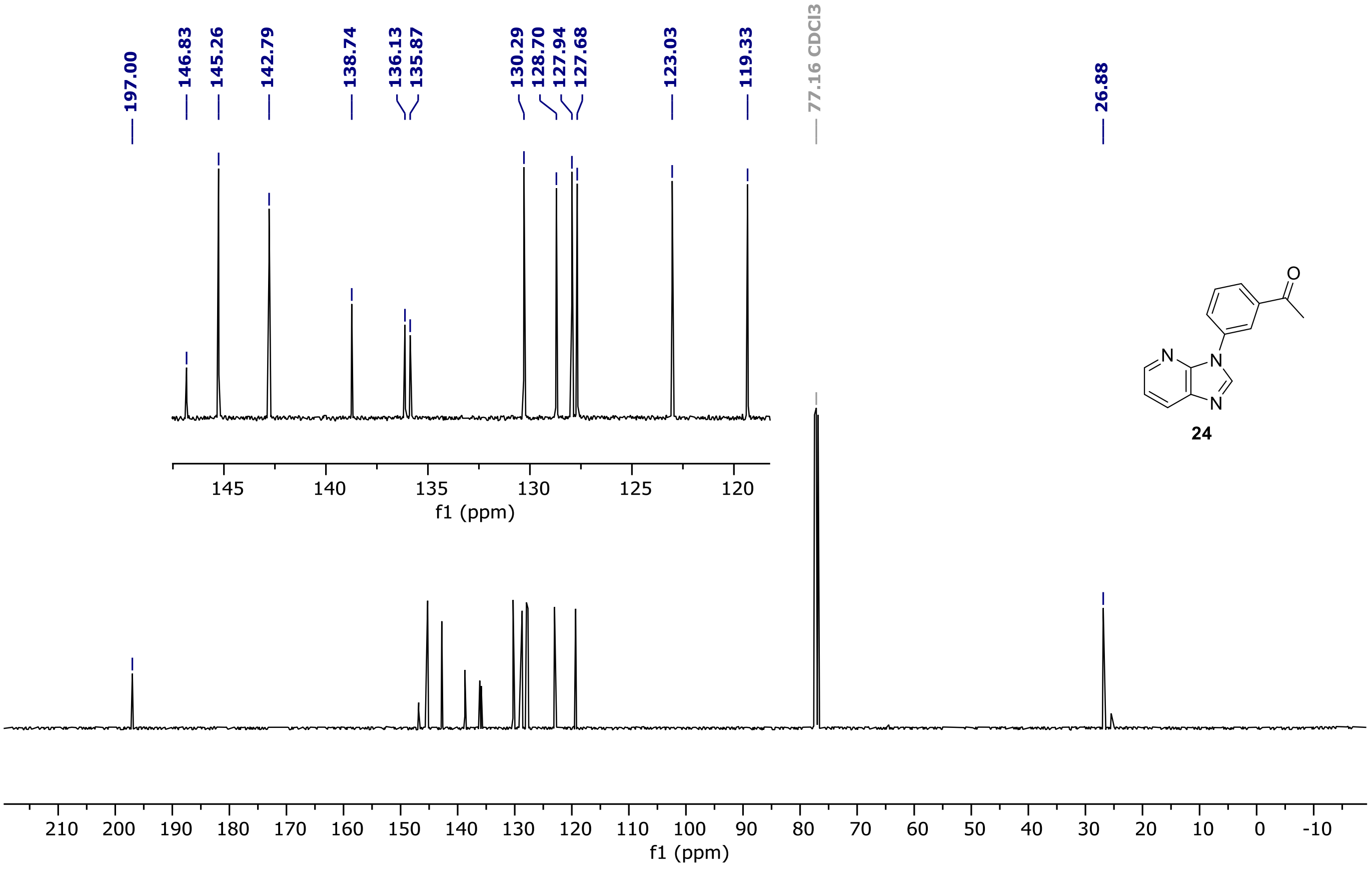

Figure S57. *$^{13}C\{^{1}H\}$ NMR (101 MHz, $CDCl_3$, 298 K) spectrum of* ***24***

^{1}H and ^{13}C NMR spectra

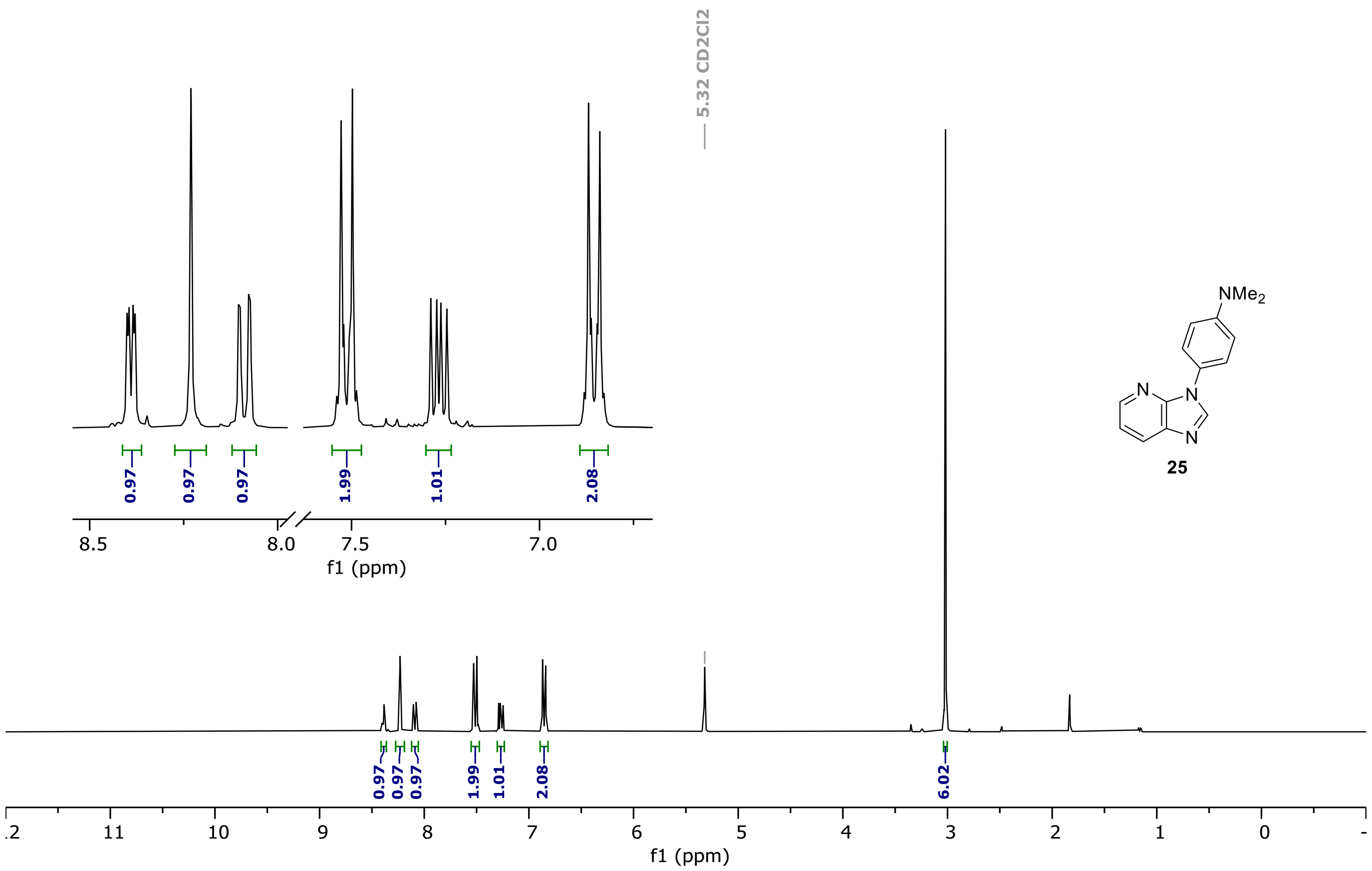

Figure S58. *^{1}H NMR (300 MHz, $CDCl_3$, 298 K) spectrum of **25***

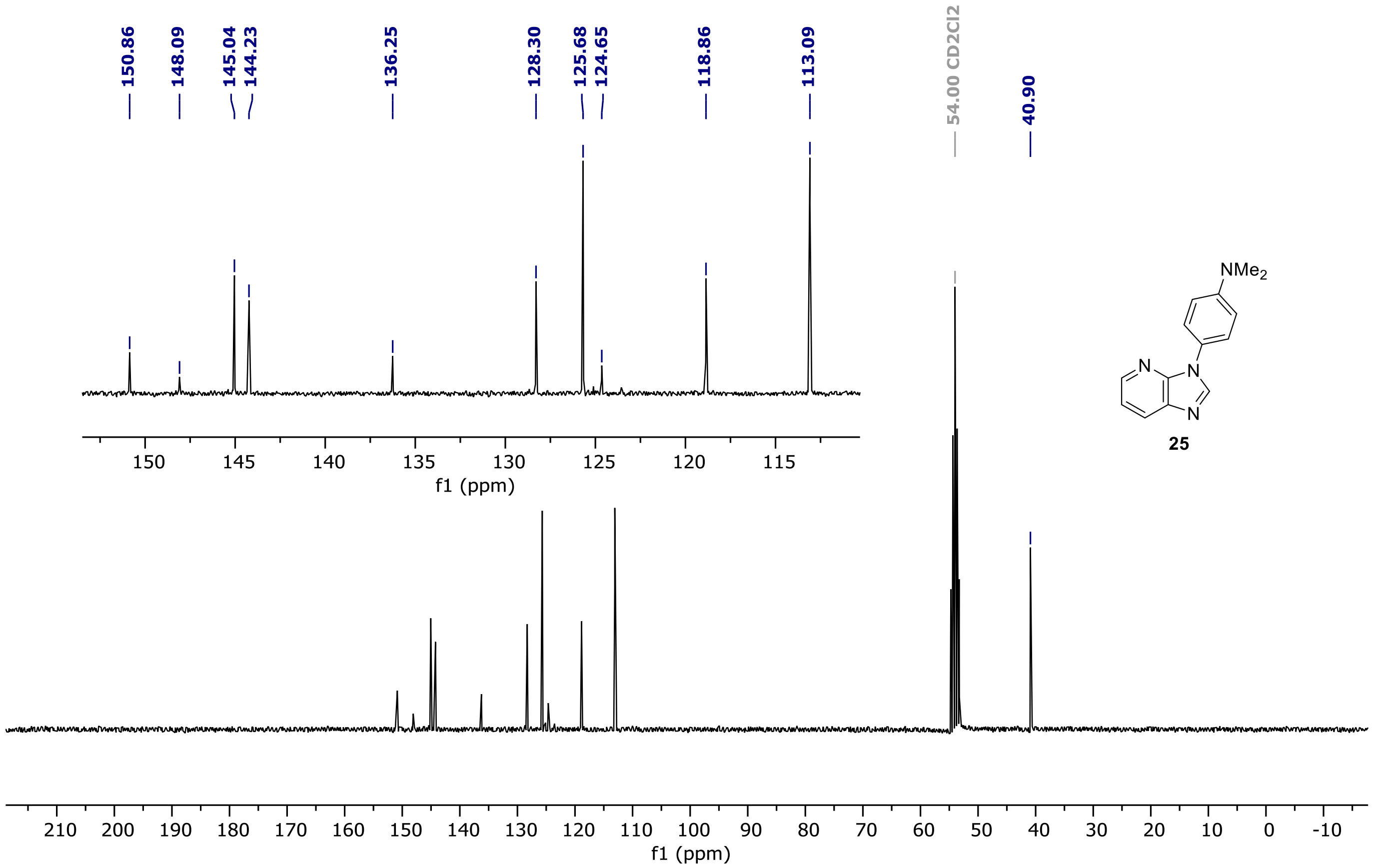

Figure S59. *^{13}C{^{1}H} NMR (75 MHz, $CDCl_3$, 298 K) spectrum of **25***

^{1}H and ^{13}C NMR spectra

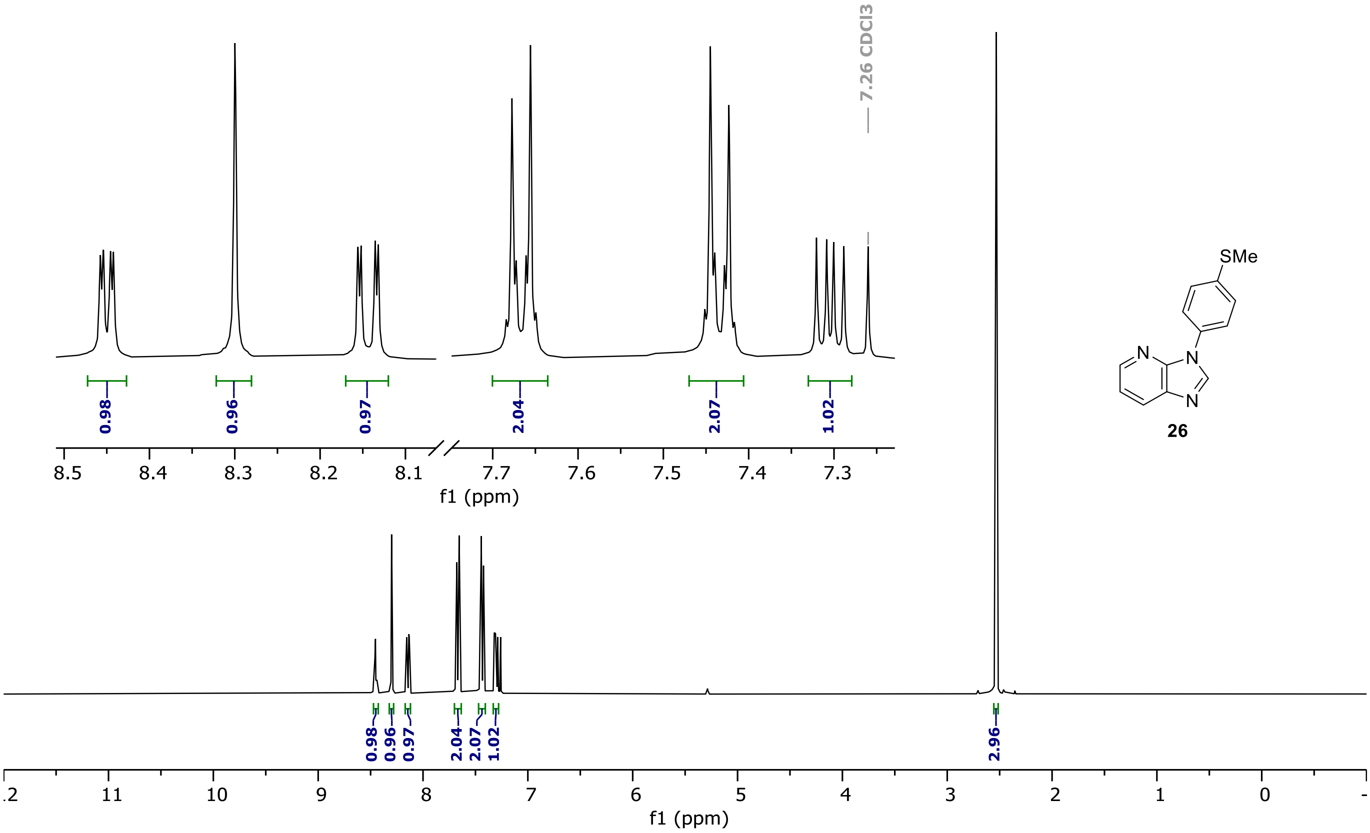

Figure S60. ^{1}H NMR (401 MHz, $CDCl_3$, 298 K) spectrum of **26**

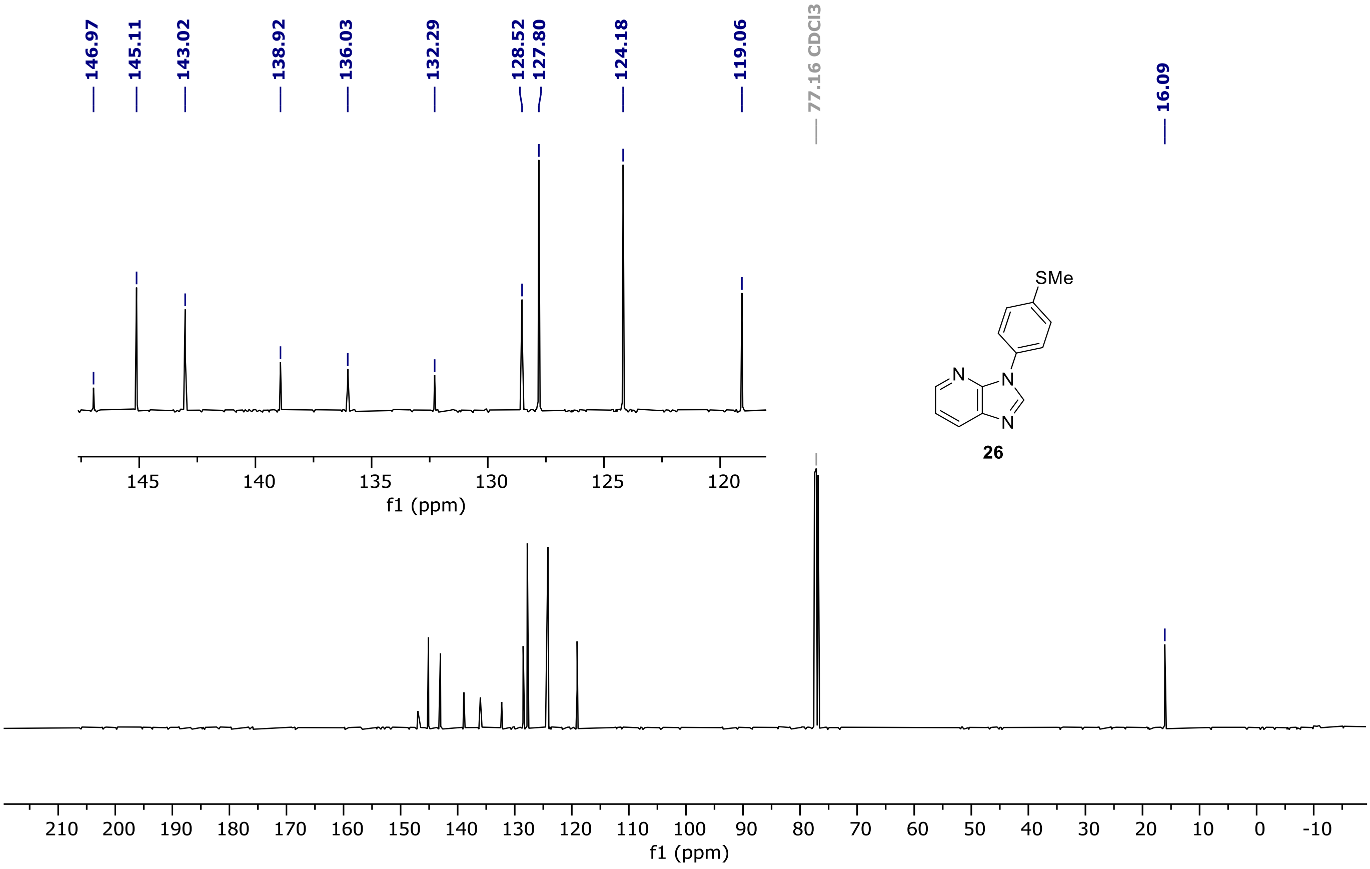

Figure S61. $^{13}C\{^{1}H\}$ NMR (101 MHz, $CDCl_3$, 298 K) spectrum of **26**

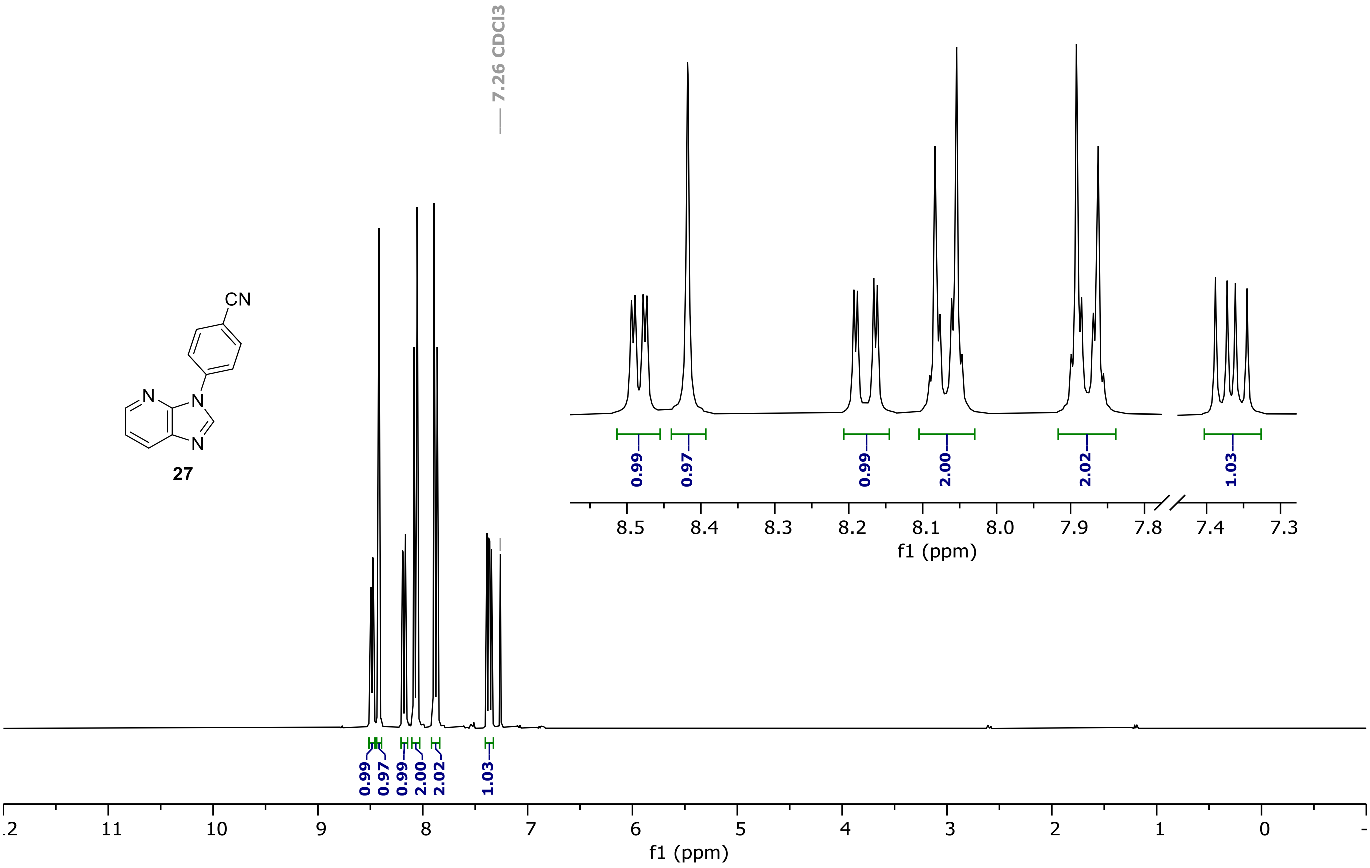

Figure S62. ^{1}H NMR (300 MHz, $CDCl_3$, 298 K) spectrum of **27**

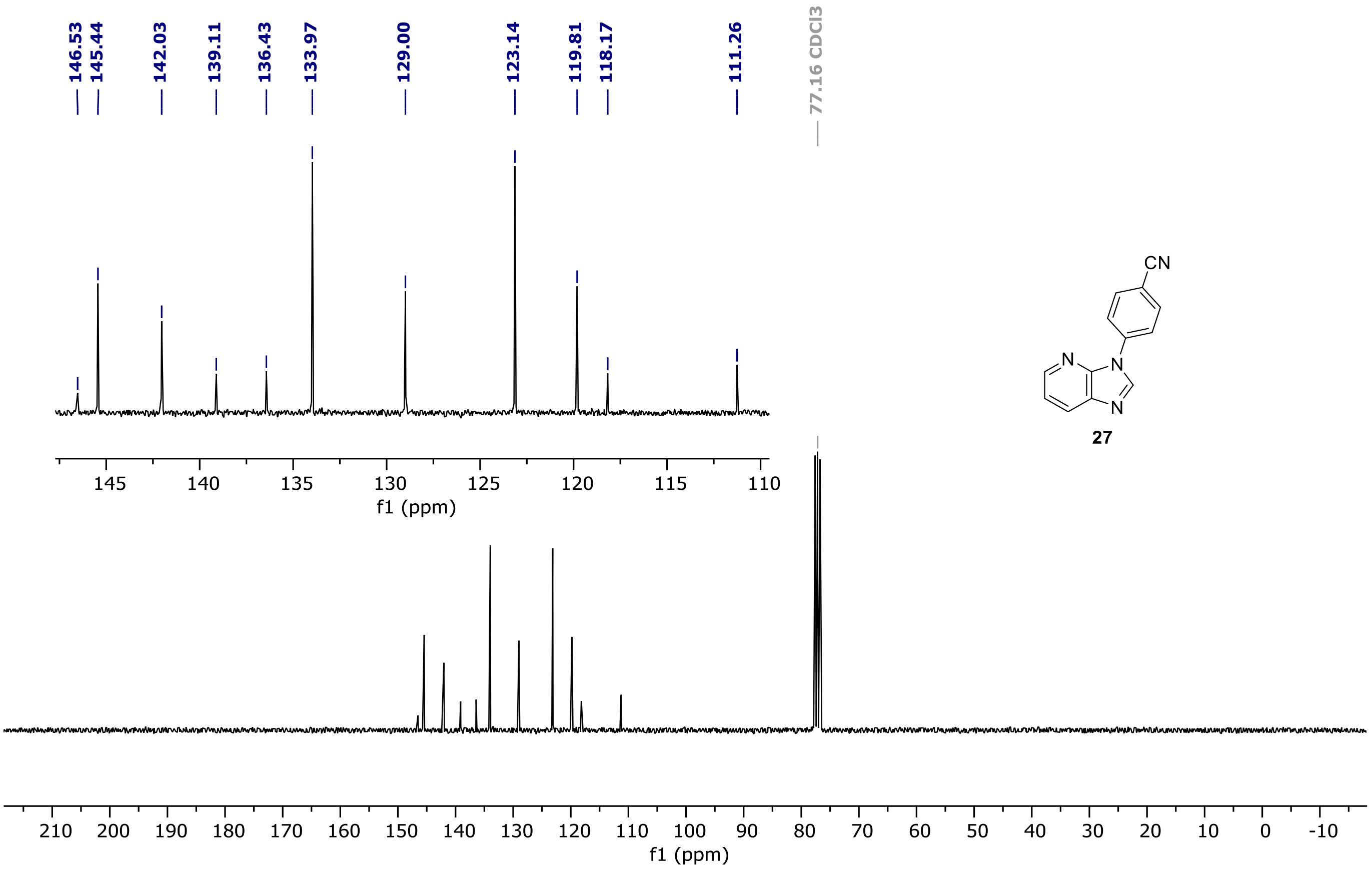

Figure S63. $^{13}C\{^{1}H\}$ NMR (75 MHz, $CDCl_3$, 298 K) spectrum of **27**

^{1}H and ^{13}C NMR spectra

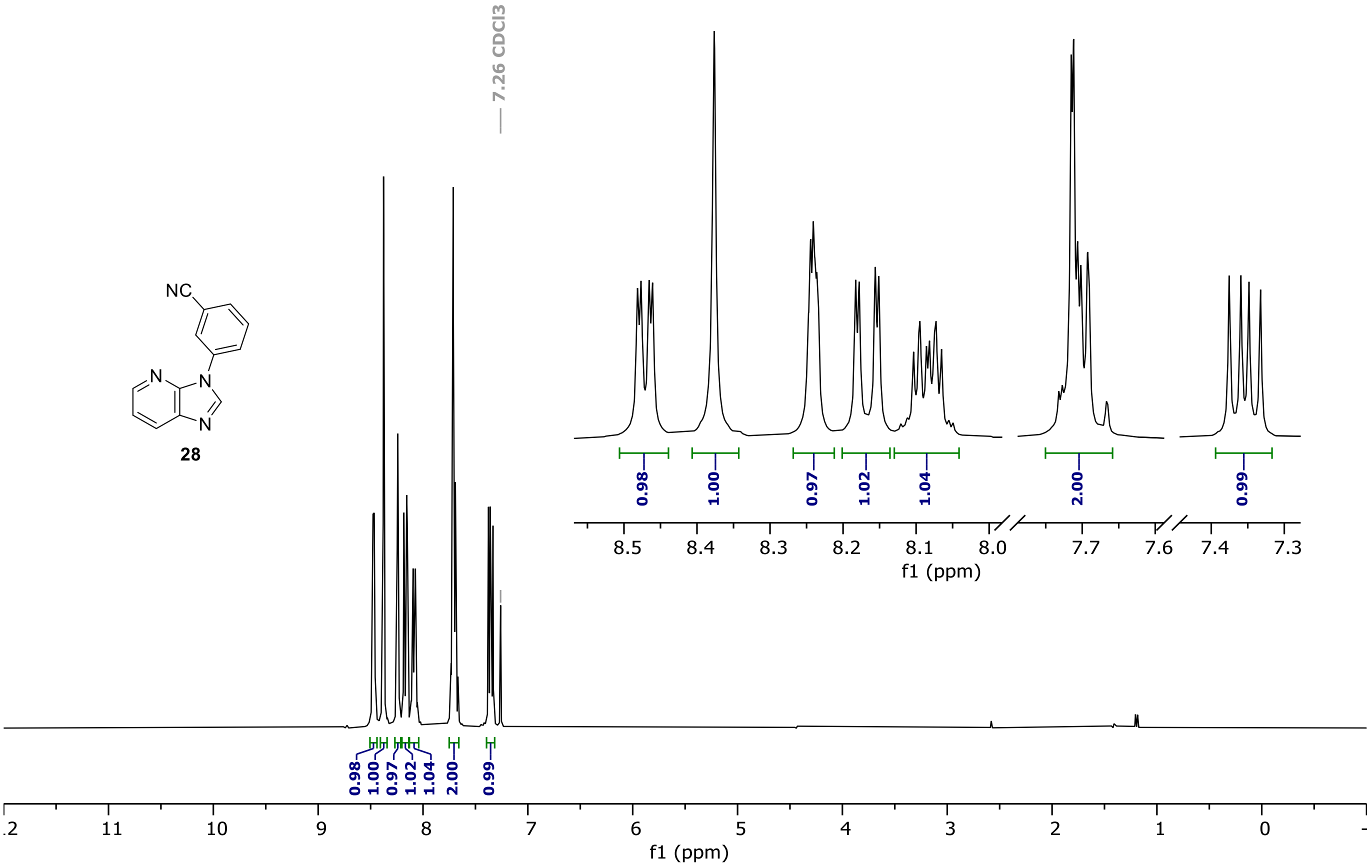

Figure S64. *^{1}H NMR (300 MHz, $CDCl_3$, 298 K) spectrum of **28***

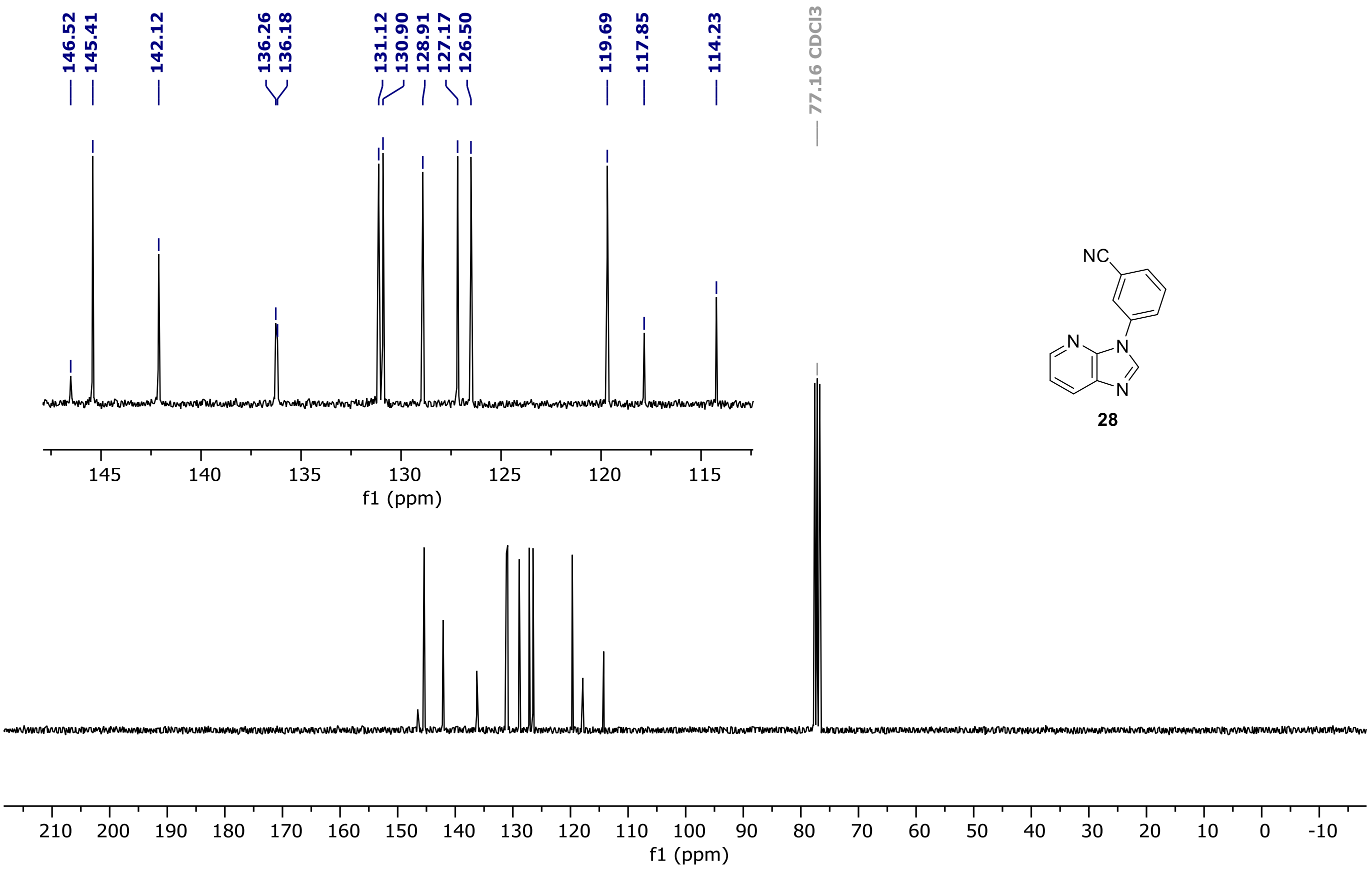

Figure S65. *$^{13}C\{^{1}H\}$ NMR (75 MHz, $CDCl_3$, 298 K) spectrum of **28***

^{1}H and ^{13}C NMR spectra

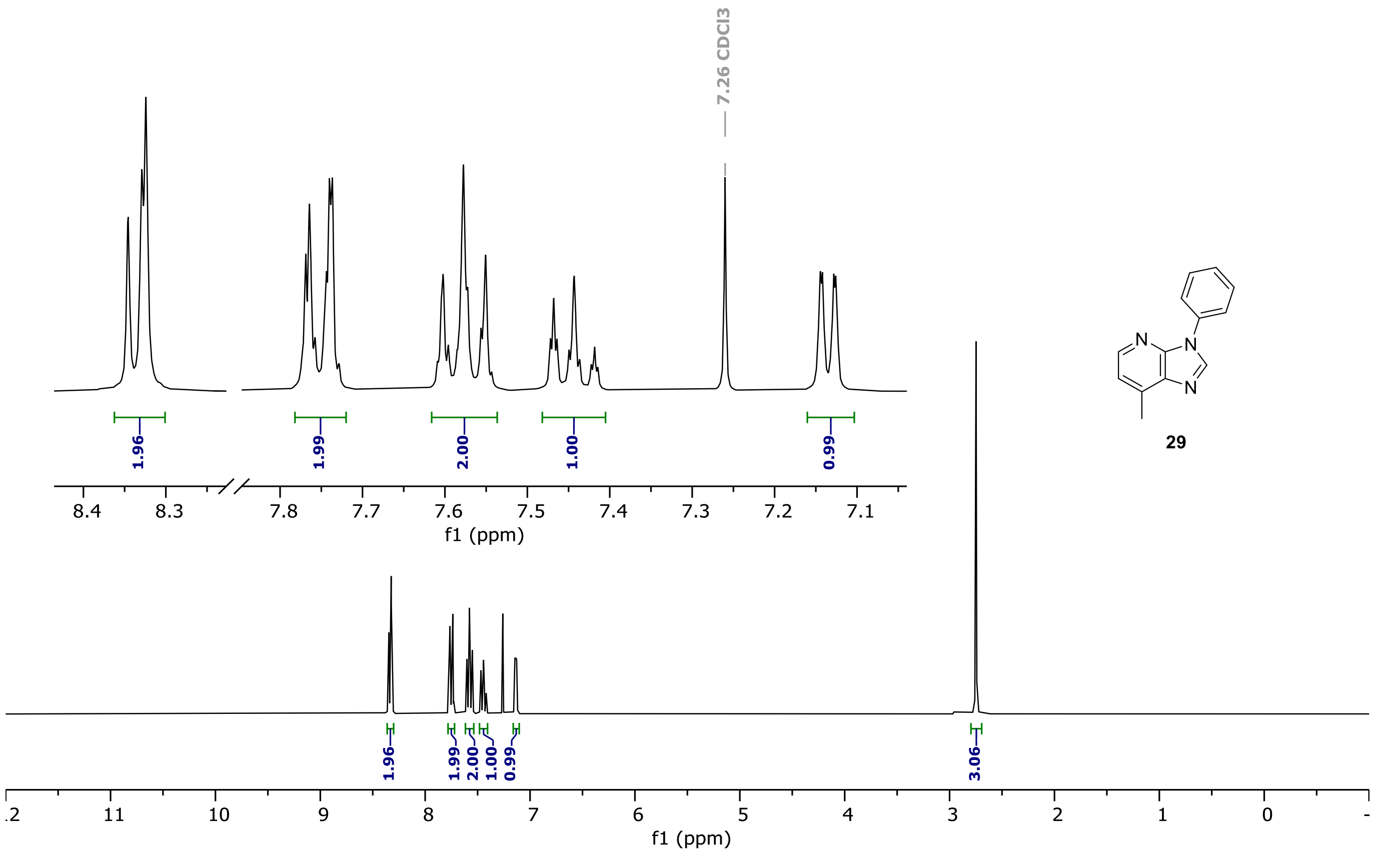

Figure S66. *^{1}H NMR (300 MHz, $CDCl_3$, 298 K) spectrum of* ***29***

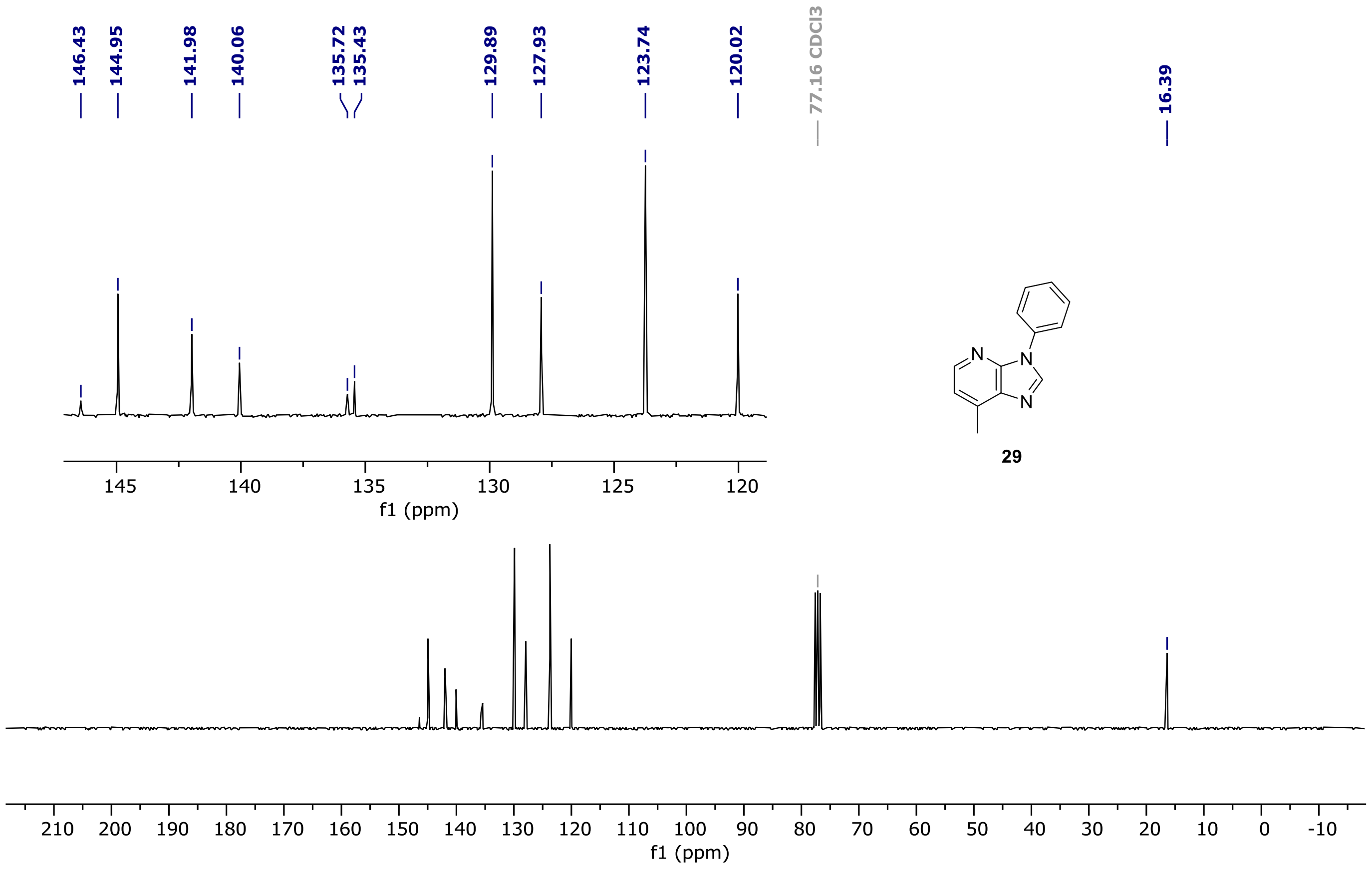

Figure S67. *$^{13}C\{^{1}H\}$ NMR (75 MHz, $CDCl_3$, 298 K) spectrum of* ***29***

^{1}H and ^{13}C NMR spectra

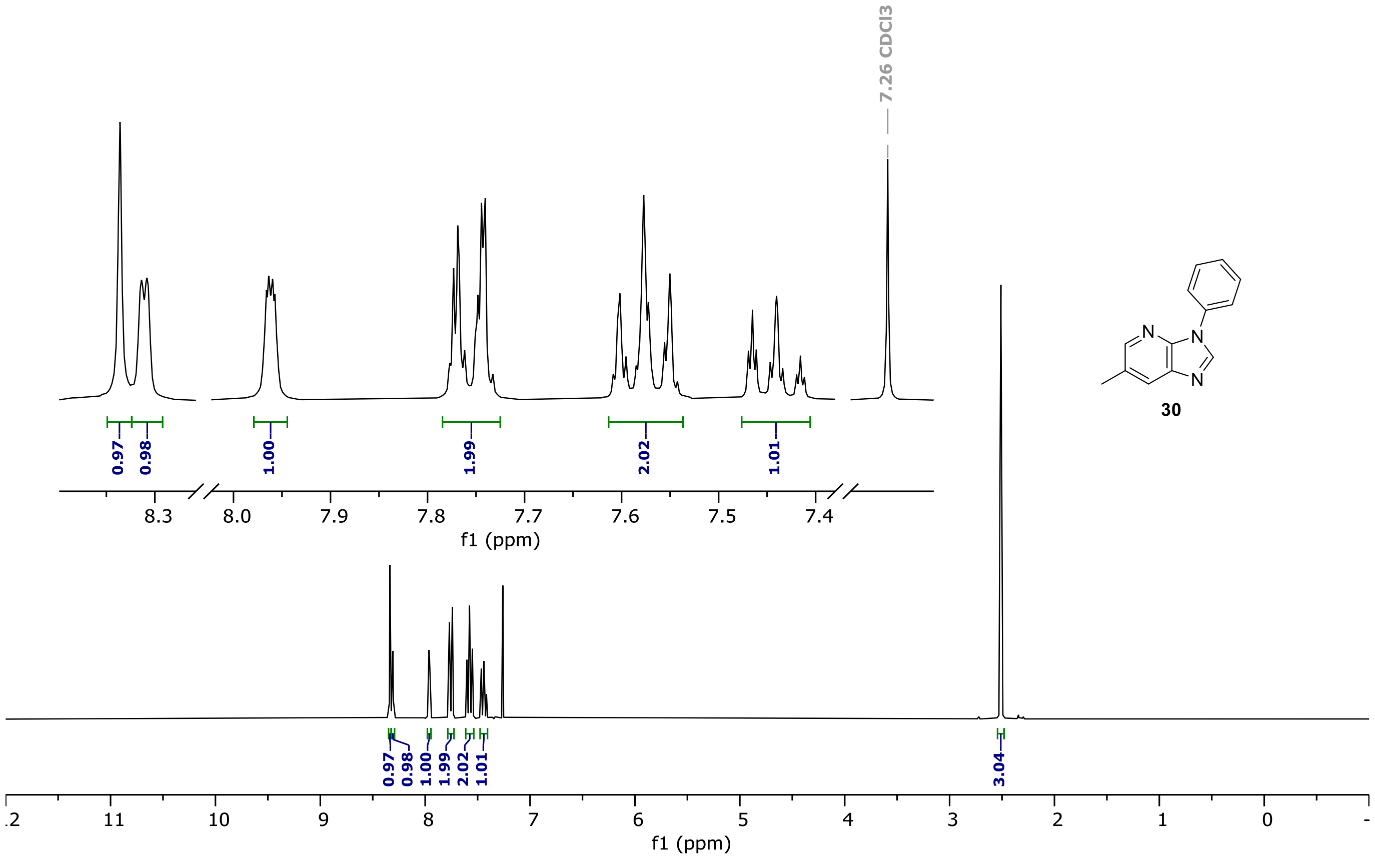

Figure S68. *^{1}H NMR (300 MHz, $CDCl_3$, 298 K) spectrum of **30***

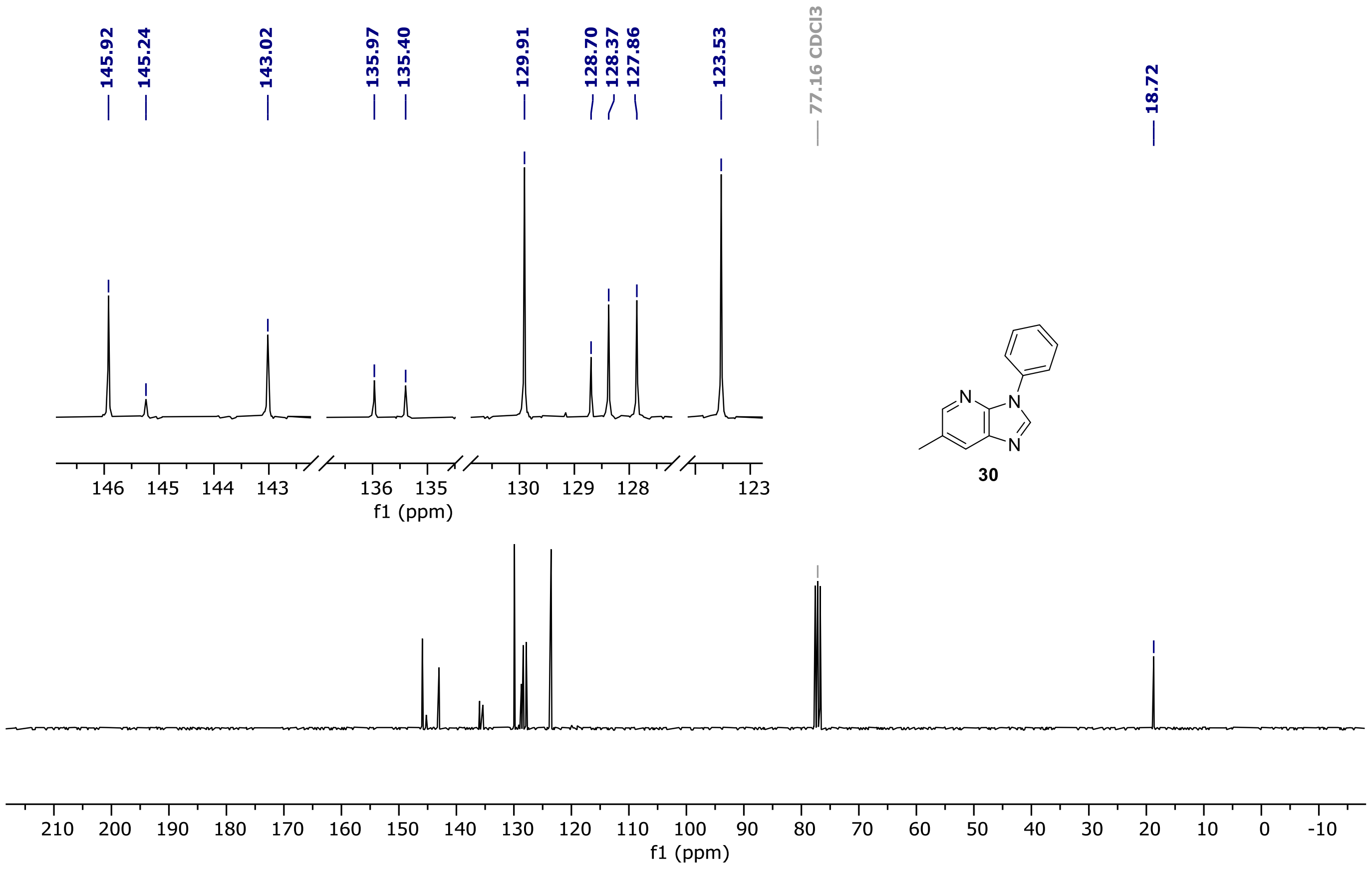

Figure S69. *$^{13}C\{^{1}H\}$ NMR (75 MHz, $CDCl_3$, 298 K) spectrum of **30***

^{1}H and ^{13}C NMR spectra

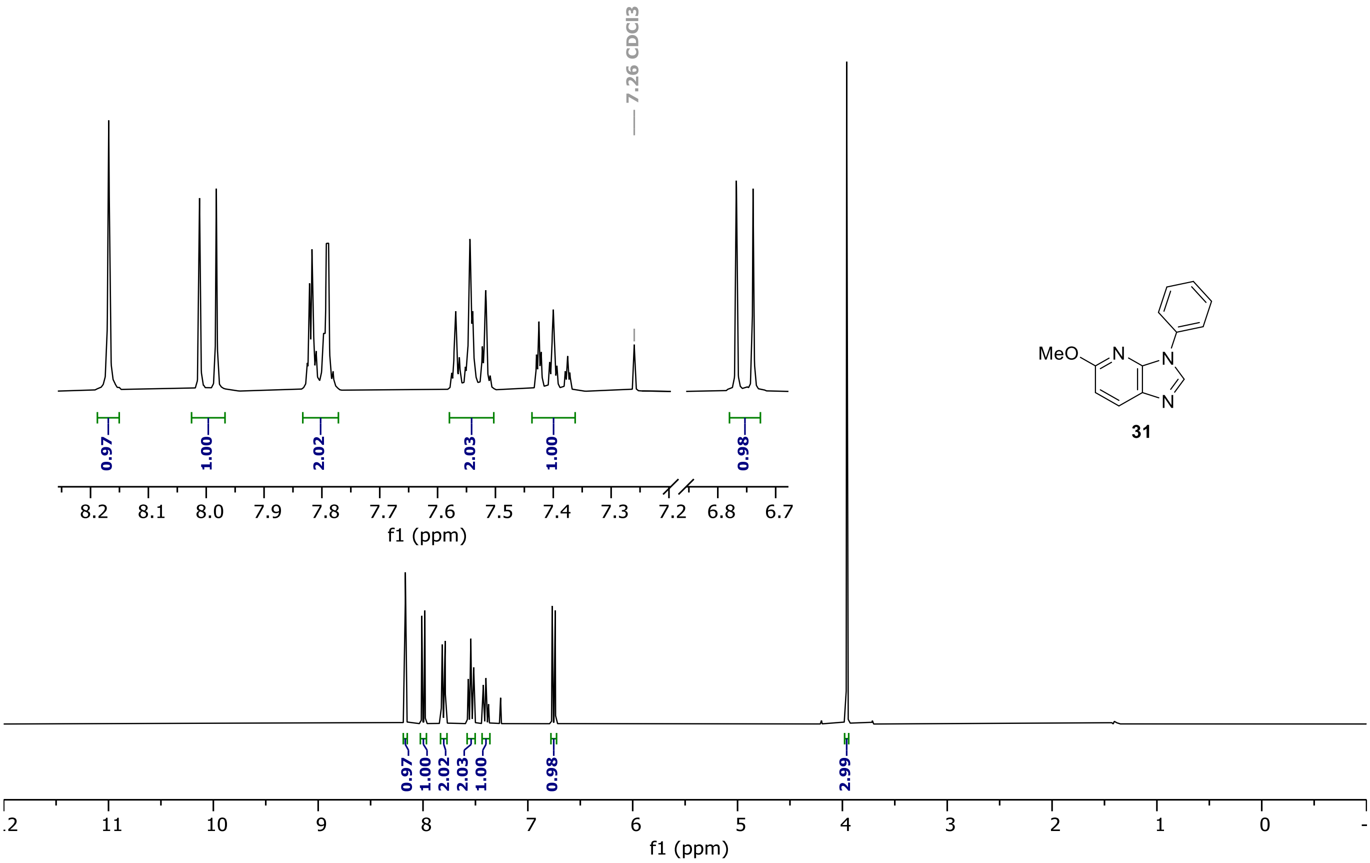

Figure S70. *^{1}H NMR (300 MHz, $CDCl_3$, 298 K) spectrum of* ***31***

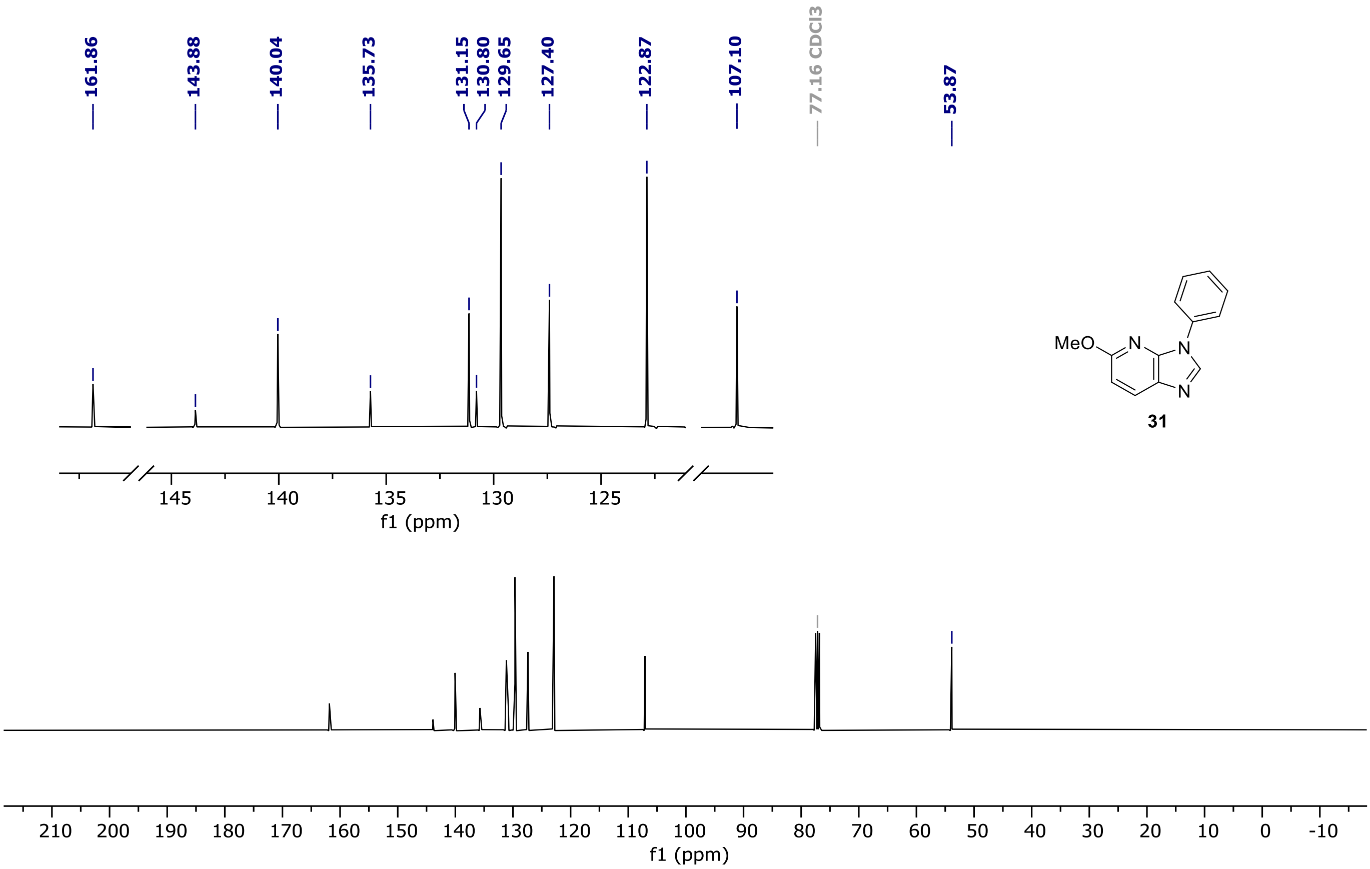

Figure S71. *$^{13}C\{^{1}H\}$ NMR (75 MHz, $CDCl_3$, 298 K) spectrum of* ***31***

^{1}H and ^{13}C NMR spectra

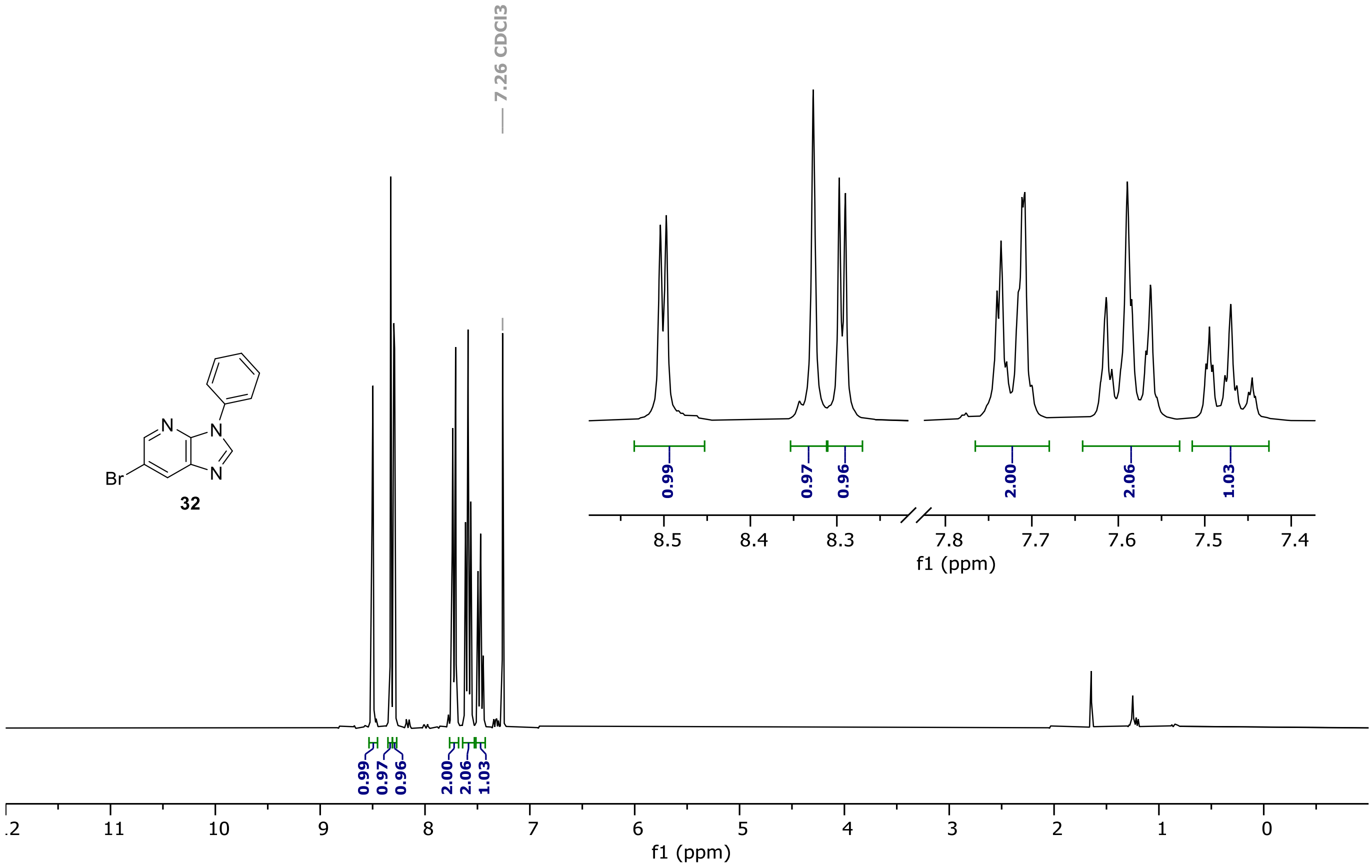

Figure S72. *^{1}H NMR (300 MHz, $CDCl_3$, 298 K) spectrum of* ***32***

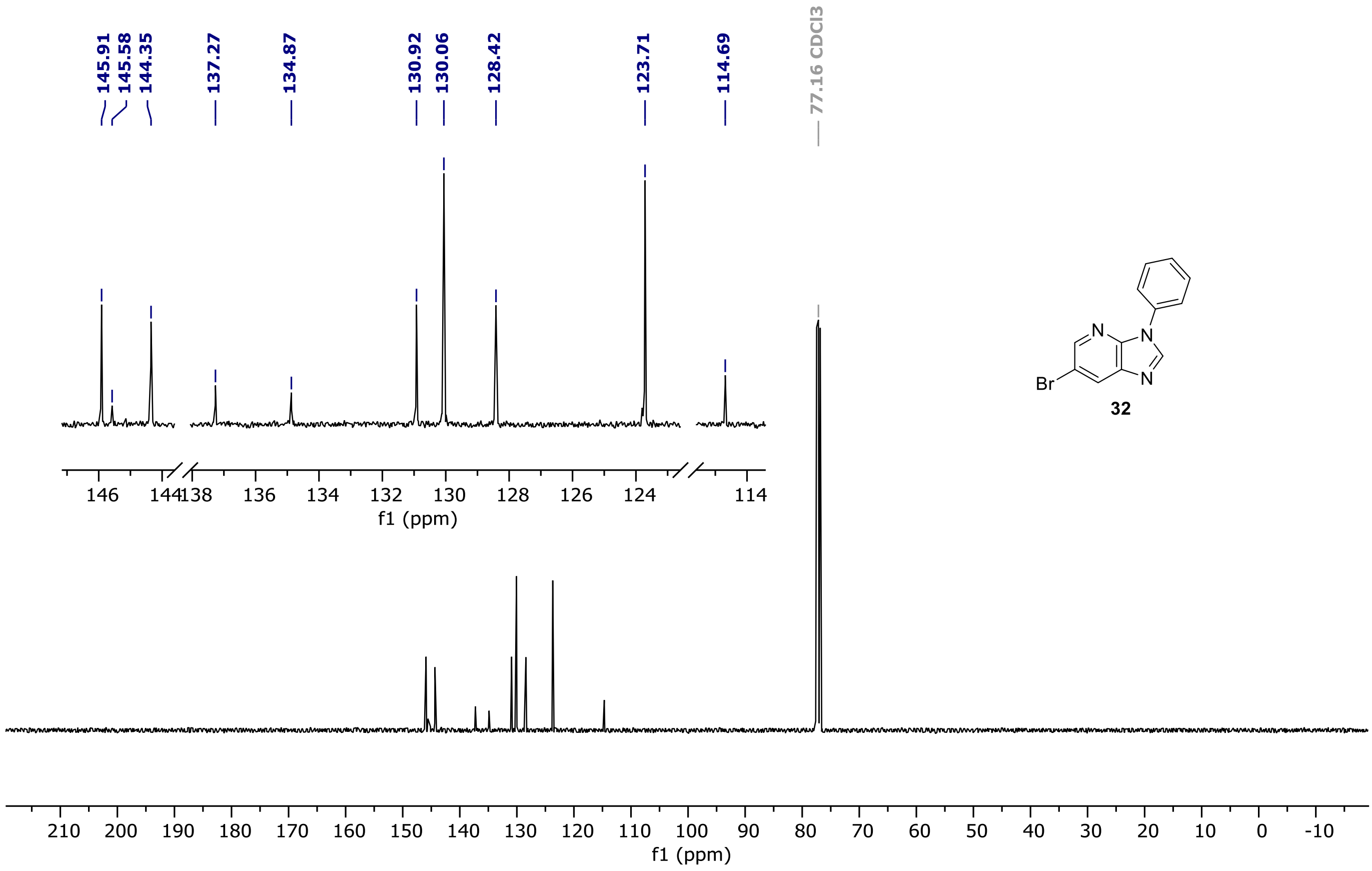

Figure S73. *$^{13}C\{^{1}H\}$ NMR (75 MHz, $CDCl_3$, 298 K) spectrum of* ***32***

^{1}H and ^{13}C NMR spectra

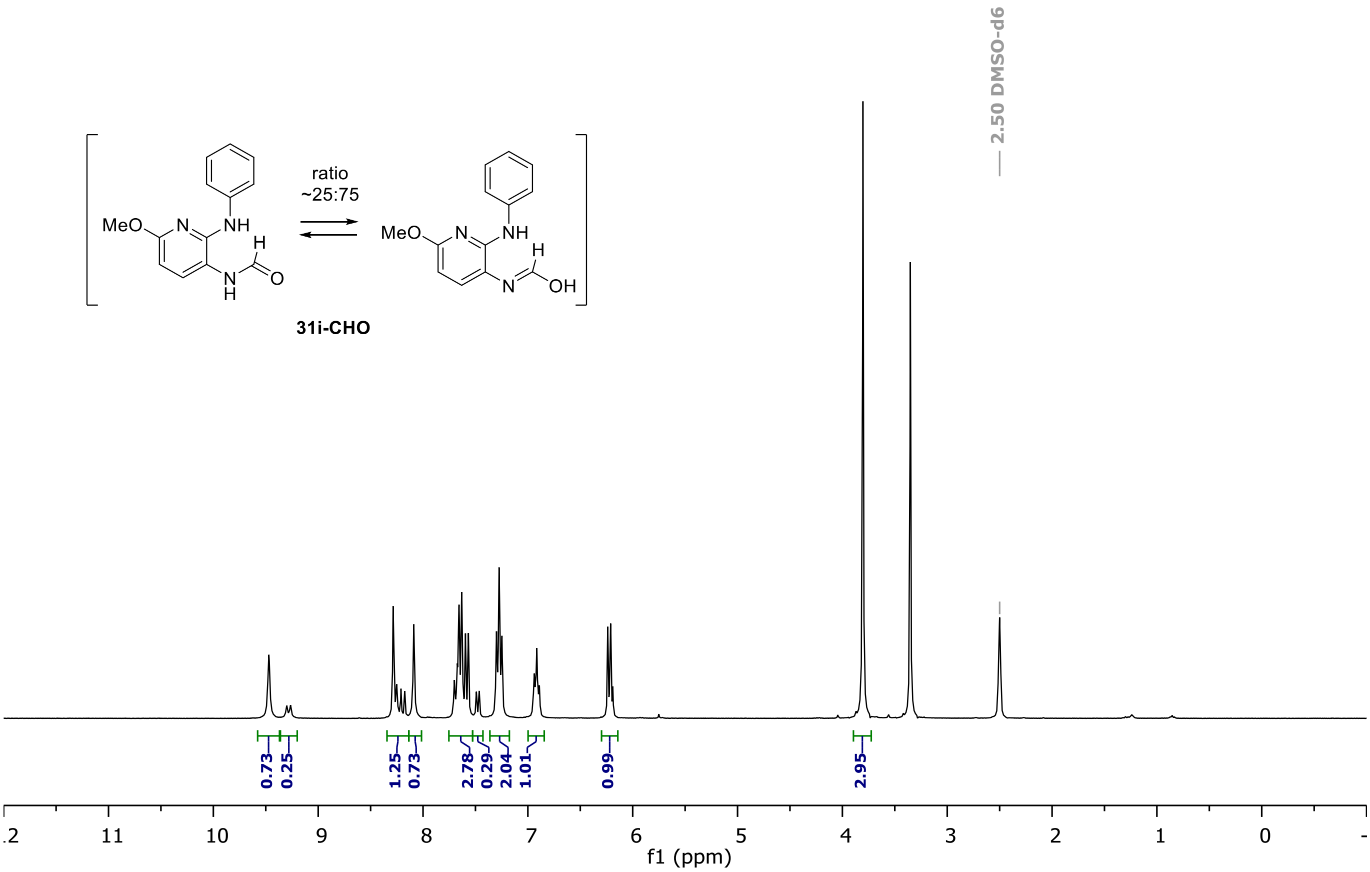

Figure S74. *^{1}H NMR (300 MHz, DMSO-d_6, 298 K) spectrum of **31i-CHO***

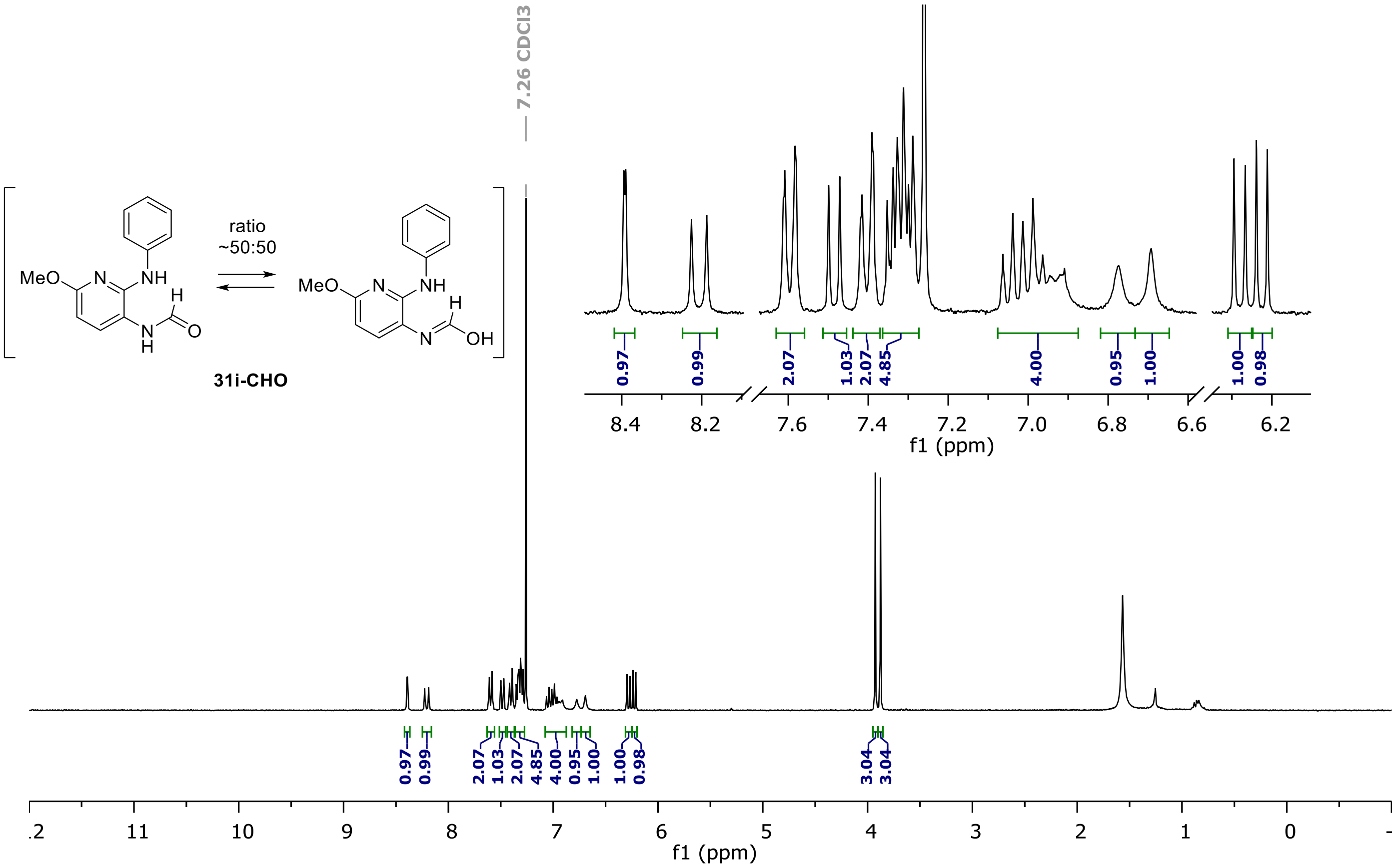

Figure S75. *^{1}H NMR (300 MHz, $CDCl_3$, 298 K) spectrum of **31i-CHO***

^{1}H and ^{13}C NMR spectra

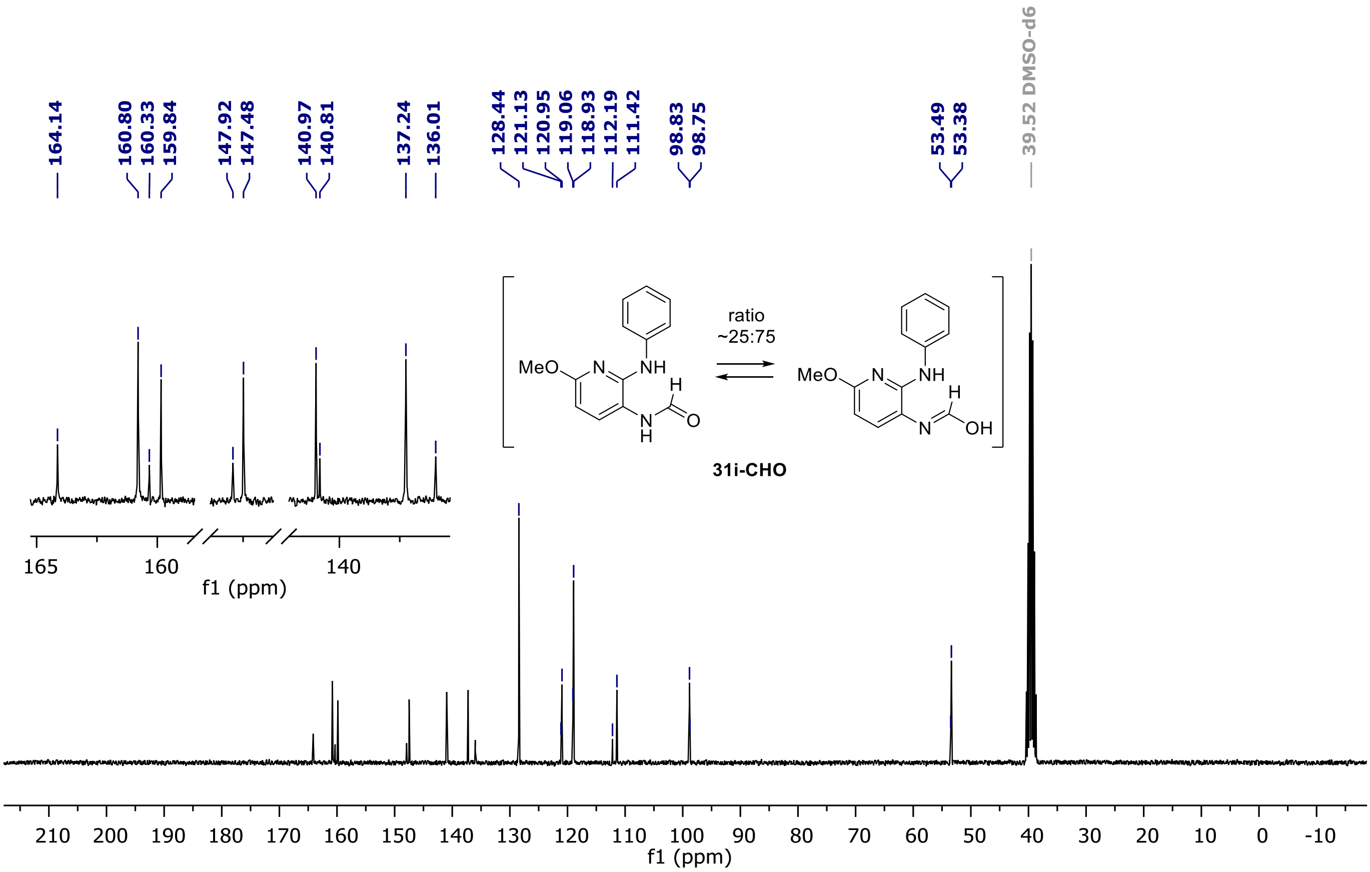

Figure S76. *$^{13}C\{^{1}H\}$ NMR (75 MHz, DMSO-d$_6$, 298 K) spectrum of **31i-CHO***

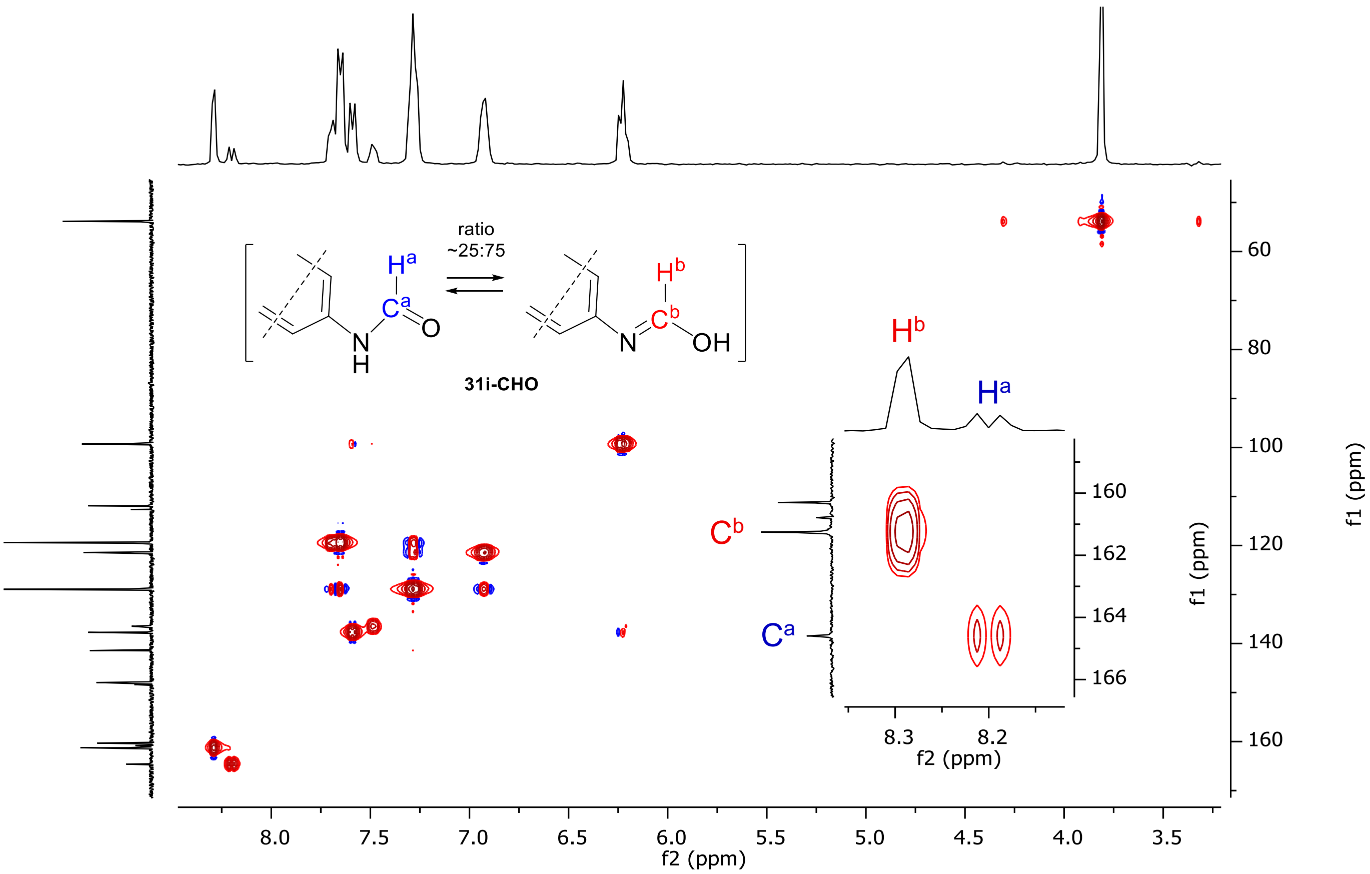

Figure S77. *HSQC (300 & 75 MHz, DMSO-d$_6$, 298 K) spectrum of **31i-CHO***

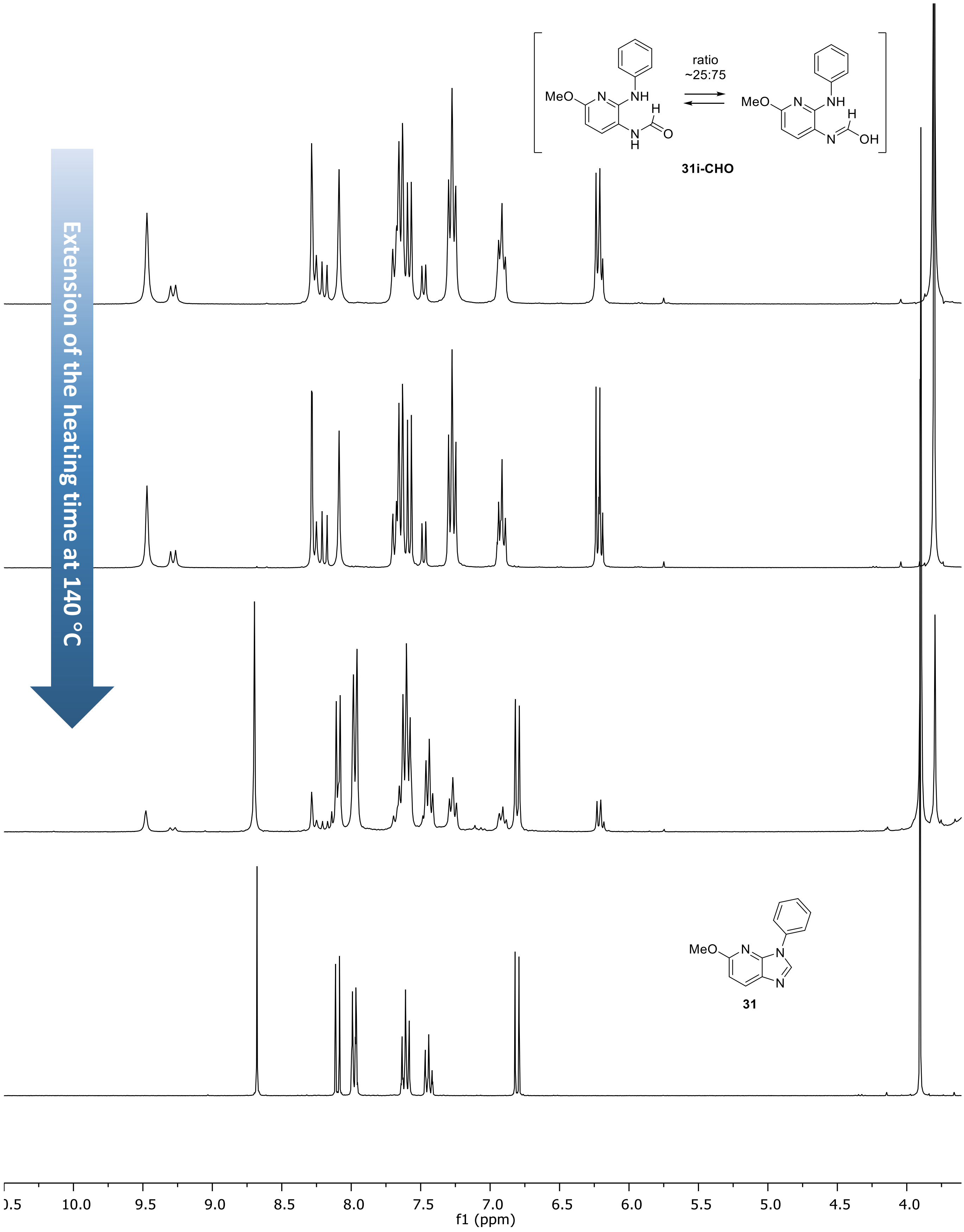

Figure S78. *^{1}H NMR (300 MHz, DMSO-d_6, 298 K) spectra of pristine **31i-CHO** (top), heated at 140 °C for 20 min (2nd), heated at 140 °C for 12 h (3rd), pure **31** (bottom)*

^1H and ^{13}C NMR spectra

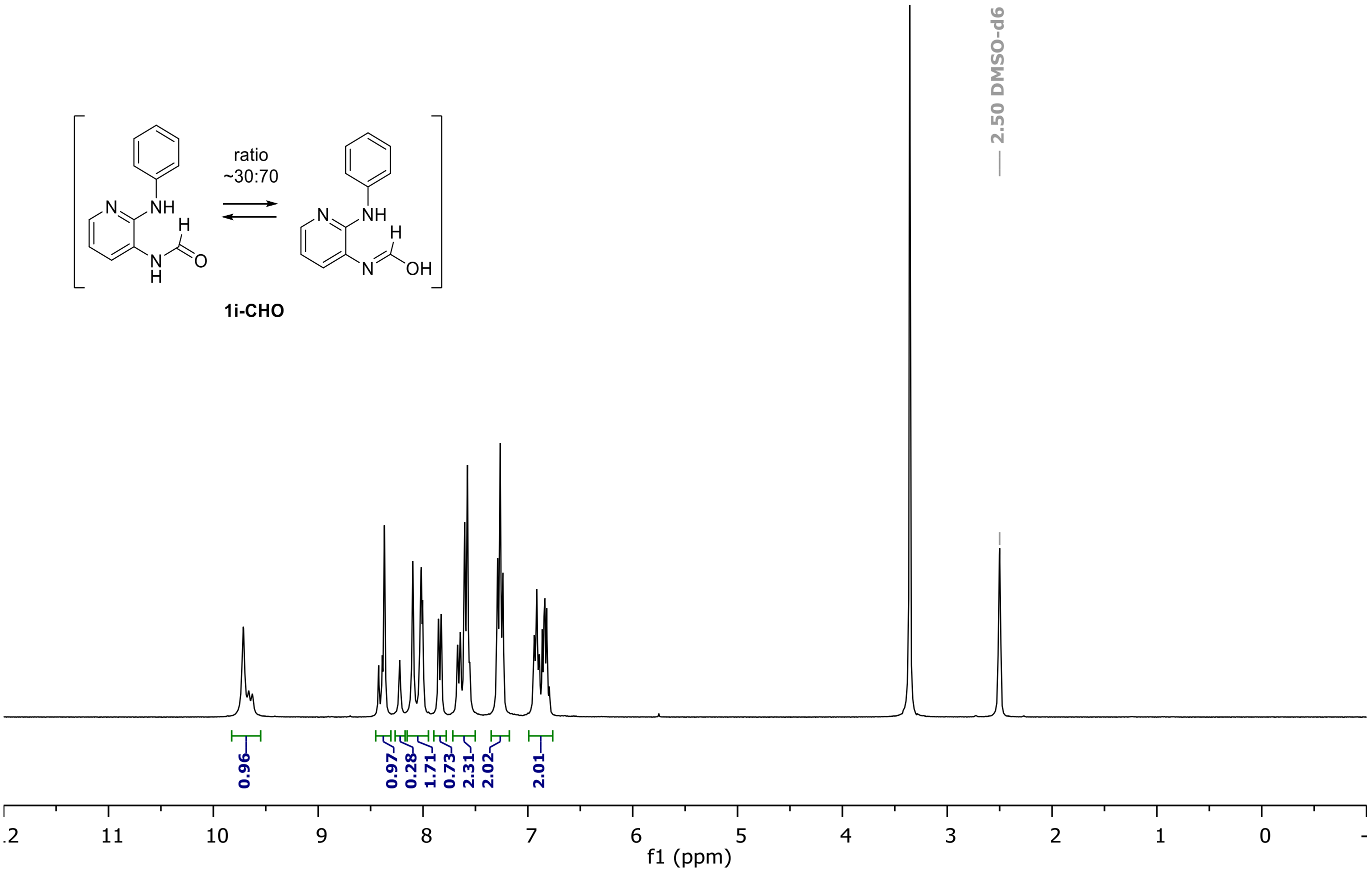

Figure S79. *^1H NMR (300 MHz, DMSO-d_6, 298 K) spectrum of* ***1i-CHO***

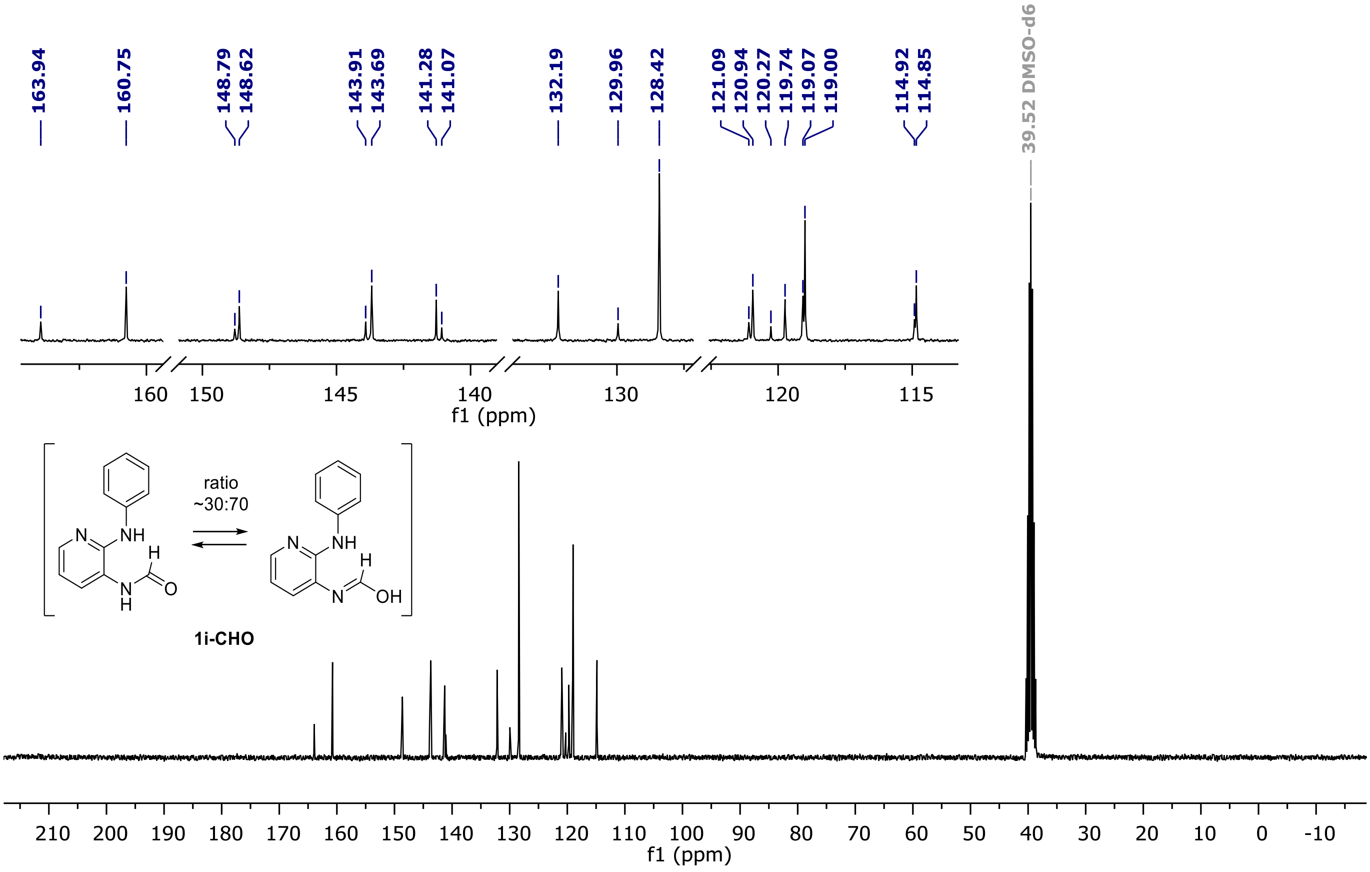

Figure S80. *$^{13}C\{^1H\}$ NMR (75 MHz, DMSO-d_6, 298 K) spectrum of* ***1i-CHO***

^{1}H and ^{13}C NMR spectra

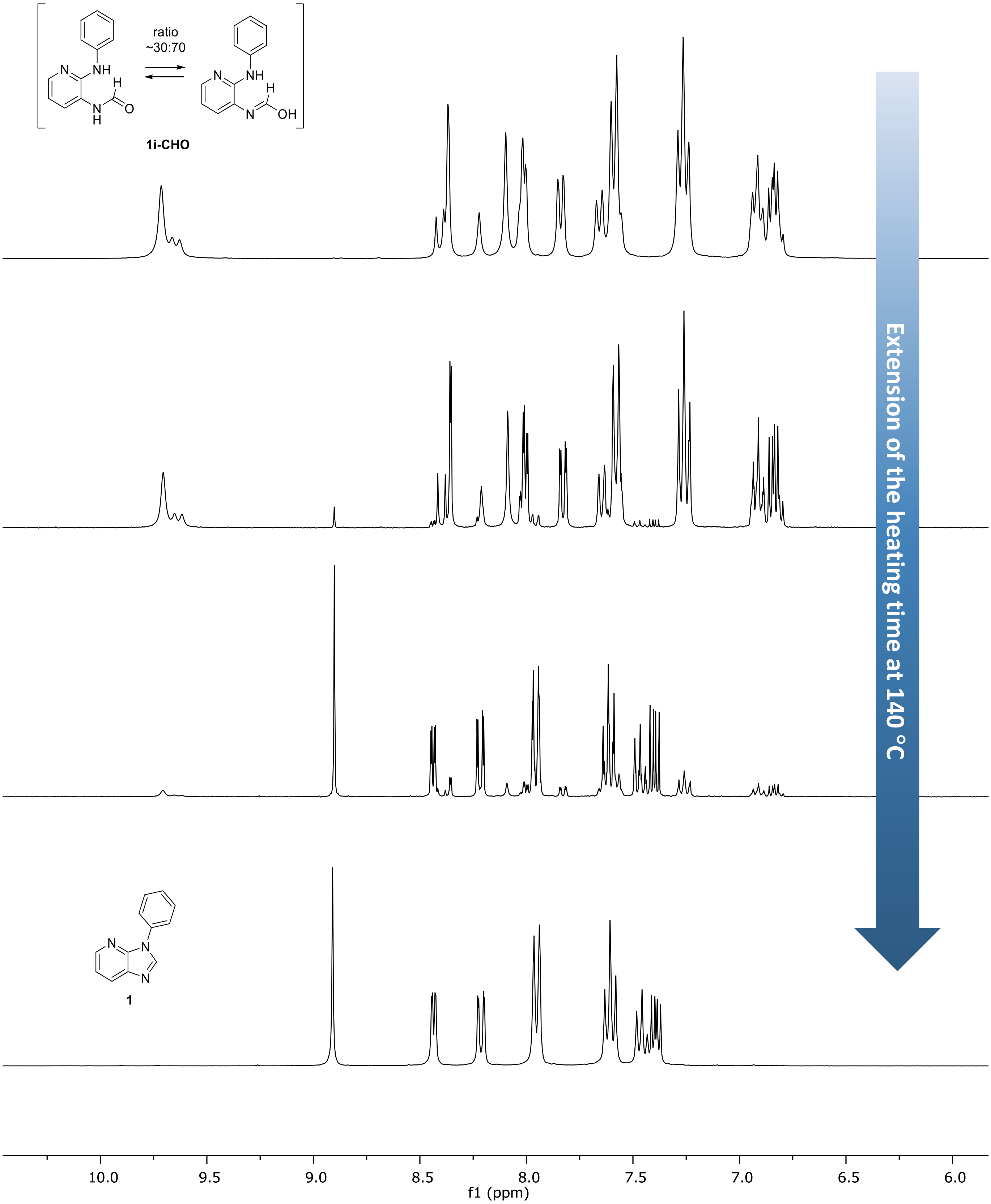

Figure S81. *^{1}H NMR (300 MHz, DMSO-d$_6$, 298 K) spectra of pristine* ***1i-CHO*** *(top), heated at 140 °C for 20 min (2nd), heated at 140 °C for 2 h (3rd), heated at 140 °C for 12 h (bottom)*

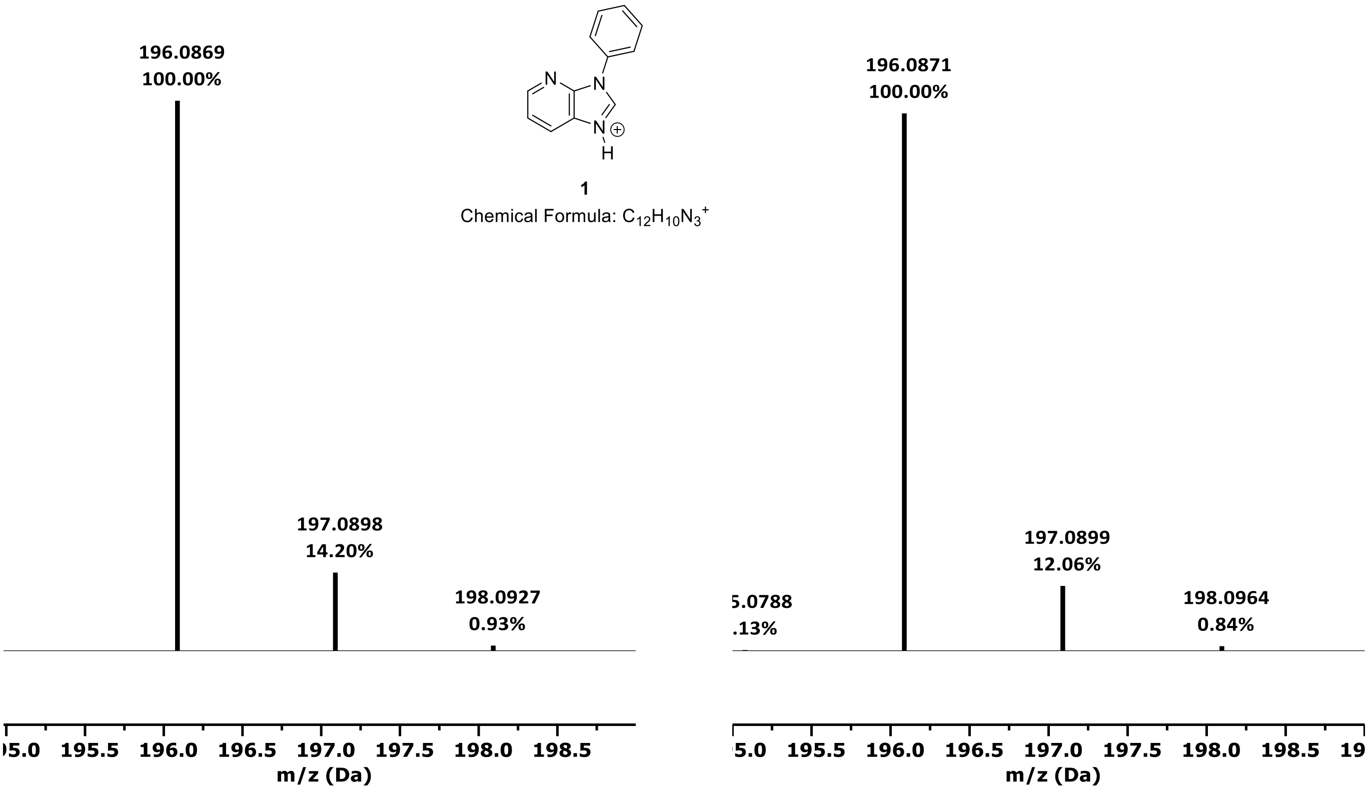

Figure S82. *ESI-HRMS spectrum of* ***1****, predicted (left) and measured (right)*

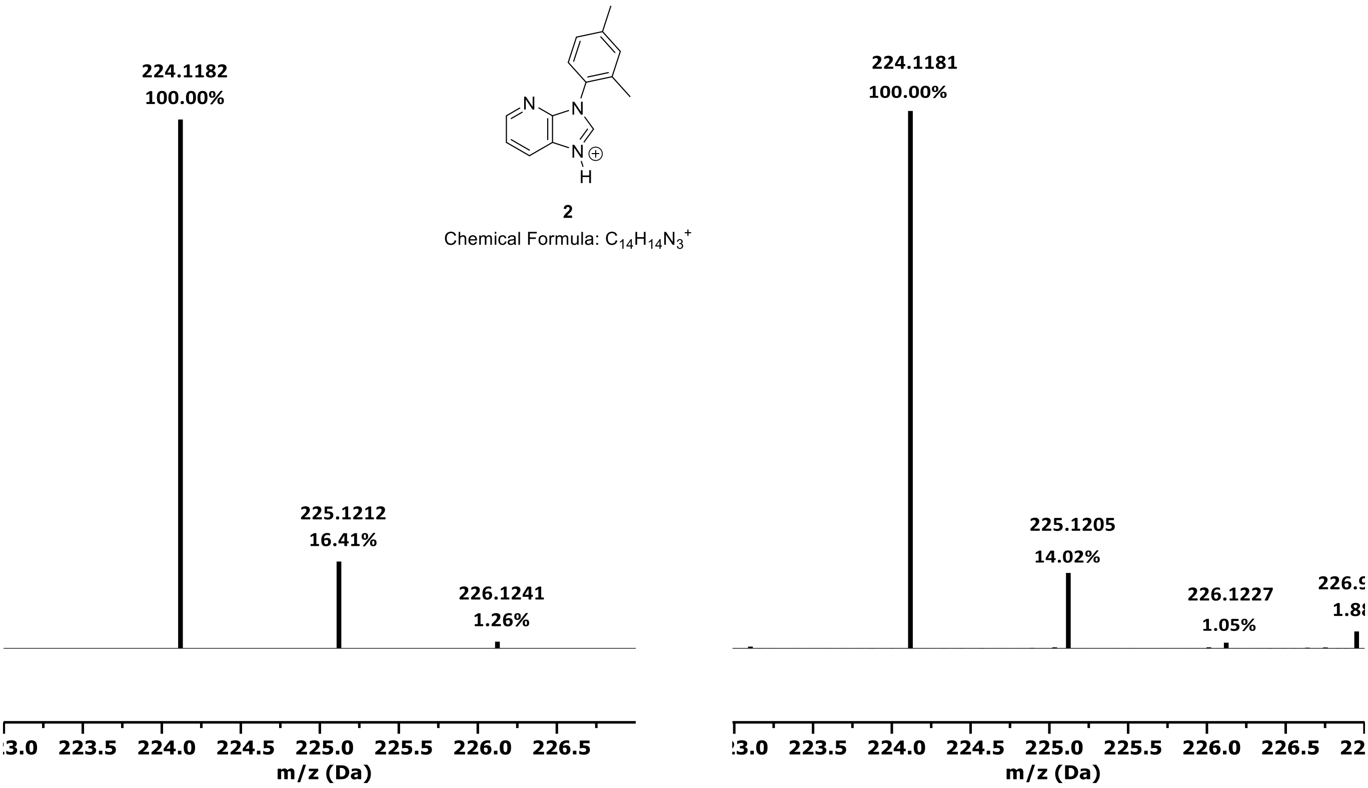

Figure S83. *ESI-HRMS spectrum of* ***2****, predicted (left) and measured (right, normalized)*

ESI-HRMS spectra

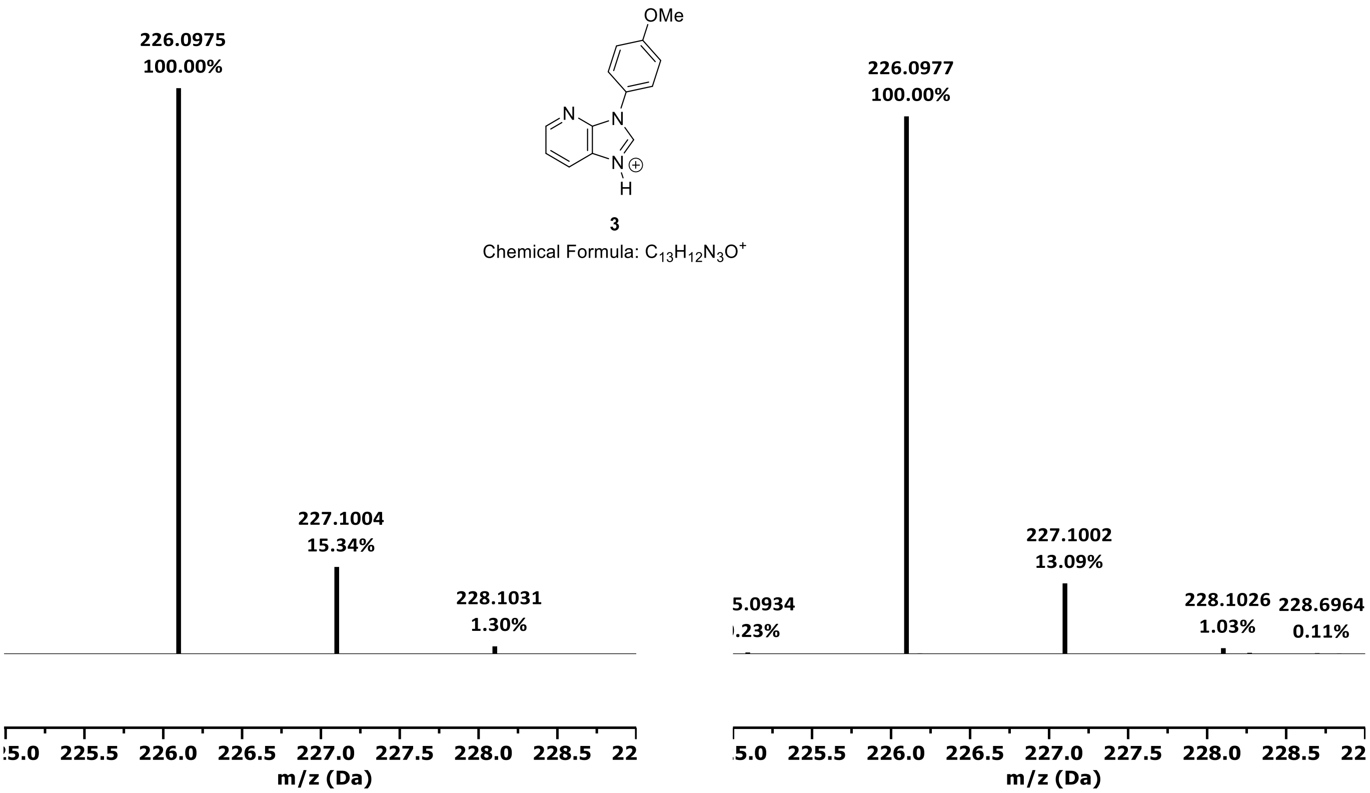

Figure S84. *ESI-HRMS spectrum of **3**, predicted (left) and measured (right)*

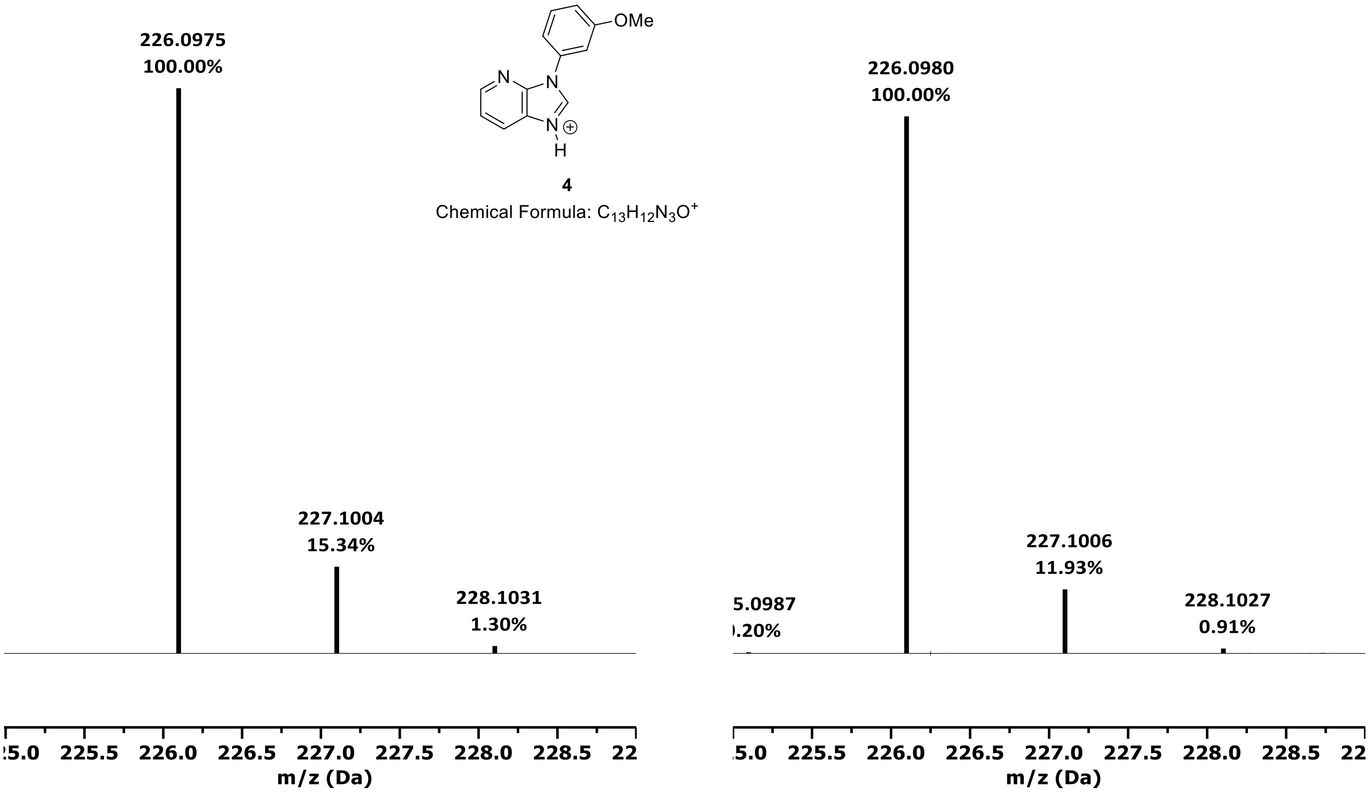

Figure S85. *ESI-HRMS spectrum of **4**, predicted (left) and measured (right)*

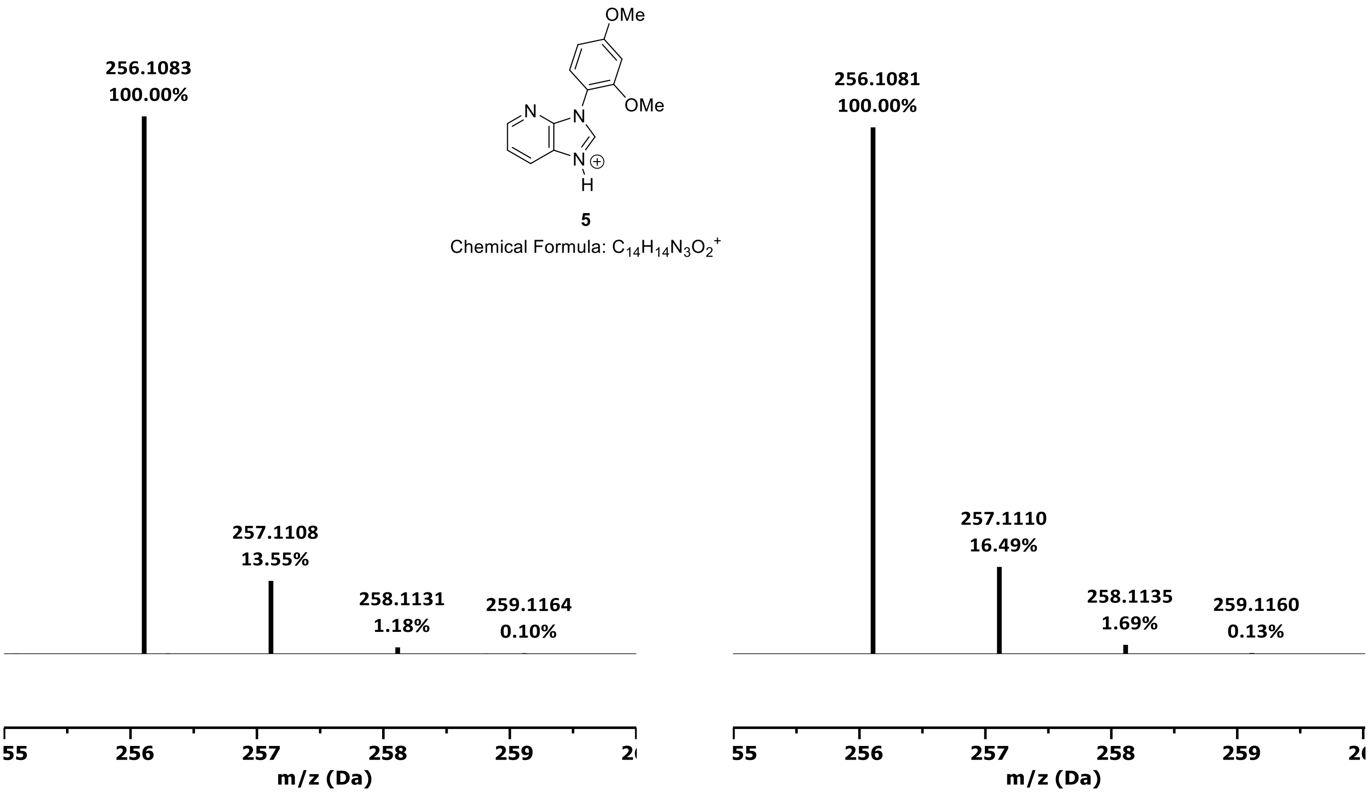

Figure S86. *ESI-HRMS spectrum of **5**, predicted (left) and measured (right)*

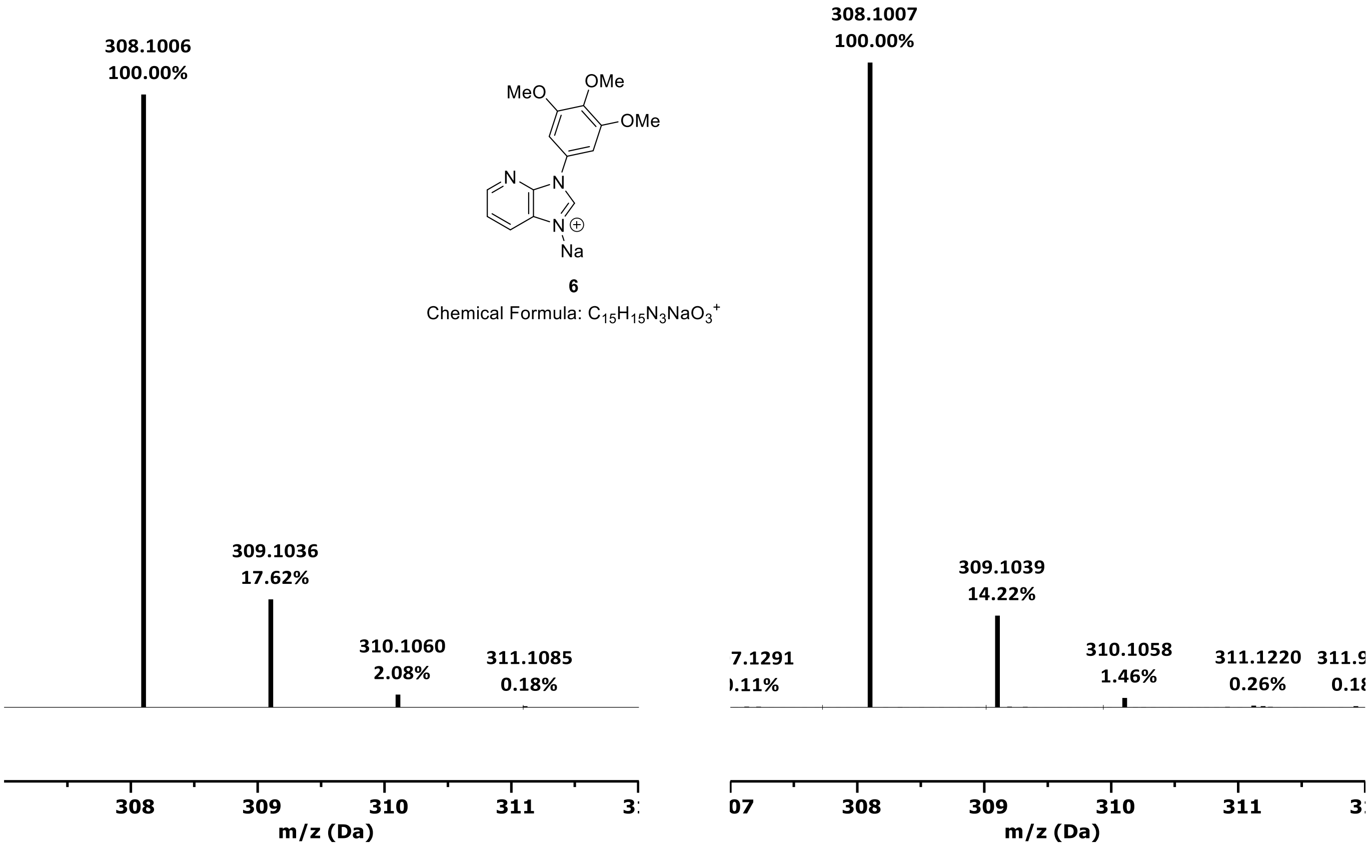

Figure S87. *ESI-HRMS spectrum of **6**, predicted (left) and measured (right)*

ESI-HRMS spectra

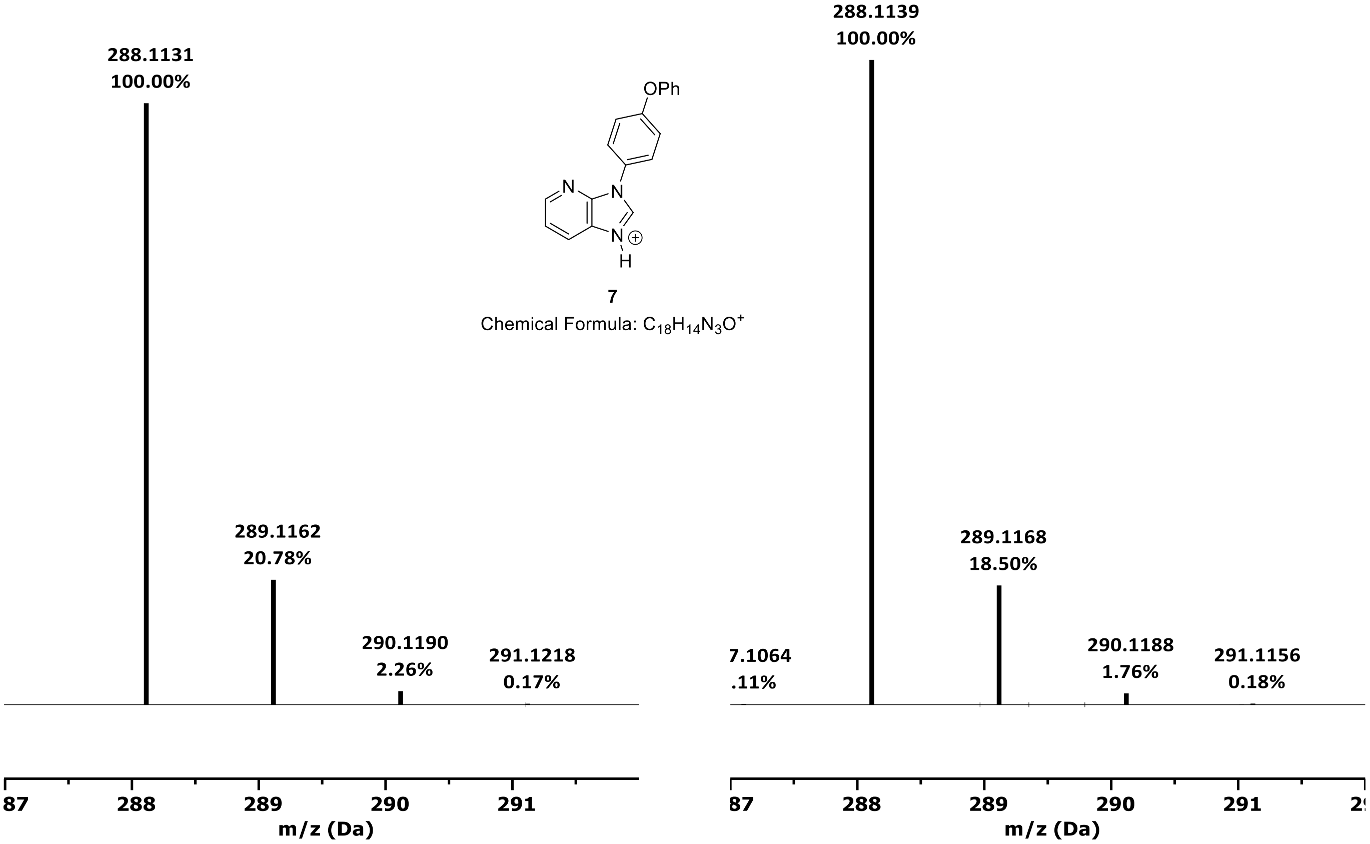

Figure S88. *ESI-HRMS spectrum of **7**, predicted (left) and measured (right)*

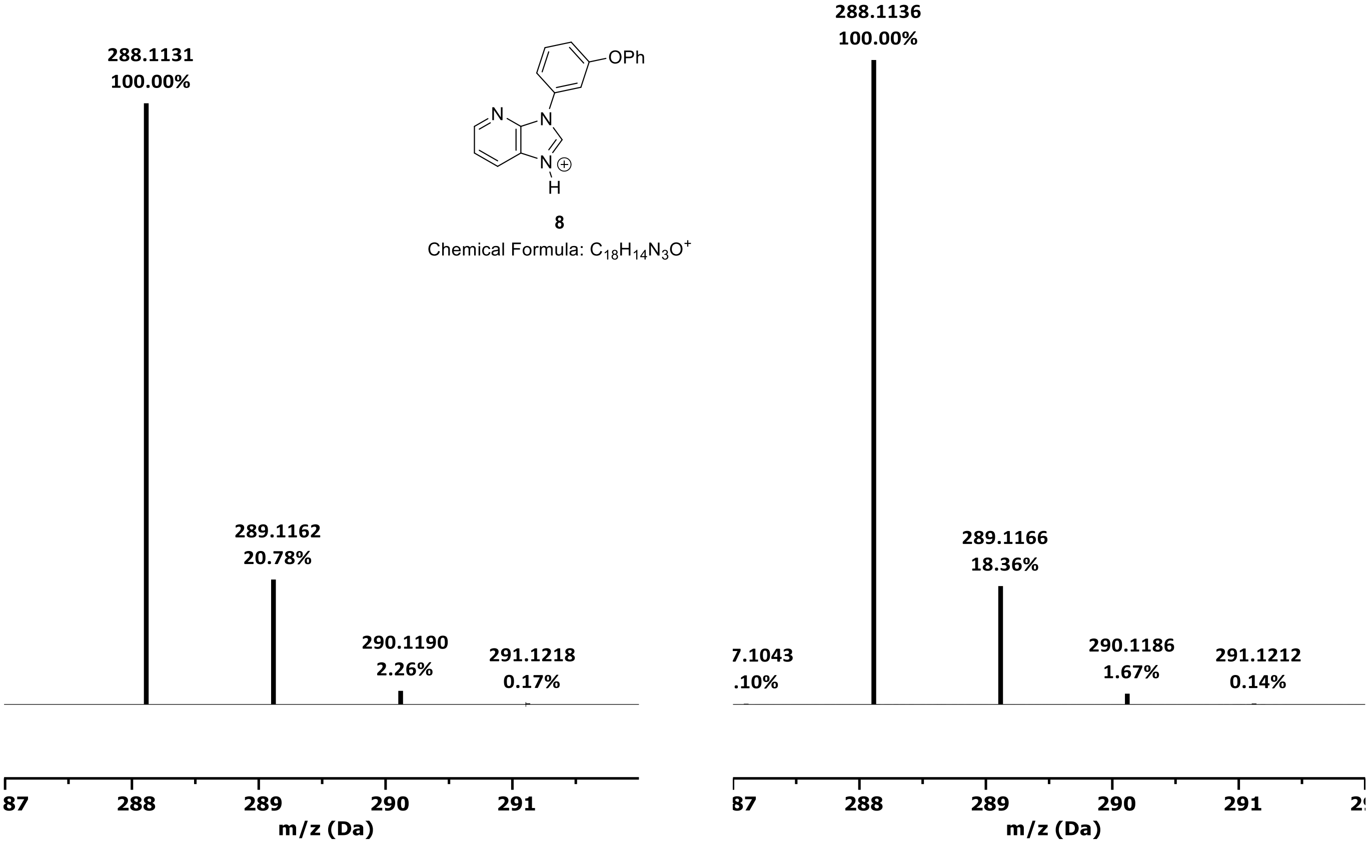

Figure S89. *ESI-HRMS spectrum of **8**, predicted (left) and measured (right)*

ESI-HRMS spectra

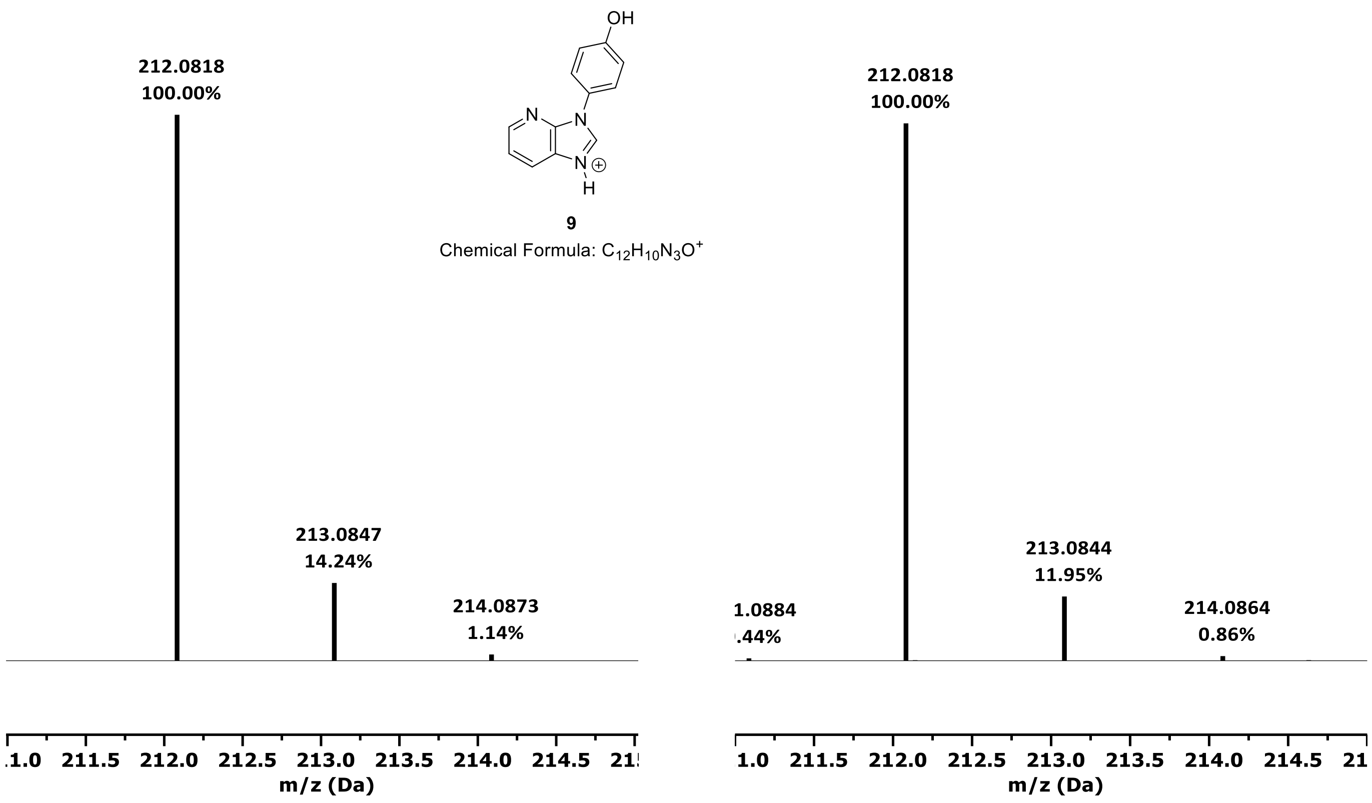

Figure S90. *ESI-HRMS spectrum of **9**, predicted (left) and measured (right)*

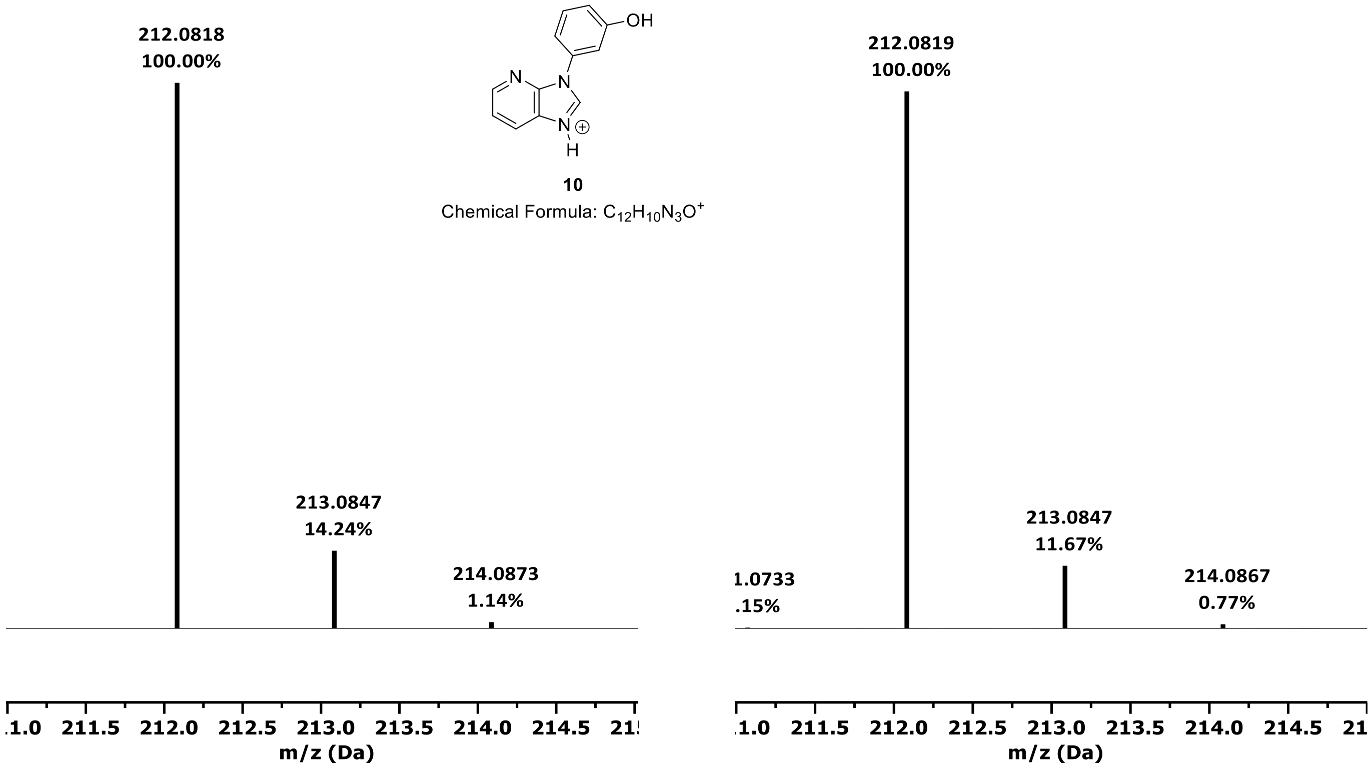

Figure S91. *ESI-HRMS spectrum of **10**, predicted (left) and measured (right)*

ESI-HRMS spectra

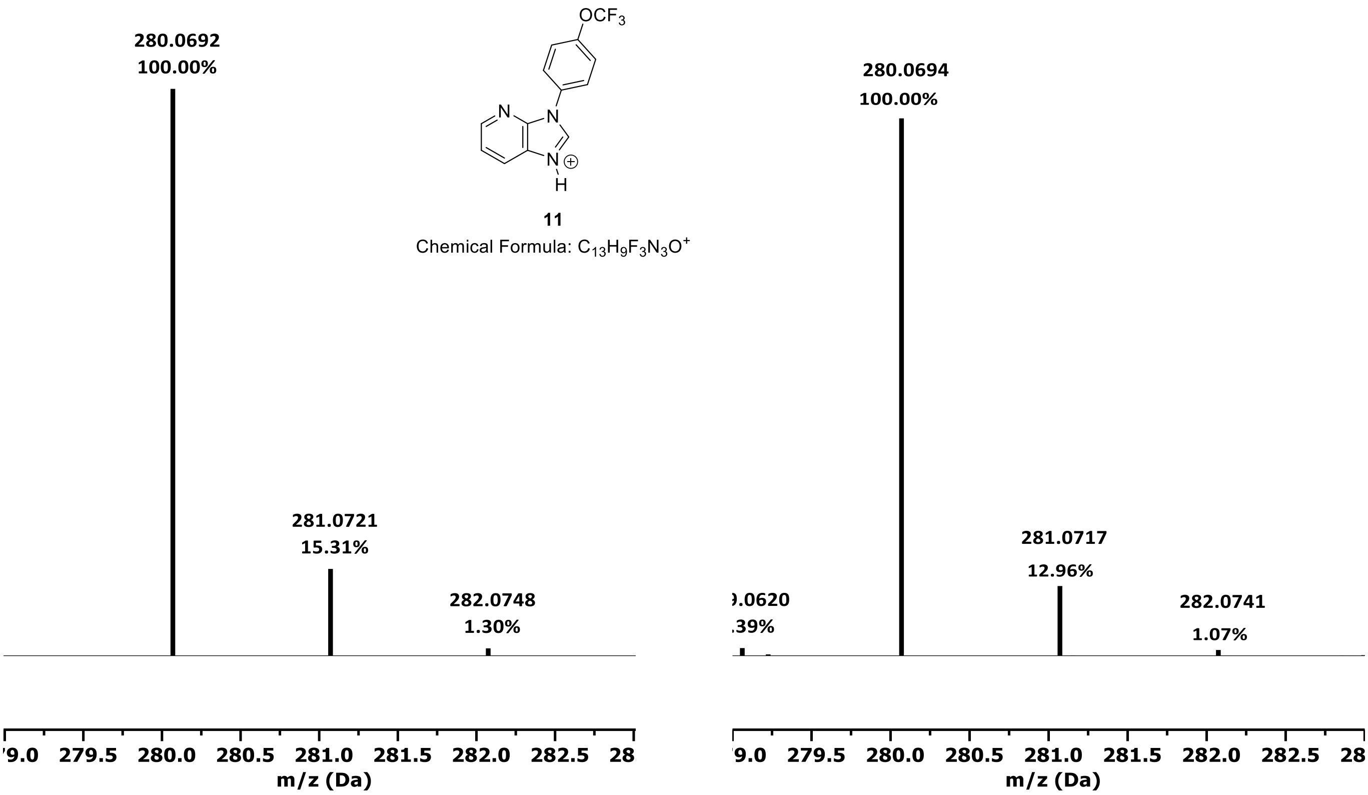

Figure S92. *ESI-HRMS spectrum of **11**, predicted (left) and measured (right, normalized)*

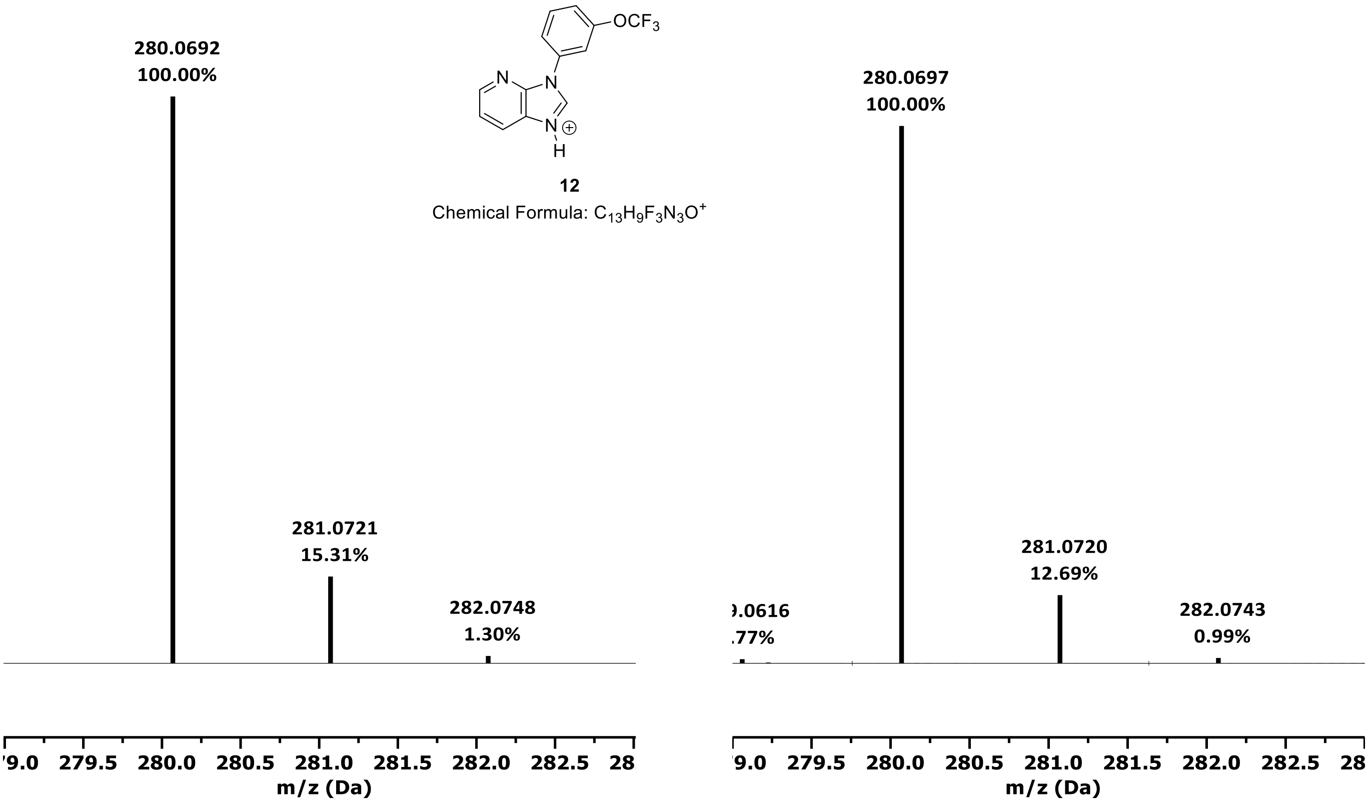

Figure S93. *ESI-HRMS spectrum of **12**, predicted (left) and measured (right)*

ESI-HRMS spectra

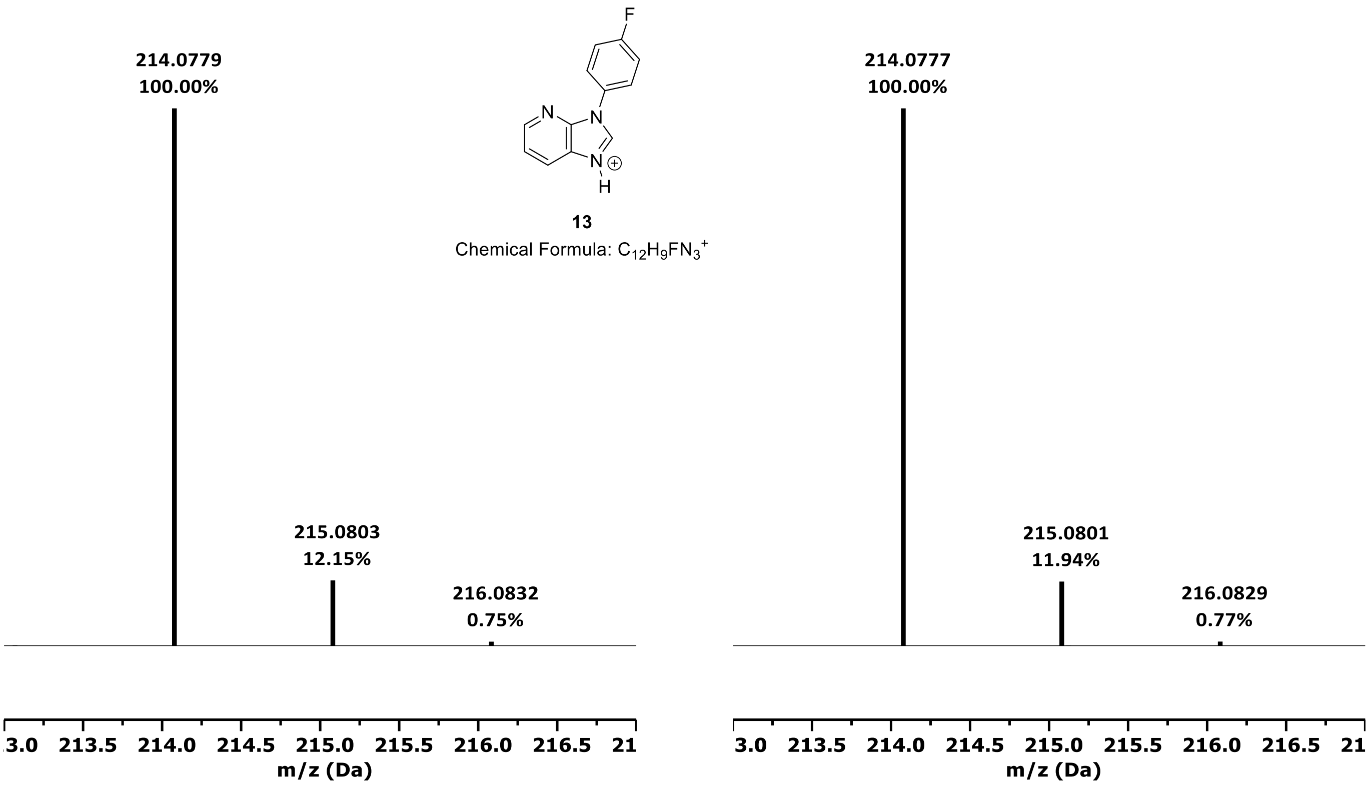

Figure S94. *ESI-HRMS spectrum of **13**, predicted (left) and measured (right)*

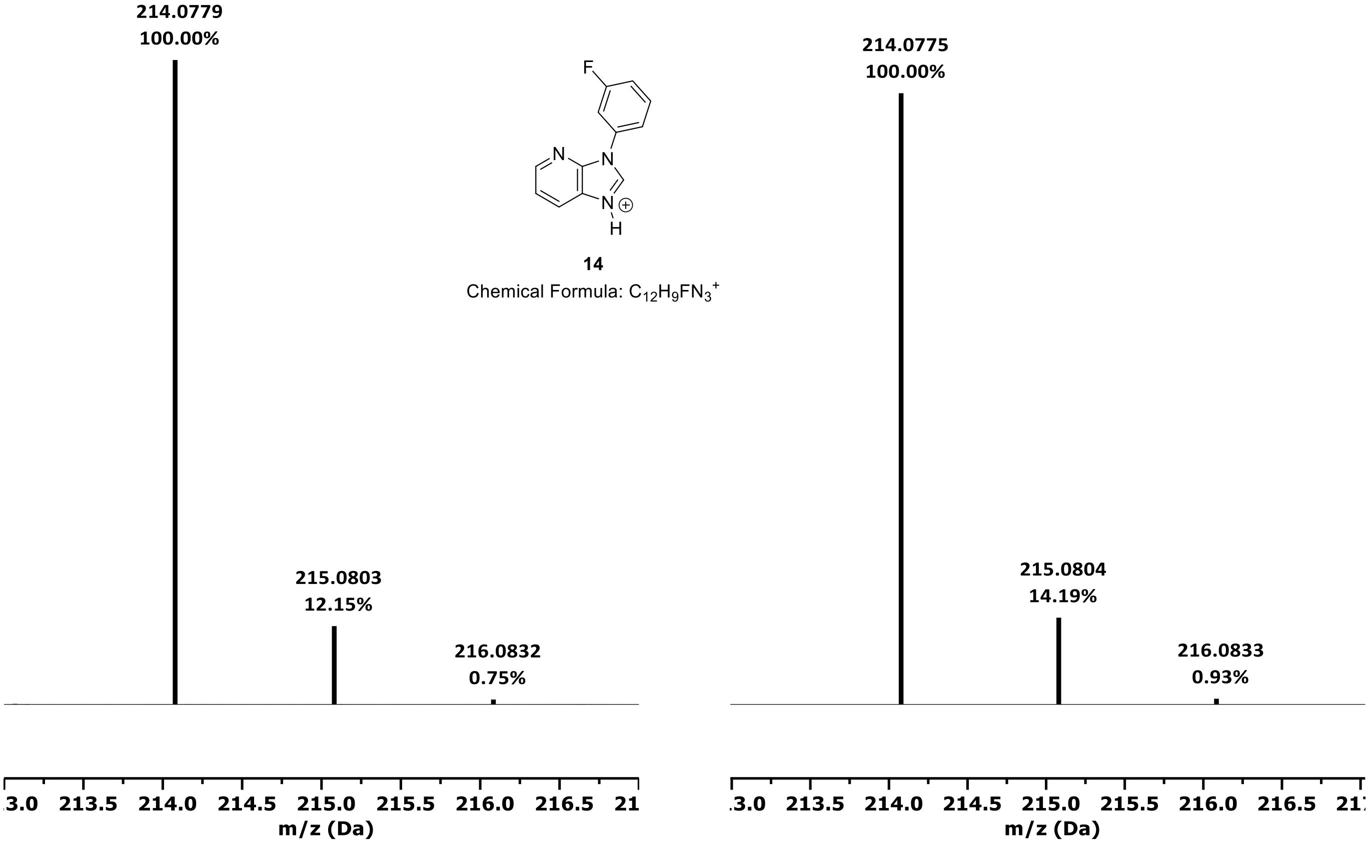

Figure S95. *ESI-HRMS spectrum of **14**, predicted (left) and measured (right)*

ESI-HRMS spectra

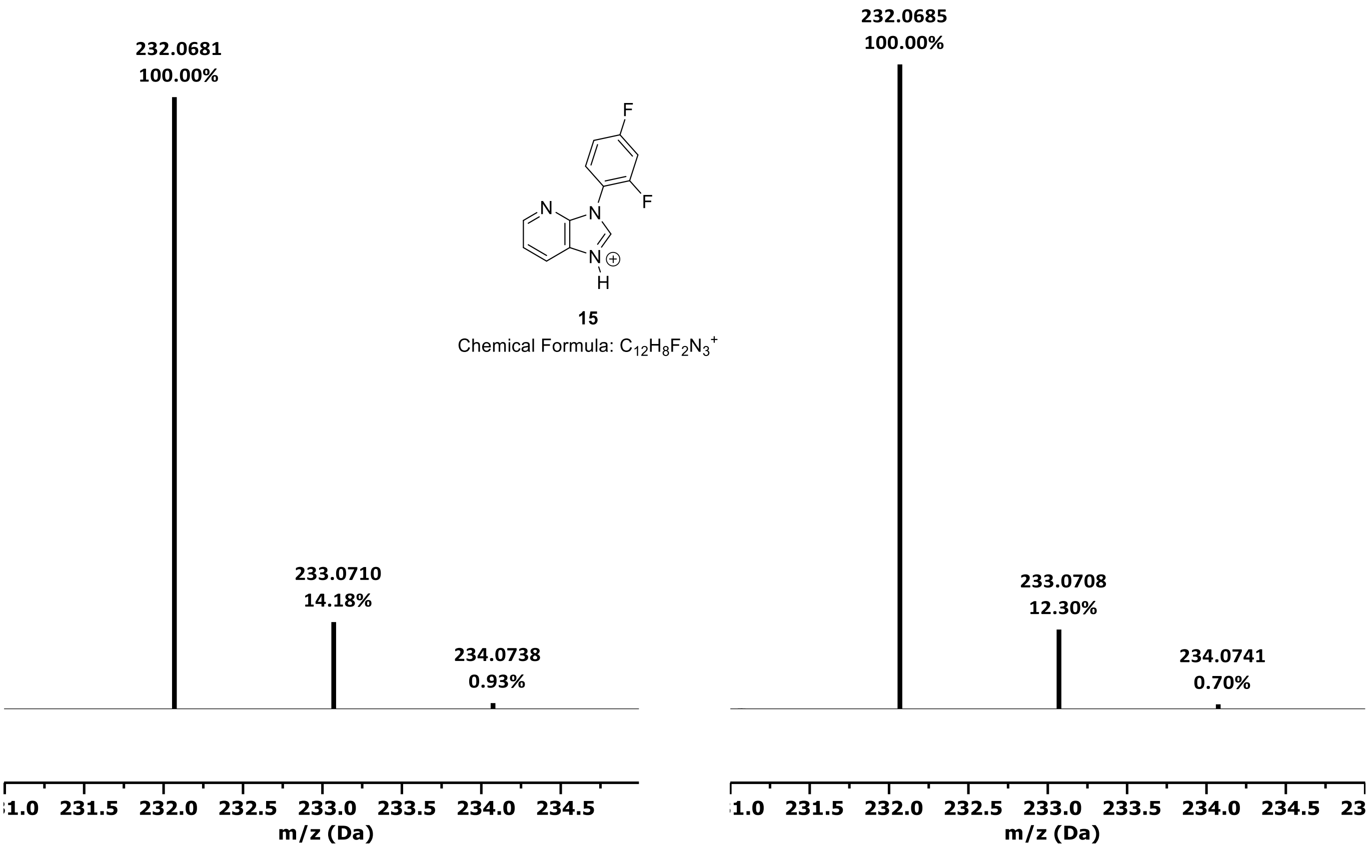

Figure S96. *ESI-HRMS spectrum of **15**, predicted (left) and measured (right)*

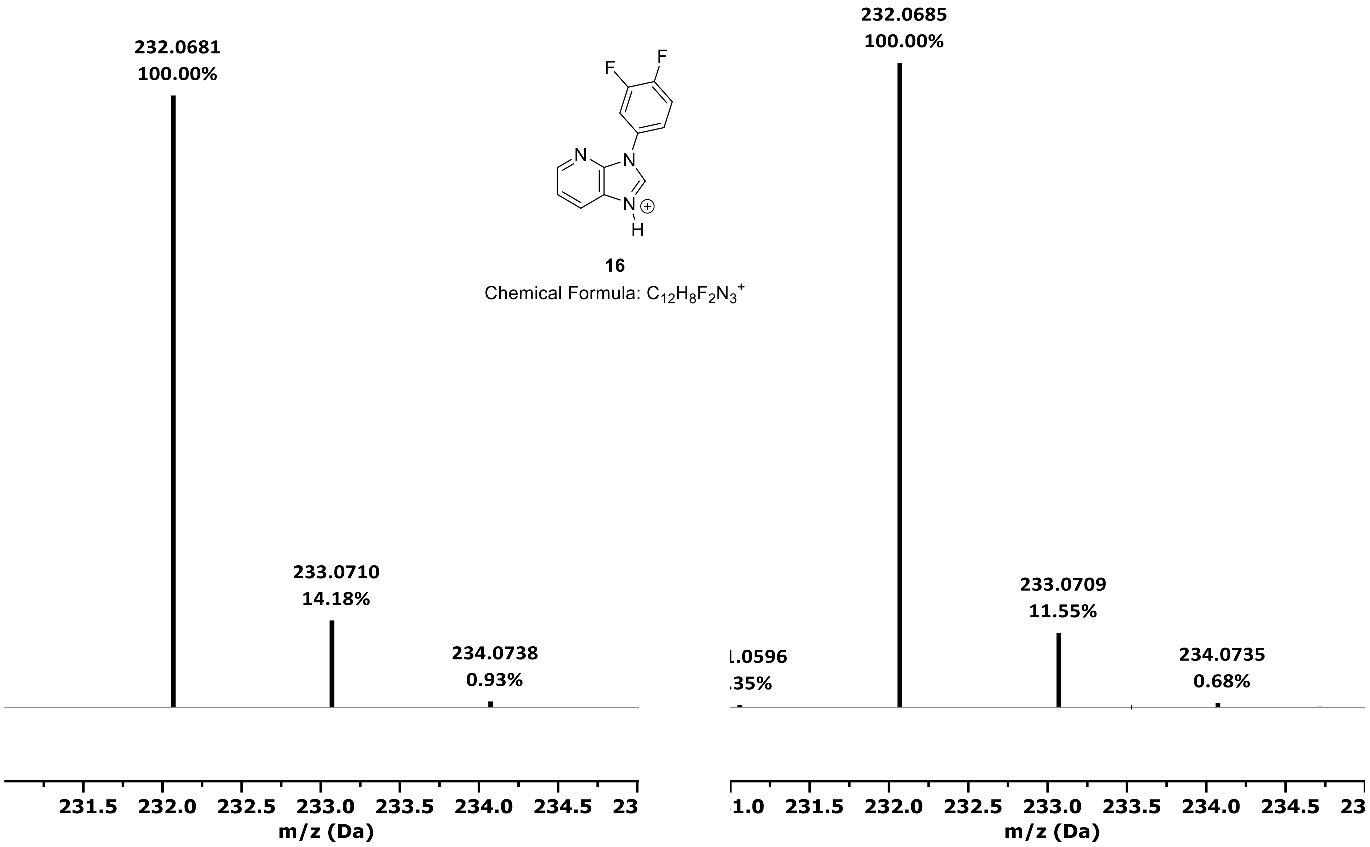

Figure S97. *ESI-HRMS spectrum of **16**, predicted (left) and measured (right)*

ESI-HRMS spectra

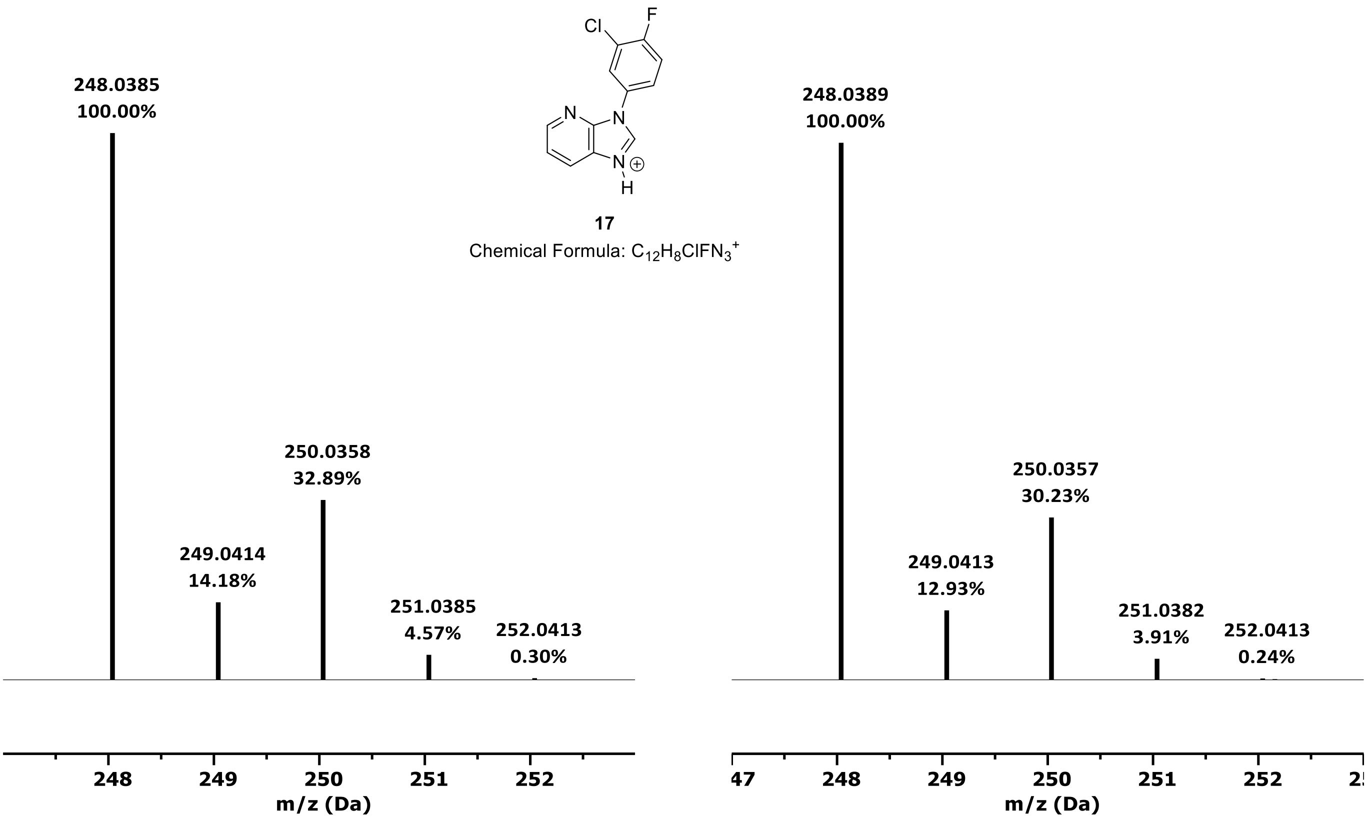

Figure S98. *ESI-HRMS spectrum of **17**, predicted (left) and measured (right)*

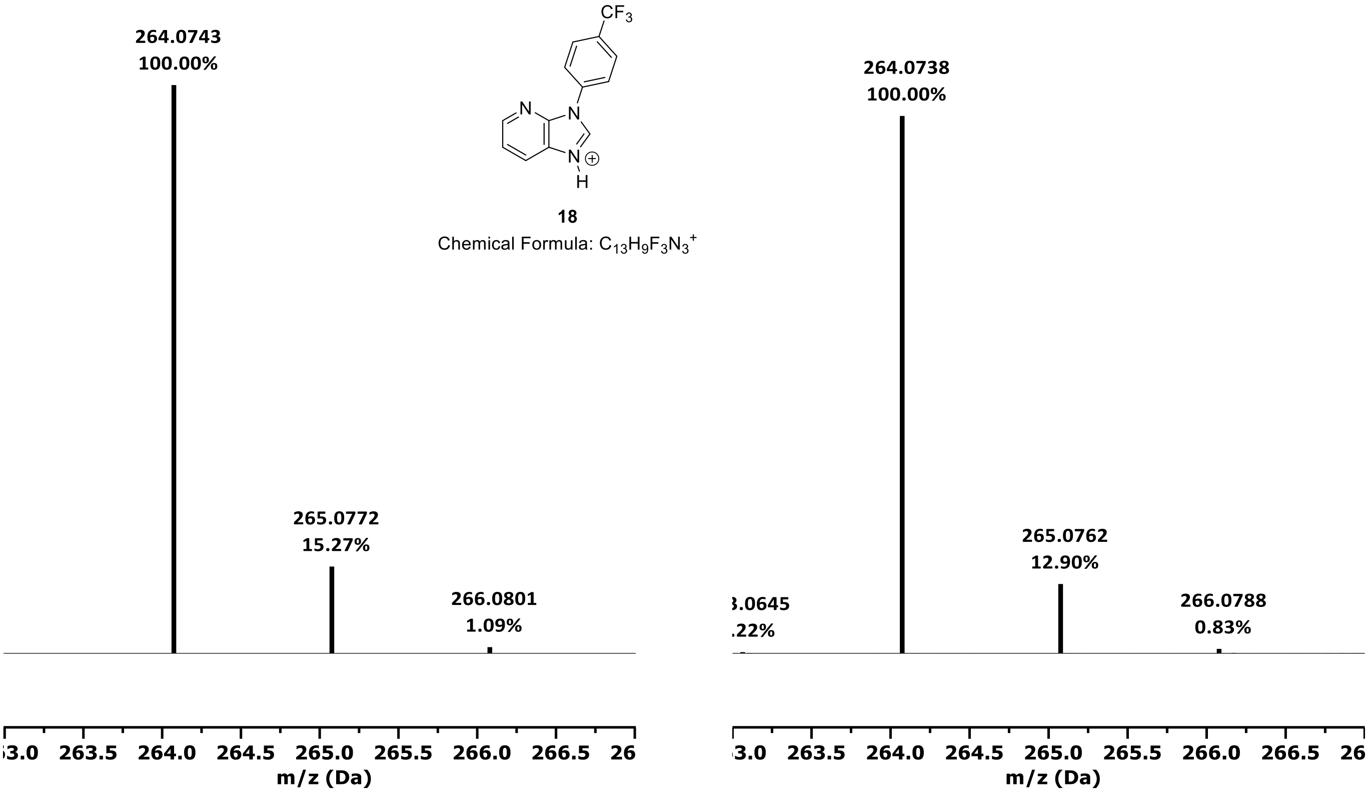

Figure S99. *ESI-HRMS spectrum of **18**, predicted (left) and measured (right)*

ESI-HRMS spectra

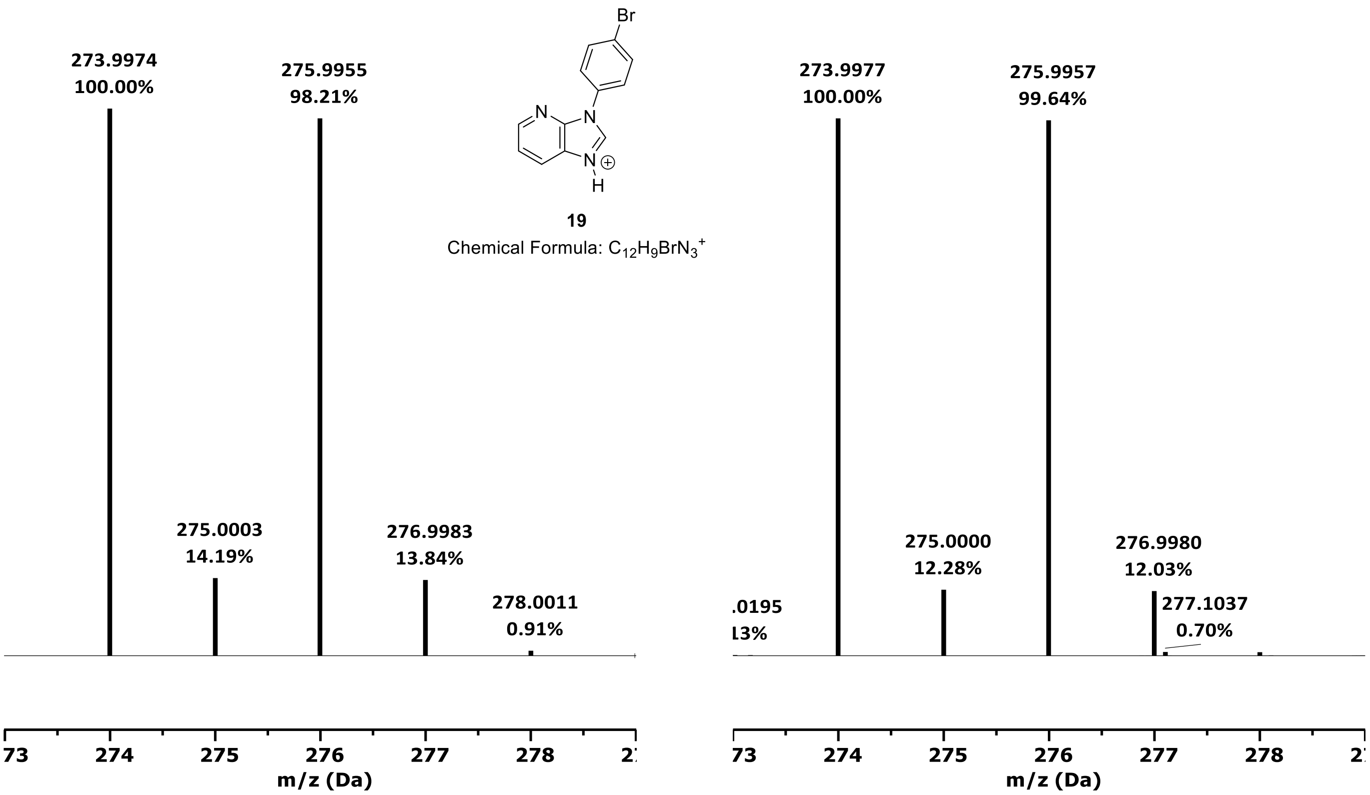

Figure S100. *ESI-HRMS spectrum of* ***19****, predicted (left) and measured (right)*

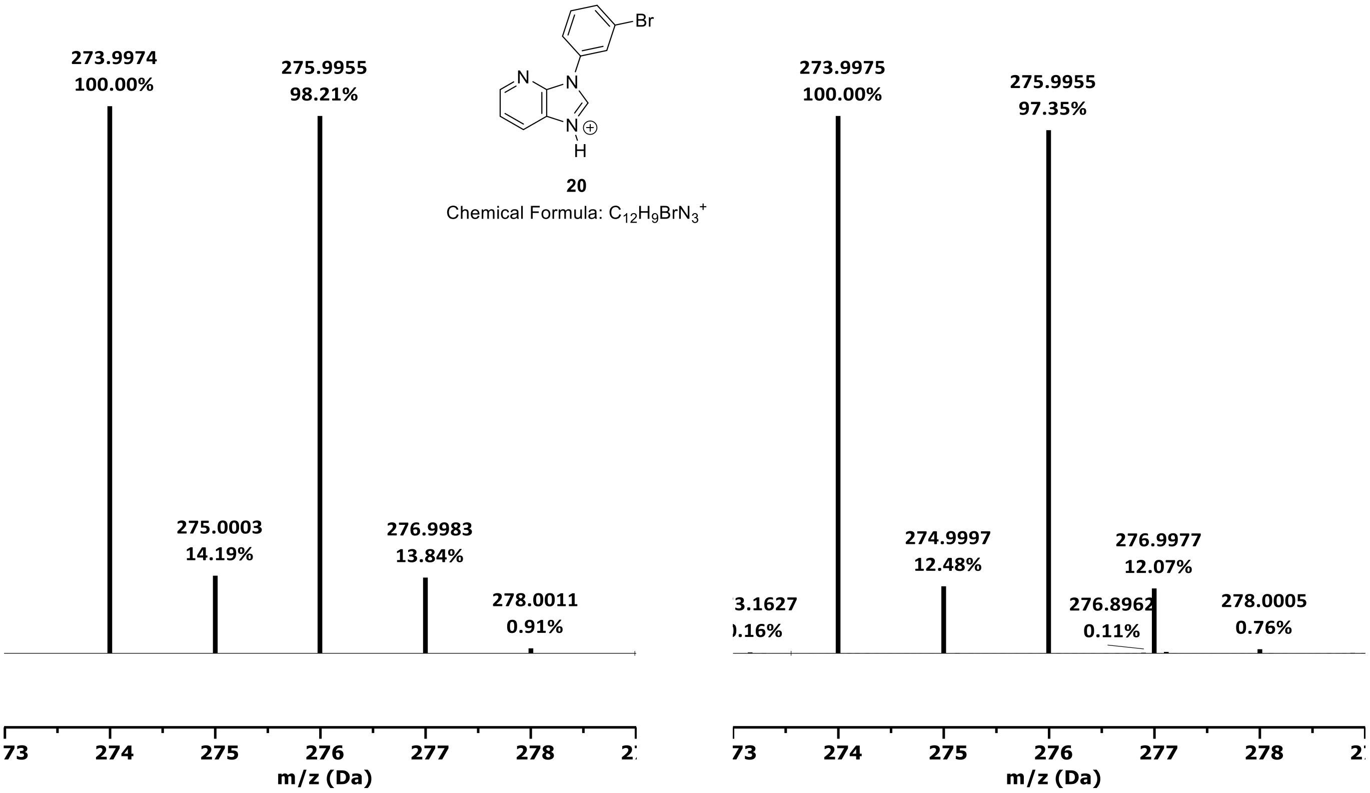

Figure S101. *ESI-HRMS spectrum of* ***20****, predicted (left) and measured (right)*

ESI-HRMS spectra

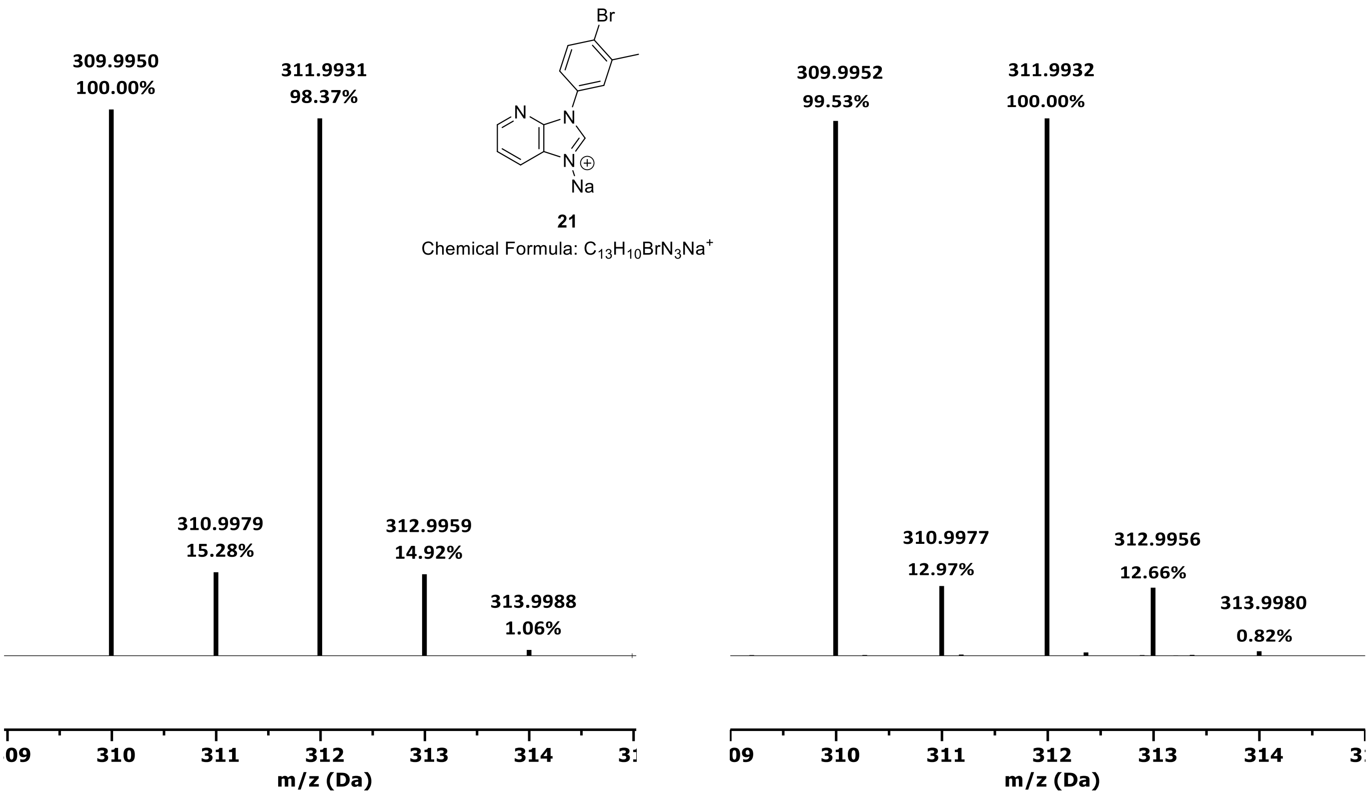

Figure S102. *ESI-HRMS spectrum of **21**, predicted (left) and measured (right, normalized)*

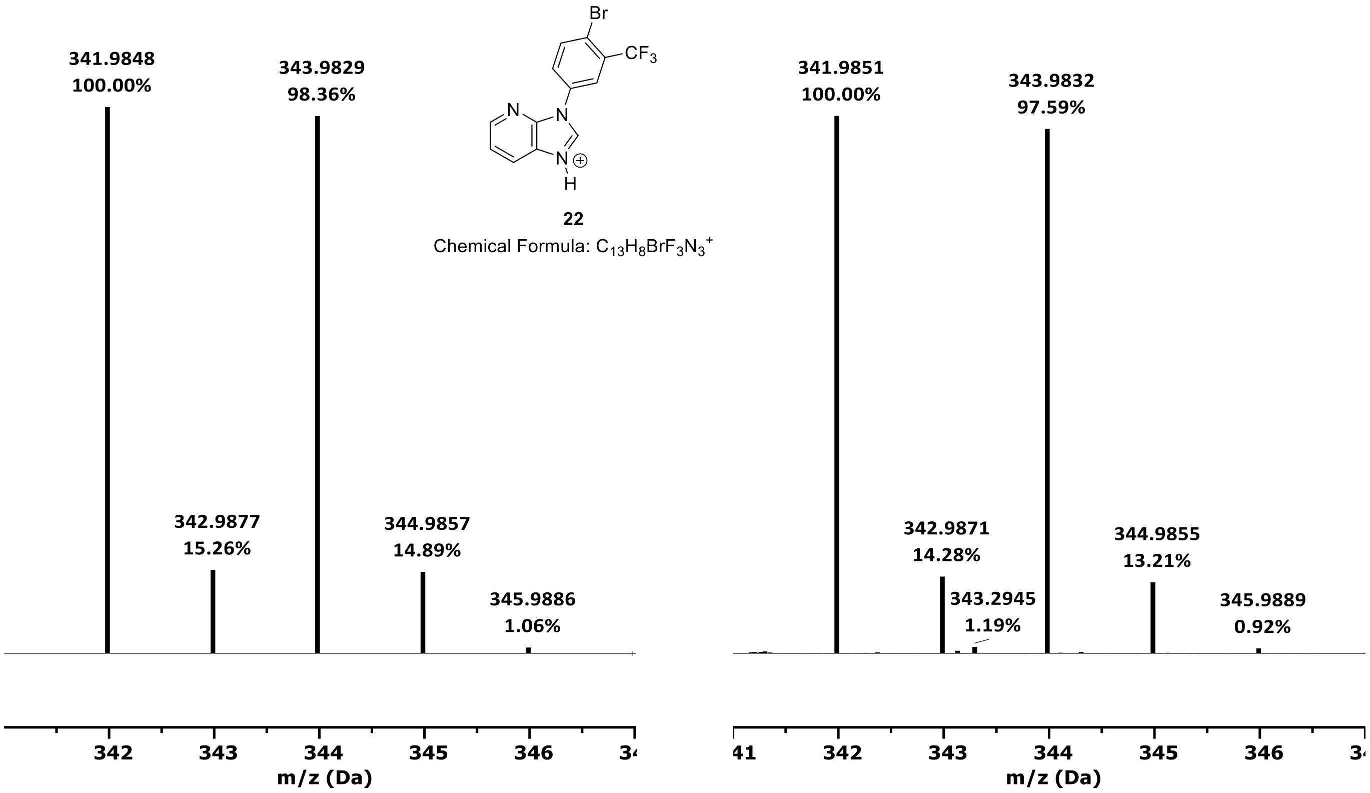

Figure S103. *ESI-HRMS spectrum of **22**, predicted (left) and measured (right)*

ESI-HRMS spectra

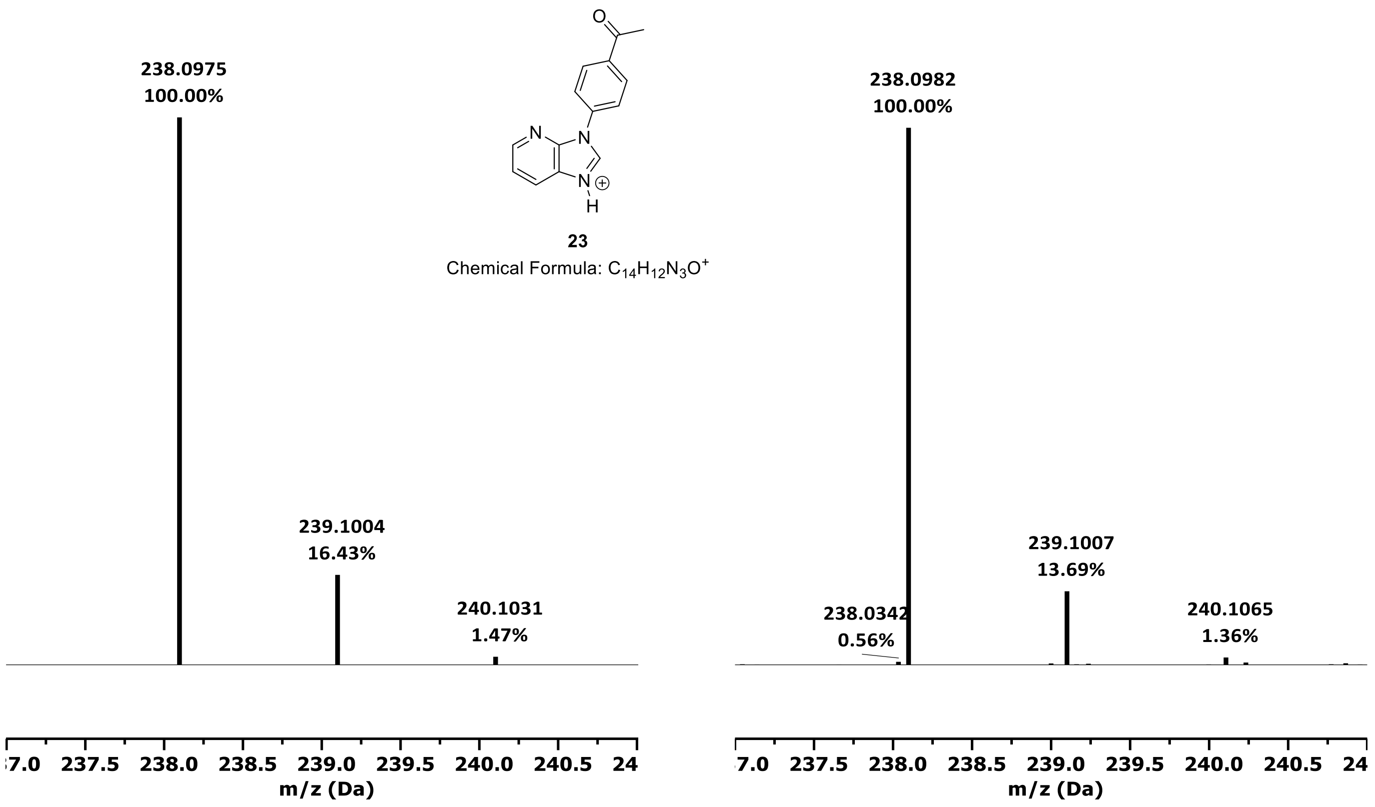

Figure S104. *ESI-HRMS spectrum of **23**, predicted (left) and measured (right)*

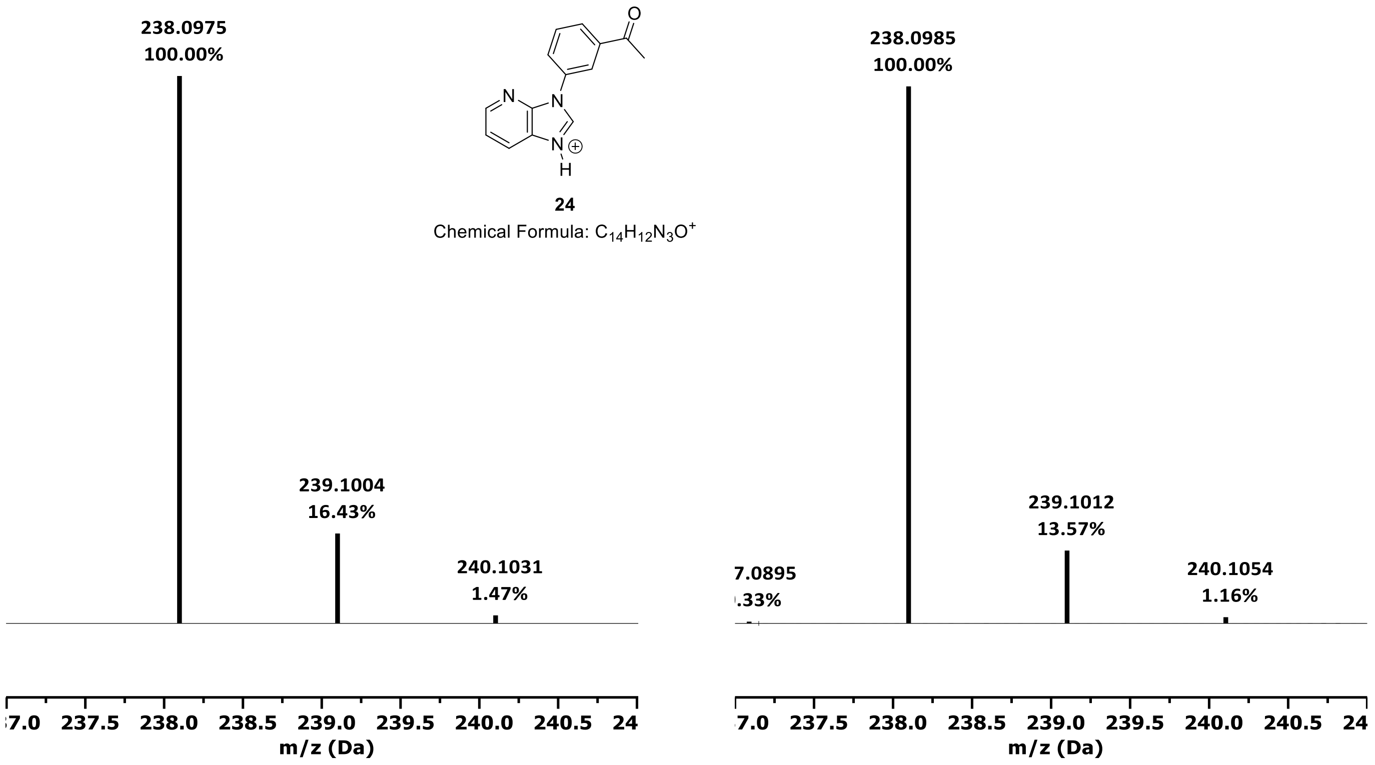

Figure S105. *ESI-HRMS spectrum of **24**, predicted (left) and measured (right)*

ESI-HRMS spectra

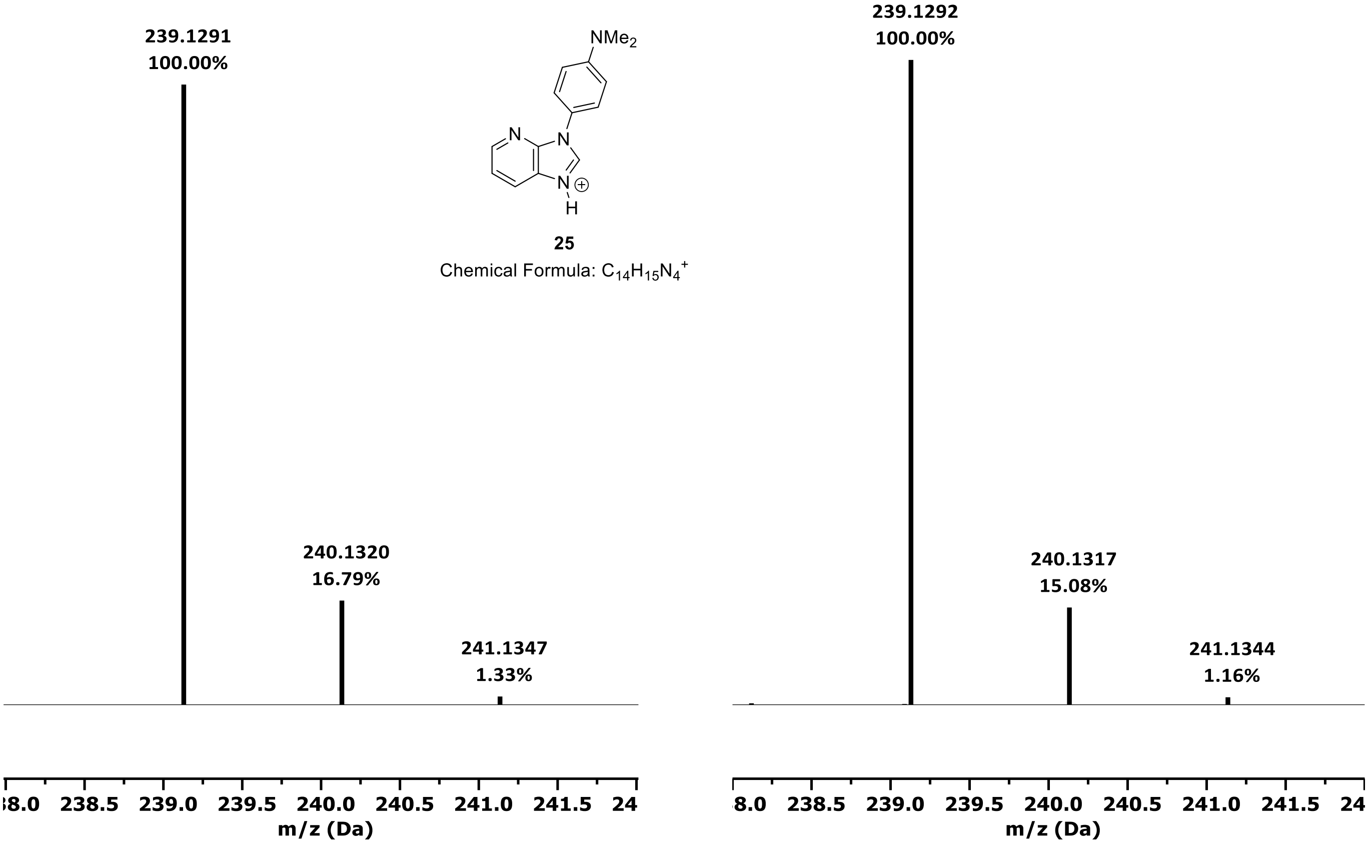

Figure S106. *ESI-HRMS spectrum of* ***25****, predicted (left) and measured (right)*

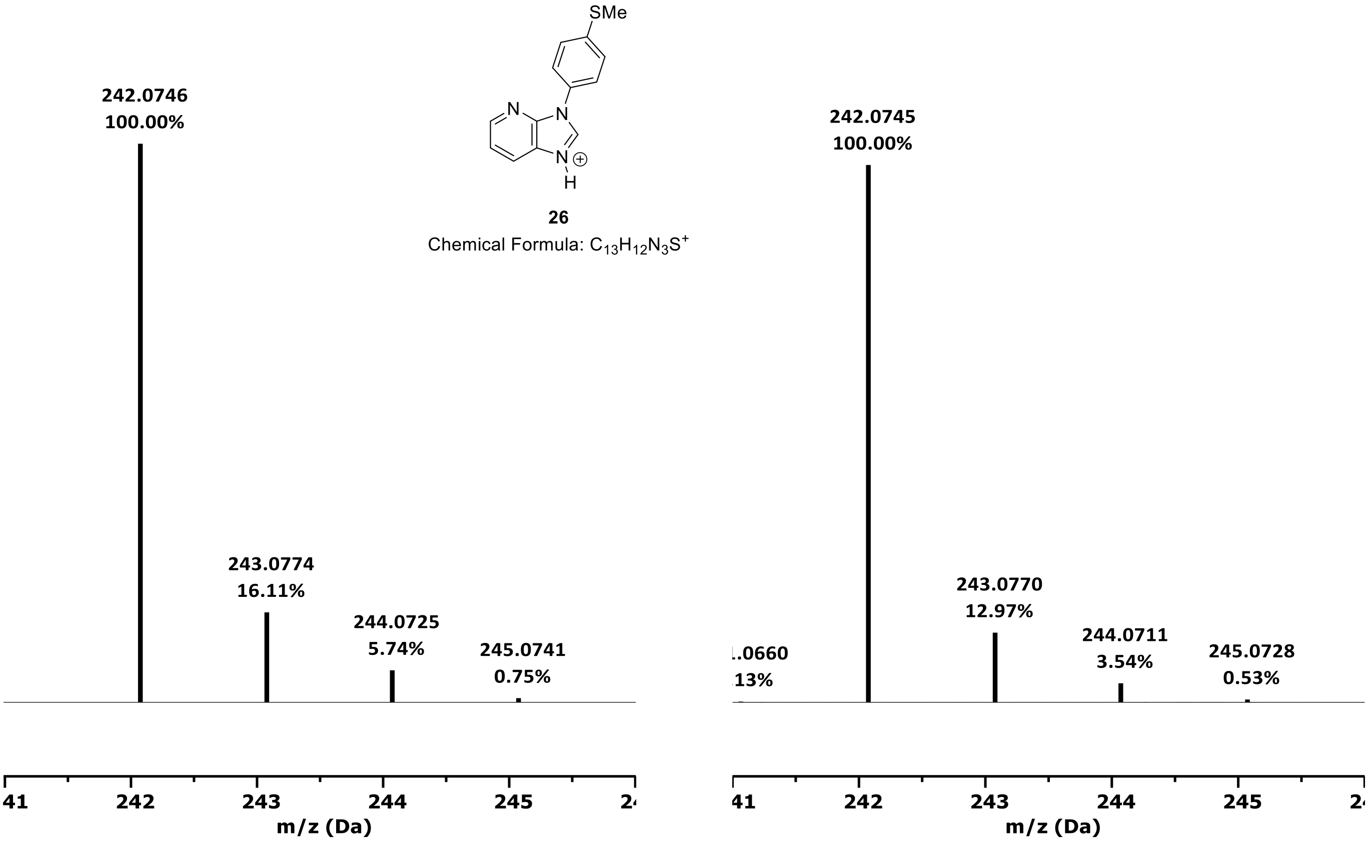

Figure S107. *ESI-HRMS spectrum of* ***26****, predicted (left) and measured (right)*

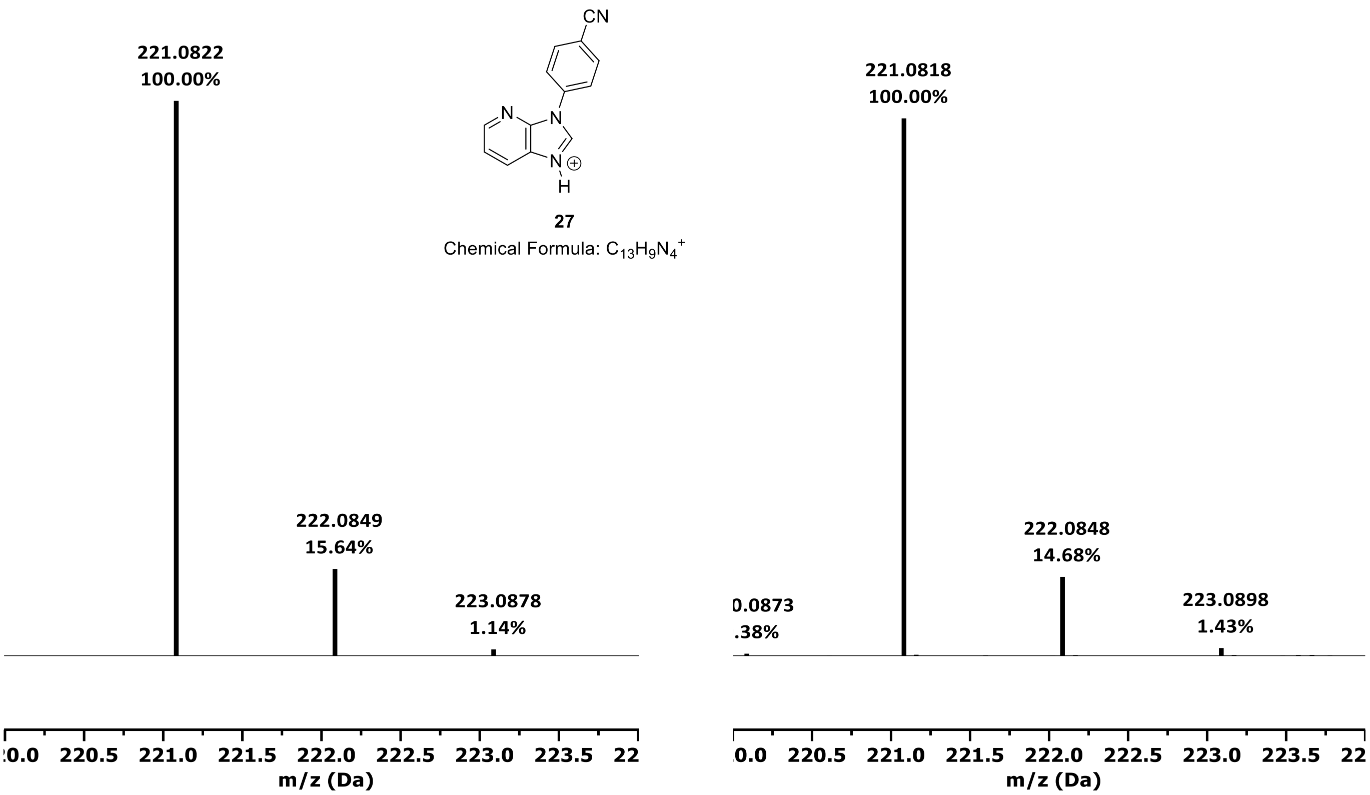

Figure S108. *ESI-HRMS spectrum of* ***27****, predicted (left) and measured (right)*

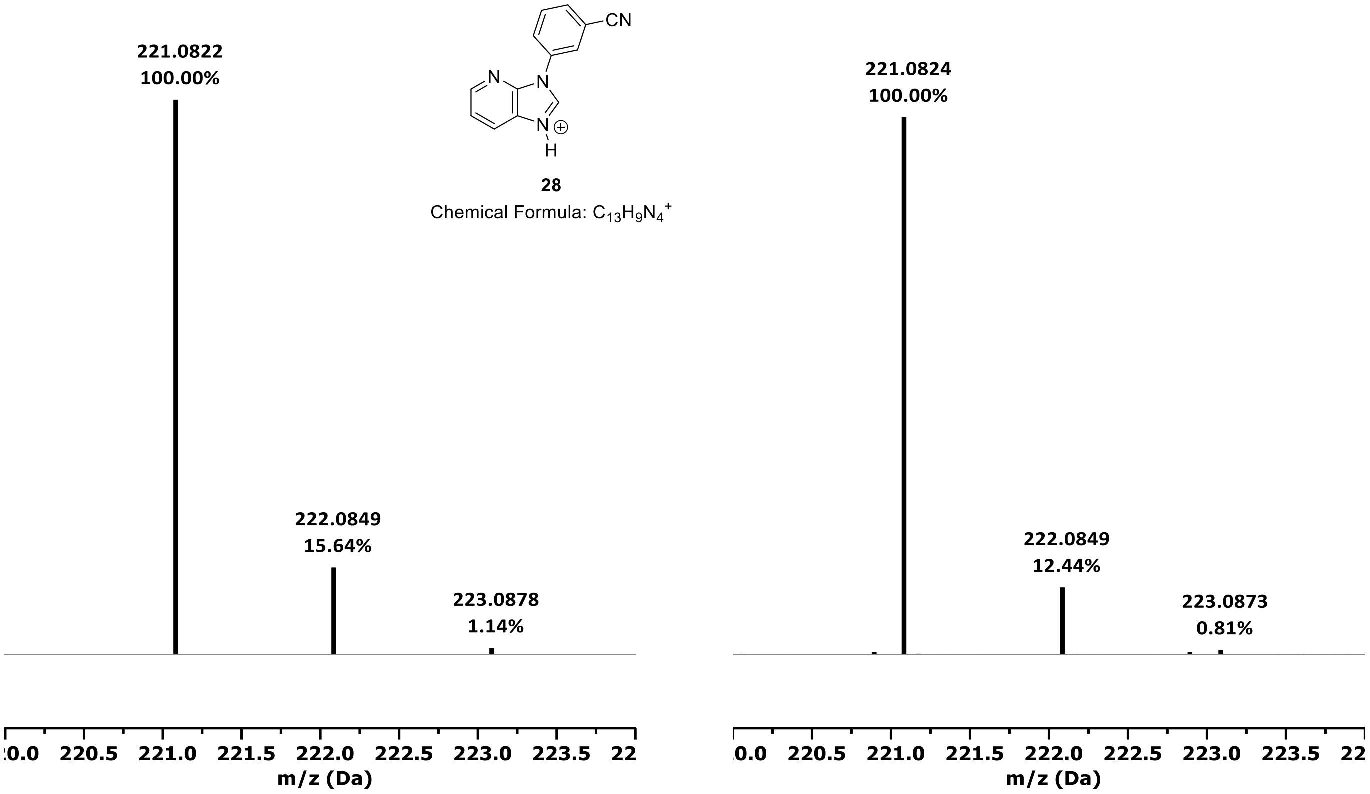

Figure S109. *ESI-HRMS spectrum of* ***28****, predicted (left) and measured (right)*

ESI-HRMS spectra

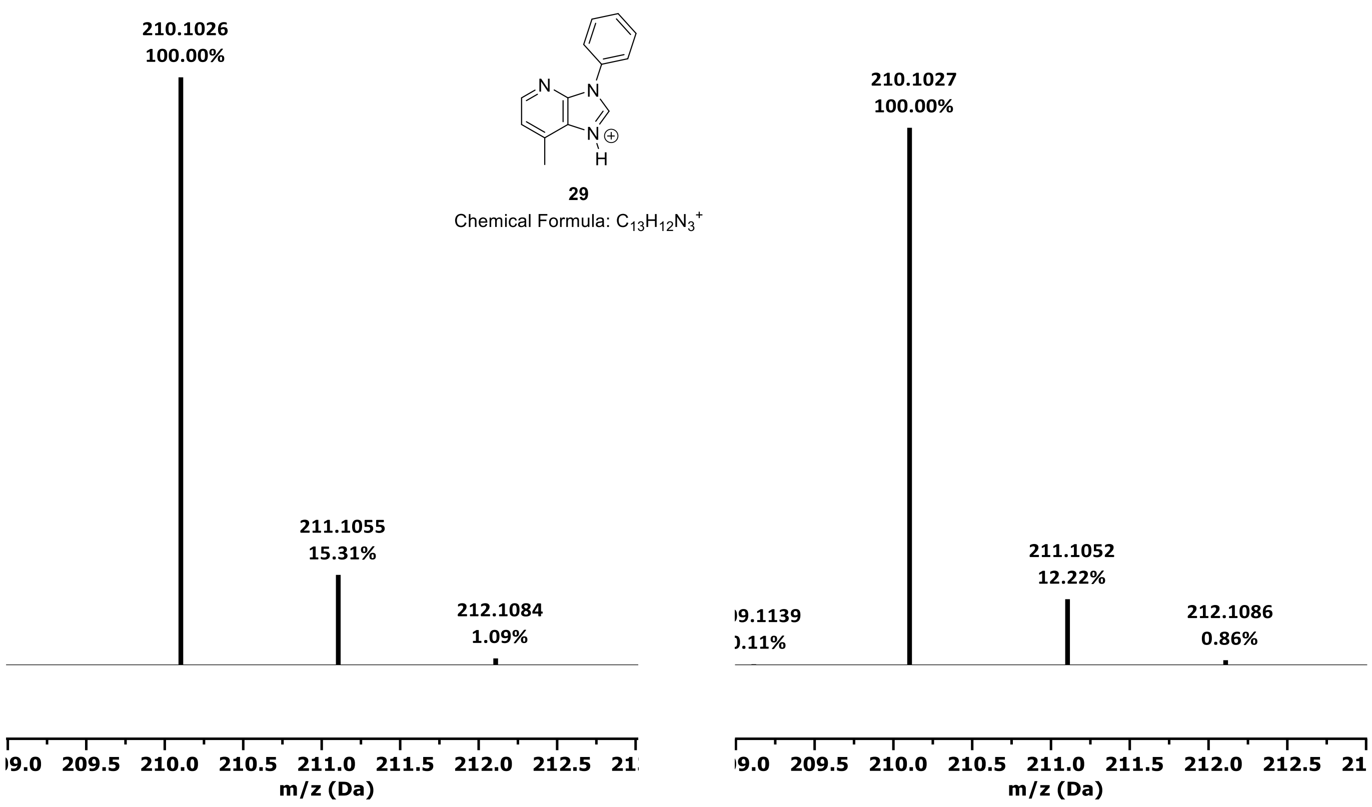

Figure S110. *ESI-HRMS spectrum of* ***29****, predicted (left) and measured (right)*

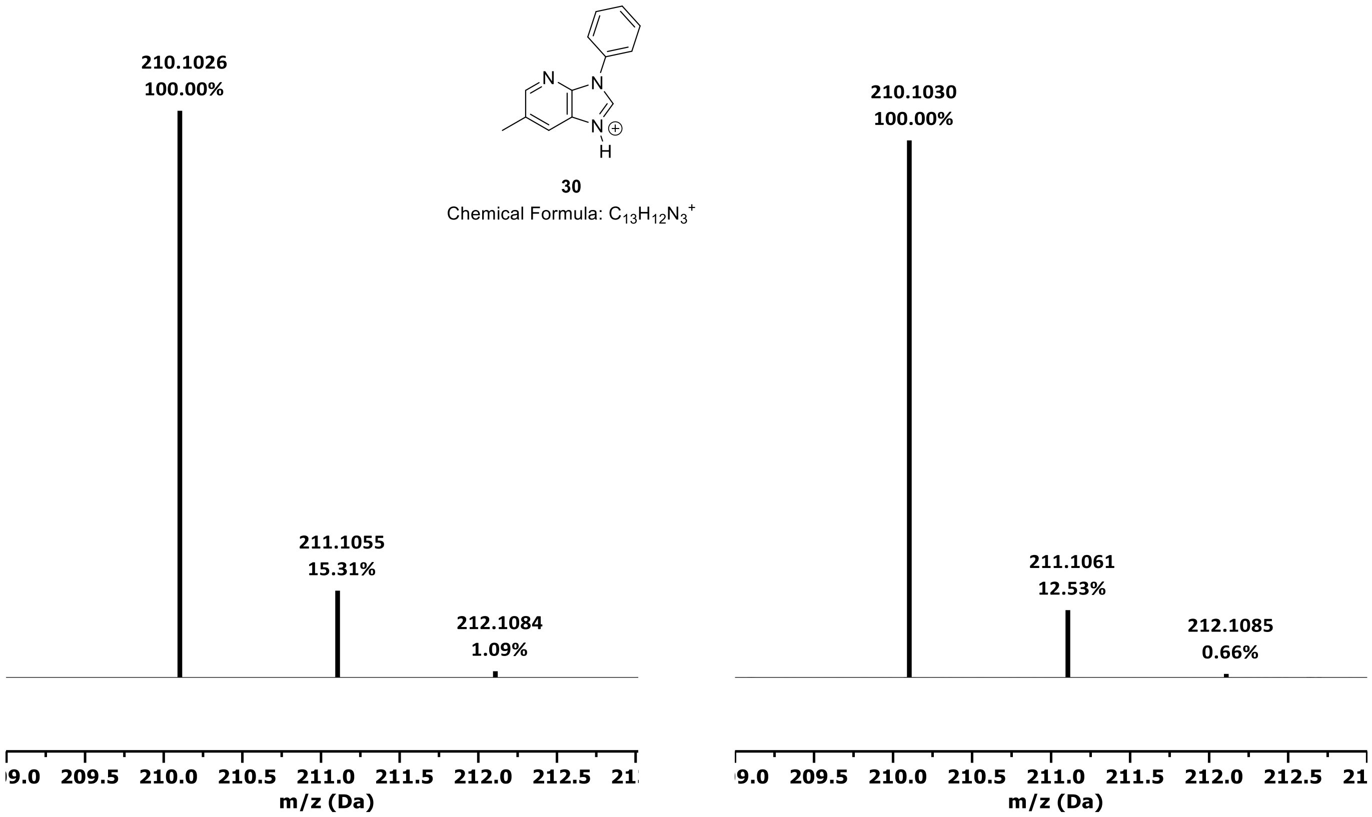

Figure S111. *ESI-HRMS spectrum of* ***30****, predicted (left) and measured (right)*

ESI-HRMS spectra

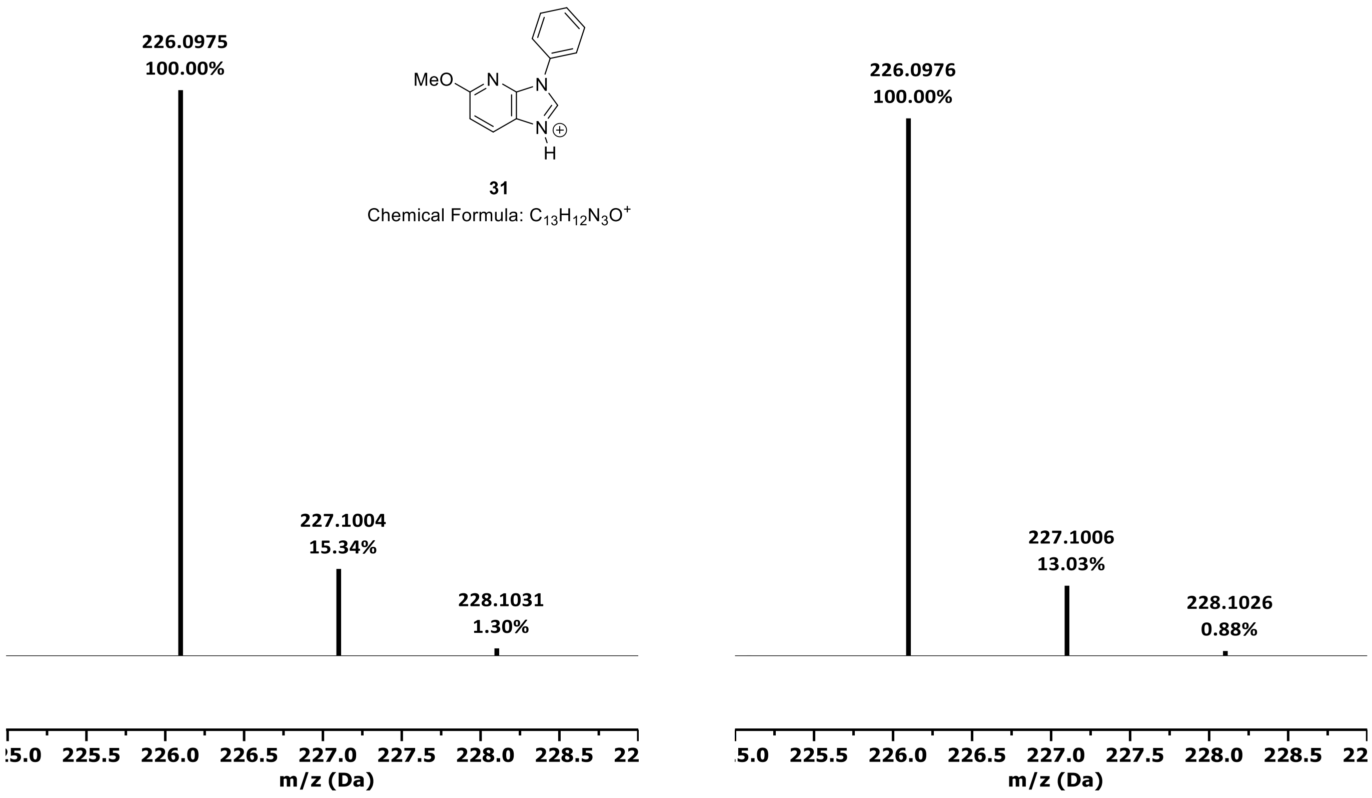

Figure S112. *ESI-HRMS spectrum of **31**, predicted (left) and measured (right)*

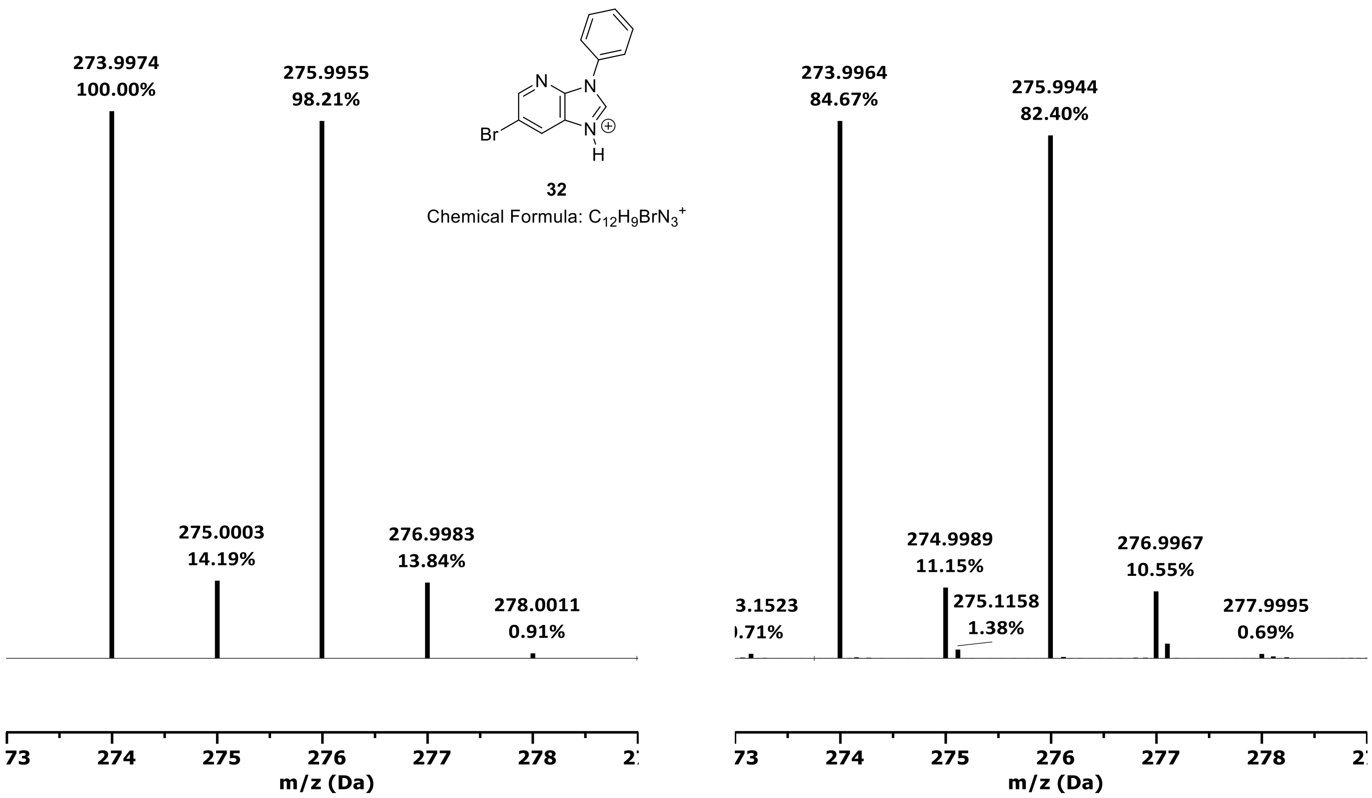

Figure S113. *ESI-HRMS spectrum of **32**, predicted (left) and measured (right)*

X-ray crystallography

Table S1. Crystal data, data collection and structure refinement details for intermediates

**Compound**	**2i**	**3i**	**4i**	**6i**	**11i**	**13i**	**14i**
**Formula**	$C_{13}H_{13}N_3O_2$	$C_{12}H_{11}N_3O_3$	$C_{12}H_{11}N_3O_3$	$C_{14}H_{15}N_3O_5$	$C_{12}H_8F_3N_3O_3$	$C_{11}H_8FN_3O_2$	$C_{11}H_8FN_3O_2$
**Formula weight**	243.26	245.24	245.24	305.29	299.21	233.20	233.20
**Crystal system**	triclinic	monoclinic	monoclinic	monoclinic	monoclinic	orthorhombic	monoclinic
**Space group**	P-1	$P2_1/n$	$P2_1/n$	$P2_1/n$	C2/c	Fdd2	P2/n
**a(Å)**	6.7973(6)	16.35198(17)	5.3510(3)	13.217(2)	11.7824(4)	42.5338(6)	15.3111(5)
**b(Å)**	7.7939(5)	4.04977(4)	14.5790(8)	7.1775(10)	8.5471(3)	25.0760(5)	3.80017(11)
**c(Å)**	22.523(3)	17.66105(18)	14.0979(8)	15.921(3)	24.0048(9)	3.73409(6)	18.1614(5)
**α(º)**	89.663(7)	90	90	90	90	90	90
**β(º)**	88.324(9)	104.1971(11)	98.161(5)	112.38(2)	96.824(4)	90	102.756(3)
**γ(º)**	76.935(7)	90	90	90	90	90	90
**V(Å^3)**	1161.8(2)	1133.82(2)	1088.67(11)	1396.6(4)	2400.29(15)	3982.70(12)	1030.64(5)
**Z**	4	2	4	4	8	16	4
**D_x(g cm^{-3})**	1.391	1.437	1.496	1.452	1.656	1.556	1.503
**F(000)**	512	512	512	640	1216	1920	480
**μ(mm^{-1})**	0.097	0.888	0.111	0.112	0.151	1.052	1.017
**Reflections:**							
**collected**	8635	11714	4302	5905	5016	18020	4546
**unique (R_{int})**	7498 (0.0704)	2340 (0.0183)	2297 (0.0148)	3012 (0.0146)	2364 (0.0160)	2087 (0.0336)	2113 (0.0183)
**with I>2σ(I)**	4573	2155	1997	2116	2076	2018	1765
**R(F) [I>2σ(I)]**	0.1327	0.0377	0.0346	0.0440	0.0322	0.0293	0.0444
**wR(F^2) [I>2σ(I)]**	0.3156	0.1111	0.0833	0.0960	0.0758	0.0786	0.1264
**R(F) [all data]**	0.1869	0.0397	0.0425	0.0727	0.0391	0.0302	0.0529
**wR(F^2) [all data]**	0.3675	0.1139	0.0893	0.1126	0.0794	0.0796	0.1355
**Goodness of fit**	1.078	1.074	1.058	1.037	1.067	1.087	1.030
**max/min Δρ (e·Å^{-3})**	0.75/-0.56	0.16/-0.13	0.26/-0.28	0.18/-0.15	0.24/-0.20	0.12/-0.17	0.18/-0.16
**CCDC deposition number**	2245087	2245094	2245093	2245085	2245088	2245095	2245097

Table S1. Crystal data, data collection and structure refinement details for intermediates, continued

**Compound**	**16i**	**17i**	**20i**	**26i**	**29i**	**30i**	**32i**
**Formula**	$C_{11}H_7F_2N_3O_2$	$C_{11}H_7ClFN_3O_2$	$C_{11}H_8BrN_3O_2$	$C_{12}H_{11}N_3O_2S$	$C_{12}H_{11}N_3O_2$	$C_{12}H_{11}N_3O_2$	$C_{11}H_8BrN_3O_2$
**Formula weight**	251.20	267.65	294.11	261.30	229.24	229.24	294.11
**Crystal system**	monoclinic	triclinic	triclinic	triclinic	triclinic	monoclinic	monoclinic
**Space group**	$P2_1/n$	P-1	P-1	P-1	P-1	C2/c	$P2_1/c$
**a(Å)**	8.72601(16)	7.2961(5)	7.4481(5)	7.1848(4)	7.5430(4)	11.6845(4)	5.6473(6)
**b(Å)**	5.47513(10)	7.7492(6)	7.7228(5)	7.5764(4)	7.5797(4)	8.9441(3)	17.341(2)
**c(Å)**	21.8635(4)	11.1003(8)	10.7010(5)	12.5326(7)	10.9898(6)	22.1069(7)	11.4252(11)
**α(º)**	90	102.996(6)	101.967(5)	106.070(5)	100.328(4)	90	90
**β(º)**	99.6342(17)	96.950(6)	93.823(5)	93.844(5)	103.328(5)	100.923(3)	92.141(8)
**γ(º)**	90	114.958(7)	111.579(6)	108.646(5)	111.907(5)	90	90
**V(Å^3)**	1029.82(3)	537.61(7)	552.99(6)	612.01(6)	542.21(5)	2268.48(13)	1118.1(2)
**Z**	4	2	2	2	2	8	4
**D_x(g cm^{-3})**	1.620	1.653	1.766	1.418	1.404	1.342	1.747
**F(000)**	512	272	292	272	240	960	584
**μ(mm^{-1})**	1.210	0.366	3.709	0.262	0.099	0.095	3.669
**Reflections:**							
**collected**	4125	3727	8211	11494	7934	6485	4246
**unique (R_{int})**	2078 (0.0163)	2254 (0.0167)	2206 (0.0251)	2452 (0.0205)	2337 (0.0184)	2008 (0.0225)	2352 (0.0227)
**with I>2σ(I)**	1896	1752	1969	2062	1662	1462	1576
**R(F) [I>2σ(I)]**	0.0402	0.0459	0.0279	0.0378	0.0505	0.0486	0.0397
**wR(F^2) [I>2σ(I)]**	0.1101	0.1094	0.0587	0.0922	0.1168	0.1281	0.0745
**R(F) [all data]**	0.0430	0.0632	0.0347	0.0465	0.0758	0.0704	0.0745
**wR(F^2) [all data]**	0.1146	0.1239	0.0619	0.0978	0.1323	0.1382	0.0881
**Goodness of fit**	1.054	1.037	1.052	1.052	1.039	1.074	1.087
**max/min Δρ (e·Å^{-3})**	0.21/-0.23	0.38/-0.27	0.48/-0.44	0.23/-0.31	0.23/-0.18	0.20/-0.16	0.39/-0.54
**CCDC deposition number**	2245096	2245086	2245089	2245090	2245092	2245091	2245084

X-ray crystallography

Table S2. Relevant geometrical parameters (Å, °) with s.u.'s in parentheses for the intermediates

Compound	2i	3i	4i	6i	11i	13i	14i
**N1-C2**	1.337(13)<br>1.323(12)	1.3265(16)	1.3264(17)	1.321(2)	1.3296(18)	1.328(2)	1.324(2)
**N1-C6**	1.369(11)<br>1.378(11)	1.3498(16)	1.3464(16)	1.334(2)	1.3465(18)	1.350(2)	1.3495(19)
**C6-N7**	1.361(12)<br>1.347(12)	1.3446(15)	1.3590(16)	1.351(2)	1.3582(18)	1.352(2)	1.3482(19)
**N7-C8**	1.369(11)<br>1.394(13)	1.4159(14)	1.4113(16)	1.406(2)	1.4068(17)	1.420(2)	1.408(2)
**C2-N1-C6**	117.8(9)<br>118.2(9)	118.96(11)	118.81(11)	118.93(15)	119.01(12)	119.02(16)	119.02(14)
**C6-N7-C8**	131.6(8)<br>133.0(8)	131.82(11)	131.62(11)	131.31(15)	131.09(12)	127.43(15)	130.39(13)
**N-H**	0.88<br>0.88	0.894(16)	0.873(17)	0.840(19)	0.841(17)	0.88(3)	0.88(2)
**H···O**	1.93<br>1.92	1.914(15)	1.911(16)	1.970(19)	1.958(16)	1.93(3)	1.95(2)
**N7···O51**	2.639(10)<br>2.630(10)	2.6504(14)	2.6382(14)	2.643(2)	2.6480(15)	2.632(2)	2.6335(19)
**N-H···O**	136<br>136	138.4(14)	139.8(14)	136.4(18)	138.6(14)	135(2)	134.3(17)
**A/B**	3.10(19)<br>2.90(5)	4.066(8)	7.66(7)	3.53(6)	7.53(8)	43.81(5)	27.43(6)

Table S2. Relevant geometrical parameters (Å, °) with s.u.'s in parentheses for the intermediates, continued

Compound	16i	17i	20i	26i	29i	30i	32i
**N1-C2**	1.3274(19)	1.323(3)	1.333(3)	1.325(2)	1.320(2)	1.324(2)	1.322(4)
**N1-C6**	1.3426(18)	1.339(3)	1.338(3)	1.3403(19)	1.330(2)	1.341(2)	1.350(4)
**C6-N7**	1.3586(17)	1.355(3)	1.358(3)	1.354(2)	1.352(2)	1.355(2)	1.352(4)
**N7-C8**	1.4070(17)	1.406(3)	1.403(3)	1.4103(19)	1.406(2)	1.411(2)	1.413(4)
**C2-N1-C6**	118.89(13)	119.0(2)	118.6(2)	119.19(14)	118.17(15)	118.86(16)	119.6(3)
**C6-N7-C8**	131.06(12)	130.8(2)	131.7(2)	130.60(13)	131.48(15)	131.63(18)	132.1(3)
**N-H**	0.867(19)	0.82(2)	0.79(2)	0.860(19)	0.89(2)	0.91(2)	0.77(3)
**H···O**	1.947(19)	1.97(2)	1.98(3)	1.962(19)	1.851(19)	1.87(2)	2.02(4)
**N7···O51**	2.6481(16)	2.655(3)	2.634(3)	2.6487(17)	2.5828(19)	2.628(2)	2.657(4)
**N-H···O**	137.1(15)	140(2)	140(2)	135.9(17)	138.0(16)	139(2)	140(4)
**A/B**	1.68(10)	4.79(9)	3.53(9)	6.86(9)	4.77(10)	1.48(13)	9.0(2)

X-ray crystallography

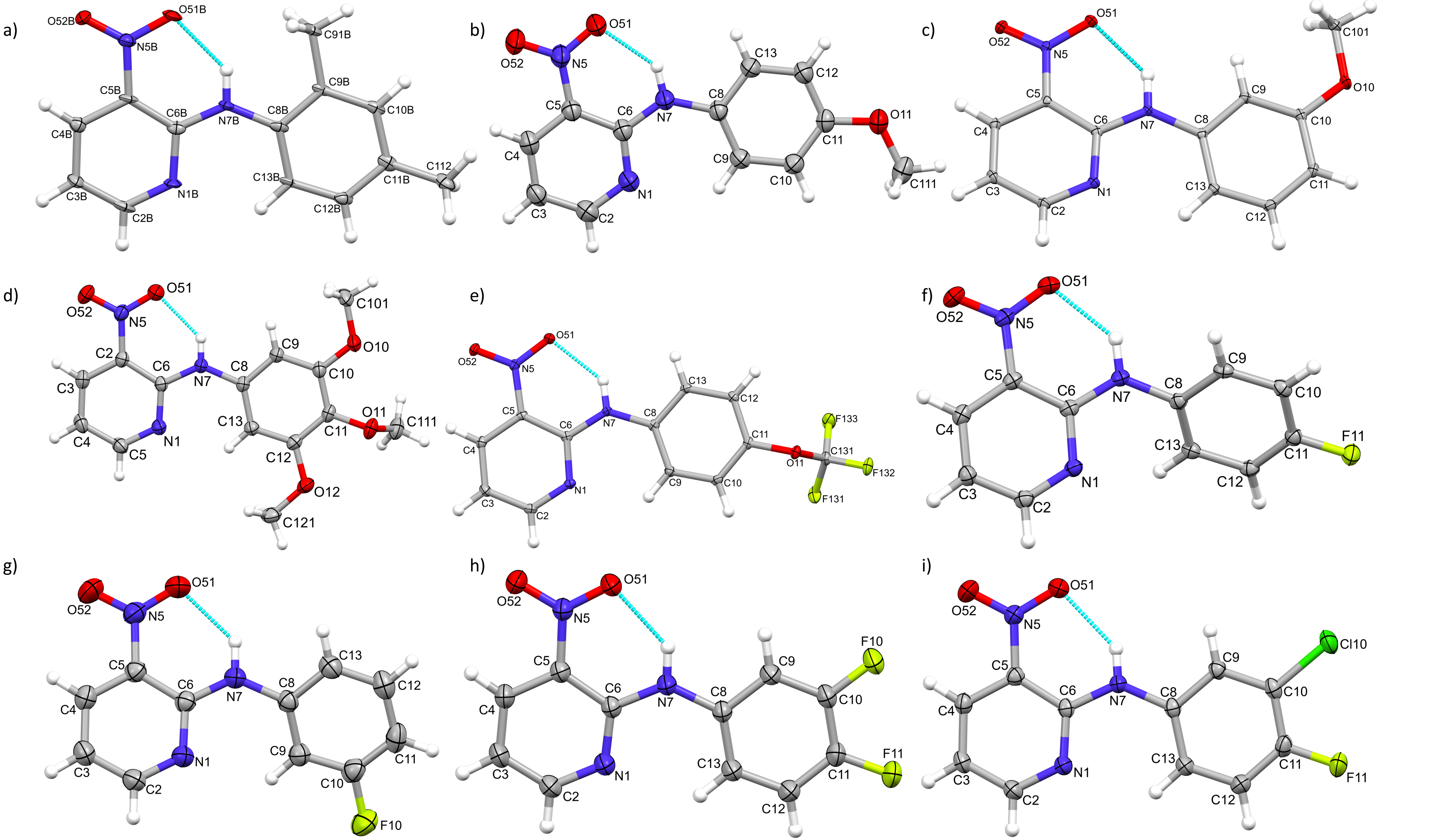

Figure S114. *Perspective views of the intermediates* ***2i*** *(a),* ***3i*** *(b),* ***4i*** *(c),* ***6i*** *(d),* ***11i*** *(e),* ***13i*** *(f),* ***14i*** *(g),* ***16i*** *(h),* ***17i*** *(i). Ellipsoids are drawn at the 50% probability level, hydrogen atoms are shown as spheres of arbitrary radii*

X-ray crystallography

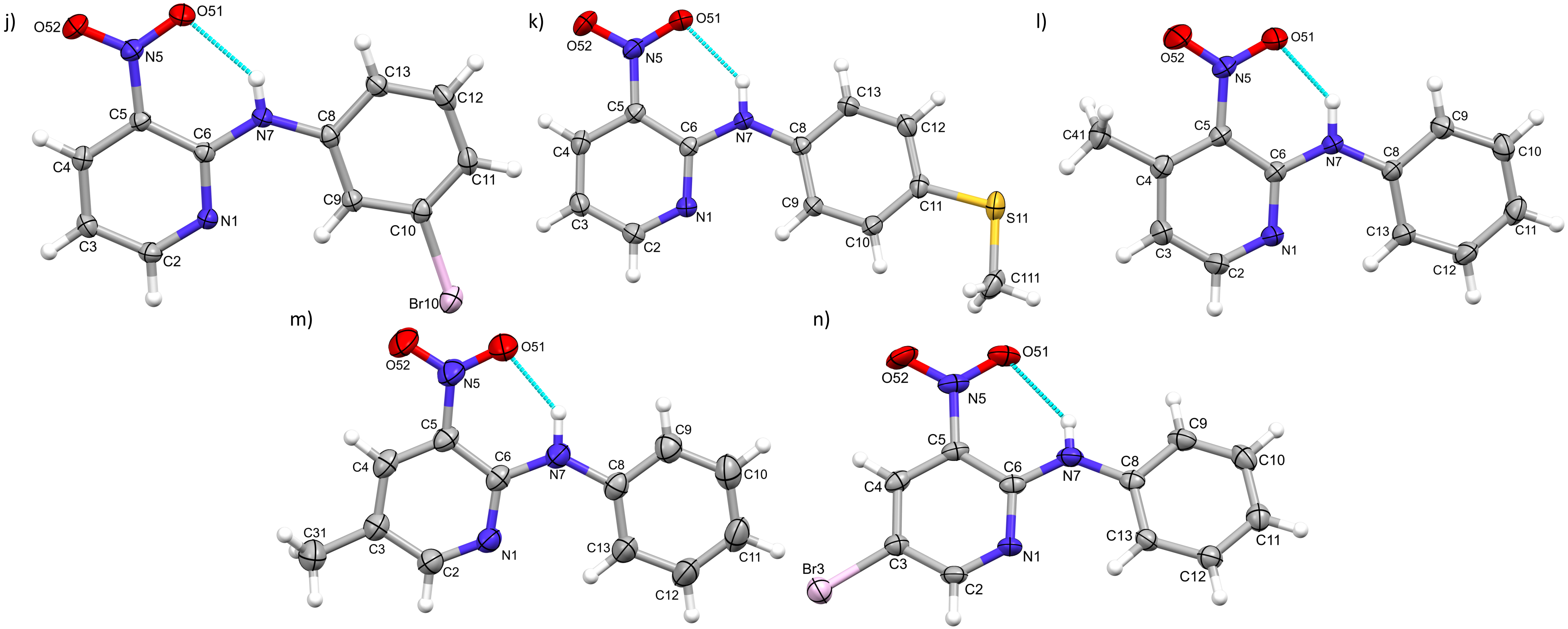

Figure S115. *Perspective views of the intermediates* ***20i*** *(j),* ***26i*** *(k),* ***29i*** *(l),* ***30i*** *(m),* ***32i*** *(n). Ellipsoids are drawn at the 50% probability level, hydrogen atoms are shown as spheres of arbitrary radii*

Table S3. Crystal data, data collection and structure refinement details for the target pyridoimidazoles

Compound	3	7	12	18	19	25
Formula	$C_{12}H_9N_3O$	$C_{18}H_{13}N_3O$	$C_{13}H_8F_3N_3O$	$C_{13}H_8F_3N_3$	$C_{12}H_8BrN_3$	$C_{14}H_{14}N_4$
Formula weight	211.22	287.31	279.22	263.22	274.12	238.29
Crystal system	monoclinic	orthorhombic	monoclinic	triclinic	monoclinic	monoclinic
Space group	$P2_1/c$	Pbca	$P2_1/n$	P-1	$P2_1/c$	$P2_1/c$
a(Å)	6.7028(2)	18.1516(8)	12.4679(9)	12.8856(4)	21.9072(2)	9.7938(3)
b(Å)	16.1733(5)	7.9990(3)	4.0143(3)	13.9152(2)	3.92885(5)	12.0354(4)
c(Å)	9.4556(3)	19.5466(10)	23.765(3)	32.9006(7)	25.0862(2)	10.3182(4)
α(º)	90	90	90	99.6488(16)	90	90
β(º)	97.095(3)	90	100.756(9)	98.952(2)	93.0214(9)	90.177(3)
γ(º)	90	90	90	95.7476(18)	90	90
V(Å^3)	1017.20(5)	2838.1(2)	1168.54(19)	5696.5(2)	2156.17(4)	1216.22(7)
Z	4	8	4	20	8	4
D_x(g cm^{-3})	1.379	1.345	1.587	1.535	1.689	1.301
F(000)	440	1200	568	2680	1088	504
μ(mm^{-1})	0.092	0.086	0.137	0.129	4.973	0.082
Reflections:						
collected	3600	14448	3658	48250	2064211494	7255
unique (R_{int})	1972 (0.0127)	2992 (0.0717)	2242 (0.0303)	20068 (0.0362)	4217 (0.0231)	2451 (0.0232)
with I>2σ(I)	1725	1921	1873	15447	3911	1950
R(F) [I>2σ(I)]	0.0335	0.0882	0.0474	0.1130	0.0341	0.0487
wR(F^2) [I>2σ(I)]	0.0796	0.2082	0.1410	0.2278	0.0931	0.1342
R(F) [all data]	0.0398	0.1245	0.0566	0.1387	0.0365	0.0613
wR(F^2) [all data]	0.0838	0.2371	0.1542	0.2380	0.0953	0.1456
Goodness of fit	1.029	1.051	1.050	1.150	1.079	1.033
max/min Δρ (e·Å^{-3})	0.20/-0.22	0.40/-0.29	0.22/-0.27	1.13/-0.60	0.54/-0.51	0.58/-0.19
CCDC deposition number	2245872	2245876	2245875	2245874	2246300	2245873

X-ray crystallography

Table S4. Relevant geometrical parameters (Å, °) with s.u.'s in parentheses for the target pyridoimidazoles

Compound	3	7	12	18	19	25
**N1-C2**	1.3681(15)	1.377(4)	1.373(2)	1.379(7)	1.374(3)<br>1.367(3)	1.360(2)
**N1-C9**	1.3868(15)	1.378(4)	1.383(2)	1.395(6)	1.388(3)<br>1.389(3)	1.381(2)
**N1-C10**	1.4316(15)	1.420(4)	1.425(20	1.420(5)	1.423(3)<br>1.426(3)	1.431(2)
**C2-N3**	1.3105(16)	1.294(4)	1.306(2)	1.307(8)	1.302(3)<br>1.303(3)	1.302(2)
**N3-C4**	1.3877(16)	1.385(5)	1.381(2)	1.394(6)	1.396(3)<br>1.390(3)	1.385(2)
**C2-N1-C9**	105.67(10)	105.0(3)	105.46(15)	105.3(3)	105.47(19)<br>105.86(18)	105.29(14)
**C2-N3-C4**	104.46(10)	104.7(3)	104.11(16)	104.2(4)	104.2(2)<br>104.31(19)	103.75(14)
**C12-C13-C14**	119.14(11)	121.2(3)	118.42(19)	119.8(6)	121.7(2)<br>121.7(2)	117.29(15)
**C2-N1-C10-C11**	45.23(17)	41.1(4	41.7(3)	24(13)	43.7(4)<br>38.3(3)	68.6(2)
**N1-C2**	1.3681(15)	1.377(4)	1.373(2)	1.379(7)	1.374(3)<br>1.367(3)	1.360(2)
**N1-C9**	1.3868(15)	1.378(4)	1.383(2)	1.395(6)	1.388(3)<br>1.389(3)	1.381(2)

X-ray crystallography

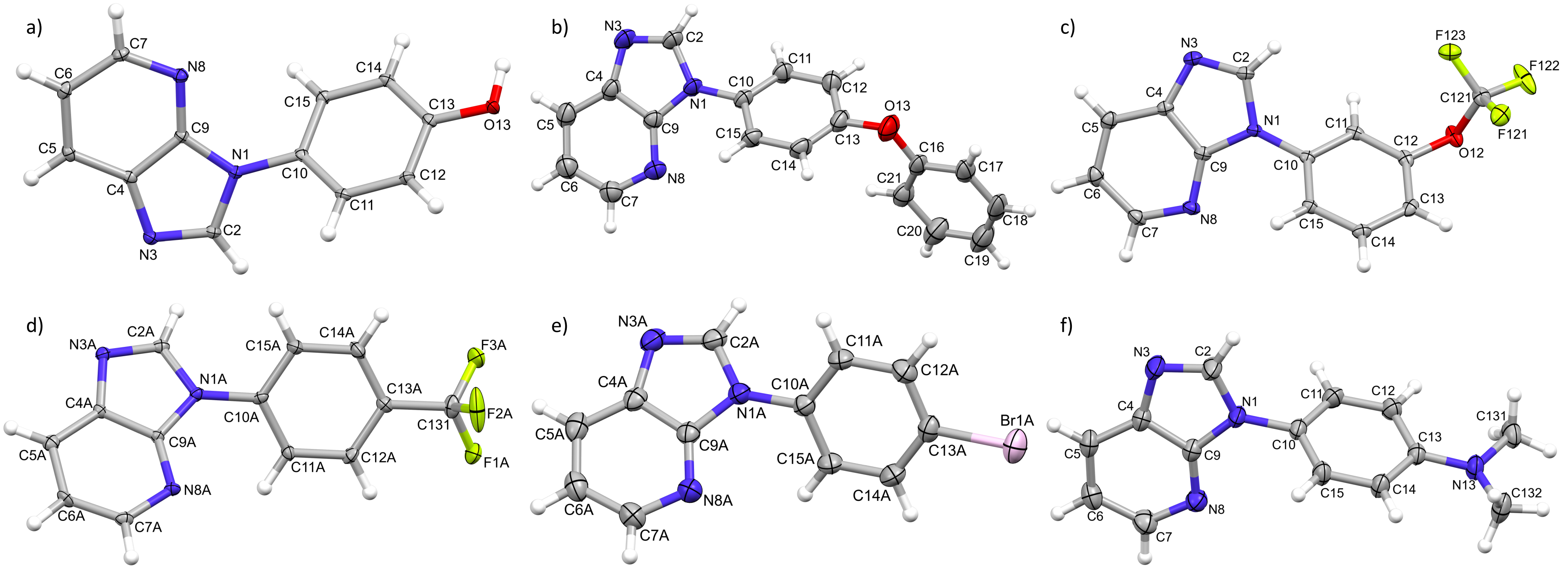

Figure S116. *Perspective views of the target pyridoimidazoles **3** (a), **7** (b), **12** (c), **18** (d), **19** (e), **25** (f). Ellipsoids are drawn at the 50% probability level, hydrogen atoms are shown as spheres of arbitrary radii*

X-ray crystallography

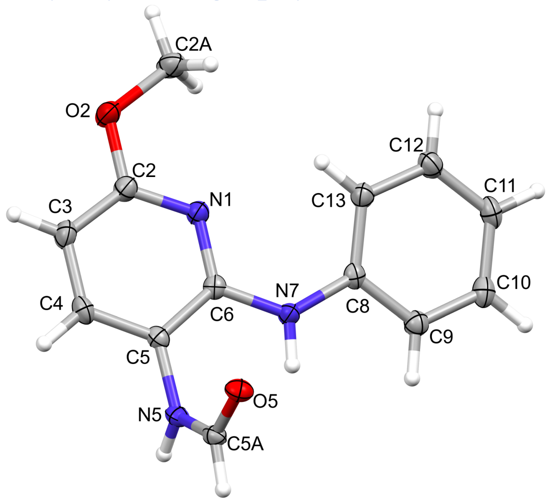

Figure S117. *Perspective views of the intermediate **31i-CHO**. Ellipsoids are drawn at the 50% probability level, hydrogen atoms are shown as spheres of arbitrary radii*

Table S5. Relevant geometrical parameters (Å, °) with s.u.'s in parentheses for the intermediate **31i-CHO**

Compound	31i-CHO
**N1-C2**	1.327(2)
**N1-C6**	1.344(2)
**C6-N7**	1.3770(19)
**N7-C8**	1.409(2)
**C2-N1-C6**	118.15(13)
**C6-N7-C8**	130.281(3)
**N5-H5**	0.87(3)
**H5···O5^{i}**	2.02(3)
**N5-H5···O5^{i}**	149(2)
**N7-H7**	0.89(2)
**H7···O5^{ii}**	2.48(2)
**N7···O5^{ii-}**	3.3124(17)
**N-H···O**	156.1(18)
**A/B**	15.87(9)

Table S6. Crystal data, data collection and structure refinement details for the intermediate **31i-CHO**

Compound	31i-CHO
**Formula**	$C_{13}H_{13}N_3O_2$
**Formula weight**	243.26
**Crystal system**	monoclinic
**Space group**	$P2_1$
**a(Å)**	9.9589(4)
**b(Å)**	4.4082(2)
**c(Å)**	14.0320(6)
**α(º)**	90
**β(º)**	108.1580(10)
**γ(º)**	90
**V(Å^3)**	585.34(4)
**Z**	2
**D_x(g cm^{-3})**	1.380
**F(000)**	256
**μ(mm^{-1})**	0.787
**Reflections:**	
**collected**	11888
**unique (R_{int})**	2122 (0.0223)
**with I>2σ(I)**	2110
**R(F) [I>2σ(I)]**	0.0243
**wR(F^2) [I>2σ(I)]**	0.0623
**R(F) [all data]**	0.0247
**wR(F^2) [all data]**	0.0624
**Goodness of fit**	1.109
**max/min Δρ (e·Å^{-3})**	0.18/-0.14
**CCDC deposition**	2502793

References

[1] Rigaku Oxford Diffraction (**2015**) CrysAlis PRO (Version 1.171.38.41)

[2] G. M. Sheldrick, *Acta Cryst.* **2015**, A71, pp 3-8

[3] G. M. Sheldrick, *Acta Cryst.* **2015**, C71, pp 3-8

[4] T. Kovač, M. Oklobdžija, G. Comisso, E. Decorte, T. Fajdiga, F. Moimas, C. Angeli, F. Zonno, R. Toso, V. Šunjić, *J. Heterocycl. Chem.* ***1983***, 20, pp 1339-1349
